# Design, Synthesis, and Antiproliferative Activity of Novel Indole/1,2,4-Triazole Hybrids as Tubulin Polymerization Inhibitors

**DOI:** 10.3390/ph18020275

**Published:** 2025-02-19

**Authors:** Esraa Mahmoud, Dalia Abdelhamid, Anber F. Mohammed, Zainab M. Almarhoon, Stefan Bräse, Bahaa G. M. Youssif, Alaa M. Hayallah, Mohamad Abdel-Aziz

**Affiliations:** 1 Department of Pharmaceutical Chemistry, Faculty of Pharmacy, Deraya University, Minia 2460271, Egypt; esraataha505@yahoo.com; 2 Department of Medicinal Chemistry, Faculty of Pharmacy, Minia University, Minia 2431436, Egypt; dalia_abdelhameed@mu.edu.eg (D.A.); abulnil@hotmail.com (M.A.-A.); 3 Department of Pharmaceutical Organic Chemistry, Faculty of Pharmacy, Assiut University, Assiut 71526, Egypt; anber_pharm_2006@yahoo.com (A.F.M.); bgyoussif2@gmail.com (B.G.M.Y.); 4 Department of Chemistry, College of Science, King Saud University, Riyadh 11451, Saudi Arabia; zalmarhoon@ksu.edu.sa; 5 Institute of Biological and Chemical Systems, IBCS-FMS, Karlsruhe Institute of Technology, 76131 Karlsruhe, Germany; 6 Department of Pharmaceutical Chemistry, Faculty of Pharmacy, Sphinx University, New-Assiut 71515, Egypt

**Keywords:** NCI, cancer, CA-4, tubulin, colchicine, anticancer

## Abstract

**Background/Objectives:** New indole/1,2,4-triazole hybrids were synthesized and tested for antiproliferative activity against the NCI 60 cell line as tubulin polymerization inhibitors. **Methods:** All final compounds, **6a**–**j** and **7a**–**j** were evaluated at a single concentration of 10 µM against a panel of sixty cancer cell lines. **Results:** Compounds **7a**–**j**, featuring the NO-releasing oxime moiety, exhibited superior anticancer activity to their precursor ketones **6a**–**j** across all tested cancer cell lines. Compounds **6h**, **7h**, **7i**, and **7j** were chosen for five-dose evaluations against a comprehensive array of 60 human tumor cell lines. The data showed that all tested compounds had significant anticancer activity throughout the nine tumor subpanels studied, with selectivity ratios ranging from 0.52 to 2.29 at the GI_50_ level. Compounds **7h** and **7j** showed substantial anticancer effectiveness against most cell lines across nine subpanels, with GI_50_ values ranging from 1.85 to 5.76 µM and 2.45 to 5.23 µM. Compounds **6h**, **7h**, **7i**, and **7j** were assessed for their inhibitory effects on tubulin polymerization. **Conclusions:** The results showed that compound **7i**, an oxime-based derivative, was the most effective at blocking tubulin, with an IC_50_ value of 3.03 ± 0.11 µM. This was compared to the standard drug CA-4, which had an IC_50_ value of 8.33 ± 0.29 µM. Additionally, cell cycle analysis and apoptosis assays were performed for compound **7i**. Molecular computational investigations have been performed to examine the binding mode of the most effective compounds to the target enzyme.

## 1. Introduction

Cancer is a set of disorders that spread to other regions of the body [1,2]. Cancer is the world’s second greatest cause of death, after cardiovascular diseases, and hence a significant health burden [3,4,5]. There are numerous successful cancer treatment options, but some have adverse effects. For example, certain cancer cells develop resistance to medications, radiotherapy has limitations, and surgery is frequently required. This demonstrates the importance of finding alternative, successful treatments that work in different ways [6,7,8].

**Figure 1:**
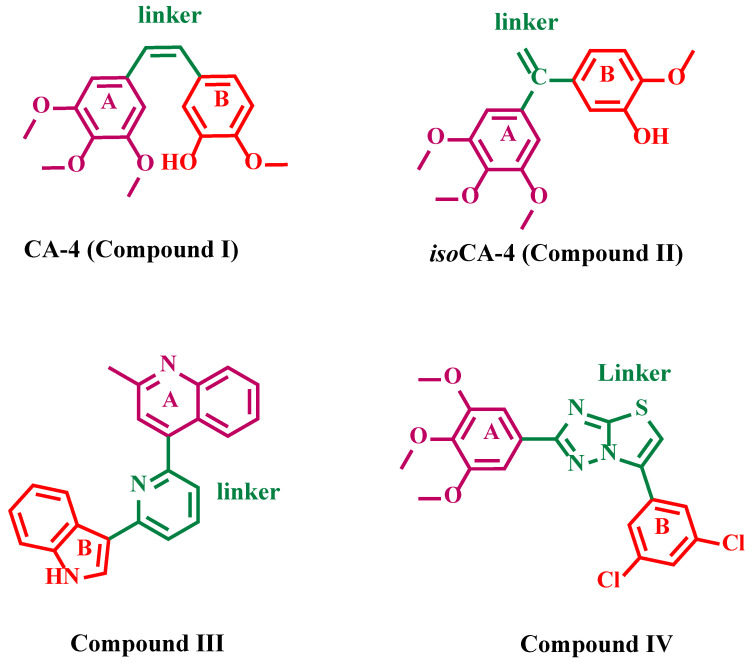
Structure of CA-4 (**I**), isoCA-4 (**II**), and compounds **III** and **IV**.

Tubulin has been identified as an important therapeutic target for cancer treatment due to its role in cell division, signaling, protein transport, and cellular structure maintenance [9,10,11]. Researchers have investigated numerous tubulin inhibitors with various scaffolds, most notably combretastatin A-4 [CA-4 (**I**), Figure 1], a prominent tubulin polymerization inhibitor that binds to the colchicine site in tubulin and effectively suppresses cancer cell proliferation at low nanomolar concentrations [12,13]. However, the isomerization of the cis-double bond to a more stable and inactive trans-form hindered its potential therapeutic application [14,15,16]. Consequently, there is growing interest in developing innovative tubulin inhibitors for cancer treatment.

Many indole-based compounds are known to be potent bioactive molecules, particularly as antitumor agents with strong tubulin polymerization inhibition [17,18,19]. Pecnard et al. [20] developed a series of cyclic bridging analogs of isocombretastatin A-4 (isoCA-4, compound **II**, Figure 1) with phenyl or pyridine linkers. A study of the structure-activity relationship (SAR) reveals that the presence of quinaldine (ring A), pyridine (linker), and indole (ring B) in the same molecule is required for its cytotoxic activity. Compound **III** (Figure 1) had the most significant antiproliferative effect against various cancer cell lines among all evaluated compounds. Compound **III** showed strong antiproliferative activity against the multi-drug resistant K562R cell line; it was 1.5 times and 12 times more effective than the reference compounds, isoCA-4 and CA-4. Compound **III** efficiently reduced tubulin polymerization in both in vitro and cellular contexts, producing cell cycle arrest in the G2/M phase. A molecular docking study of **III** in the colchicine binding site of the tubulin β subunit showed that the overall binding mode was the same as that found for isoCA-4. The quinaldine ring system fit into the lipophilic pocket normally occupied by the trimethoxyphenyl nucleus of isoCA-4, and the indole ring was placed similarly to isoCA-4′s B ring [20].

Researchers conducted numerous studies to constrain the cis-orientation of the ethylene bond in CA-4 by substituting it with rigid heterocyclic rings, particularly triazoles [12,21]. Additionally, many studies have documented significant antiproliferative activity of numerous 1,2,4-triazole derivatives as tubulin inhibitors [22,23,24]. Moreover, derivatives of 1,2,4-triazole have been thoroughly investigated for their antibacterial, antifungal, and antiviral activities, especially as inhibitors of metallo-β-lactamases [25,26,27].

In a recent study [28], the authors reported a group of 6-aryl-2-(3,4,5-trimethoxyphenyl)thiazolo [3,2-b][1,2,4]triazoles that might be able to reduce tubulin polymerization by attaching to the colchicine domain on tubulin. Among the compounds produced, **IV** (Figure 1) demonstrated significant efficacy against the SGC-7901 cancer cell line, with an IC_50_ value of 0.21 µM. The data indicated that compound **IV** may function as an antitubulin, inducing cell cycle arrest in the G2/M phase.

**Figure 2:**
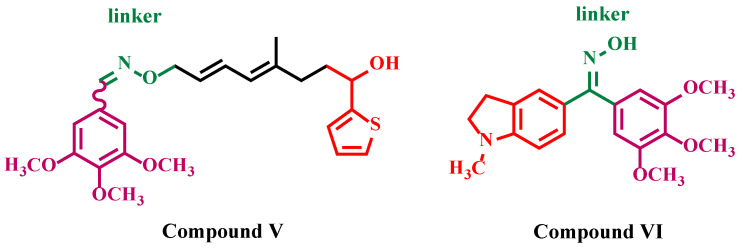
Structure of oxime-based binding tubulin compounds **V** and **VI**.

Oximes are hydroxy-imine derivatives popular in medicinal chemistry because they are simple to synthesize from carbonyl compounds and have diverse biological effects [29,30,31]. Concerning tubulin-binding oximes, the curacin A oxime analog **V** (Figure 2) blocks tubulin polymerization like CA-4, with the oxime group acting as a bioisosteric substitute for a (Z)-alkene group [32]. Moreover, diaryl methyl oxime **VI** (Figure 2), which has an indole ring, has demonstrated significant inhibitory effects on tubulin polymerization [33].

**Figure 3:**
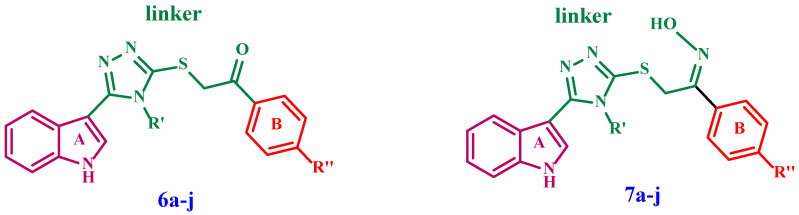
Structures of new targets **6a**–**j** and **7a**–**j**.

Inspired by previous findings of indole and/or 1,2,4-triazole derivatives as tubulin polymerization inhibitors and in continuation of our efforts to develop novel antitumor agents [34,35,36,37,38], we aim to combine these two moieties into a unique molecular framework to develop a novel series of indole-triazole hybrids **6a**–**j** and **7a**–**j** (Figure 3). The new compounds comprise the three main parts of CA-4: ring A, which is an indole ring; ring B, which is a substituted phenyl group; and the linker, which is the 1,2,4-triazole moiety. We employed oxime formation (**7a**–**j**) to enhance the efficacy of **6a**–**j** analogs. This mechanism could either augment chemotherapeutic activity through nitric oxide release or enhance binding to the colchicine binding site via hydrogen bond formation. This study presents comprehensive synthesis pathways, antiproliferative properties, and tubulin polymerization inhibitory effects.

## 2. Results and Discussion

### 2.1. Chemistry

**Scheme 1:**
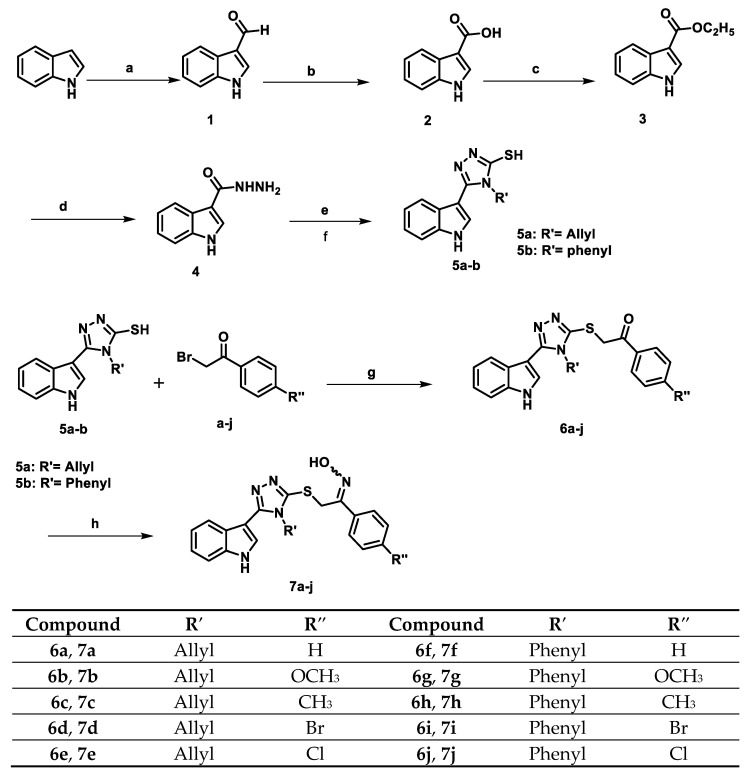
Synthesis of target compounds **6a**–**j** and **7a**–**j**.

Scheme 1 outlines the synthetic pathway for synthesizing new compounds **6a**–**j** and **7a**–**j**. Using POCl_3_ and DMF in the Vielsmeier-Haack reaction on indole produced a high yield of 1*H*-indole-3-carbaldehyde (**1**) [39]. KMnO_4_ in acetone oxidized 1*H*-indole-3-carbaldehyde (**1**), forming its acid counterpart (**2**) [40]. The oxidation product **2** was mixed with an excess of ethanol and a catalytic amount of concentrated H_2_SO_4_ to synthesize the ethyl ester derivative (**3**) [41]. 1*H*-indole-3-carbohydrazide (**4**) was synthesized by stirring the ethyl ester (**3**) with hydrazine monohydrate (95%) [42]. After refluxing product **4** with allyl/phenyl isothiocyanate and adding 2 N KOH solution, the thiol derivatives **5a**–**b** were formed in good yields [42]. The target ketone compounds **6a**–**j** were synthesized by combining compounds **5a**–**b**, phenacyl bromide derivatives, and TEA in acetonitrile.

Compounds **6a**–**j** were validated by ^1^H NMR, ^13^C NMR spectroscopy, and elemental microanalysis. As an example, the ^1^H NMR spectrum of **6b** exhibited a typical pattern of the indole scaffold at their anticipated chemical shifts. Also, the spectrum displayed a distinctive singlet signal at δ = 3.85 ppm for O-CH_3_ and δ = 4.94 ppm for the methylene S-CH_2_ linker, confirming the alkylation process. The signals characteristic of the allyl moiety in 6b manifest as a doublet at δ: 4.82 ppm corresponding to (N-CH_2_-CH=CH_2_), a doublet of doublets in the range of δ: 4.90–4.74 ppm and δ: 5.23–4.26 ppm for (N-CH_2_-CH=CH_2_), and a multiplet signal at δ: 6.10–6.01 ppm associated with (N-CH_2_-CH=CH_2_). The ^13^C NMR spectrum of compound **6b** exhibited a signal of C=O in the range of δ: 194.48–193.00 ppm; furthermore, all other carbon signals appeared at their anticipated chemical shifts.

**Reagent and reaction conditions:** (a) DMF, POCl_3_, ice, 8 h, 84% yield; (b) KMnO_4_, acetone, rt, 12–24 h, 70% yield; (c) absolute ethanol, H_2_SO_4_, 70 °C, 20 h; (d) ethanol, NH_2_-NH_2_·H_2_O, 150 °C, 8 h; (e) allyl/phenyl isothiocyanate, 150 °C, 3 h; (f) KOH, 150 °C, overnight; (g) TEA, acetonitrile, 100 °C, 4 h; and (h) NH_2_OH.HCl, CH_3_COONa, 50–70 °C, 2–8 h.

Oximes **7a**–**j** were synthesized by refluxing the corresponding ketones **6a**–**j** with hydroxylamine hydrochloride and anhydrous sodium acetate in absolute ethanol, resulting in acceptable yields of the target compounds **7a**–**j**. The chemical structure of oximes **7a**–**j** was confirmed by ^1^H NMR, ^13^C NMR spectroscopy, and elemental analysis. The ^1^H NMR spectrum of compound **7c** showed a singlet signal for methyl protons at δ: 2.24 ppm and a significant singlet signal at δ: 11.88 ppm, which corresponded to the OH group of the oxime moiety. The upfield shift of the S-CH_2_ linker adjacent to C=N from δ: 4.94 ppm to δ: 4.43 ppm is another distinguishing feature of the oxime spectrum. This occurs because the N atom in oximes is less electronegative than the O atom in ketones. All other protons manifest at their anticipated chemical changes. The ^13^C NMR of compound **7c** displayed a shift of C=O signals from δ: 194.48–193.00 ppm to δ: 152.18–152.63 ppm for the C=N group. All remaining carbons exhibit their anticipated chemical shifts. There is only one diastereomer detected.

### 2.2. Biology

#### 2.2.1. Evaluation of Anticancer Efficacy

##### In Vitro One Dose NCI Assay

The National Cancer Institute (NCI), Bethesda, USA, selected the final compounds **6a**–**j** and **7a**–**j** for in vitro anticancer screening [43,44,45]. An initial in vitro single-dose anticancer experiment was performed with the entire NCI 60 cell lines derived from nine tumor subpanel cancer cell lines. The results for each compound were illustrated as a mean graph showing the percentage growth of treated cells compared to untreated control cells (see Supplementary Materials). Table 1 displays the percentage of growth inhibition for the most active derivatives **6h**, **7h**, **7i**, and **7j**.

The NCI findings indicated weak to moderate anticancer efficacy for indole-N-allyl-triazole-phenyl ethanone derivatives (**6a**–**e**) and their corresponding oxime derivatives (**7a**–**e**). Compound **7d** exhibited modest anticancer efficacy against eleven cell lines, with growth inhibition percentage ranging from 34% to 47%. Compound **7e** exhibited moderate anticancer efficacy against HL-60(TB), K-562, MOLT-4, HCT-116, UACC-62, UO-31, T-47D, and MDA-MB-468 cell lines, with growth inhibition percentages ranging from 35% to 46.5%. Compounds **6a**, **6c**, and **7c** demonstrated moderate anticancer activity against a single cell line each: **6a** against UO-31 with a growth inhibition of 35%, **6c** against HOP-92 with a growth inhibition of 50%, and **7c** against UO-31 with a growth inhibition of 35%. Conversely, compounds **6b**, **6d**, **6e**, **7a**, and **7b** exhibited minimal anticancer activity across all cell lines.

Conversely, the NCI results showed that the anticancer effects of indole-N-phenyl-triazole-phenyl ethanone derivatives (**6f**–**j**) and their oxime derivatives (**7f**–**j**) were greater than those seen with similar ketones and oximes that contained the N-allyl triazole moiety. Among all evaluated compounds, **6h**, **7h**, **7i**, and **7j** exhibited the most effective anticancer activity against all tested cancer cell lines.

Compound **6h** exhibited complete cell death across ten cancer cell lines, with cell growth inhibition percentages varying from 101 to 141. It also exhibited significant anticancer efficacy, with cell growth inhibition percentages ranging from 67% to 99% across twenty-five cell lines, as demonstrated in Table 1. Compound **7h** resulted in complete cell death in thirty-six cell lines, exhibiting a cell growth inhibition percentage ranging from 105.5 to 200. Compound **7h** demonstrated significant anticancer efficacy against sixteen cell lines, with cell growth inhibition percentages ranging from 72% to 98%, while it showed modest activity against the other evaluated cancer cell lines.

Compound **7i** induced complete cell death in forty-nine cell lines, exhibiting cell growth inhibition percentages from 106.14 to 200. It demonstrated significant anticancer efficacy against eight cell lines, with inhibition percentages ranging from 86 to 99. It also demonstrated moderate activity against the 786-0 and A498 cell lines, with inhibition percentages of 65 and 38, respectively, as detailed in Table 1. Compound **7j** exhibited total cell death across twenty-eight cell lines, with cell growth inhibition percentages ranging from 102 to 200. It demonstrated significant anticancer activity against twenty-four cell lines, with cell growth inhibition percentages between 69.48 and 99.26. It also demonstrated moderate anticancer activity against six cell lines, with cell growth inhibition percentages ranging from 52 to 66.

The data indicated that indole derivatives containing an oxime moiety and their corresponding ketones with an N-phenyl triazole backbone exhibited superior anticancer activity compared to those with an N-allyl triazole backbone, suggesting the N-phenyl moiety’s significant role in enhancing anticancer efficacy. Additionally, compounds **7a**–**j** with the NO-releasing oxime moiety showed more anticancer activity than their precursor ketones **6a**–**j** across all examined cancer cell lines. The increased activity may be attributed to these compounds’ ability to produce NO, which is cytotoxic, in contrast to their precursor ketones. This demonstrates the significance of the oxime moiety in the anticancer efficacy of the target drugs.

##### In Vitro Five-Dose Full NCI 60 Cell Panel Assay

Compounds **6h**, **7h**, **7i**, and **7j**, the most efficacious derivatives, were chosen for a five-dose experiment against a panel of 60 human tumor cell lines across nine tumor subpanels. The evaluated derivatives were incubated with five different dosages (Supplementary Materials). The data was used to develop log concentration versus percentage growth inhibition curves, and three parameters (GI_50_, TGI, and LC_50_) were calculated for each cell line. The growth inhibitory activity (GI_50_) value signifies the concentration of a compound that causes a 50% decrease in net cell growth; the total growth inhibition (TGI) value indicates the concentration that results in complete growth inhibition, whereas the LC_50_ value reflects the concentration that leads to a 50% reduction of initial cells following a 48 h incubation period. The selectivity of a compound is quantified by the extent to which the whole panel MID (the mean sensitivity of all cell lines to the test agent) is less than or equal to the subpanel MID for each individual cell line. Ratios ranging from three to six indicate moderate selectivity; ratios exceeding six indicate high selectivity for the specific cell line, but chemicals that do not meet either requirement are categorized as nonselective [46]. The outcomes are presented in Table 2, Table 3, Table 4 and Table 5.

The data revealed that all tested compounds demonstrated broad anticancer efficacy across all nine tumor subpanels examined, with selectivity ratios ranging from 0.52 to 2.29 at the GI_50_ level. Compounds **7h** and **7j** (Table 3 and Table 5) showed remarkable anticancer activity against most of the tested cell lines across nine different subpanels, with GI_50_ values for most of the cells ranging from 1.85 to 5.76 µM and 2.45 to 5.23 µM, respectively. When tested against nine different types of cancer cells, compound **7i** (Table 4) showed strong anticancer activity, with GI_50_ values ranging from 2.10 to 3.23 µM. Also, Compound **6h** (Table 2) showed enhanced anticancer activity against most of the tested cell lines across nine separate subpanels, with GI_50_ values for most of the cells ranging from 8.42 to 33.48 µM.

##### Tubulin Polymerization Inhibitory Assay

The impact of new synthetic compounds **6h**, **7h**, **7i**, and **7j** on tubulin polymerization, with CA-4 as a reference compound [34,35], is presented in Table 6. The results of this in vitro experiment are consistent with the one-dose and five-dose NCI assays.

Compound **7i** (R = Ph, R_1_ = Br; oxime-based derivative) was the most potent tubulin inhibitor, with an IC_50_ value of 3.03 ± 0.11µM, compared to the reference CA-4 (IC_50_ value of 8.33 ± 0.29 µM). Compound **7i** was found to be approximately three times as effective as CA-4 as a tubulin inhibitor. With an IC_50_ value of 6.26 ± 0.15 µM, compound **7j** (R = Ph, R_1_ = Cl; oxime-based derivative) was the second most effective tubulin inhibitor. It was half as potent as compound **7i** but still better than the standard compound CA-4. The data suggest that the bromine atom at the *para*-position of ring B is more favorable to activity than the chlorine atom.

Compounds **6h** (R = Ph, R_1_ = CH_3_; ketone-based derivative) and **7h** (R = Ph, R_1_ = CH_3_; oxime-based derivative) had the lowest potency as tubulin inhibitors, at IC_50_ values of 9.50 ± 0.30 µM and 18.37 ± 0.70 µM, respectively. In comparison, CA-4 had an IC_50_ of 8.33 ± 0.29 µM, demonstrating that compounds **6h** and **7h** are less potent tubulin inhibitors than the reference drug CA-4.

The activity of compound **6h** was three times lower than compound **7i** and 1.5 times lower than compound **7j**. This shows how important the type of substitution at the *para*-position of the phenyl ring B is, with activity going up in the order of Br > Cl > CH_3_.

Finally, these results support the in vitro NCI results, showing that the oxime-based derivatives with a phenyl group at the 1,2,4-triazole ring are much more effective than other derivatives. This underscores the significance of the oxime group in the efficacy of these compounds.

#### 2.2.2. Cell Cycle Analysis and Detection of Apoptosis

##### Cell Cycle Analysis

**Figure 4:**
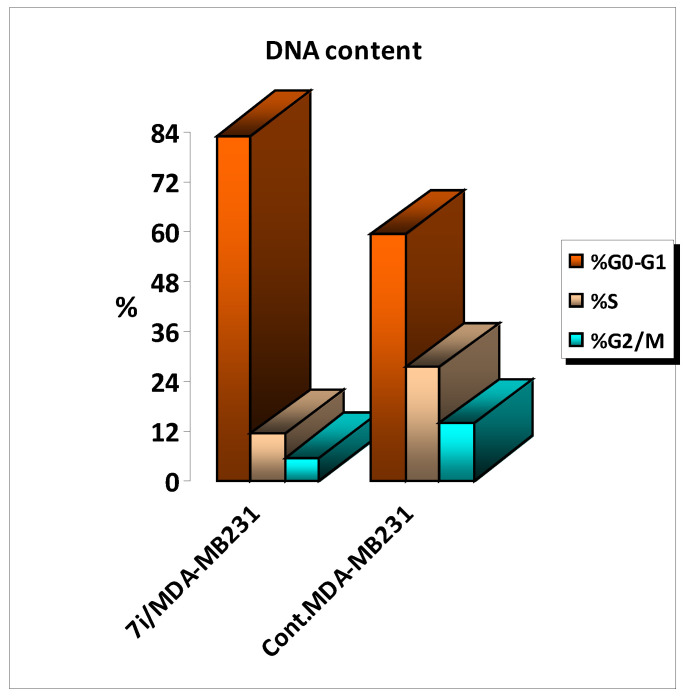
Cell cycle analysis of **7i** in MDA-MB 231 cell line.

The impact of compound **7i** on cell cycle progression was investigated in MDA-MB 231 breast cancer cells. The breast cancer (MDA-MB 231) cell line was treated for 24 h with an IC_50_ concentration of **7i**. The cell line was handled with PI/Annexin V, and flow cytometry was utilizing a BD FACS Caliber [47]. The results (Figure 4) indicated that MDA-MB 231 cells subjected to compound **7i** exhibited a substantial accumulation of 83% in the G0/G1 phase following 24 h of incubation. This indicates a cell cycle arrest at the G1 phase transition.

##### Assay for Induction of Apoptosis

**Figure 5:**
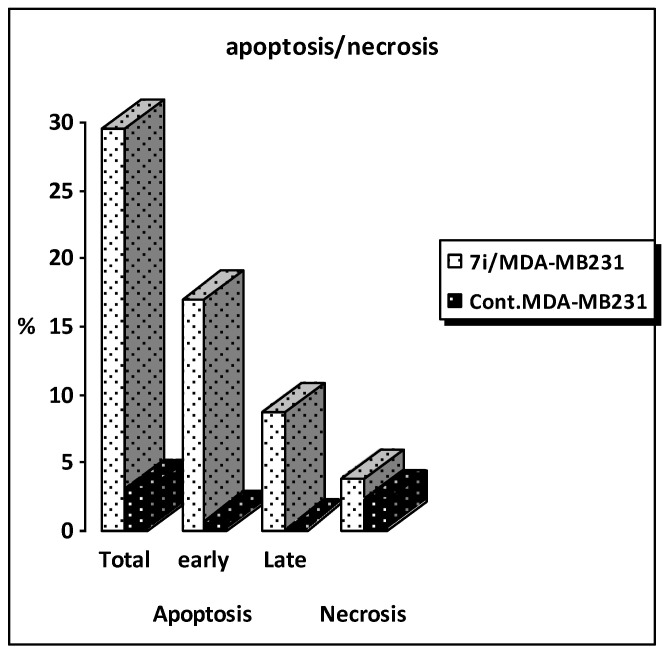
Results for apoptosis induction assay of **7i**.

**Figure 6:**
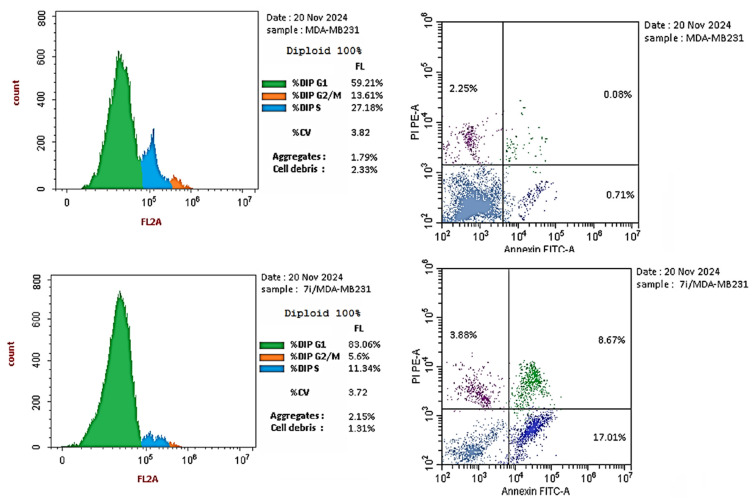
Results for cell cycle analysis and apoptosis induction of **7i**.

The MDA-MB 231 cell was labeled with Annexin V/PI, cultured for 24 h, and examined to assess the capacity of **7i** to induce apoptosis. Examining early and late apoptosis demonstrated that compound **7i** elicited substantial apoptosis, accompanied by a necrosis rate 3.88 (Figure 5 and Figure 6).

### 2.3. Molecular Modelling

Docking simulations of the most potent compounds, **6h**, **7h**, **7i**, and **7j**, were performed at the α/β-tubulin to explore their potential binding modes and rationalize the biological results. The X-ray crystal structure of Combretastatin A4 (cis-CA-4) bound to its tubulin complex was used in this investigation [PBD: 5LYJ] [48,49]. For ligand, protein, and docking simulations utilizing MOE software 2019, the Amber10:EHT forcefield and reaction field solvation model were applied [50,51].

**Figure 7:**
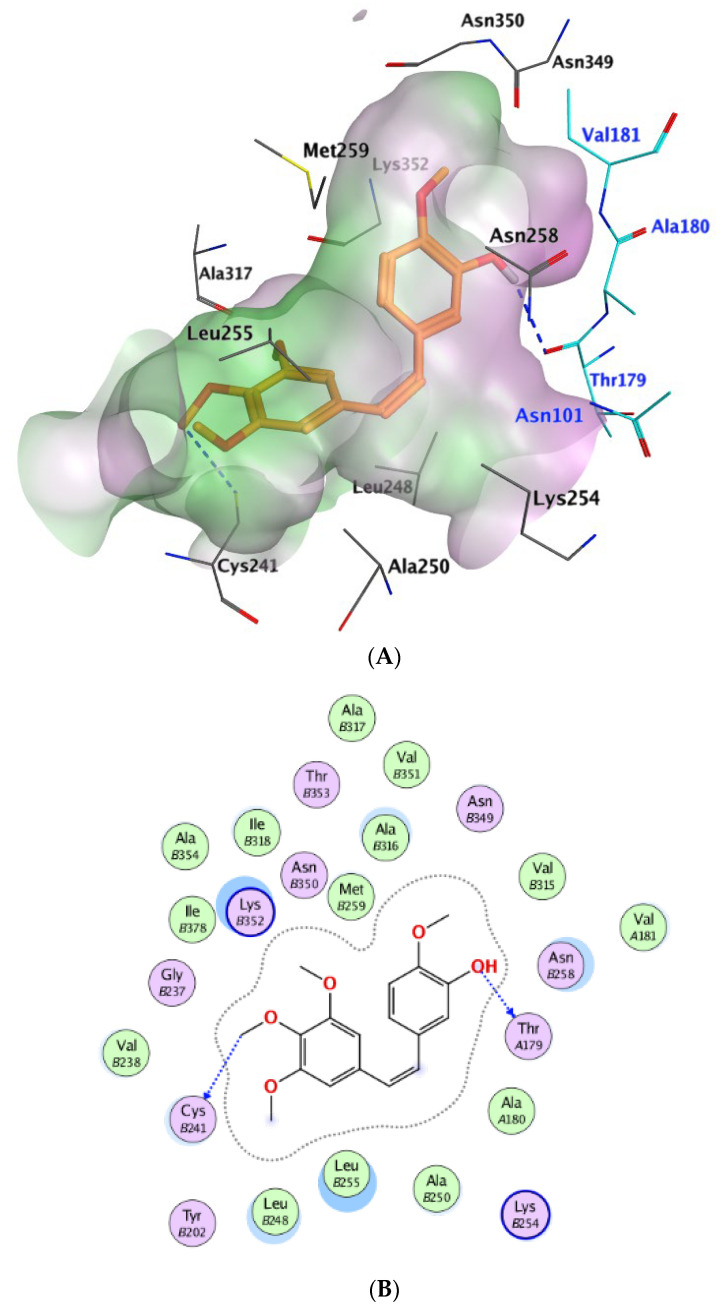
Ligplots at α/β-tubulin colchicine binding site: (**A**) 3D-docked model of CA-4 (dark yellow) showing the protein lipophilicity surface (purple: hydrophilic, white: neutral, and green: lipophilic), (cyan for α chain residues and brown for β chain residues), and (**B**) 2D-docked model of CA-4.

**Figure 8:**
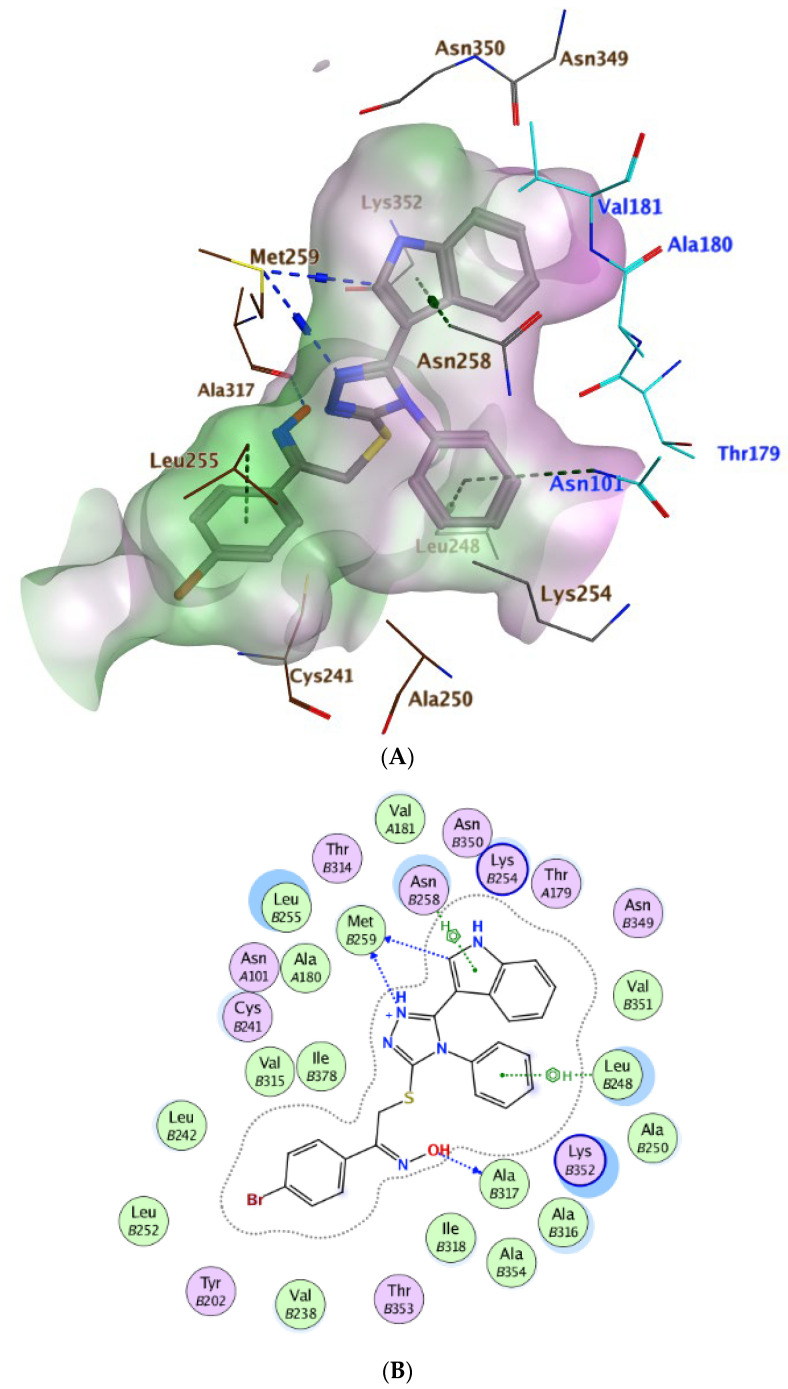
Ligplots at α/β-tubulin colchicine binding site: (**A**) a 3D-docked model of compound **7i** (grey) showing the protein lipophilicity surface (purple: hydrophilic, white: neutral, and green: lipophilic), (cyan for α chain residues and brown for β chain residues), and (**B**) a 2D-docked model of **7i**.

CA-4 occupies the same space as the microtubule-destabilizing agent, colchicine, and binds to the colchicine binding site (Figure 7). The protocol was initially validated by redocking the co-crystallized ligand, where the *RMSD* value obtained for the docked ligand was 0.77 Å (*S1*). Docked compounds displayed docking scores ranging from −7.39 to −8.31 kcal/mol compared with CA-4 (−8.68 kcal/mol). The simulation results of the compounds were compared with CA-4, and the data are shown in (Table 7). The visualization of the ligand complexes revealed that the oxime-based derivatives **7h**, **7i**, and **7j** exhibit better binding affinity than the ketone-based derivative **6h**. The indole ring (ring A) of the oxime-based derivatives **7h**, **7i**, and **7j** is lying in the pocket that allows a stacking interaction between βAsn258 and βLys352 along with forming pi-H contacts with βAsn258 or βLys352 residues, (Figure 8). The ligand indole NH or H-2 additionally forms an H-bond interaction with βMet259. Furthermore, the indole scaffold is stabilized by forming hydrophobic interactions with residues αThr179, αAla180, αVal181, βAsn350, and βAsn349. In addition, the ligand triazole linker donates an H-bond to βMet259 in the **7i** and **7j** complexes and establishes a pi-H contact with βLys352 in the **7j** complex. Moreover, in the **7h** complex, the N-substituted phenyl moiety establishes pi-H interactions with βLeu248 and αAsn101, and in the **7i** complex, with βLeu248.

**Figure 9:**
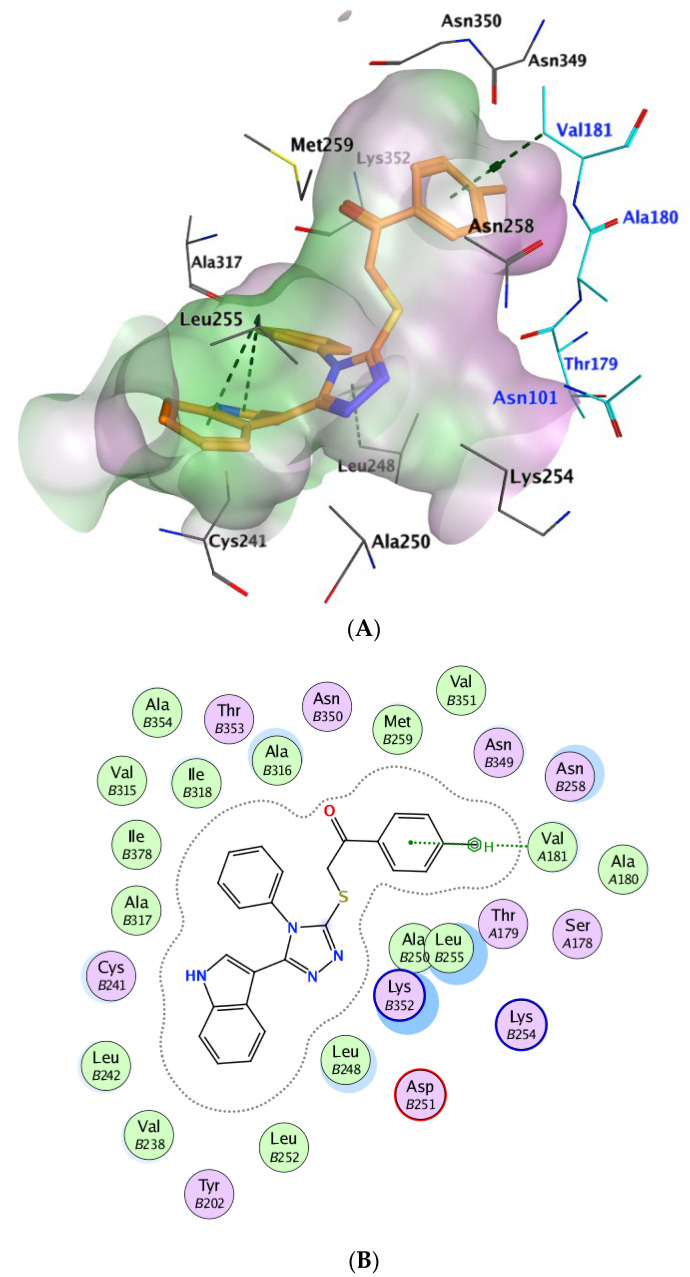
Ligplots at α/β-tubulin colchicine binding site: (**A**) 3D-docked model of compound **6h** (dark yellow) showing the protein lipophilicity surface (purple: hydrophilic, white: neutral, and green: lipophilic), (cyan for α chain residues and brown for β chain residues), and (**B**) 2D-docked model of **6h**.

Interestingly, the bulky bromine group in the **7i** complex enables the oxime group to touch the protein interaction surface and donate an H-bond to βAla317 with 2.05 Å. The complexes of the methyl-substituted derivative, **7h**, or the chlorine-substituted one, **7j**, lack H-bond interaction. In addition, the phenyl (ring B) is stacked between βCys241 and βLeu255 residues within the hydrophobic pocket (Figure 9), as well as forming pi-H contact with βLeu255 in the **7h** and **7j** complexes. The presence of a highly lipophilic and hydrophobic bromophenyl group in **7i** may also account for its increased activity when compared to tolyl and chlorophenyl moieties in **7h** and **7j**, respectively.

On the other hand, in the case of the ketone-based derivative, **6h** (Figure 3), the ligand is being upturned so the indole (ring A) is buried in the hydrophobic pocket and stacked between βCys241 and βLeu255. Also, the ligand N-phenyl triazole loses interactions with βMet259, βLys352, or βLeu248 at the junction of the binding site compared to the oxime-based derivatives. However, the ligand 4-methylphenyl moiety (ring B) forms pi-H contact with αVal181 and hydrophobic interactions with residues αThr179, βAsn350, and βAsn349, αAla180, and αVal181. Furthermore, the alignment of the **7i** complex onto the *cis*-CA-4 one revealed that the 3-HO-4-MeO-substituted ring of *cis*-CA-4 (ring B) superimposes on the indole moiety (ring A) of **7i**. Also, the 4-bromo-phenyl moiety of **7i** is buried deeper inside the pocket than is the 3,4,5-MeO-substituted ring of *cis*-CA-4 (ring B).

## 3. Materials and Methods

### 3.1. Chemistry

**General details:** See Appendix A.

Compounds **1** [39], **2** [40], **3** [41], **4** [42], and **5a**–**b** [42] were synthesized according to previously reported procedures.

#### 3.1.1. Synthesis of 2-((5-(1*H*-indol-3-yl)-4-allyl/phenyl-4*H*-1,2,4-triazol-3-yl)thio)-1-phenylethanone Derivatives (**6a**–**j**)

Compound **5** in an equimolar ratio with phenacyl bromide derivatives (1.00 mol), and TEA (1.00 mol) in 25 mL of acetonitrile were stirred at room temperature for 2 to 4 h. The reaction mixture evaporated to dryness. The residue was crystallized from aqueous ethanol, yielding compounds **6a**–**j** in good yields.

##### 2-((4-Allyl-5-(1*H*-indol-3-yl)-4*H*-1,2,4-triazol-3-yl)thio)-1-phenylethanone (**6a**)

White crystals in (0.109 g, 62% yield); m.p 189–193 °C ^1^H NMR (400 MHz, DMSO-d_6_) δ (ppm): 11.71 (s, 1H, Indole-N*H*), 8.09–8.02 (m, 3H, Ar-*H*), 7.75 (d, *J* = 8.00 Hz, 1H, Ar-*H*), 7.73–7.67 (m, 1H, Ar-*H*), 7.58 (t, *J* = 8.00 Hz, 2H, Ar-*H*), 7.50 (d, *J* = 8.00 Hz, 1H, Ar-*H*), 7.22 (t, *J* = 8.00 Hz, 1H, Ar-*H*), 7.14 (t, *J* = 8.00 Hz, 1H, Ar-*H*), 6.10–6.01 (m, 1H, N-CH_2_-C*H*=CH_2_), 5.24 (d, *J*_cis_ = 12.00 Hz, 1H, N-CH_2_-CH=C*H*_2_), 4.95 (s, 2H, S-C*H*_2_), 4.88 (d, *J_trans_* = 16.00 Hz,1H, N-CH_2_CH=C*H*_2_), 4.82 (d, *J* = 4.6 Hz, 2H, N-C*H*_2_-CH=CH_2_); ^13^C NMR (101 MHz, DMSO-d_6_) δ (ppm): 194.48, 151.66, 148.38, 136.39, 135.76, 134.23, 133.01, 129.32, 128.92, 126.09, 125.58, 122.89, 121.23, 120.76, 117.21, 112.32, 99.90, 47.27, 42.3; anal. calcd. for C_21_H_18_ClN_5_OS (374.46): C, 59.50; H, 4.28; N, 16.52; S, 7.56, found: C, 59.31; H, 4.39; N, 16.80; S, 7.64.

##### 2-((4-Allyl-5-(1*H*-indol-3-yl)-4*H*-1,2,4-triazol-3-yl)thio)-1-(4-methoxyphenyl) Ethenone (**6b**)

Yellow crystals in (0.115 g, 49% yield); m.p 191–194 °C ^1^H NMR (400 MHz, DMSO-d_6_) δ (ppm): 11.74 (s, 1H, Indole-N*H*), 8.09 (d, *J* = 8.00 Hz, 1H, Ar-*H*), 8.03 (d, *J* = 8.00 Hz, 2H, Ar-*H*), 7.76 (d, *J* = 2.8 Hz, 1H, Ar-*H*), 7.52 (d, *J* = 8.00 Hz, 1H, Ar-*H*,), 7.22 (t, *J* = 8.00 Hz, 1H, Ar-*H*), 7.14 (t, *J* = 8.00 Hz, 1H, Ar-*H*), 7.07 (d, *J* = 8.00 Hz, 2H, Ar-*H*), 6.10–6.01 (m, 1H, N-CH_2_-C*H*=CH_2_), 5.24 (d, *J*_cis_ = 12.00 Hz, 1H, N-CH_2_-CH=C*H*_2_), 4.92–4.85 (m, 3H, N-CH_2_CH=C*H*_2_+2H, S-C*H*_2_), 4.82 (d, *J* = 4.60 Hz, 2H, N-C*H*_2_-CH=CH_2_), 3.85 (s, 3H, O-CH_3_); ^13^C NMR (101 MHz, DMSO-d_6_) δ (ppm): 192.30, 164.05, 151.98, 149.20, 136.42, 133.04, 131.32, 128.58, 126.13, 125.52, 122.90, 121.26, 120.77, 117.17, 114.51, 112.34, 101.98, 56.05, 46.72, 41.3; anal. calcd. for C_22_H_20_N_4_O_2_S (404.48): C, 65.33; H, 4.98; N, 13.85; S, 7.93, found: C, 65.49; H, 5.12; N, 14.09; S, 8.01.

##### 2-((4-allyl-5-(1*H*-indol-3-yl)-4*H*-1,2,4-triazol-3-yl)thio)-1-(*p*-tolyl)ethanone (**6c**)

Yellow crystals in (0.200 g, 60% yield); m.p 219–224 °C ^1^H NMR (500 MHz, DMSO-d_6_) δ (ppm): 11.72 (s, 1H, Indole-N*H*), 8.06 (d, *J* = 8.00 Hz, 1H, Ar-*H*), 7.95 (d, *J* = 8.00 Hz, 2H, Ar-*H*), 7.74 (d, *J* = 2.8 Hz, 1H, Ar-*H*), 7.48 (d, *J* = 8.00 Hz, 1H, Ar-*H*), 7.38 (d, *J* = 8.00 Hz, 2H, Ar-*H*) 7.21 (t, *J* = 8.00 Hz, 1H, Ar-*H*), 7.14 (t, *J* = 8.00 Hz, 1H, Ar-*H*), 6.09–6.01 (m, 1H, N-CH_2_-C*H*=CH_2_), 5.24 (d, *J*_cis_ = 12.00 Hz, 1H, N-CH_2_-CH=C*H*_2_), 4.90 (s, 2H, S-C*H*_2_), 3.83 (d, *J_trans_* = 16.00 Hz,1H, N-CH_2_CH=C*H*_2_), 4.82 (d, *J* = 4.60 Hz, 2H, N-C*H*_2_-CH=CH_2_), 2.39 (s, 3H, -C*H*_3_); ^13^C NMR (126 MHz, DMSO-d_6_) δ (ppm): 193.48, 151.95, 149.05, 144.75, 136.40, 133.27, 133.09, 129.84, 129.05, 126.12, 125.51, 122.86, 121.28, 120.73, 117.16, 112.31, 101.97, 46.72, 41.83, 20.57.; anal. calcd. for C_22_H_20_N_4_OS (388.49): C, 68.02; H, 5.19; N, 14.42; S, 8.25, found: C, 68.31; H, 5.42; N, 14.67; S, 8.34.

##### 2-((4-Allyl-5-(1*H*-indol-3-yl)-4*H*-1,2,4-triazol-3-yl)thio)-1-(4-bromophenyl)ethenone (**6d**)

Yellowish white crystals in (0.144 g, 41% yield); m.p 227–231 °C ^1^H NMR (400 MHz, DMSO-d_6_) δ (ppm): 11.72 (s, 1H, Indole-N*H*), 8.07 (d, *J* = 8.00 Hz, 1H, Ar-*H*), 7.99–7.96 (d, *J* = 8.00 Hz, 2H, Ar-*H*), 7.78 (d, *J* = 8.00 Hz, 2H, Ar-*H*), 7.75 (d, *J* = 2.8 Hz, 1H, Ar-*H*), 7.51 (d, *J* = 8.00 Hz, 1H, Ar-*H*), 7.22 (t, *J* = 8.00 Hz, 1H, Ar-*H*), 7.14 (t, *J* = 8.00 Hz, 1H, Ar-*H*), 6.10–6.01 (m, 1H,N-CH_2_-C*H*=CH_2_), 5.24 (d, *J*_cis_ = 12.00 Hz, 1H, N-CH_2_-CH=C*H*_2_), 4.92 (s, 2H, S-C*H*_2_), 4.87 (d, *J_trans_* = 16.00 Hz,1H, N-CH_2_CH=C*H*_2_), 4.82 (d, *J* = 4.6 Hz, 2H, N-C*H*_2_-CH=CH_2_); ^13^C NMR (101 MHz, DMSO-d_6_) δ (ppm): 193.30, 152.02, 148.91, 136.41, 134.81, 133.02, 132.35, 130.92, 128.36, 126.13, 125.52, 122.87, 121.27, 120.75, 117.21, 112.32, 101.96, 48.02, 41.30; anal. calcd. for C_21_H_17_BrN_4_OS (453.35): C, 55.64; H, 3.78; N, 12.36; S, 7.07, found: C, 55.89; H, 3.91; N, 12.50; S, 7.23.

##### 2-((4-Allyl-5-(1*H*-indol-3-yl)-4*H*-1,2,4-triazol-3-yl)thio)-1-(4-chlorophenyl)ethenone (**6e**)

Yellow crystals in (0.120 g, 63% yield); m.p 155–157 °C; ^1^H NMR (400 MHz, DMSO-d_6_) δ (ppm): 11.75 (s, 1H, indole-N*H*), 8.08–8.03 (m, 3H, Ar-*H*), 7.77 (t, J = 8.00 Hz, 1H, Ar-*H*), 7.65 (d, 2H, *J=* 8.00 Hz, Ar-*H*), 7.51 (d, *J* = 8.00 Hz, 1H, Indole-*H*), 7.22 (t, *J* = 8.00 Hz, 1H, Ar-*H*), 7.14 (t, *J* = 8.00 Hz, 1H, Indole-C*H*), 6.10–6.01 (m, 1H, N-CH_2_C*H*=CH_2_), 5.25 (d, *J*_cis_ = 12.00 Hz, 1H, N-CH_2_-CH=C*H*_2_), 4.94 (s, 2H, S-C*H*_2_), 4.90 (d, *J_trans_* = 16.00 Hz,1H, N-CH_2_CH=C*H*_2_), 4.82 (d, *J* = 4.6 Hz, 2H, N-C*H*2-CH=CH_2_); ^13^C NMR (101 MHz, DMSO-d_6_) δ (ppm): 193.00, 151.89, 149.25, 139.17, 136.41, 134.45, 132.85, 130.85, 129.43, 126.00, 125.91, 122.96, 121.13, 120.86, 117.34, 112.40, 101.41, 46.87, 41.32; anal. calcd. for C_21_H_17_ClN_4_OS (408.90): C, 61.68; H, 4.19; N, 13.70; S, 7.84 found: C, 61.90; H, 4.37; N, 13.94; S, 7.95.

##### 2-((5-(1*H*-indol-3-yl)-4-phenyl-4*H*-1,2,4-triazol-3-yl)thio)-1-phenylethanone (**6f**)

Yellow crystals (0.100 g, 49% yield); m.p 230–233^1^H NMR (500 MHz, DMSO-d_6_) δ (ppm): 11.39 (s, 1H, Indole-N*H*), 8.16 (d, *J* = 8.00 Hz, 1H, Pyrrole-C*H*), 8.05–8.03 (m, 2H, Ar-*H*), 7.71–7.68 (m, 1H, Ar-*H*), 7.65 (d, *J* = 8.00 Hz, 2H, Ar-*H*), 7.64 (d, *J* = 8.00,1H, Ar-*H*) 7.57 (t, *J* = 8.00, 2H, Ar-*H*), 7.52–7.50 (m, 2H, Ar-*H*), 7.41 (d, *J* = 8.00 Hz, 1H, Ar-*H*), 7.17 (t, *J* = 8.00 Hz, 1H, Ar-*H*), 7.11 (t, *J* = 8.00 Hz, 1H, Ar-*H*), 6.50 (d, *J* = 5 Hz, 1H, Ar-*H*), 4.91 (s, 2H, S-C*H*_2_); ^13^C NMR (126 MHz, DMSO-d_6_) δ (ppm): 192.45, 152.00, 150.27, 136.03, 135.81, 134.69, 134.22, 130.88, 130.76, 129.32, 128.91, 128.50, 125.75, 124.82, 122.94, 121.63, 120.83, 112.28, 102.32, 40.72; anal. calcd. for C_24_H_18_N_4_OS (410.49): C, 70.22; H, 4.42; N, 13.65; S, 7.81; found: C, 70.43; H, 4.56; N, 13.91; S, 7.89.

##### 2-((5-(1*H*-Indol-3-yl)-4-phenyl-4*H*-1,2,4-triazol-3-yl)thio)-1-(4-methoxyphenyl) Ethenone (**6g**)

Yellow crystals in (0.141g, 62% yield); m.p 236–239 ^1^H NMR (500 MHz, DMSO-d_6_) δ (ppm): 11.38 (s, 1H, Indole-N*H*), 8.16 (d, *J* = 8.00 Hz, 1H, Ar-*H*), 8.01 (d, *J* = 8.00 Hz, 2H, Ar-*H*), 7.66–7.63 (m, 3H, Ar-*H*), 7.51–7.49 (m, 2H, Ar-*H*), 7.40 (d, *J* = 8.00 Hz, 1H, Ar-*H*), 7.18 (t, *J* = 8.00 Hz, 1H, Ar-*H*), 7.13 (t, *J* = 8.00 Hz, 1H, Ar-*H*), 7.08 (d, *J* = 8.00 Hz,2H, Ar-*H*), 6.49 (d, *J* = 3.5 Hz, 1H, Ar-*H*), 4.85 (s, 2H, S-C*H*_2_), 3.87 (s, 3H, OC*H*_3_); ^13^C NMR (126 MHz, DMSO-d_6_) δ (ppm): 187.90, 164.06, 151.97, 147.24, 136.03, 134.71, 131.32, 130.86, 130.74, 128.64, 128.51, 125.75, 124.81, 122.94, 121.63, 120.83, 114.54, 112.27, 102.34, 56.12, 40.57; anal. calcd. for C_25_H_20_N_4_O_2_S (440.52): C, 68.16; H, 4.58; N, 12.72; S, 7.28, found: C, 68.40; H, 4.67; N, 12.98; S, 7.40.

##### 2-((5-(1*H*-Indol-3-yl)-4-phenyl-4*H*-1,2,4-triazol-3-yl)thio)-1-(*p*-tolyl)ethenone (**6h**)

Yellow crystals (0.100 g, 53% yield); m.p 235–239 ^1^H NMR (500 MHz, DMSO-d_6_) δ (ppm): 11.37 (s, 1H, Indole-N*H*), 8.15 (d, *J* = 8.00 Hz, 1H, Ar-*H*), 7.93 (d, *J* = 8.00 Hz, 2H, Ar-*H*), 7.67–7.62 (m, 3H, Ar-*H*), 7.53–7.50 (m, 2H, Ar-*H*), 7.41–7.37 (m, 3H, Ar-*H*), 7.17 (t, *J* = 8.00 Hz, 1H, Ar-*H*), 7.12 (t, *J* = 8.00 Hz, 1H, Ar-*H*), 6.49 (d, *J* = 3.5 Hz, 1H, Ar-*H*), 4.87 (s, 2H, S-C*H*_2_), 2.41 (s, 3H, C*H*_3_); ^13^C NMR (126 MHz, DMSO-d_6_) δ (ppm): 193.24, 151.97, 149.52, 144.76, 136.02, 134.69, 133.30, 130.87, 130.76, 129.68, 129.86, 128.50, 125.74, 124.82, 122.94, 121.62, 120.83, 112.27, 102.32, 40.69, 21.70; anal. calcd. for C_25_H_20_N_4_OS (424.52): C, 70.73; H, 4.75; N, 13.20; S, 7.55, found: C, 70.98; H, 4.89; N, 13.47; S, 7.62.

##### 2-((5-(1*H*-Indol-3-yl)-4-phenyl-4*H*-1,2,4-triazol-3-yl)thio)-1-(4-bromophenyl) Ethenone (**6i**)

Yellow crystals in (0.159 g, 63% yield); m.p 229–232 ^1^H NMR (500 MHz, DMSO-d_6_) δ (ppm): 11.37 (s, 1H, Indole-N*H*), 8.15 (d, *J* = 8.00 Hz, 1H, Ar-*H*), 7.97(d, *J* = 8.00 Hz, 2H, Ar-*H*), 7.79 (d, *J* = 8.00 Hz, 2H, Ar-*H*), 7.66–7.63 (m, 3H, Ar-*H*), 7.53–7.50 (m, 2H, Ar-*H*), 7.40 (d, *J* = 8.00 Hz, 1H, Ar-*H*), 7.18 (t, *J* = 8.00, 1H, Ar-*H*), 7.13 (t, *J* = 8.00, 1H, Ar-*H*), 6.49 (d, *J* = 3.5 Hz, 1H, Ar-*H*), 4.87 (s, 2H, S-C*H*_2_); ^13^C NMR (126 MHz, DMSO-d_6_) δ (ppm): 193.11, 152.03, 149.34, 136.03, 134.85, 134.66, 132.38, 130.93, 130.89, 130.76, 128.48, 128.33, 125.73, 124.84, 122.94, 121.62, 120.83, 111.59, 101.63, 40.59; anal. calcd. for C_24_H_17_BrN_5_OS (489.39): C, 58.90; H, 3.50; Br, 16.33; N, 11.45; S, 6.55, found: C, 59.12; H, 3.66; N, 11.72; S, 6.64.

##### 2-((5-(1*H*-indol-3-yl)-4-phenyl-4*H*-1,2,4-triazol-3-yl)thio)-1-(4-chlorophenyl) Ethenone (**6j**)

Yellow crystals in (0.078 g, 34% yield); m.p 234–237 ^1^H NMR (500 MHz, DMSO-d_6_) δ (ppm): 11.37 (s, 1H, indole-N*H*), 8.15 (d, *J* = 8.00 Hz, 1H, Ar-*H*), 8.05 (d, *J* = 8.00 Hz, 2H, Ar-*H*), 7.66–7.64 (m, 5H, Ar-*H*), 7.52–7.50 (m, 2H, Ar-*H*), 7.40 (d, *J* = 8.00 Hz, 1H, Ar-*H*), 7.17 (t, *J* = 8.00 Hz, 1H, Ar-*H*), 7.12 (t, *J* = 8.00 Hz, 1H, Ar-*H*), 6.49 (d, *J* = 3.5 Hz, 1H, Ar-*H*), 4.87 (s, 2H, S-C*H*_2_); ^13^C NMR (126 MHz, DMSO-d_6_) δ (ppm): 192.35, 152.03, 149.35, 139.12, 136.02, 134.65, 134.52, 130.89, 130.85, 130.76, 129.43, 127.57, 125.72, 124.84, 122.95, 121.61, 120.84, 112.27, 101.92, 40.56; anal. calcd. for C_24_H_17_ClN_4_OS (444.94): C, 64.79; H, 3.85; Cl, 7.97; N, 12.59; S, 7.21, found: C, 65.02; H, 4.01; N, 12.80; S, 7.35.

#### 3.1.2. General Procedure for the Synthesis of (Z)-2-((5-(1*H*-indol-3-yl)-4-allyl/phenyl-4*H*-1,2,4-triazol-3-yl)thio)-1-phenylethanone Oxime Derivatives (**7a**–**j**)

A mixture of the appropriate ketone **6a**–**j** (1.00 mol), hydroxylamine hydrochloride (5.00 mol), and anhydrous sodium acetate (5.00 mol) in absolute ethanol (30 mL) was heated under reflux at a temperature of 50–70 °C for 2–8 h. The reaction mixture was filtered to separate hydroxylamine hydrochloride and anhydrous sodium acetate. The filtrate was evaporated, and the separated solid was washed with a diluted ammonia solution (10%), dried, and crystallized from aqueous ethanol, affording the target compounds **7a**–**j**.

##### (Z)-2-((4-Allyl-5-(1*H*-indol-3-yl)-4*H*-1,2,4-triazol-3-yl)thio)-1-phenylethanone Oxime (**7a**)

Yellow crystals in (0.085 g, 48% yield); m.p 203–209 °C; ^1^H NMR (400 MHz, DMSO-d_6_) δ (ppm): 11.81 (s, 1H, O*H*), 11.69 (s, 1H, indole-N*H*), 8.07 (d, *J* = 8.00 Hz, 1H, Ar-*H*), 7.71–7.69 (m, 3H, 2Ar-*H+*CO-C*H*=CH), 7.49 (d, *J* = 8.00 Hz, 1H, Ar-*H*), 7.42–7.34 (m, 3H, 2Ar-*H*+CO-CH=C*H*), 7.23 (t, *J* = 8.00 Hz, 1H, Ar-*H*), 7.16 (t, *J* = 8.00 Hz, 1H, Ar-*H*), 6.00–5.91 (m, 1H, N-CH_2_-C*H*=CH_2_), 5.13 (d, *J*_cis_ = 12.00 Hz, 1H, N-CH_2_-CH=C*H*_2_), 4.74 (*J_trans_* = 16.00 Hz,1 H, N-CH_2_CH=C*H*_2_), 4.69 (d, *J* = 4.6 Hz, 2H, N-C*H*_2_-CH=CH_2_), 4.43 (s, 2H, S-C*H*_2_); ^13^C NMR (101 MHz, DMSO-d_6_) δ (ppm): ^13^C NMR 152.26, 151.67, 148.50, 135.90, 134.41, 132.67, 129.10, 128.50, 125.89, 125.67, 125.14, 122.38, 120.89, 120.26, 116.53, 111.82, 101.58, 46.52, 25.94; anal. calcd. for C_21_H_19_N_5_OS (389.47): C, 64.76; H, 4.92; N, 17.98; S, 8.23, found: C, 64.52; H, 5.07; N, 18.15; S, 8.40.

##### 2-((4-allyl-5-(1*H*-indol-3-yl)-4*H*-1,2,4-triazol-3-yl)thio)-1-(4-methoxyphenyl) Ethanone Oxime (**7b**)

Yellow crystals in (0.120 g, 47% yield); m.p 191–194 °C ^1^H NMR (500 MHz, DMSO-d_6_) δ (ppm): 11.77 (s, 1H, -O*H*), 11.63 (s, 1H, Indole-N*H*), 8.08 (d, *J* = 8.00 Hz, 1H, Ar-*H*), 8.69 (s, 1H, Ar-*H*), 7.61 (d, *J* = 8.00 Hz, 2H, Ar-*H*), 7.50 (d, *J* = 8.00 Hz, 1H, Ar-*H*), 7.22 (t, *J* = 8.00 Hz, 1H, Ar-*H*), 7.15 (t, *J* = 8.00 Hz, 1H, Ar-*H*), 6.94 (d, *J* = 8.00 Hz, 2H, Ar-*H*), 6.00–5.92 (m, 1H, N-CH_2_-C*H*=CH_2_), 5.14 (d, *J*_cis_ = 12.00 Hz, 1H, N-CH_2_-CH=C*H*_2_), 4.71 (d, *J_trans_* = 16.00 Hz, N-CH_2_CH=C*H*_2_), 4.68 (d, *J* = 2.30 Hz, 2H, N-C*H*_2_-CH=CH_2_), 4.39 (s, 2H, S-*CH*_2_), 3.71 (s, 3H, O-CH_3_); ^13^C NMR (126 MHz, DMSO-d_6_) δ (ppm): 160.44, 152.33, 152.11, 149.06, 136.38, 133.18, 127.72, 127.20, 126.14, 125.58, 122.83, 121.34, 120.70, 116.96, 114.37, 112.31, 101.08, 55.61, 46.57, 26.77.; anal. calcd. for C_22_H_21_N_5_O_2_S (419.50): C, 62.99; H, 5.05; N, 16.69; S, 7.64, found: C, 63.18; H, 5.42; N, 14.67; S, 8.34.

##### (Z)-2-((4-allyl-5-(1*H*-indol-3-yl)-4*H*-1,2,4-triazol-3-yl)thio)-1-(*p*-tolyl)ethanone Oxime (**7c**)

Yellowish white crystals (0.750 g, 70% yield); m.p 128–204 °C ^1^H NMR (400 MHz, DMSO) δ 11.98 (s, 1H, O*H*), 11.72 (s, 1H, Indole-N*H*), 8.08 (d, *J* = 8 Hz, 1H, Pyrrole-C*H*), 7.69 (d, *J* = 2.8 Hz, 1H, Ar-*H*), 7.58 (d, *J* = 8 Hz, 2H, Ar-*H*), 7.52 (d, *J* = 8 Hz, 1H, Ar-*H*), 7.24–7.20 (t, *J* = 8 Hz, 1H, Indole-C*H*),7.18–7.16 (d, *J* = 8 Hz, 2H, Ar-*H)*, 7.17–7.12 (t, *J* = 8.0 Hz, 1H, Indole-C*H*), 6.00–5.91 (m, 1H, N-CH2-C*H*=CH2), 5.15 (d, *J*_cis_ = 12 Hz, 1H, N-CH2-CH=C*H*2), 4.73 (d, *J_trans_* = 16 Hz,1H, N-CH_2_CH=C*H*_2_), 4.68 (d, J = 4.6 Hz, 2H, N-C*H*2-CH=CH_2_), 4.41 (s, 2H, S-C*H*_2_), 2.24 (s, 3H,C*H*_3_); ^13^C NMR (101 MHz, DMSO) δ 152.63, 152.13, 149.00, 139.09, 136.40, 133.15, 132.04, 129.52, 126.24, 126.13, 125.57, 122.82, 121.30, 120.70, 116.95, 112.35, 101.96, 47.27, 26.64, 21.19; anal. calcd. for C_22_H_21_N_5_OS (403.15): C, 65.49; H, 5.25; N, 17.36; O, 3.97; S, 7.95, found: C, 65.32; H, 5.43; N, 17.52; S, 8.09.

##### (Z)-2-((4-allyl-5-(1*H*-indol-3-yl)-4*H*-1,2,4-triazol-3-yl)thio)-1-(4-bromophenyl) Ethanone Oxime (**7d**)

Yellowish white crystals in (0.131 g, 55% yield); m.p 217–222 ^1^H NMR (400 MHz, DMSO-d_6_) δ (ppm): 11.98 (s, 1H, O*H*), 11.72 (s, 1H, Indole-N*H*), 8.09 (d, *J* = 8.00 Hz, 1H, Ar-*H*), 7.72 (d, *J* = 2.8 Hz, 1H, Ar-*H*), 7.66 (d, *J* = 8.00 Hz, 2H, Ar-*H*), 7.59 (d, *J* = 8.00 Hz, 2H, Ar-*H*), 7.52–7.49 (m, 1H, Ar-*H*), 7.23 (t, *J* = 8.00 Hz, 1H, Ar-*H*), 7.16 (t, *J* = 8.00 Hz, 1H, Ar-*H*) 6.01–5.92 (m, 1H, N-CH_2_-C*H*=CH_2_), 5.16 (d, *J*_cis_ = 12.00 Hz, 1H, N-CH_2_-CH=C*H*_2_), 4.76 (d, *J_trans_* = 16.00 Hz,1H, N-CH_2_CH=C*H*_2_), 4.72 (d, *J* = 4.6 Hz, 2H, N-C*H*_2_-CH=CH_2_), 4.43 (s, 2H, S-C*H*_2_); ^13^C NMR (101 MHz, DMSO-d_6_) δ (ppm): 152.18, 152.03, 148.70, 136.40, 134.11, 133.13, 131.90, 128.42, 126.16, 125.64, 122.99, 122.88, 121.34, 120.78, 117.03, 112.31, 102.03, 46.63, 27.02; anal. calcd. for C_21_H_18_BrN_5_OS (468.37): C, 53.85; H, 3.87; N, 14.95; S, 6.85, found: C, 54.03; H, 4.03; N, 15.17; S, 6.98.

##### (Z)-2-((4-allyl-5-(1*H*-indol-3-yl)-4*H*-1,2,4-triazol-3-yl)thio)-1-(4-chlorophenyl) Ethanone Oxime (**7e**)

Yellow crystals in (0.048 g, 43% yield); m.p 163–167 °C; ^1^H NMR (400 MHz, DMSO-d_6_) δ (ppm): 11.98 (s, 1H, O*H*), 11.72 (s, 1H, N*H*), 8.09 (d, *J* = 8.00 Hz, 1H, pyrrole-C*H*), 7.74–7.72 (m, 3H, Ar-*H*), 7.51 (d, *J* = 8.00 Hz, 1H, Ar-*H*), 7.45 (d, *J* = 8.00 Hz, 2H, Ar-*H*), 7.23 (t, *J* = 8.00 Hz, 1H, Ar-*H*), 7.16 (t, *J* = 8.00 Hz, 1H, Ar-*H*), 6.02–5.92 (m, 1H, N-CH_2_-C*H*=CH_2_), 5.16 (d, *J*_cis_ = 8.00 Hz, 1H, N-CH_2_-CH=C*H*_2_), 4.74 (d, *J_trans_* = 16.00 Hz,1H, N-CH_2_CH=C*H*_2_), 4.72 (d, *J* = 4.6 Hz, 2H, N-C*H*_2_-CH=CH_2_), 4.43 (s, 2H, S-C*H*_2_); ^13^C NMR (101 MHz, DMSO-d_6_) δ (ppm): 152.18, 151.94, 148.71, 136.39, 134.29, 133.73, 133.13, 129.40, 128.15, 126.14, 125.64, 122.88, 121.34, 120.77, 117.01, 112.31, 102.02, 47.65, 27.46; anal. calcd. for C_21_H_18_ClN_5_OS (423.92): C, 59.50; H, 4.28; N, 16.52; S, 7.56, found: C, 59.72; H, 4.40; N, 16.78; S, 7.67.

##### (Z)-2-((5-(1*H*-Indol-3-yl)-4-phenyl-4*H*-1,2,4-triazol-3-yl)thio)-1-phenylethanone Oxime (**7f**)

Yellow crystals in (0.040 g, 42% yield); m.p 260–267 °C ^1^H NMR (500 MHz, DMSO-d_6_) δ (ppm): 11.79 (s,1H, O*H*), 11.38 (s, 1H, Indole-N*H*), 8.22 (d, *J* = 8.00 Hz, 1H, Pyrrole-C*H*), 7.68–7.66 (m, 2H, Ar-*H*), 7.63–7.58 (m, 3H, Ar-*H*), 7.45–7.43 (m, 2H, Ar-*H*), 7.42–7.41 (m, 2H, Ar-*H*), 7.40–7.38 (m, 2H, Ar-*H*), 7.20 (t, *J* = 8.00 Hz, 1H, Ar-*H*), 7.14 (t, *J* = 8.00 Hz, 1H, Ar-*H*), 6.44 (s, 1H, Ar-*H*), 4.39 (s, 2H, S-C*H*_2_); ^13^C NMR (126 MHz, DMSO-d_6_) δ (ppm): 152.45, 152.39, 149.26, 136.03, 134.85, 134.81, 130.78, 130.63, 129.59, 128.98, 128.59, 126.37, 125.78, 124.84, 122.96, 121.76, 120.85, 113.01, 102.43, 28.10; anal. calcd. for C_24_H_19_N_5_OS (425.51): C, 67.74; H, 4.50; N, 16.46; S, 7.54, found: C, 68.01; H, 4.67; N, 16.72; S, 7.61.

##### (Z)-2-((5-(1*H*-Indol-3-yl)-4-phenyl-4*H*-1,2,4-triazol-3-yl)thio)-1-(4-methoxyphenyl) Ethanone Oxime (**7g**)

Yellow crystals in (0.076 g, 65% yield); m.p 202–206 °C ^1^H NMR (500 MHz, DMSO-d_6_) δ (ppm): 11.56 (s,1H, O*H*), 11.42 (s, 1H, Indole-N*H*), 8.22 (d, *J* = 8.00 Hz, 1H, Pyrrole-C*H*), 7.63–7.58 (m, 5H, Ar-*H*), 7.45– 7.43 (m, 2H, Ar-*H*), 7.41 (d, *J* = 8.00 Hz 1H, Ar-*H*), 7.20 (t, *J* = 8.00 Hz, 1H, Ar-*H*), 7.14 (t, *J* = 8.00 Hz, 1H, Ar-*H*), 6.95 (d, *J* = 8.00 Hz,2H, Ar-*H*), 6.49 (d, *J* = 3.00Hz, 1H, Ar-*H*), 4.37 (s, 2H, S-C*H*_2_), 3.76 (s, 3H, OC*H*_3_); ^13^C NMR (126 MHz, DMSO-d_6_) δ (ppm): 160.50, 152.34, 152.04, 149.42, 136.02, 134.79, 130.78, 130.35, 128.58, 127.76, 126.77, 125.78, 124.84, 122.95, 121.74, 120.84, 114.41, 112.27, 101.40, 55.66, 26.61; anal. calcd. for C_25_H_21_N_5_O_2_S (455.53): C, 65.92; H, 4.65; N, 15.37; S, 7.04, found: C, 65.71; H, 4.82; N, 15.54; S, 7.18.

##### (Z)-2-((5-(1*H*-Indol-3-yl)-4-phenyl-4*H*-1,2,4-triazol-3-yl)thio)-1-(*p*-tolyl)ethanone Oxime (**7h**)

Yellow crystals in (0.035 g, 25% yield); m.p 239–241 °C ^1^H NMR (500 MHz, DMSO) δ 11.66 (s,1H, O*H*), 11.37 (s, 1H, Indole-N*H*), 8.22 (d, *J* = 8.00 Hz, 1H, Pyrrole-C*H*), 7.63–7.59 (m, 3H, Ar-*H*), 7.55 (d, *J* = 8.00 Hz, 2H, Ar-*H*), 7.45–7.43 (m, 2H, Ar-*H*), 7.41 (d, *J* = 8.00 1H, Ar-*H*), 7.21–7.19 (m, 2H, Ar-*H*), 7.17–7.13 (m. 2H, Ar-*H*) 6.44 (d, *J* = 2.00 Hz, 1H, Ar-*H*), 4.37 (s, 2H, S-C*H*_2_), 2.31 (s, 3H, C*H*_3_); ^13^C NMR (126 MHz, DMSO-d_6_) δ (ppm): 152.35, 152.31, 149.33, 138.77, 136.02, 134.80, 132.04, 130.77, 130.63, 129.56, 128.59, 126.28, 125.78, 124.84, 122.95, 121.74, 120.45, 112.26, 102.43, 26.22, 23.18; anal. calcd. for C_25_H_21_N_5_OS (439.53): C, 68.32; H, 4.82; N, 15.93; S, 7.30, found: C, 68.50; H, 4.97; N, 16.17; S, 7.26.

##### (Z)-2-((5-(1*H*-Indol-3-yl)-4-phenyl-4*H*-1,2,4-triazol-3-yl)thio)-1-(4-bromophenyl) Ethanone Oxime (**7i**)

Yellow crystals in (0.070 g, 66% yield); m.p 242–244 °C ^1^H NMR (500 MHz, DMSO-d_6_) δ (ppm): 11.90 (s,1H, O*H*), 11.38 (s, 1H, Indole-N*H*), 8.21 (d, *J* = 8.00 Hz, 1H, Pyrrole-C*H*), 7.65–7.59 (m, 7H, Ar-*H*), 7.45–7.41 (m, 2H, Ar-*H*), 7.40 (d, J = 8.00 Hz, 1H, Ar-*H*), 7.18 (t, *J* = 8.00 Hz, 1H, Ar-*H*), 7.14 (t, *J* = 8.00 Hz, 1H, Ar-*H*), 6.44 (d, *J* = 3.5 Hz, 1H, Ar-*H*), 4.35 (s, 2H, S-C*H*_2_); ^13^C NMR (126 MHz, DMSO) δ 152.43, 151.68, 148.88, 136.01, 134.80, 134.08, 132.43, 131.92, 130.78, 130.63, 128.58, 128.45, 125.77, 124.87, 122.97, 121.74, 120.86, 113.28, 102.66, 25.49; anal. calcd. for C_24_H_18_BrN_5_OS (504.40): C, 57.15; H, 3.60; Br, 15.84; N, 13.88; S, 6.36, found: C, 57.38; H, 3.74; N, 14.12; S, 6.45.

##### (Z)-2-((5-(1*H*-Indol-3-yl)-4-phenyl-4*H*-1,2,4-triazol-3-yl)thio)-1-(4-chlorophenyl) Ethanone Oxime (**7j**)

Yellow crystals in (0.70 g, 66% yield); m.p 248–250 °C ^1^H NMR (400 MHz, DMSO-d_6_) δ (ppm): 11.90 (s, 1H, O*H*), 11.40 (s, 1H, Indole-N*H*), 8.24 (d, *J* = 8.00 Hz, 1H, Pyrrole-C*H*), 7.71 (d, *J* = 8.00 Hz, 2H, Ar-*H*), 7.63–7.61 (m, 3H, Ar-*H*), 7.48–7.42 (m, 5H, Ar-*H*), 7.26–7.14 (m, 2H, Ar-*H*), 6.47 (s, 1H, Ar-*H*), 4.38 (s, 2H, S-C*H*_2_); ^13^C NMR (101 MHz, DMSO-d_6_) δ (ppm): 152.47, 151.67, 149.04, 136.06, 134.78, 134.31, 133.70, 130.93, 130.78, 130.62, 129.00, 128.55, 128.18, 125.80, 124.93, 123.00, 121.75, 120.91, 112.29, 102.41, 28.1; anal. calcd. for C_24_H_18_ClN_5_OS (459.95): C, 62.67; H, 3.94; N, 15.23; S, 6.97, found: C, 62.51; H, 4.12; N, 15.45; S, 7.08.

### 3.2. Biology

#### 3.2.1. NCI’s In Vitro Study of Antiproliferative Activity

The NCI anticancer screening process has been thoroughly detailed [43,47]. The Drug Evaluation Branch of the National Cancer Institute in Bethesda, MA, USA, established the procedure and conducted the anticancer assay on 60 human tumor cell lines from nine neoplastic cancers [43,47].

#### 3.2.2. Tubulin Polymerization Assay

The substances’ effects on tubulin polymerization were examined using the Tubulin Polymerization Assay Kit (Cytoskeleton Inc., Denver, CO, USA) according to the supplier’s instructions, with details described in Appendix A. [34,35].

#### 3.2.3. Cell Cycle Analysis and Apoptosis Detection

The MDA-MB231 breast cancer cell line was used for cell cycle study and apoptosis detection. The assay was performed as previously documented [52,53] (Appendix A).

## 4. Conclusions

We synthesized and evaluated a novel class of indole/1,2,4-triazole hybrids for their anticancer potential. NCI selected compounds **6h**, **7h**, **7i**, and **7j** for a five-dose assay, which showed selectivity ratios ranging from 0.52 to 2.29 at the GI_50_ values. Compounds **7i** and **7j** demonstrated the greatest efficacy among all of the novel compounds in inhibiting cancer cell proliferation, and they also showed substantial inhibitory activity against tubulin. The NO-releasing oxime moiety in compounds **7a**–**j** made them more effective against cancer than their precursor ketones **6a**–**j** in all of the tested cancer cell lines. Compound **7i** demonstrated significant cellular accumulation during the G1 phase, affirming its capacity to induce cell cycle arrest at this stage. Compound **7i** effectively induced substantial amounts of apoptosis, with a necrosis percentage of 3.88. Compounds **7i** and **7j** displayed significant antiproliferative activity as tubulin inhibitors and may serve as lead compounds for further optimization and comprehensive biological tests.

## Figures and Tables

**Table 1:** Cell growth inhibition % from the NCI’s in vitro human tumor cell lines screen for the most active compounds **6h, 7h, 7i,** and **7j**.

Subpanel Cancer Cell Lines	Compound			
	6h	7h	7i	7j
Leukemia				
CCRF-CEM	39.16	135	106.14	105.19
HL-60(TB)	56.65	113.75	136.27	125.94
K-562	72.84	98.41	111.09	93.07
MOLT-4	38.26	127.84	119.16	107.37
RPMI-8226	41.06	119.08	113.13	113.41
SR	46.96	90.21	98.71	87.18
Non-small cell lung cancer				
A549/ATCC	46.73	81.56	118.21	78.52
EKVX	46.98	71.75	163.3	97.95
HOP-62	92.2	156.56	154.84	164.1
HOP-92	107.56	105.5	111.15	108.42
NCI-H226	141.33	141.06	93.7	85.66
NCI-H23	82.67	46.7	85.88	52.69
NCI-H322M	25.12	142.43	172.69	149.35
NCI-H460	70.73	98.01	121.92	89.51
NCI-H522	98.57	76.24	129.74	87.63
Colon cancer				
COLO 205	24.98	87.63	141.19	89.79
HCC-2998	33.62	141.9	166.48	83.03
HCT-116	92.32	97.38	136.26	99.26
HCT-15	56.21	162.39	192.84	148.11
HT29	92.87	93.65	176.88	95.53
KM12	48.96	49.84	97.89	78.61
SW-620	80.99	180.39	186.34	137
CNS cancer				
SF-268	70.26	160.1	155.61	149.04
SF-295	88.94	149.9	114.97	85.93
SF-539	77.87	89.94	164.9	70.52
SNB-19	80.34	118.53	109.28	82.56
SNB-75	101.21	144.17	119.93	51.98
U251	90.22	89.6	90.27	69.48
Melanoma				
LOX IMVI	119.53	188.21	187.44	189.32
MALME-3M	64.85	83.33	110.21	88.86
M14	48.85	164.43	167.68	147.28
MDA-MB-435	61.42	200	200	199.76
SK-MEL-2	15.3	37.27	166.28	79.86
SK-MEL-28	51.05	37.46	126.35	78.78
SK-MEL-5	67.12	65.93	124.46	78.78
UACC-257	22.72	64.28	108.6	55.74
UACC-62	76.14	152.56	190.48	143.19
Ovarian cancer				
IGROV1	75.77	162.41	165.42	134.1
OVCAR-3	34.8	85.26	99.3	65.77
OVCAR-4	115.56	187.15	183.4	181.32
OVCAR-5	86.97	148.92	182.53	143.38
OVCAR-8	88.71	127.82	141.65	93.63
NCI/ADR-RES	127.71	168.42	173.87	170.19
SK-OV-3	75.97	88.51	150.17	101.58
Renal cancer				
786-0	135.44	97.83	65.25	65.86
A498	14.19	8.13	37.85	16.29
ACHN	79.61	161.68	183.52	96.66
CAKI-1	87.64	127.37	194.29	110.75
SN12C	75.12	136.7	177.16	116.18
TK-10	102.18	165.14	182.84	123.17
UO-31	69.65	93.55	96.42	65.61
Prostate cancer				
PC-3	56.97	146.54	164.4	138.01
DU-145	21.05	160.24	188.46	130.92
Breast cancer				
MCF7	87.52	154.96	142.51	96.73
MDA-MB-231/ATCC	102.39	182.05	184.18	154.53
HS 578 T	100.73	153.52	139.38	131.67
BT-549	77.64	175.54	172.67	166.72
T-47D	53.34	147.3	94.04	96.09
MDA-MB-468	52.02	188.85	194.51	185.12

**Table 2:** Screening results of compound **6h** at five dose levels in µM.

Panel	Cell Lines	GI_50_			TGI	LC_50_
		Conc. per Cell Lines	Subpanel MID ^b^	Selectivity Ratio (MID ^a^/MID ^b^)	Conc. per Cell Lines	
**Leukemia**	CCRF-CEM	54.40	33.48	0.58	˃100	>100
	HL-60(TB)	30.80			˃100	>100
	K-562	22.10			˃100	>100
	MOLT-4	21.80			˃100	>100
	RPMI-8226	29.80			˃100	>100
	SR	42.00			˃100	>100
**Non-small cell lung cancer**	A549/ATCC	13.90	14.93	1.29	48.50	>100
	EKVX	19.00			98.90	>100
	HOP-62	16.10			35.90	80.00
	HOP-92	14.70			34.70	82.10
	NCI-H226	9.22			26.10	69.80
	NCI-H23	13.70			27.50	54.90
	NCI-H322M	16.30			55.50	>100
	NCI-H460	15.20			31.00	63.50
	NCI-H522	16.30			43.70	>100
**Colon cancer**	COLO205	34.40	25.39	0.76	>100	>100
	HCC-2998	20.00			51.70	>100
	HCT-116	18.70			60.40	>100
	HCT-15	22.60			>100	>100
	HT29	15.60			50.50	>100
	KM12	36.10			>100	>100
	SW-620	30.30			>100	>100
**CNS cancer**	SF-268	24.20	17.35	1.11	78.90	>100
	SF-295	12.60			25.90	53.30
	SF-539	15.00			28.50	54.20
	SNB-19	15.80			34.50	75.30
	SNB-75	12.90			29.60	68.00
	U251	23.60			65.60	>100
**Melanoma**	LOX IMVI	14.30	21.77	0.88	27.30	5.22
	MALME-3M	16.20			36.20	8.06
	M14	32.10			>100	>100
	MDA-MB-435	22.80			65.40	>100
	SK-MEL-2	30.20			>100	>100
	SK-MEL-28	19.60			52.10	>100
	SK-MEL-5	16.50			39.10	92.40
	UACC-257	28.90			>100	>100
	UACC-62	15.30			38.10	95.10
Ovarian cancer	IGROV1	16.10	17.40	1.10	31.30	61.00
	OVCAR-3	18.00			43.20	˃100
	OVCAR-4	14.50			41.50	>100
	OVCAR-5	19.40			37.20	71.50
	OVCAR-8	24.50			˃100	>100
	NCI/ADR-RES	11.90			30.20	76.80
	SK-OV-3	17.00			33.10	64.40
Renal cancer	786-0	14.70	16.98	1.13	31.20	66.40
	A498	22.10			69.40	>100
	ACHN	13.70			29.20	62.30
	CAKI-1	14.90			29.80	59.40
	RXF393	13.60			28.90	61.50
	SN12C	19.40			48.90	>100
	TK-10	23.20			44.40	85.00
	UO-31	14.20			31.40	69.50
Prostate cancer	PC-3	12.40	8.42	2.29	37.80	>100
	DU-145	4.43			>100	>100
Breast cancer	MCF7	15.70	18.00	1.07	39.20	98.10
	MDA/MB-231-ATCC	15.40			32.60	69.00
	HS 578T	14.10			56.30	>100
	BT-549	25.10			46.70	87.20
	T-47D	24.90			˃100	>100
	MDA-MB-468	12.80			3.23	81.30
MID ^a^			19.30			

^a^ Average sensitivity of all cell lines in µM. ^b^ Average sensitivity of all cell lines of a particular subpanel in µM.

**Table 3:** Screening results of compound **7h** at five dose levels in µM.

Panel	Cell Lines	GI_50_			TGI	LC_50_
		Conc. per Cell Lines	Subpanel MID ^b^	Selectivity Ratio (MID ^a^/MID ^b^)	Conc. per Cell Lines	
Leukemia	CCRF-CEM	2.14	2.03	1.47	4.98	>100
	HL-60(TB)	1.92			3.96	nd
	K-562	2.01			4.97	>100
	MOLT-4	2.04			4.23	nd
	RPMI-8226	2.05			4.81	>100
	SR	Nd			˃100	>100
Non-small cell lung cancer	A549/ATCC	3.55	4.44	0.68	69.70	>100
	EKVX	2.15			5.58	>100
	HOP-62	1.69			3.42	6.93
	HOP-92	11.90			37.40	>100
	NCI-H226	1.63			3.36	6.91
	NCI-H23	10.1			52.20	>100
	NCI-H322M	3.54			19.80	>100
	NCI-H460	3.18			12.20	6.55
	NCI-H522	2.24			6.04	>100
Colon cancer	COLO205	3.77	3.128	0.959	37.40	>100
	HCC-2998	2.09			3.93	7.39
	HCT-116	1.87			3.65	nd
	HCT-15	1.75			3.34	6.37
	HT29	1.99			4.43	nd
	KM12	8.64			>100	>100
	SW-620	1.79			3.55	7.04
CNS cancer	SF-268	1.99	2.86	1.04	4.66	>100
	SF-295	1.64			3.18	6.15
	SF-539	2.06			4.41	9.43
	SNB-19	1.87			3.97	8.46
	SNB-75	1.40			2.77	5.48
	U251	8.22			>100	>100
Melanoma	LOX IMVI	1.75	2.78	1.08	3.17	5.73
	MALME-3M	2.26			5.57	59.1
	M14	1.79			3.86	nd
	MDA-MB-435	1.63			3.05	5.72
	SK-MEL-2	2.88			8.96	>100
	SK-MEL-28	5.67			38.7	>100
	SK-MEL-5	4.76			48.1	>100
	UACC-257	2.57			Nd	>100
	UACC-62	1.69			3.26	6.31
Ovarian cancer	IGROV1	2.09	2.24	1.34	4.35	9.07
	OVCAR-3	4.26			2.55	˃100
	OVCAR-4	1.75			3.51	7.04
	OVCAR-5	1.69			3.11	5.73
	OVCAR-8	2.19			4.89	1.49
	NCI/ADR-RES	2.00			3.90	7.59
	SK-OV-3	1.68			3.64	7.89
Renal cancer	786-0	10.00	5.77	0.52	31.40	98.10
	A498	24.08			89.10	>100
	ACHN	1.79			3.35	6.26
	CAKI-1	1.63			3.06	5.74
	RXF393	1.85			3.76	7.65
	SN12C	2.33			6.80	36.7
	TK-10	1.90			3.87	7.90
	UO-31	1.82			4.15	9.47
Prostate cancer	PC-3	1.77	1.855	1.61	3.74	nd
	DU-145	1.94			4.01	nd
Breast cancer	MCF7	2.42	1.90	1.58	7.01	>100
	MDA/MB-231-ATCC	1.77			3.33	6.27
	HS 578T	2.07			4.82	>100
	BT-549	1.59			3.03	5.78
	T-47D	2.07			4.75	>100
	MDA-MB-468	1.50			2.91	5.64
MID ^a^			3.00			

^a^ Average sensitivity of all cell lines in µM. ^b^ Average sensitivity of all cell lines of a particular subpanel in µM. nd: Not Determined.

**Table 4:** Screening results of compound **7i** at five dose levels in µM.

Panel	Cell Lines	GI_50_			TGI	LC_50_
		Conc. per Cell Lines	Subpanel MID ^b^	Selectivity Ratio (MID ^a^/MID ^b^)	Conc. per Cell Lines	
Leukemia	CCRF-CEM	2.78	2.29	1.22	nd	>100
	HL-60(TB)	1.92			4.55	>100
	K-562	2.22			nd	>100
	MOLT-4	2.09			4.80	>100
	RPMI-8226	2.45			nd	>100
	SR	Nd			>100	>100
Non-small cell lung cancer	A549/ATCC	2.49	3.23	0.87	6.66	31.00
	EKVX	2.60			9.09	34.20
	HOP-62	1.86			3.98	nd
	HOP-92	3.66			24.10	˃100
	NCI-H226	3.33			14.90	51.40
	NCI-H23	4.31			19.60	84.30
	NCI-H322M	4.80			24.20	93.30
	NCI-H460	3.53			12.90	68.40
	NCI-H522	2.52			7.70	61.60
Colon cancer	COLO205	3.80	3.19	0.88	16.60	>100
	HCC-2998	3.36			12.30	35.80
	HCT-116	3.45			17.40	>100
	HCT-15	2.11			4.72	12.10
	HT29	2.23			5.03	17.80
	KM12	4.15			29.20	>100
	SW-620	3.28			12.10	62.10
CNS cancer	SF-268	2.37	3.15	1.30	nd	>100
	SF-295	1.93			4.70	13.60
	SF-539	4.04			14.20	38.40
	SNB-19	4.32			19.80	84.50
	SNB-75	1.87			4.95	28.10
	U251	4.38			1.73	51.20
Melanoma	LOX IMVI	1.71	2.89	0.97	3.12	5.69
	MALME-3M	3.22			14.30	87.00
	M14	3.40			17.60	>100
	MDA-MB-435	2.01			3.89	7.57
	SK-MEL-2	3.10			10.4	37.50
	SK-MEL-28	3.40			12.50	44.80
	SK-MEL-5	3.55			12.80	35.80
	UACC-257	3.87			17.50	72.60
	UACC-62	1.77			3.74	7.93
Ovarian cancer	IGROV1	3.41	2.79	1.00	11.70	38.30
	OVCAR-3	4.98			23.8	˃100
	OVCAR-4	2.14			4.57	Nd
	OVCAR-5	2.18			5.21	1.59
	OVCAR-8	2.45			6.50	>100
	NCI/ADR-RES	1.90			3.92	8.08
	SK-OV-3	2.53			6.03	27.40
Renal cancer	786-0	6.85	2.87	0.98	27.00	93.40
	A498	1.63			51.70	>100
	ACHN	2.53			7.05	24.90
	CAKI-1	2.63			8.10	38.00
	RXF393	1.75			3.65	7.65
	SN12C	2.52			7.97	30.10
	TK-10	2.80			7.63	30.40
	UO-31	2.29			7.81	34.30
Prostate cancer	PC-3	2.15	2.1	1.33	4.96	>100
	DU-145	2.05			4.08	nd
Breast cancer	MCF7	3.49	2.73	1.02	15.3	63.6
	MDA/MB-231-ATCC	1.71			3.26	6.21
	HS 578T	2.84			11.90	>100
	BT-549	3.16			7.25	34.20
	T-47D	3.51			17.20	>100
	MDA-MB-468	1.72			3.30	6.32
MID ^a^			2.80			

^a^ Average sensitivity of all cell lines in µM. ^b^ Average sensitivity of all cell lines of a particular subpanel in µM.

**Table 5:** Screening results of compound **7j** at five dose levels in µM.

Panel	Cell Lines	GI_50_			TGI	LC_50_
		Conc. per Cell Lines	Subpanel MID ^b^	Selectivity Ratio (MID ^a^/MID ^b^)	Conc. per Cell Lines	
Leukemia	CCRF-CEM	2.85	2.80	1.30	nd	>100
	HL-60(TB)	2.17			6.34	>100
	K-562	2.55			nd	>100
	MOLT-4	2.16			5.69	>100
	RPMI-8226	2.38			7.29	>100
	SR	4.74			>100	>100
Non-small cell lung cancer	A549/ATCC	3.35	3.86	0.945	16.8	>100
	EKVX	3.21			19.2	>100
	HOP-62	1.74			3.65	nd
	HOP-92	3.84			37.6	˃100
	NCI-H226	3.99			1.76	61.8
	NCI-H23	6.52			>100	>100
	NCI-H322M	5.71			7.33	>100
	NCI-H460	3.33			15.7	>100
	NCI-H522	3.11			12.5	81.7
Colon cancer	COLO205	4.47	3.73	0.978	25.9	>100
	HCC-2998	4.74			2.25	>100
	HCT-116	2.92			13.7	>100
	HCT-15	3.20			13.6	>100
	HT29	3.04			11.8	>100
	KM12	4.72			>100	>100
	SW-620	3.08			16.1	83.2
CNS cancer	SF-268	2.11	4.78	0.763	5.08	>100
	SF-295	3.11			13.4	˃100
	SF-539	10.2			29.5	51.2
	SNB-19	6.89			45.2	˃100
	SNB-75	1.68			5.80	˃100
	U251	4.73			81.2	46.9
Melanoma	LOX IMVI	1.72	3.42	1.06	3.12	17.4
	MALME-3M	4.99			27.5	>100
	M14	3.15			14.1	>100
	MDA-MB-435	1.74			3.41	60.5
	SK-MEL-2	3.64			1.71	>100
	SK-MEL-28	4.41			>100	57.7
	SK-MEL-5	3.87			19.5	38.7
	UACC-257	4.67			87.8	>100
	UACC-62	2.62			10.1	>100
Ovarian cancer	IGROV1	5.35	3.61	1.01	19.8	˃100
	OVCAR-3	4.58			32.4	˃100
	OVCAR-4	1.96			4.32	>100
	OVCAR-5	3.37			11.4	˃100
	OVCAR-8	3.72			15.8	>100
	NCI/ADR-RES	2.95			9.50	>100
	SK-OV-3	3.38			1.24	71.4
Renal cancer	786-0	7.29	5.23	0.69	>100	93.5
	A498	15.4			65.8	88.6
	ACHN	3.69			14.5	˃100
	CAKI-1	2.93			12.4	˃100
	RXF393	2.22			6.29	46.3
	SN12C	3.79			16.3	>100
	TK-10	3.70			17.7	˃100
	UO-31	2.88			15.7	71.1
Prostate cancer	PC-3	2.31	2.45	1.48	7.29	>100
	DU-145	2.59			6.32	>100
Breast cancer	MCF7	3.63	2.93	1.24	21.6	22.5
	MDA/MB-231-ATCC	1.87			3.66	˃100
	HS 578T	3.09			16.6	>100
	BT-549	3.71			11.1	6.28
	T-47D	3.60			31.4	>100
	MDA-MB-468	1.72			3.47	46.0
MID ^a^			3.65			

^a^ Average sensitivity of all cell lines in µM. ^b^ Average sensitivity of all cell lines of a particular subpanel in µM.

**Table 6:** Tubulin inhibitory assay of compounds **6h**, **7h**, **7i**, and **7j**.

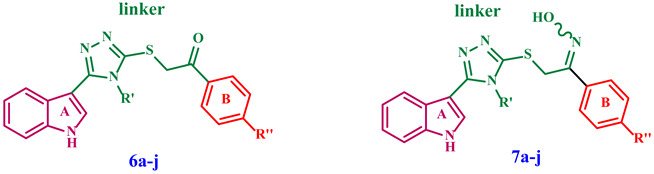				
No	Compound	R	R_1_	Tubulin Inhibitory Effect, IC_50_ (µM)
**1**	**6h**	Ph	CH_3_	9.50 ± 0.30
**2**	**7h**	Ph	CH_3_	18.37 ± 0.70
**3**	**7i**	Ph	Br	3.03 ± 0.11
**4**	**7j**	Ph	Cl	6.26 ± 0.15
**5**	**CA-4**	--	--	8.33 ± 0.29

**Table 7:** Ligand–protein complex interactions of the tested compounds **6h**, **7h**, **7i**, and **7j** within the colchicine binding site of tubulin.

Compd.	MOE Score kcal/mol	H-Bond Interactions (Å)	Hydrophobic Interactions	Pi-H Interactions (Å)
**CA-4**	−8.68	αThr179 (3.42, 3.36)	βAsn258,αAla316, βCys241, βLeu248, βLys352, βAla250, βLeu255, βAla316,	
**6h**	−7.39		βAsn258, βLys352, αThr179, βLeu255, βCys241, βLeu248, αAsn349, βAla316	αVal181 (3.95)
**7h**	−8.31	βMet259 (2.90)	βLeu255, βCys241, βLeu248, αAsn349, βAla316, βAsn258, βLys352, αThr179	αAsn101 (4.45) βLeu248 (3.84) Leu255 (3.92) Lys352 (3.67)
**7i**	−7.50	βMet259 (3.84, 3.28) Ala317 (2.70)	βLeu255, βAsn258 βCys241, βLeu248, αAsn349, βAla316, βLys352, αThr179	Leu248 (3.78) Asn258 (3.31)
**7j**	−7.75	βMet259 (3.33, 3.80)	βLeu255, βAsn258 βCys241, βLeu248, αAsn349, βAla316, βLys352, αThr179.	Leu255 (3.67) Asn258 (3.32) Lys352 (4.20)

## Data Availability

Data can be obtained on request from the authors.

## Supplementary Materials

The following supporting information can be downloaded at https://www.mdpi.com/article/10.3390/ph18020275/s1, All supplementary data to this article were submitted in a separate file.

## Appendix A

**General Details:**

Chemicals were purchased from Sigma-Aldrich (St. Louis, MO, USA), Kanto Chemical (Tokyo, Japan), Nacalai tesque (Kyoto Japan), Tokyo Chemical Industry (Tokyo, Japan), and Wako (Osaka, Japan). Thin layer chromatography (TLC) was performed on precoated plates [Merck Millipore (Burlington, MA, USA), TLC sheets silica 60 F_254_, and Fuji Silysia Chemical Ltd. (Kasugai, Japan) TLC sheets Chromatorex NH silica]. Chromatography was carried out on Silica Gel 60 N (40–100 mesh) (Fuji Silysia Chemical Ltd., Greenville, NC, USA). NMR spectra BRUKER AVANCE 600 MHz (Karlsruhe, Germany) were referenced to TMS. Infra-red spectra were recorded on a JASCO FT/IR-410 or JEOL JIR-6500 W/PerkinElmer Frontier (Gifu, Japan). The samples were prepared as KBr discs or Film or using the ATR method.

**Tubulin polymerization assay**

The activity of compounds on tubulin polymerization was investigated using the Tubulin Polymerization Assay Kit (Cytoskeleton Inc., Denver, CO, USA), which works via fluorescent reporter enhancement. The fluorescence of compounds (dissolved in DMSO at 5 and 25 µM concentration) was recorded in triplicates using FLUO star OPTIMA. Docetaxel and vincristine (Apoteket AB, Stockholm, Sweden) served as positive stabilizing and destabilizing controls. Both were used at a 3 µM concentration in PBS.
