# Supplementary material for: Design, Synthesis, and Antiproliferative Activity of Novel Indole/1,2,4-Triazole Hybrids as Tubulin Polymerization Inhibitors
Source: Pharmaceuticals (Basel). 2025 Feb 19;18(2):275. doi: 10.3390/ph18020275 (PMC11859928; doi:10.3390/ph18020275)
Supplement: Supplementary file 1 [file pharmaceuticals-18-00275-s001.zip › pharmaceuticals-3472633-supplementary.pdf]

Design, synthesis, and antiproliferative activity of novel indole/1,2,4-triazole hybrids as tubulin polymerization inhibitors

Esraa Mahmoud[1], Dalia Abdelhamid[2,3], Anber F. Mohammed[4], Zainab M. Almarhoon[5], Stefan Bräse[6]*, Bahaa G. M. youssif[4]*, Alaa M. Hayallah[4,7]*, Mohamad Abdel-Aziz[2]*

[1]Department of Pharmaceutical Chemistry, Faculty of Pharmacy, Deraya University, Minia, Egypt; [2]Department of Medicinal Chemistry, Faculty of Pharmacy, Minia University, Minia, Egypt; [3]Raabe College of Pharmacy, Ohio Northern University, Ohio, USA‡; [4]Department of Pharmaceutical Organic Chemistry, Faculty of Pharmacy, Assiut University, Assiut, Egypt; [5]Department of Chemistry, College of Science, King Saud University, Riyadh 11451, Saudi Arabia; [6]Institute of Biological and Chemical Systems, IBCS-FMS, Karlsruhe Institute of Technology, 76131 Karlsruhe, Germany., [7]Department of Pharmaceutical Chemistry, Faculty of Pharmacy, Sphinx University, New-Assiut, Egypt.

‡: Current Position

**To whom correspondence should be addressed:*

Mohamed Abdel-Aziz, Ph.D. Department of Medicinal Chemistry, Faculty of Pharmacy, Minia University, 61519-Minia, Egypt.

Tel.:(002)-01003311327; E-mail address: abulnil@hotmail.com

Alaa M. Hayallah, PhD. Faculty of Pharmacy, Sphinx University, New-Assiut, Egypt.

E-mail address: alaa_hayalah@yahoo.com

Bahaa G. M. Youssif, Ph.D. Pharmaceutical Organic Chemistry Department, Faculty of Pharmacy, Assiut University, Assiut 71526, Egypt.

Tel.: (002)-01098294419

E-mail address: bgyoussif2@gmail.com, bahaa.youssif@pharm.aun.edu.eg

Stefan Bräse

Institute of Biological and Chemical Systems, IBCS-FMS, Karlsruhe Institute of Technology,

76131 Karlsruhe, Germany. E-mail: braese@kit.edu

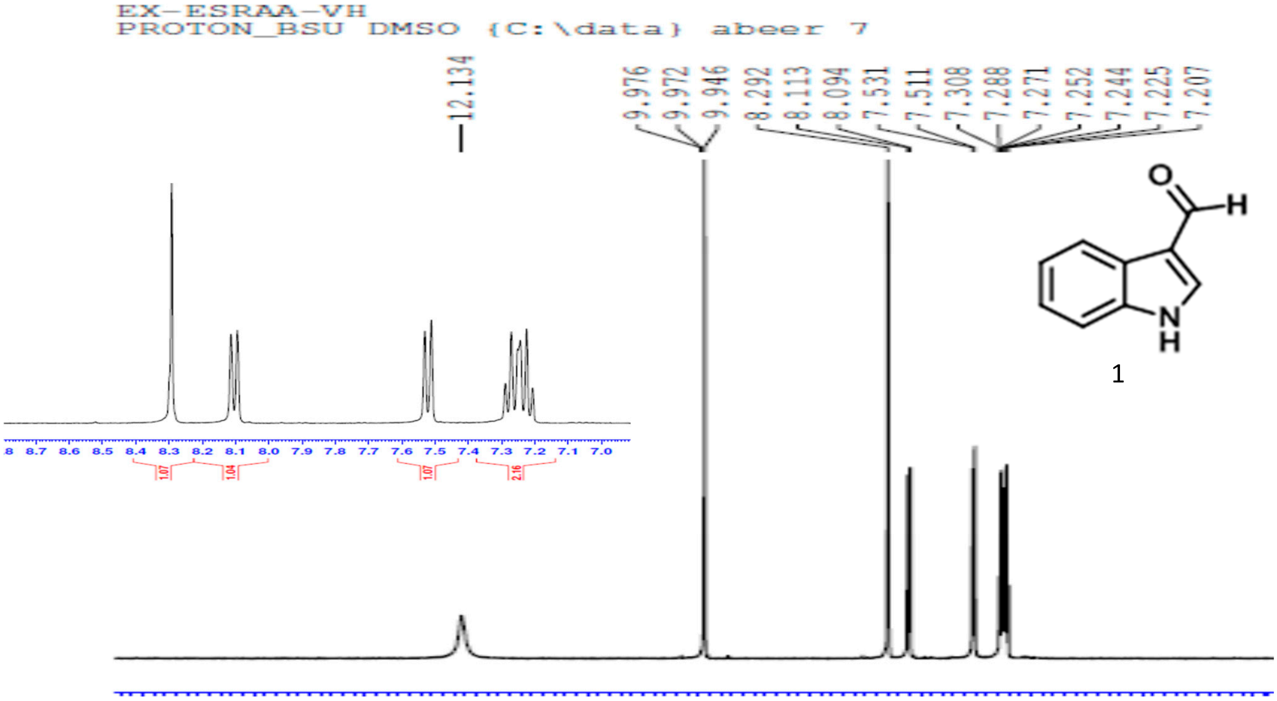

Fig. 1. ^{1}H NMR spectra of 1*H*-indole-3-carbaldehyde **(1)** (DMSO-d_6,400MHZ)

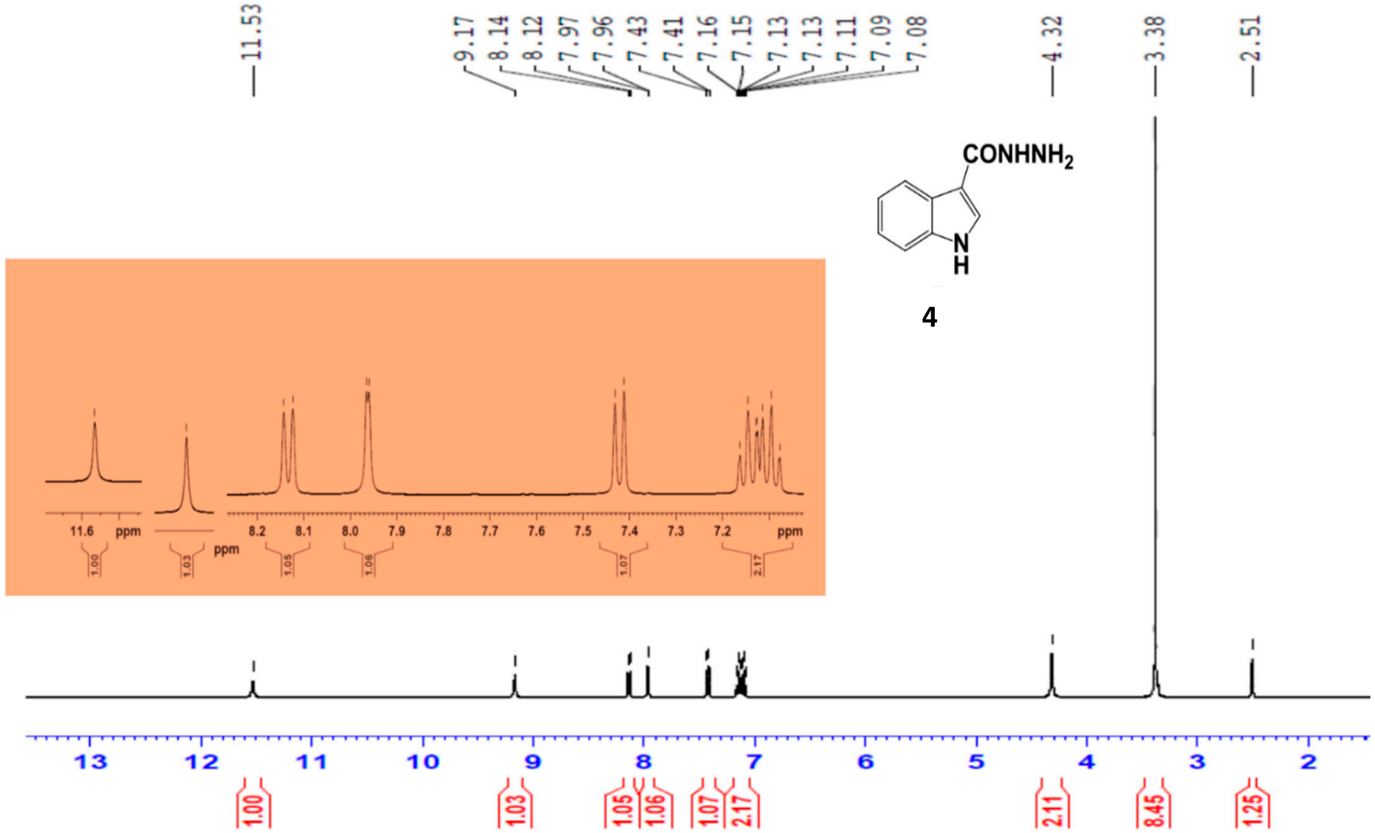

Fig.2. ^{1}H NMR spectra of 1H-indole-3-carbohydrazide **(4)** (DMSO-d_6,400MHz)

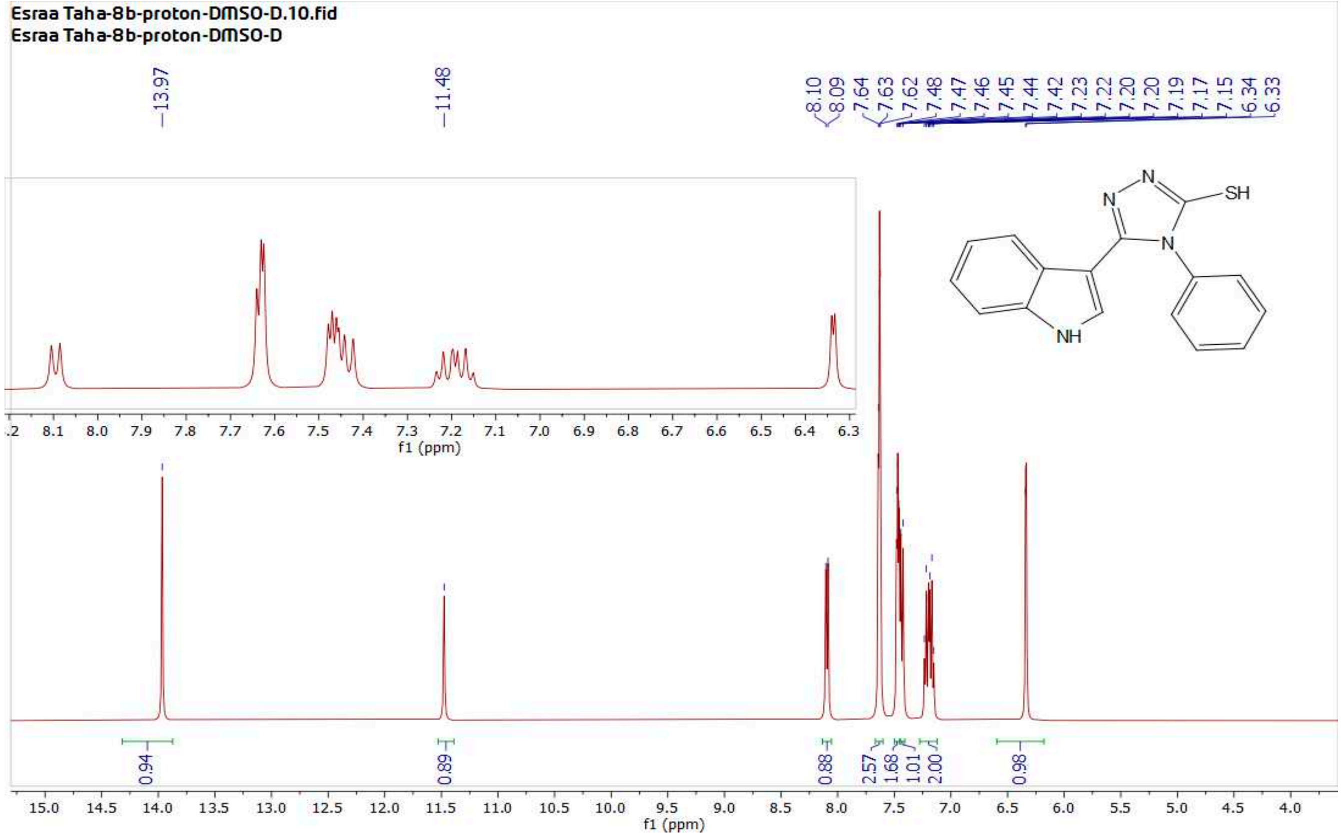

Fig. 3. ^{1}H NMR spectra of 5-(1H-indol-3-yl)-4-phenyl-4H-1,2,4-triazole-3-thiol **(5b)** (DMSO-d_6,400MHz)

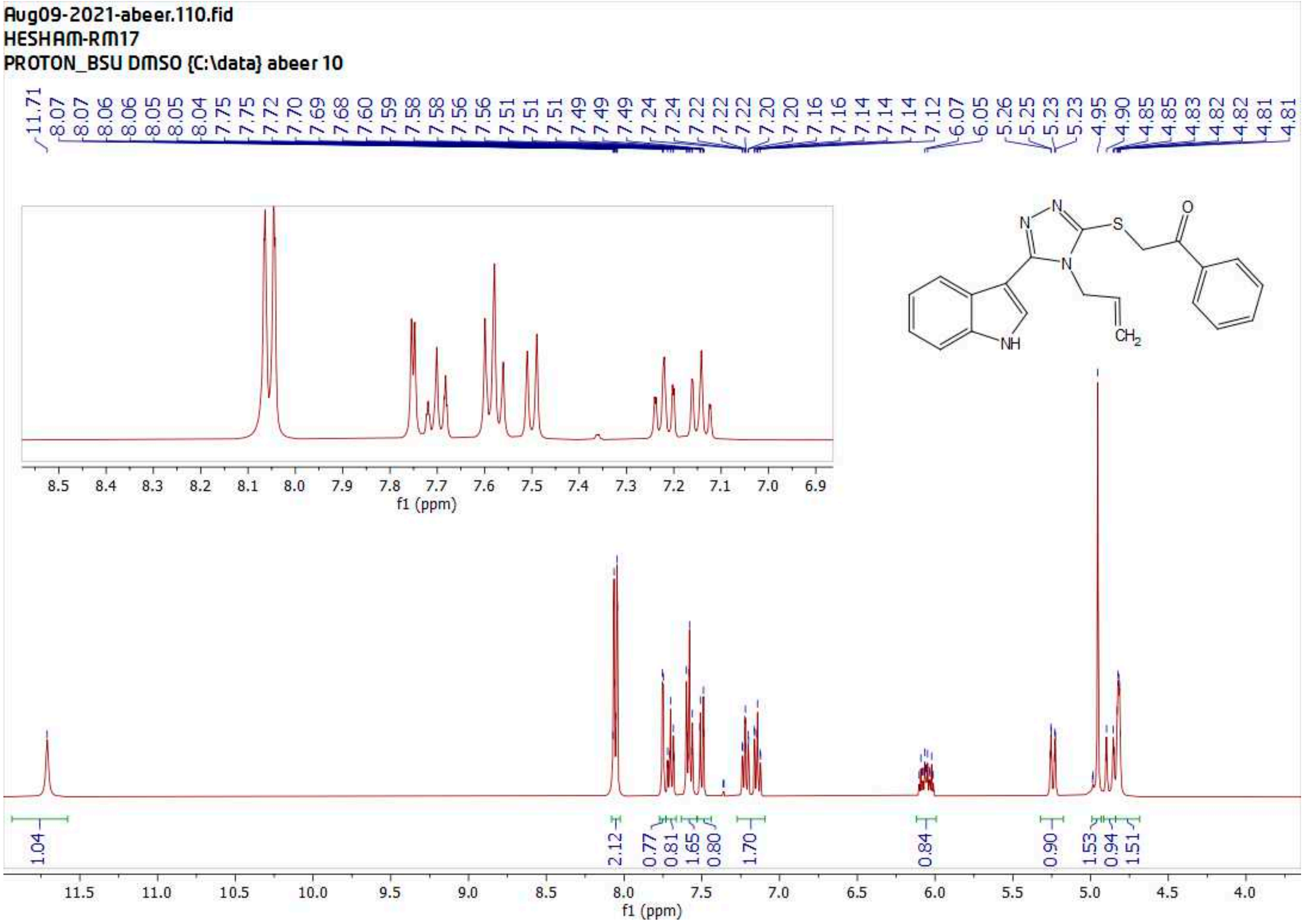

Fig. 4. ^{1}HNMR spectra of compound **6a** (DMSO-d_6,400MHZ)

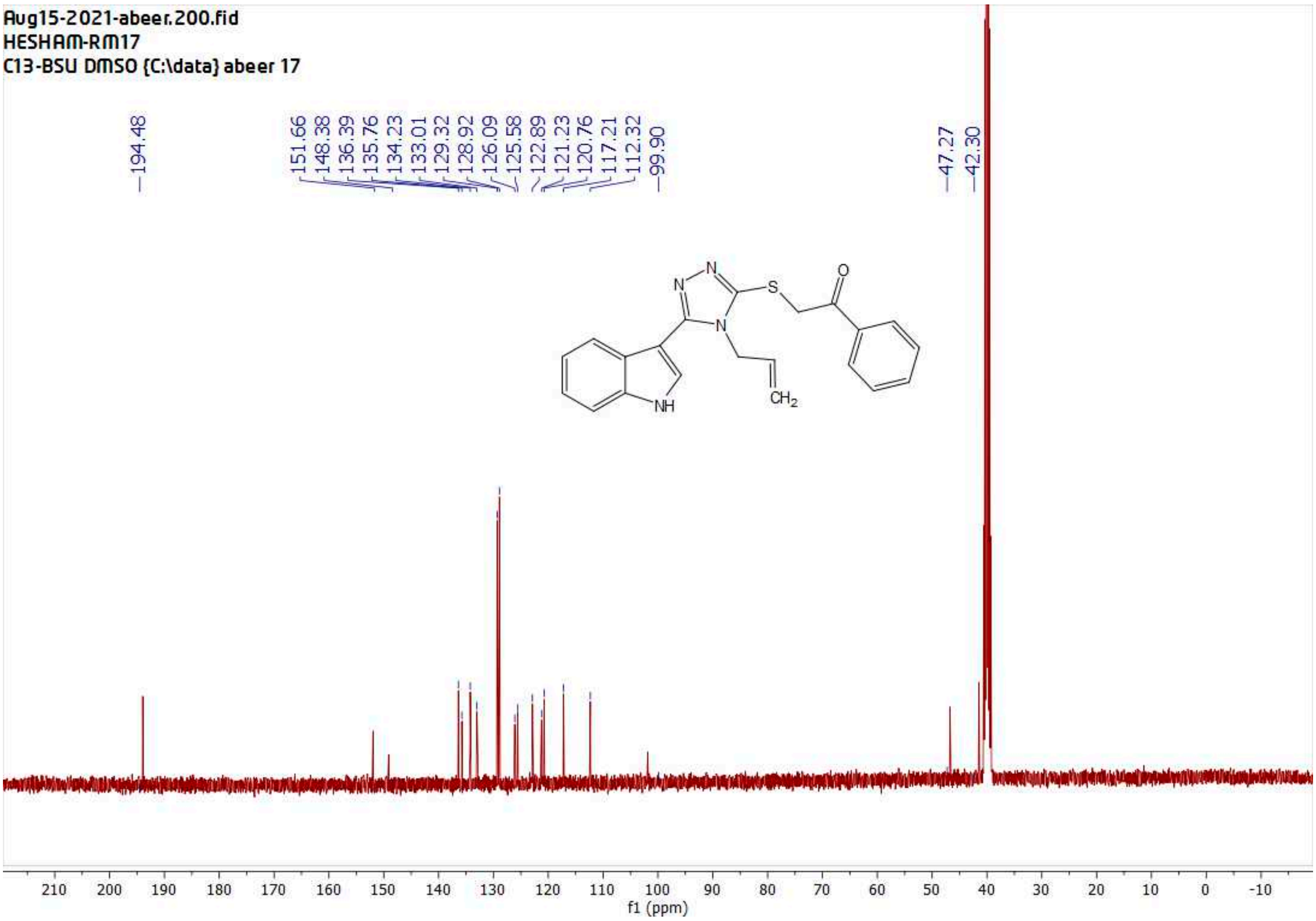

Fig. 5. ^{13}CNMR spectra of compound **6a** (DMSO-d_6,101MH)

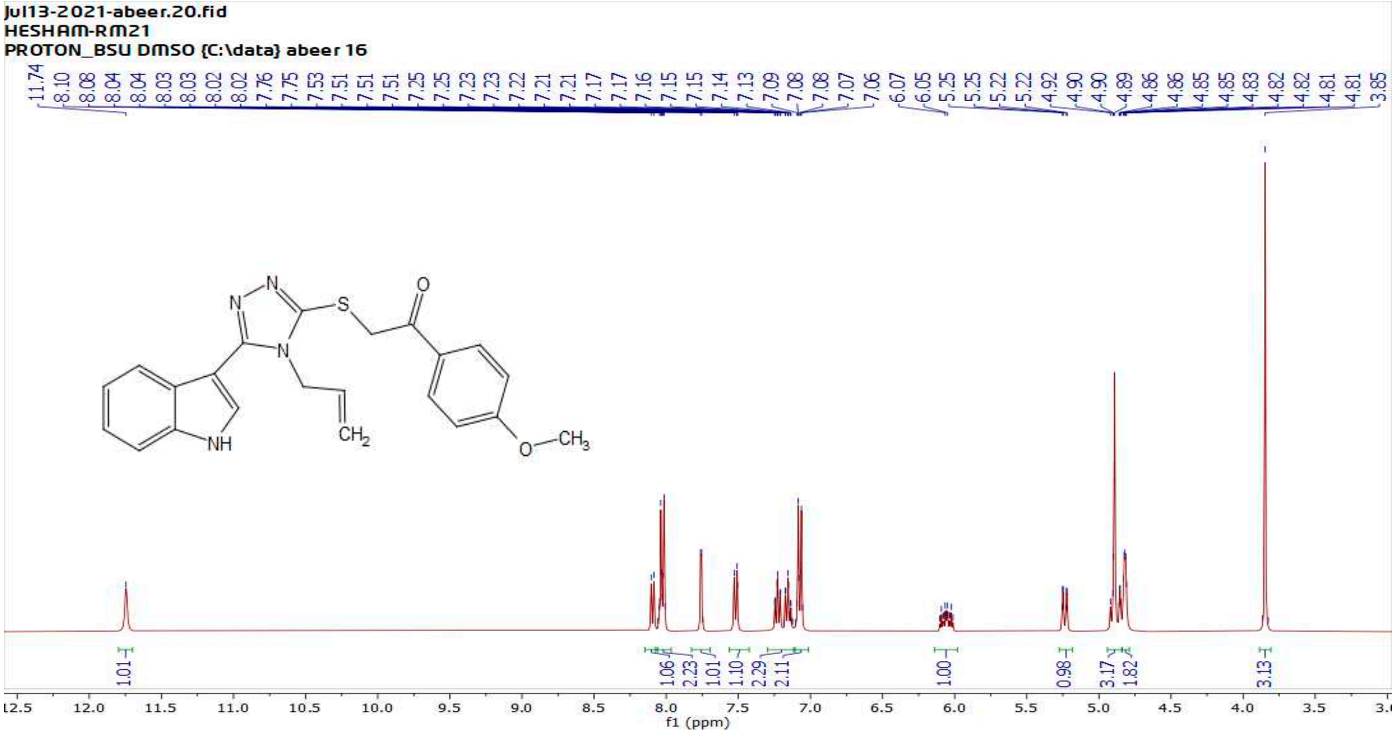

Fig. 6. ^{1}HNMR spectra of compound **6b** (DMSO-d_6,400MHZ)

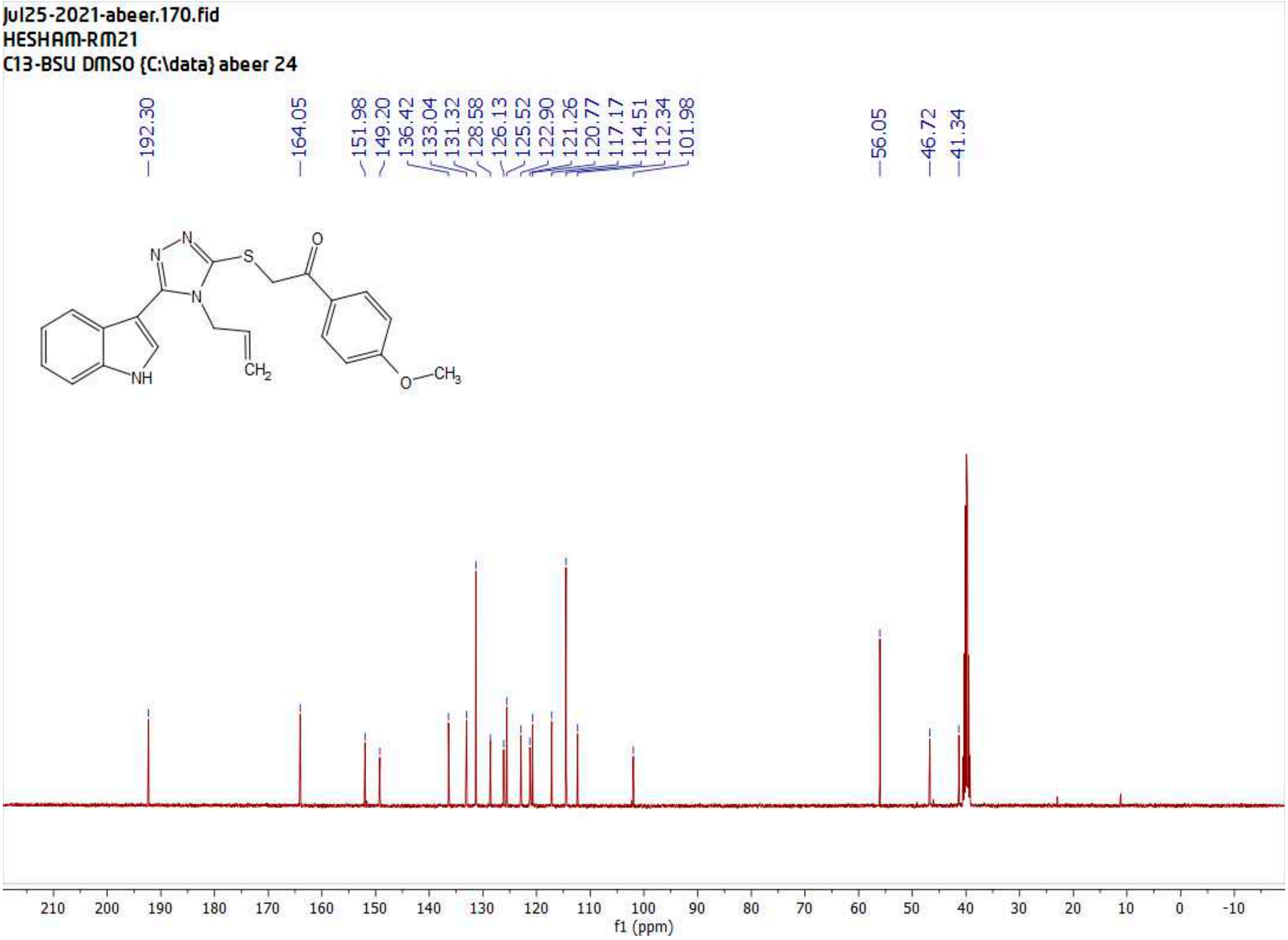

Fig. 7. ^{13}CNMR spectra of compound **6b** (DMSO-d$_6$,101MHZ)

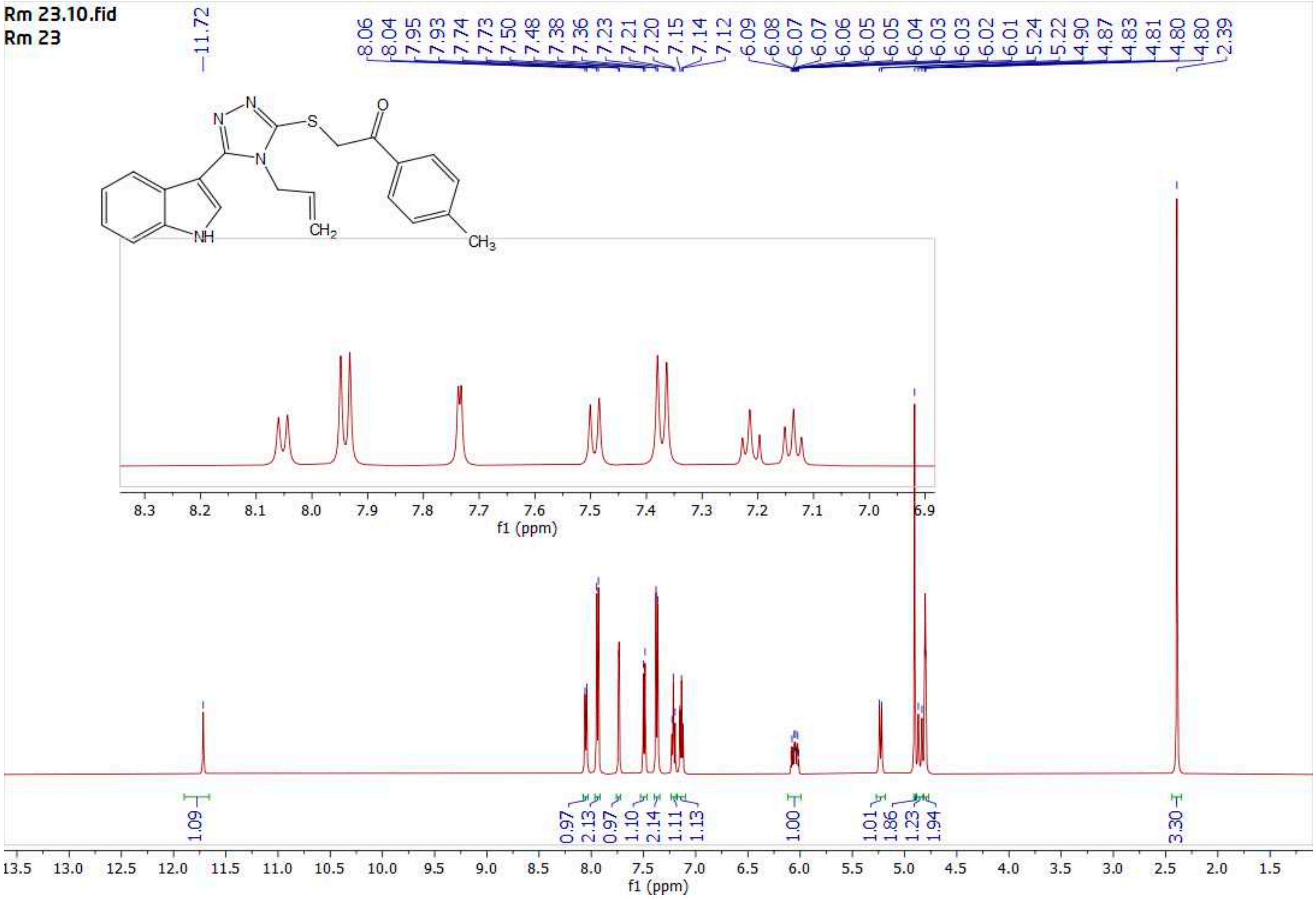

Fig. 8. ^{1}HNMR spectra of compound **6c** (DMSO-d$_6$,500MHZ)

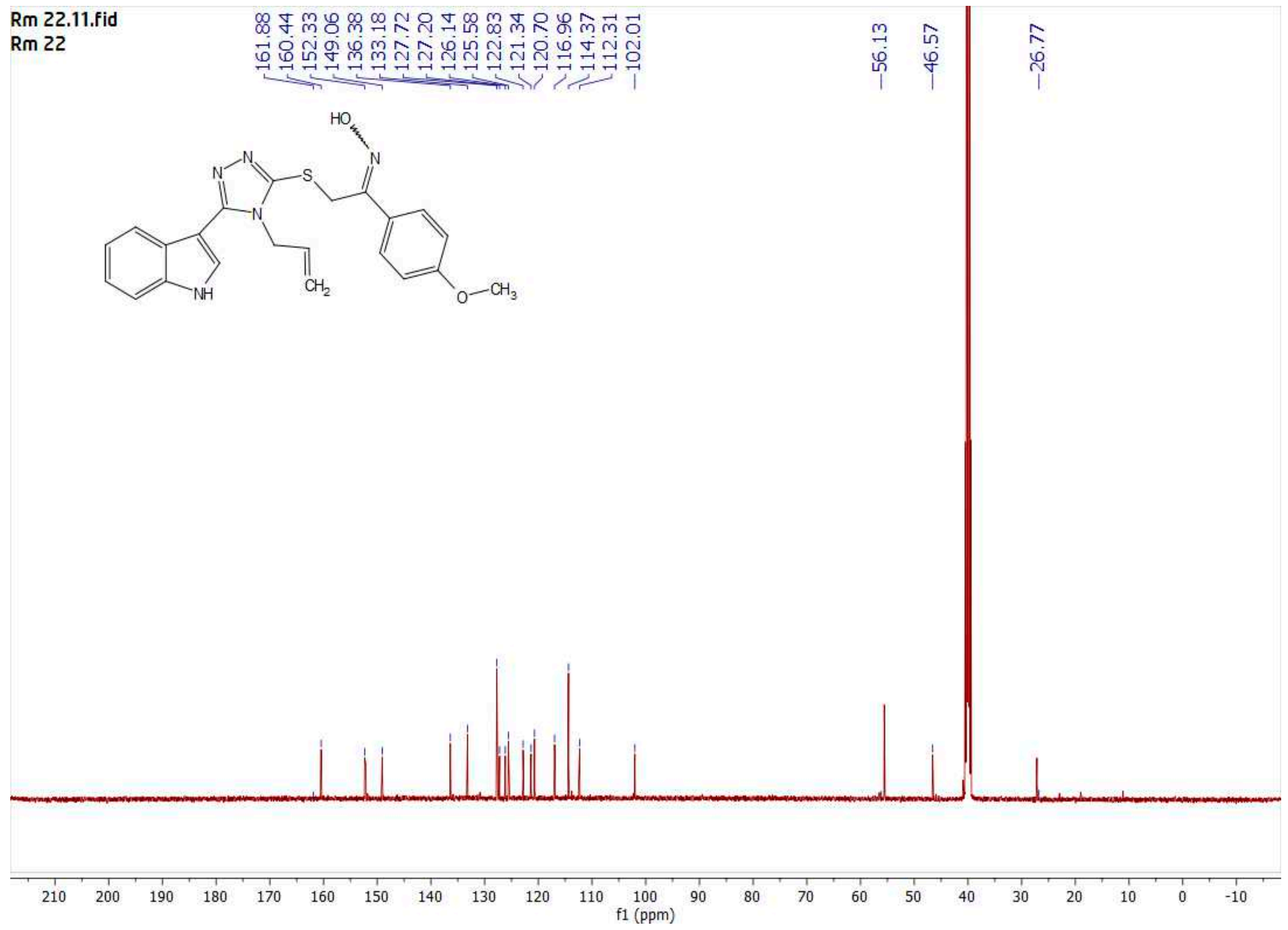

Fig.9. ^{13}CNMR spectra of compound **6c** (DMSO-d6,126MHZ)

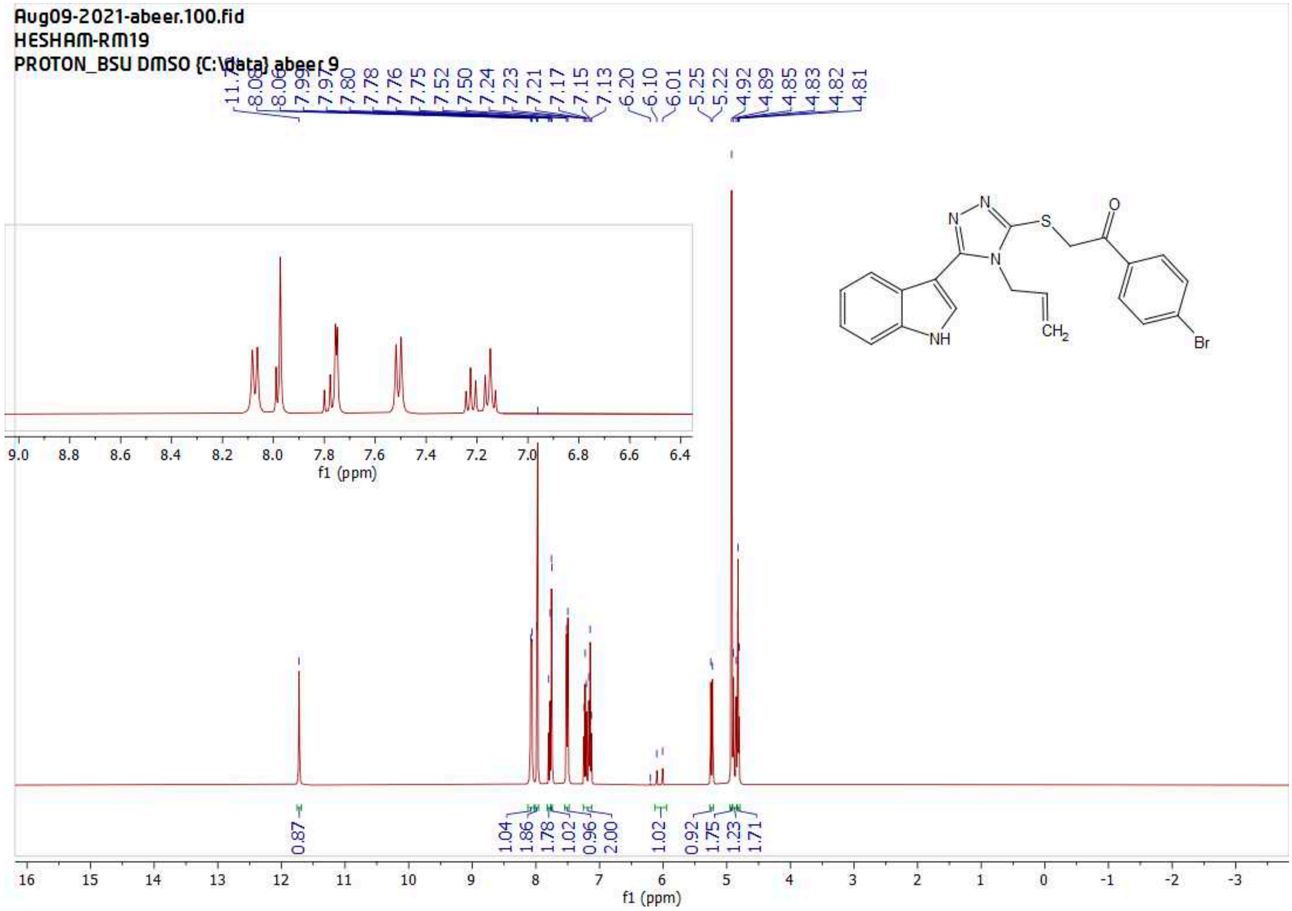

Fig. 10. ^{1}HNMR spectra of compound **6d** (DMSO-d6,400MHZ)

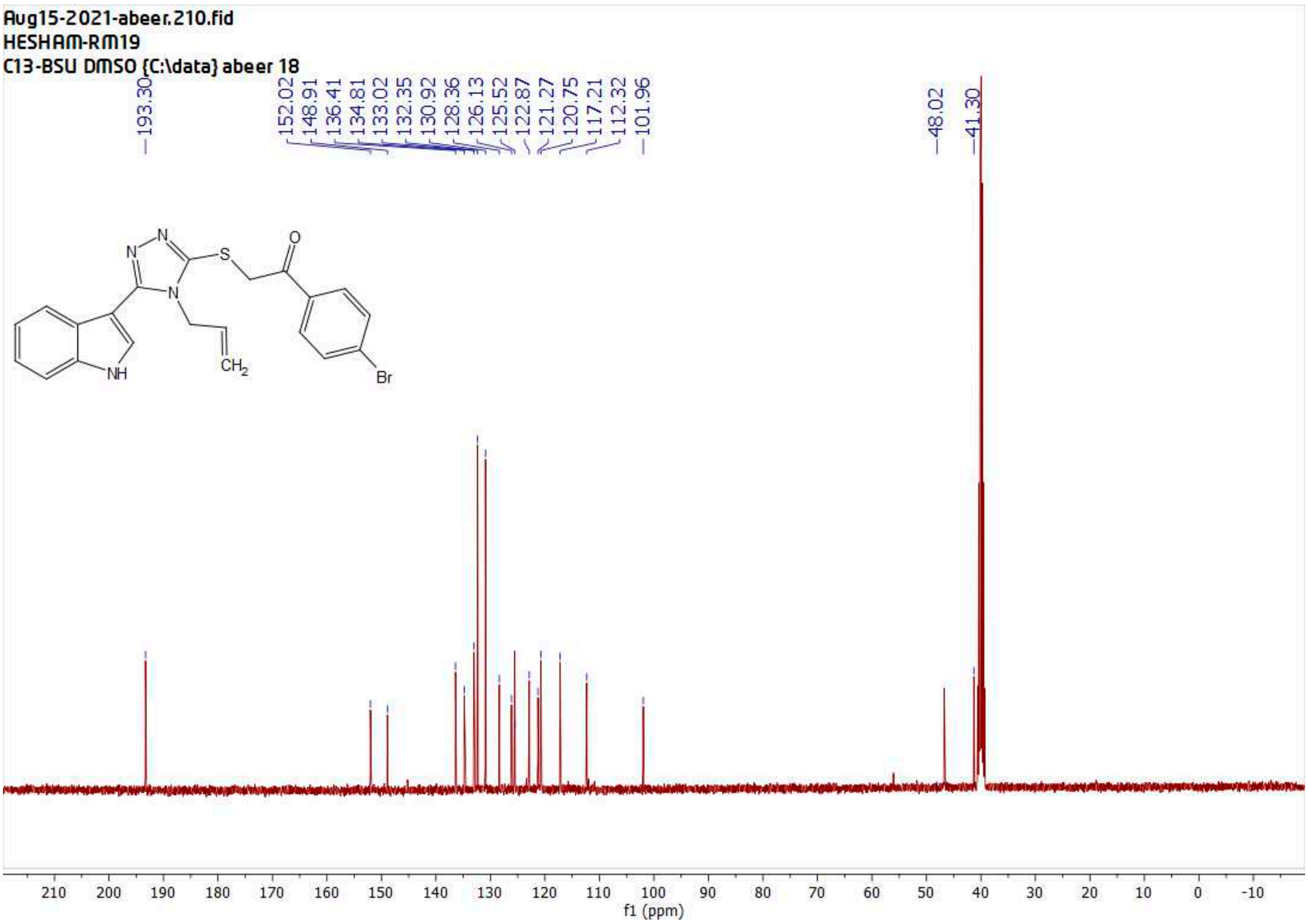

Fig. 11. ^{13}CNMR spectra of compound **6d** (DMSO-d_6,101MHZ)

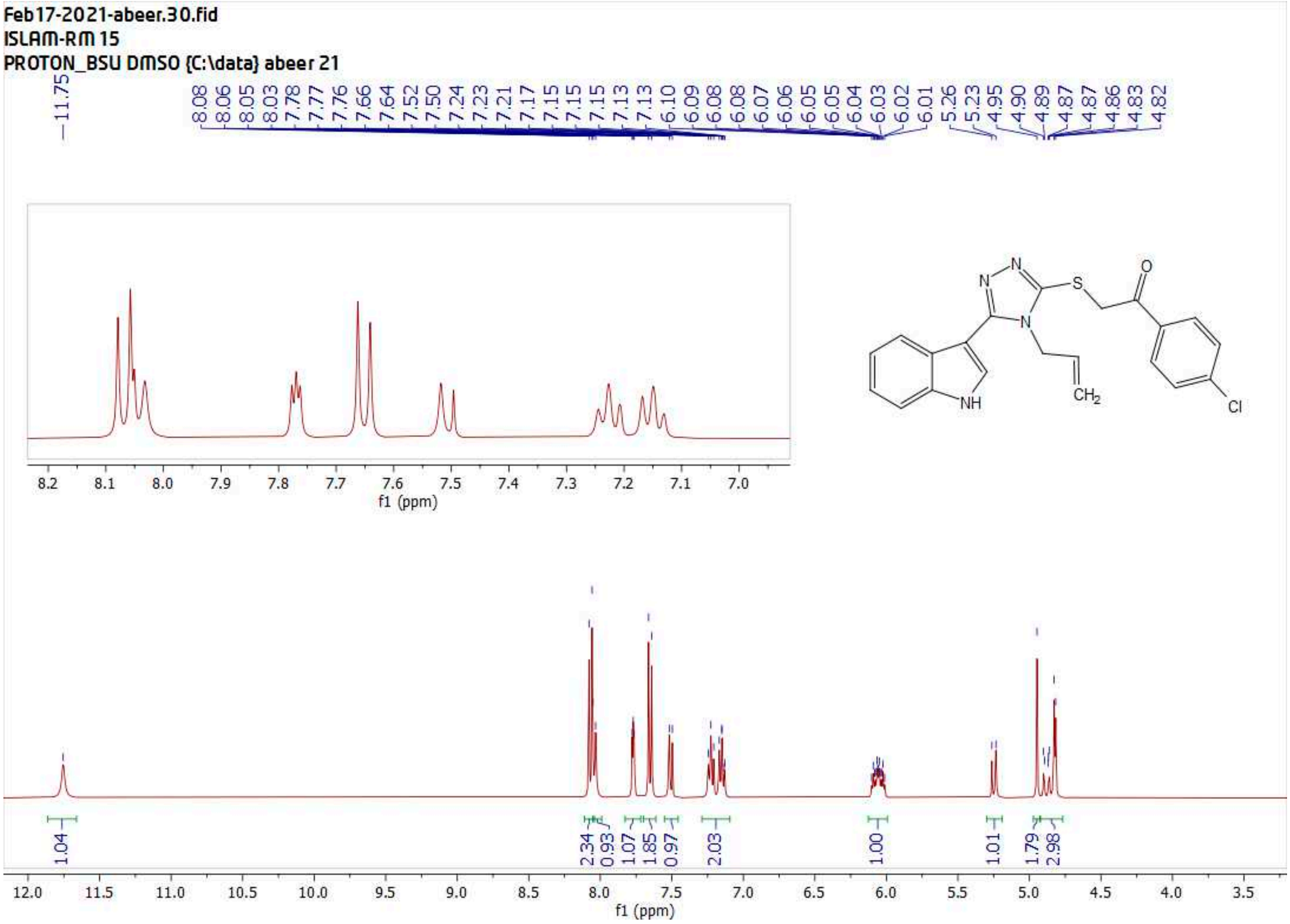

Fig. 12. ^{1}HNMR spectra of compound **6e** (DMSO-d$_6$,400MHZ)

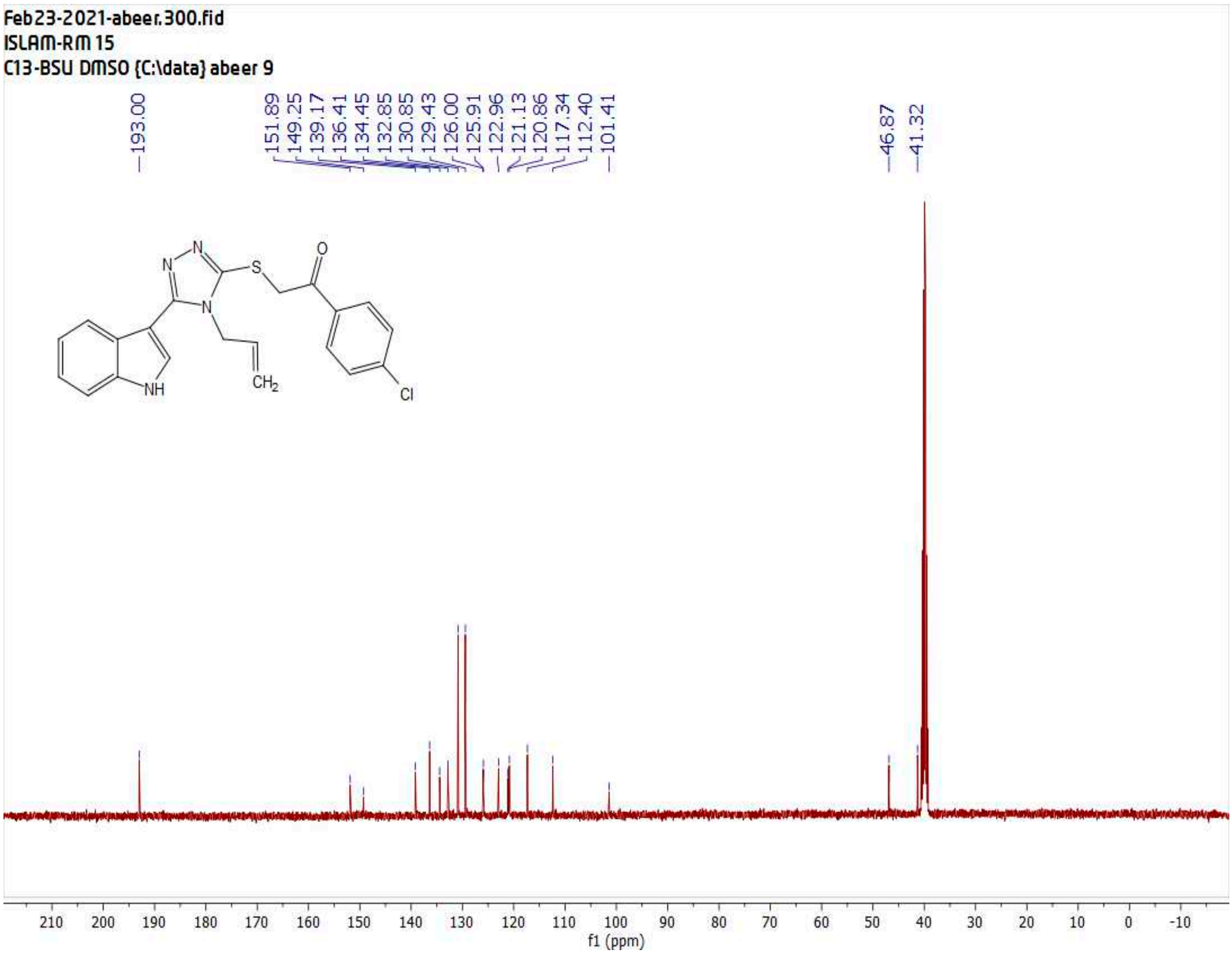

Fig. 13. ^{13}CNMR spectra of compound **6e** (DMSO-d$_6$,101MHZ)

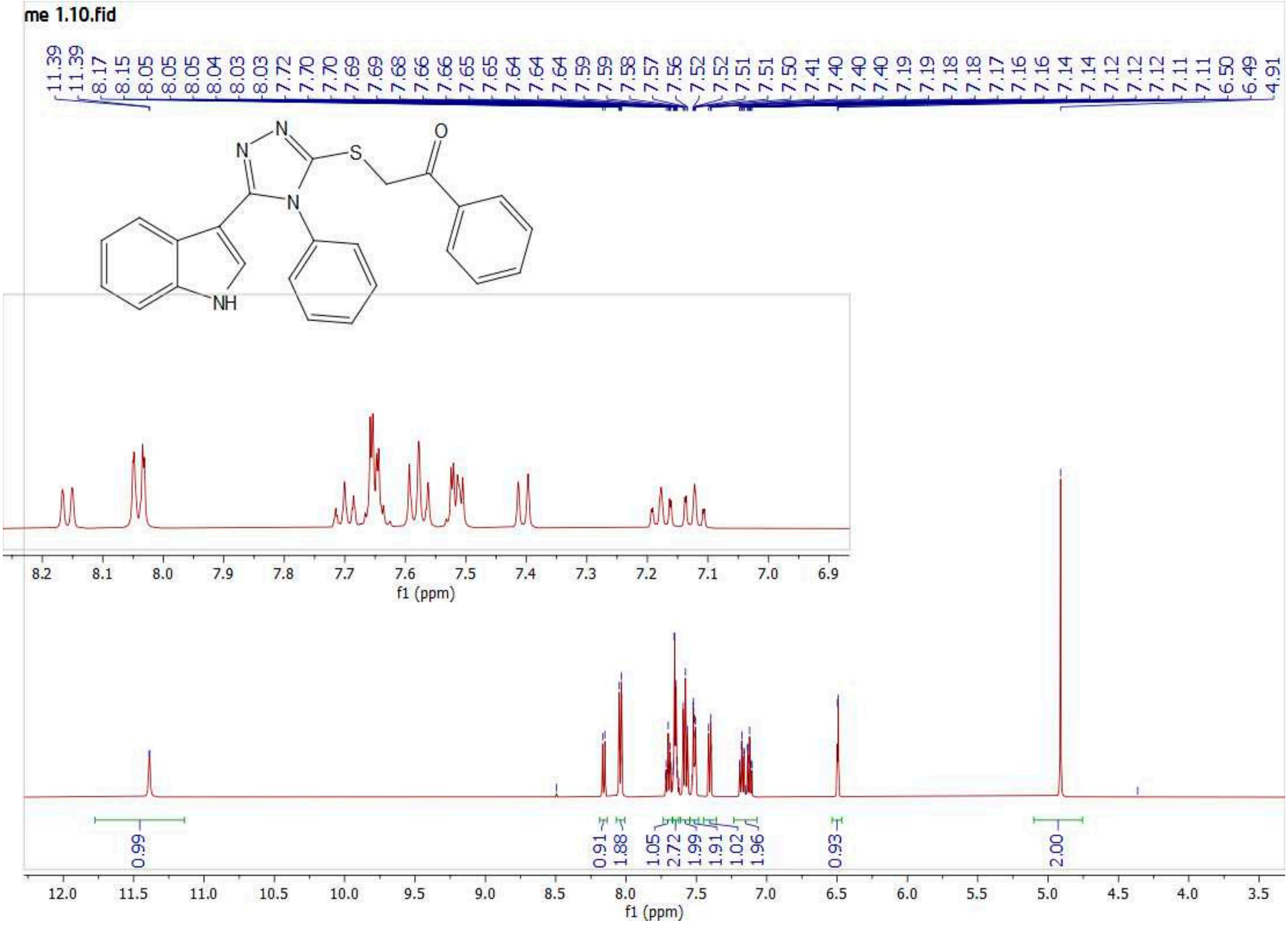

Fig.14. ^{1}HNMR spectra of compound **6f** (DMSO-d_6, 500MHZ)

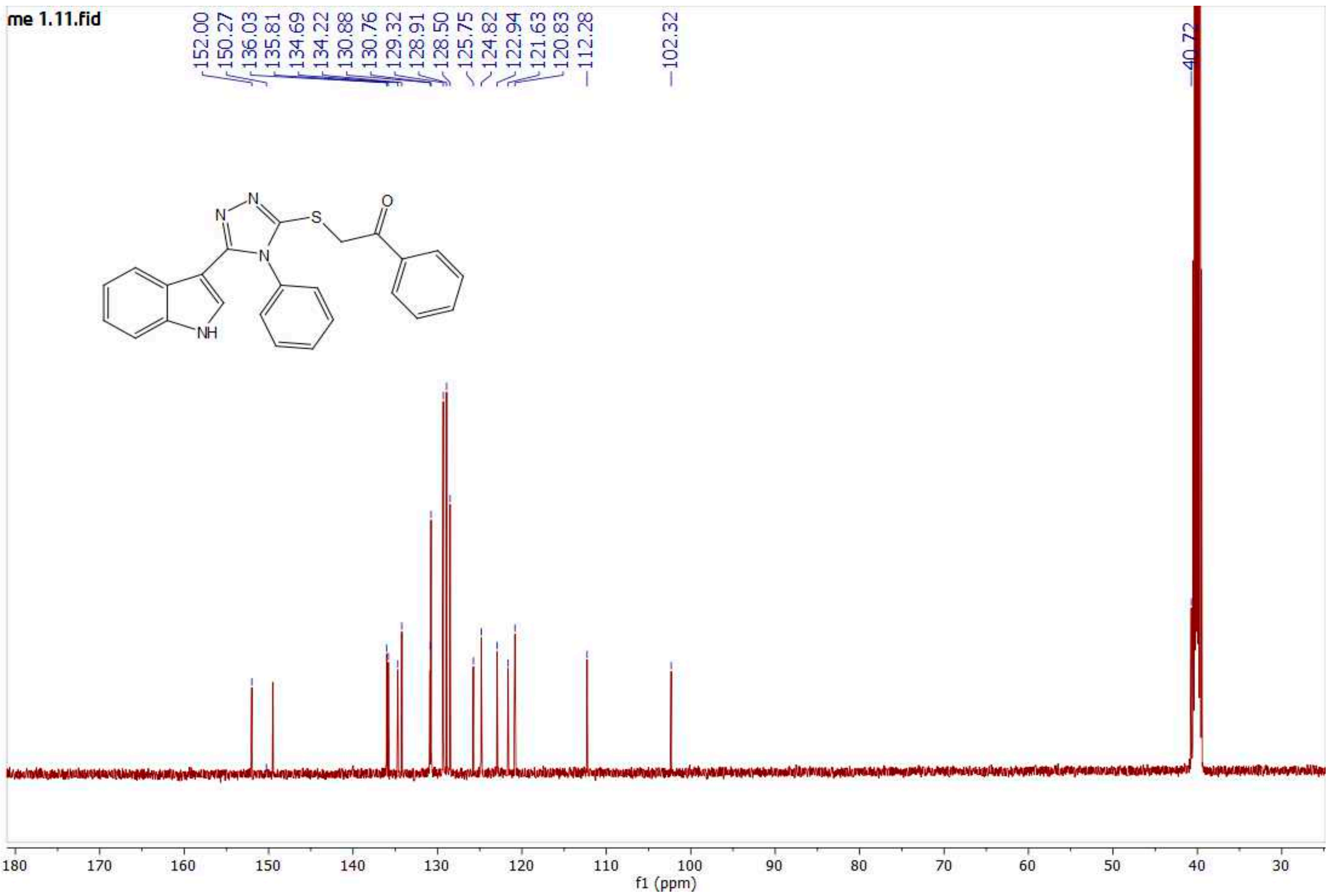

Fig. 15. ^{13}CNMR spectra of compound **6f** (DMSO-d6,126MHZ)

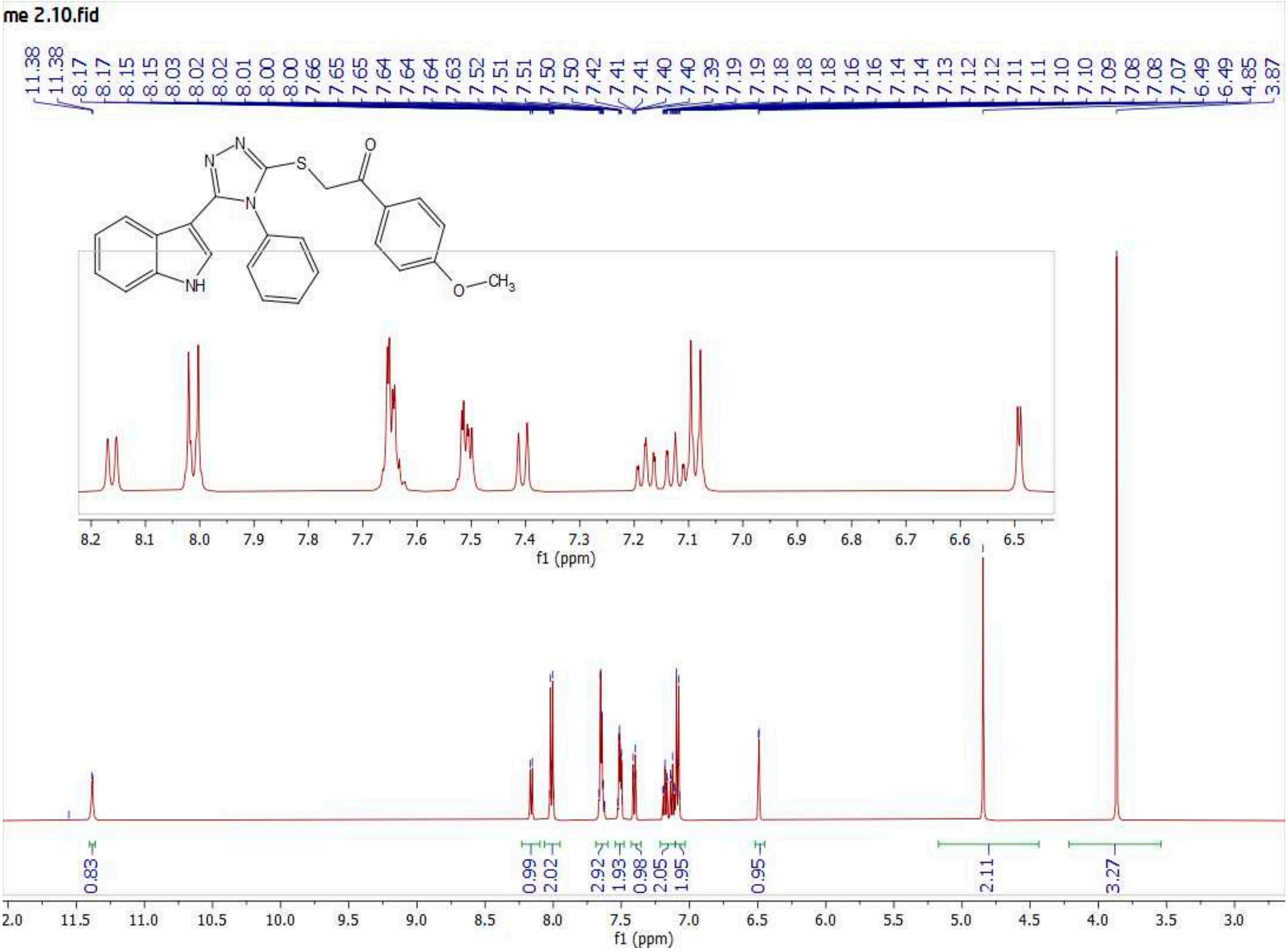

Fig. 16. ^{1}HNMR spectra of compound **6g** (DMSO-d_6, 500MHZ)

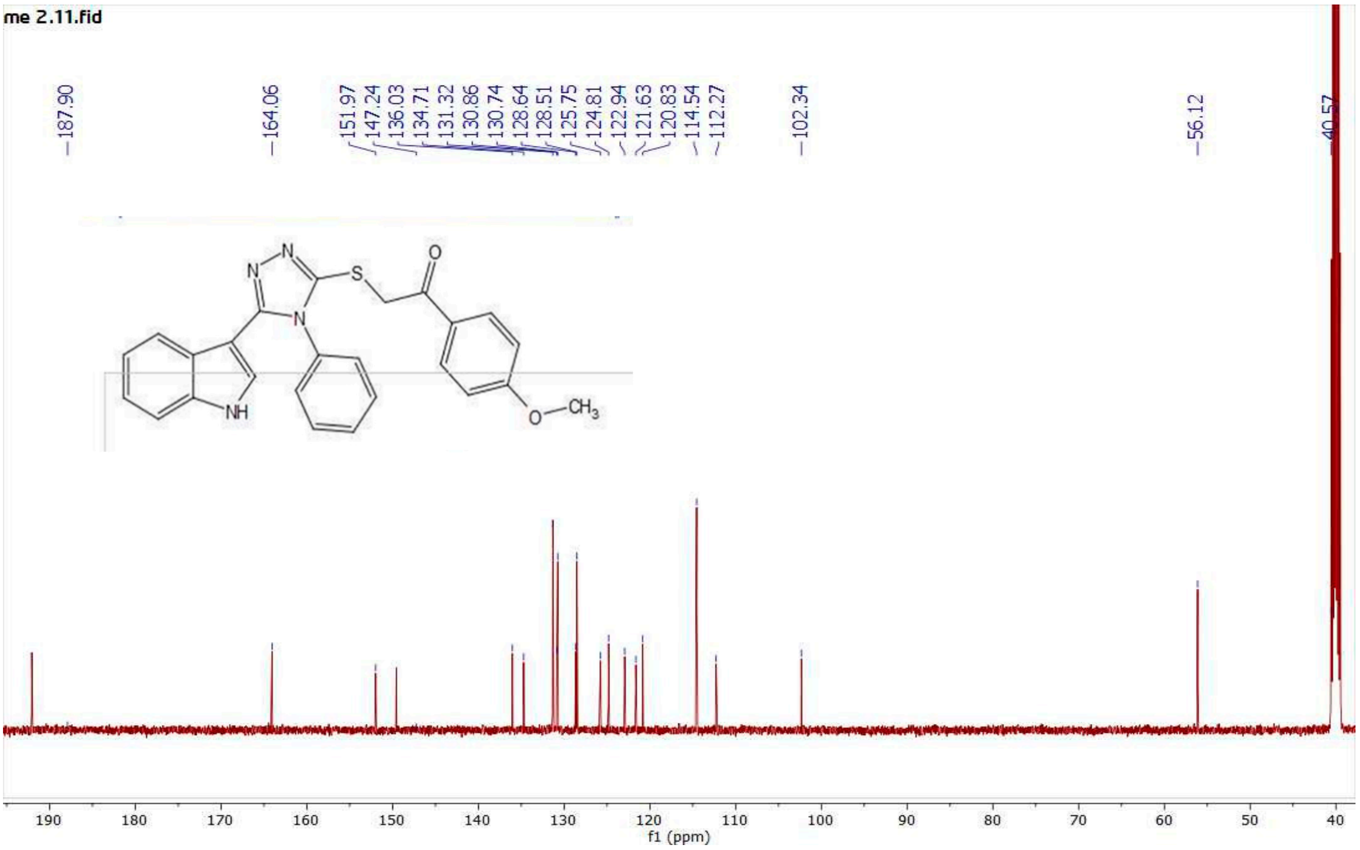

Fig. 17. ^{13}CNMR spectra of compound **6g** (DMSO-d_6,126MHZ)

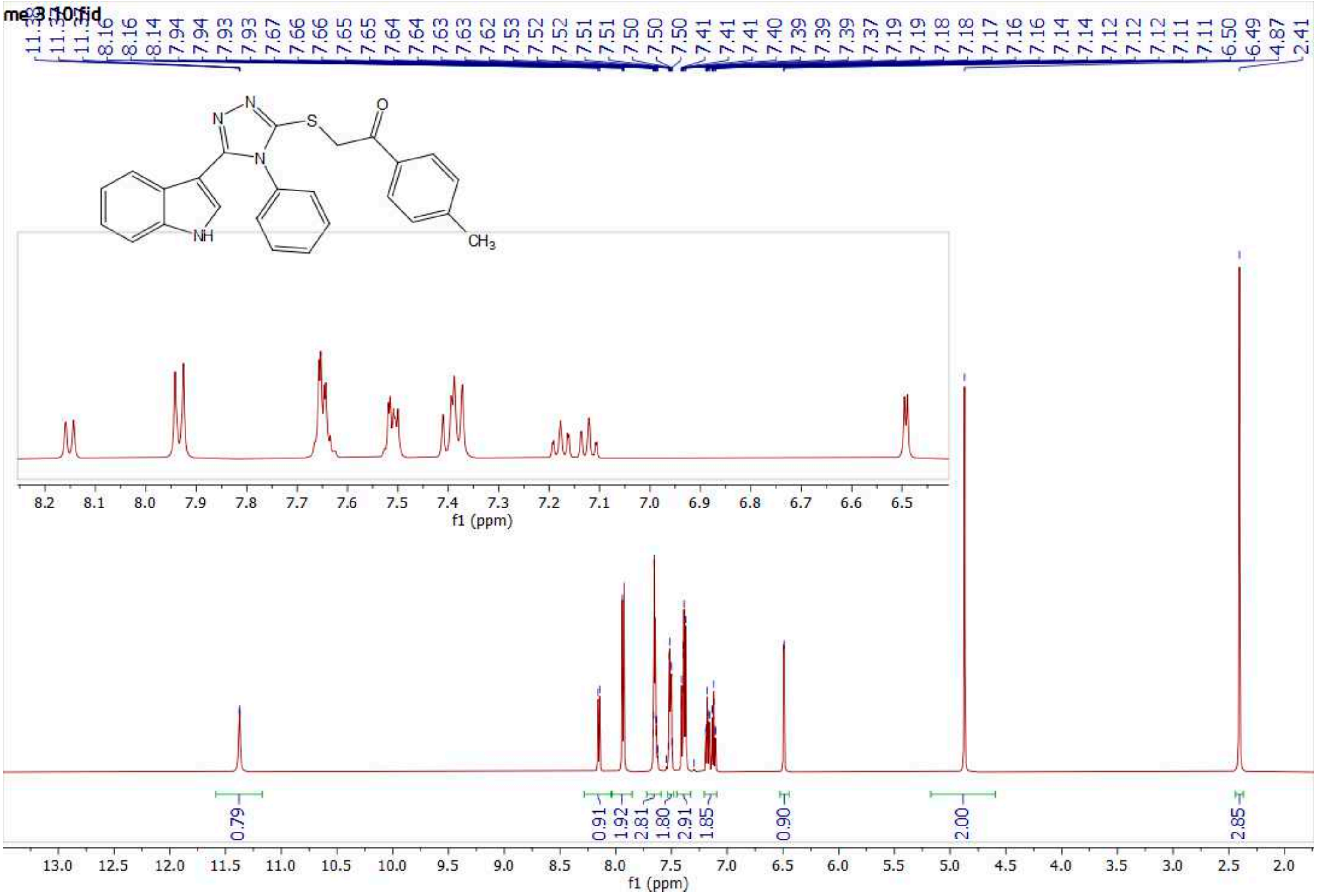

Fig. 18. ^{1}HNMR spectra of compound **6h** (DMSO-d_6, 500MHZ)

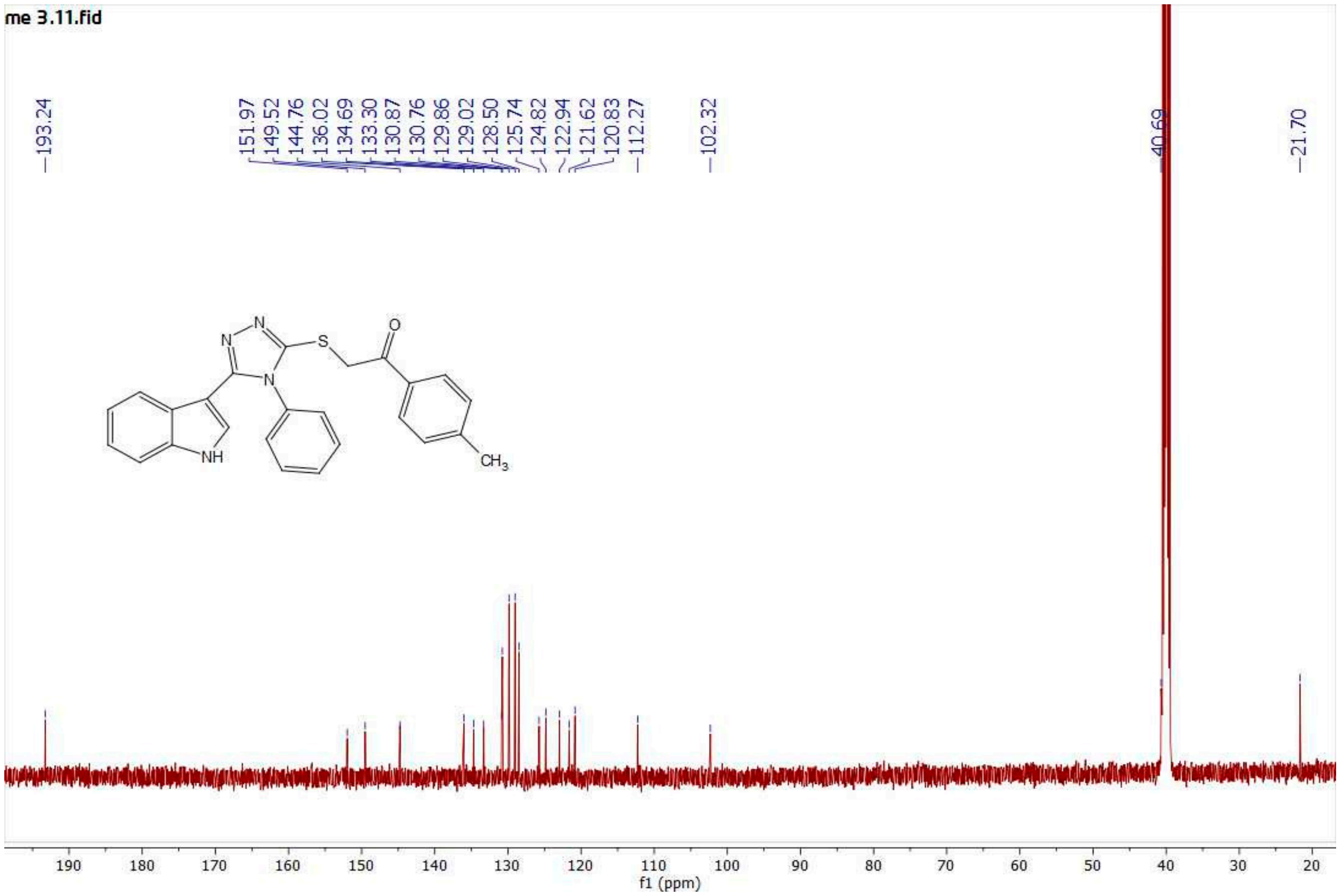

Fig. 19. ^{13}CNMR spectra of compound **6h** (DMSO-d_6,126MHZ)

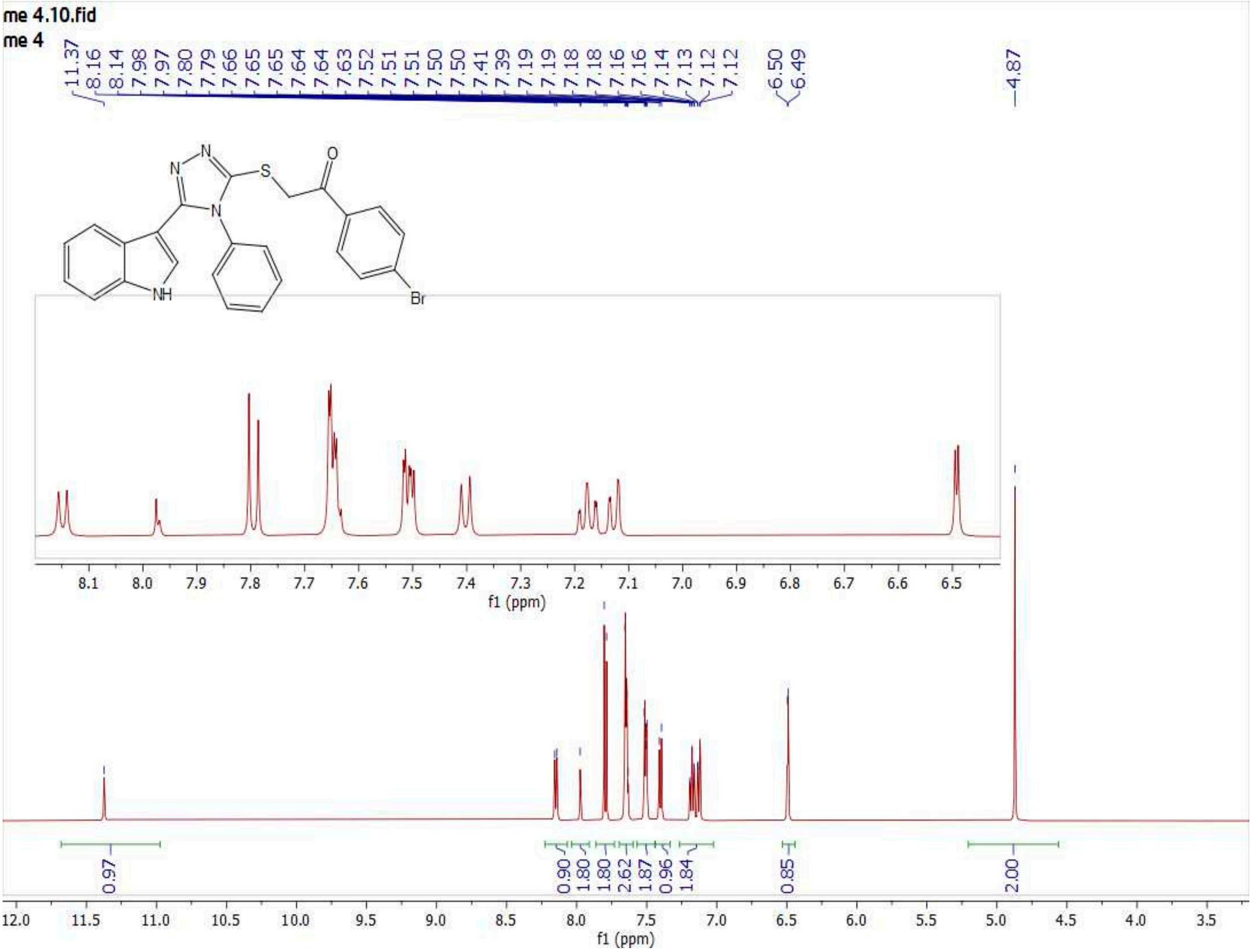

Fig. 20. ^{1}HNMR spectra of compound **6i** (DMSO-d_6, 500MHZ)

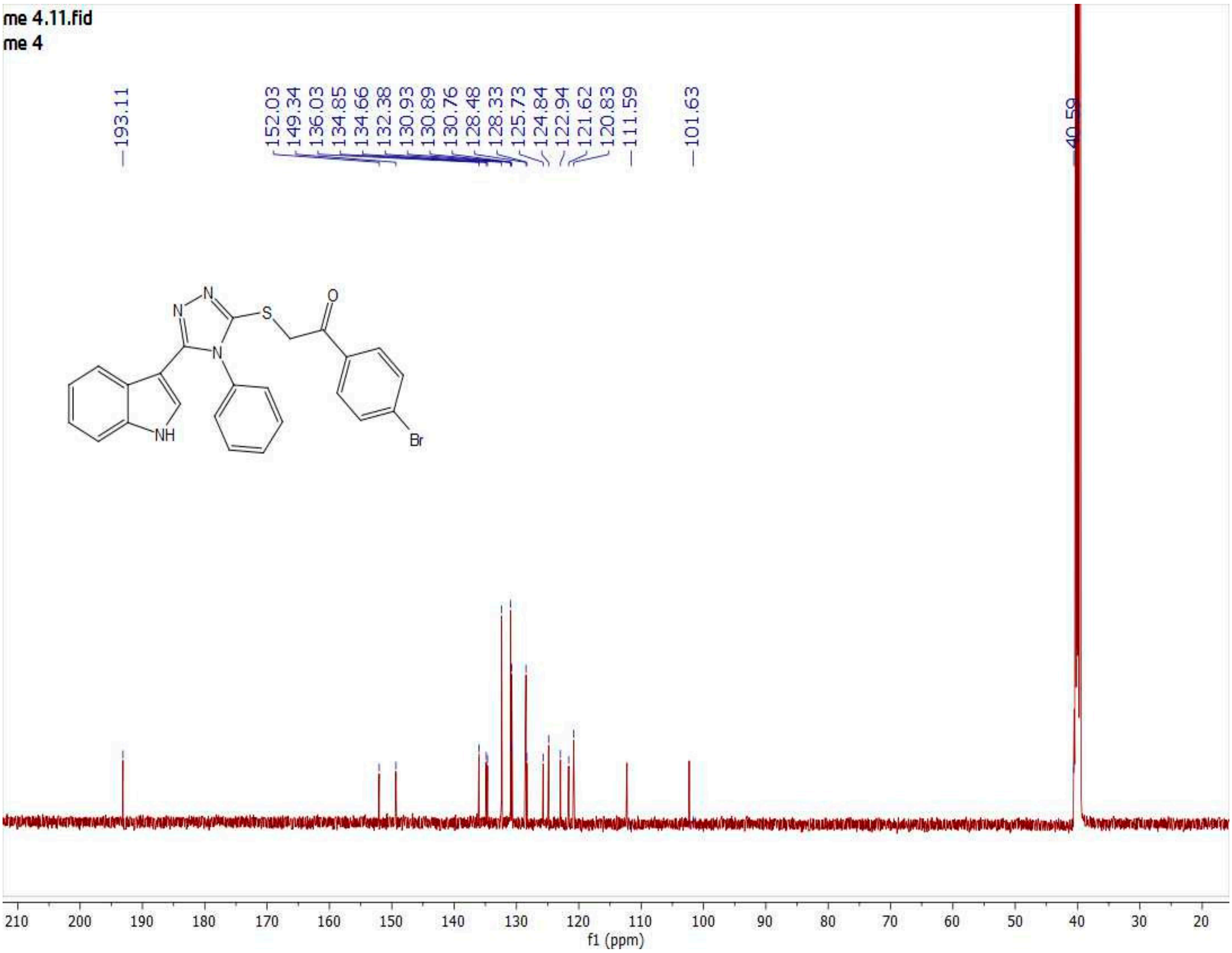

Fig. 21. ^{13}CNMR spectra of compound **6i** (DMSO-d$_6$,126MHZ)

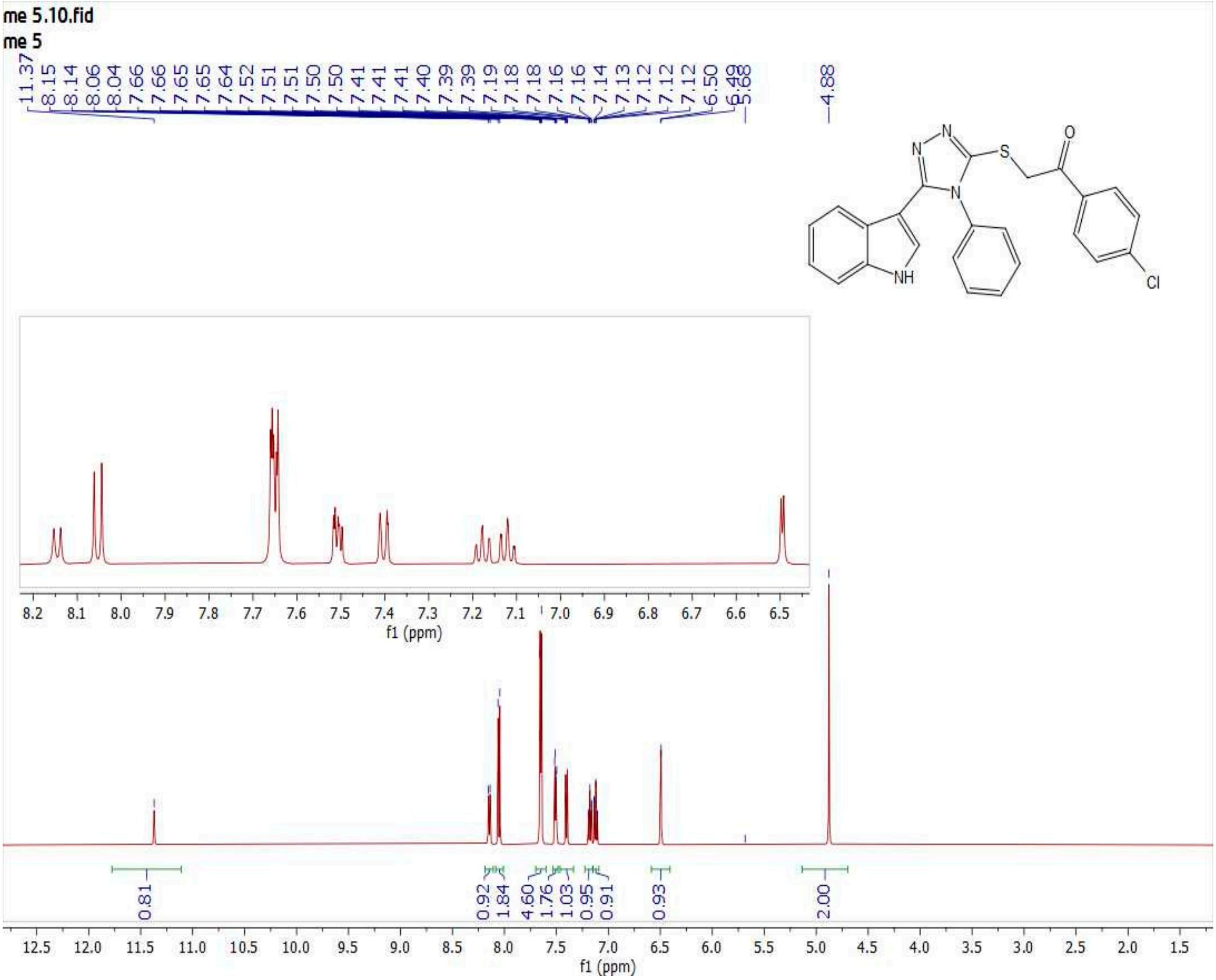

Fig. 22. ^{1}HNMR spectra of compound **6j** (DMSO-d$_6$, 500MHZ)

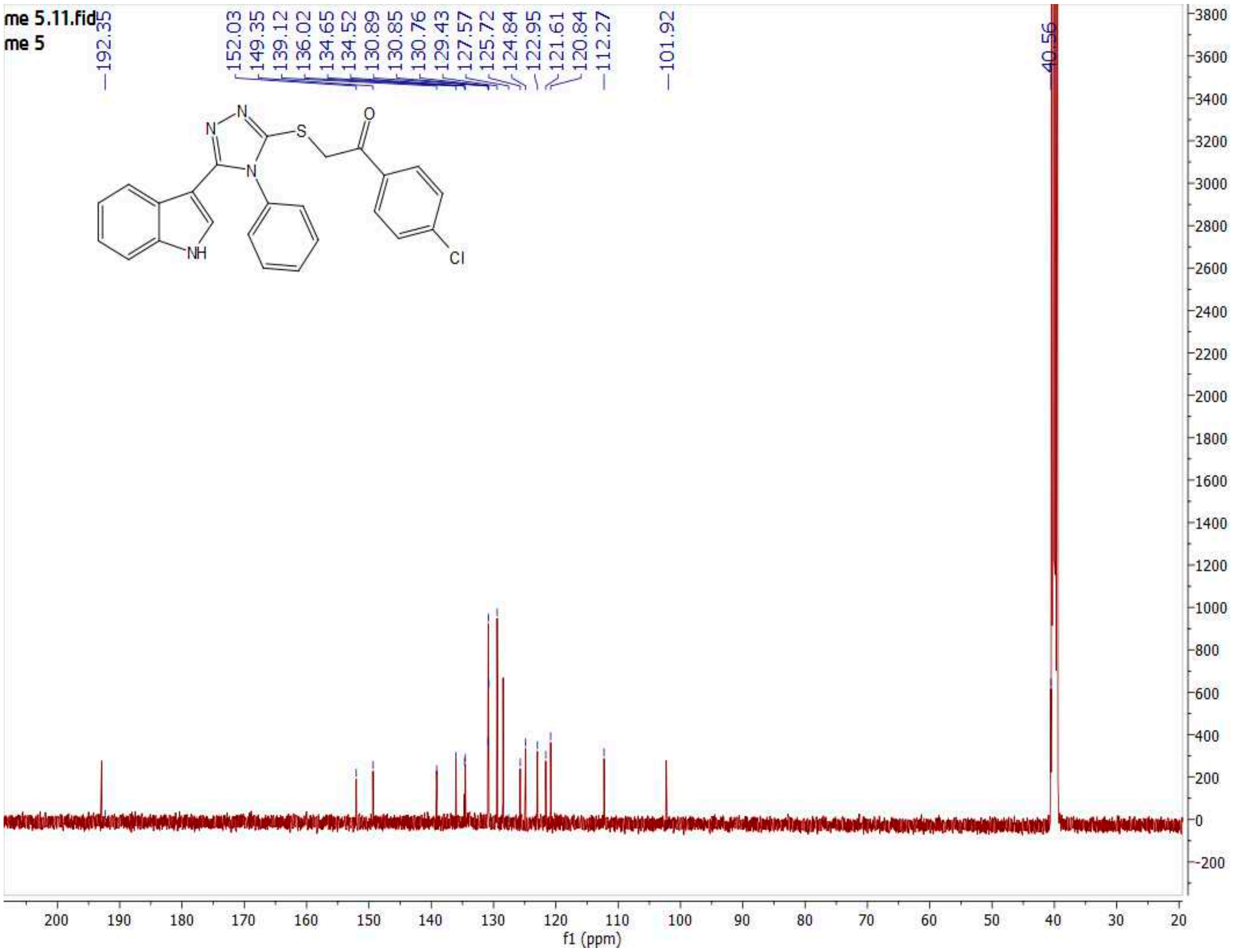

Fig.23. ^{13}CNMR spectra of compound **6j** (DMSO-d6,126MHZ)

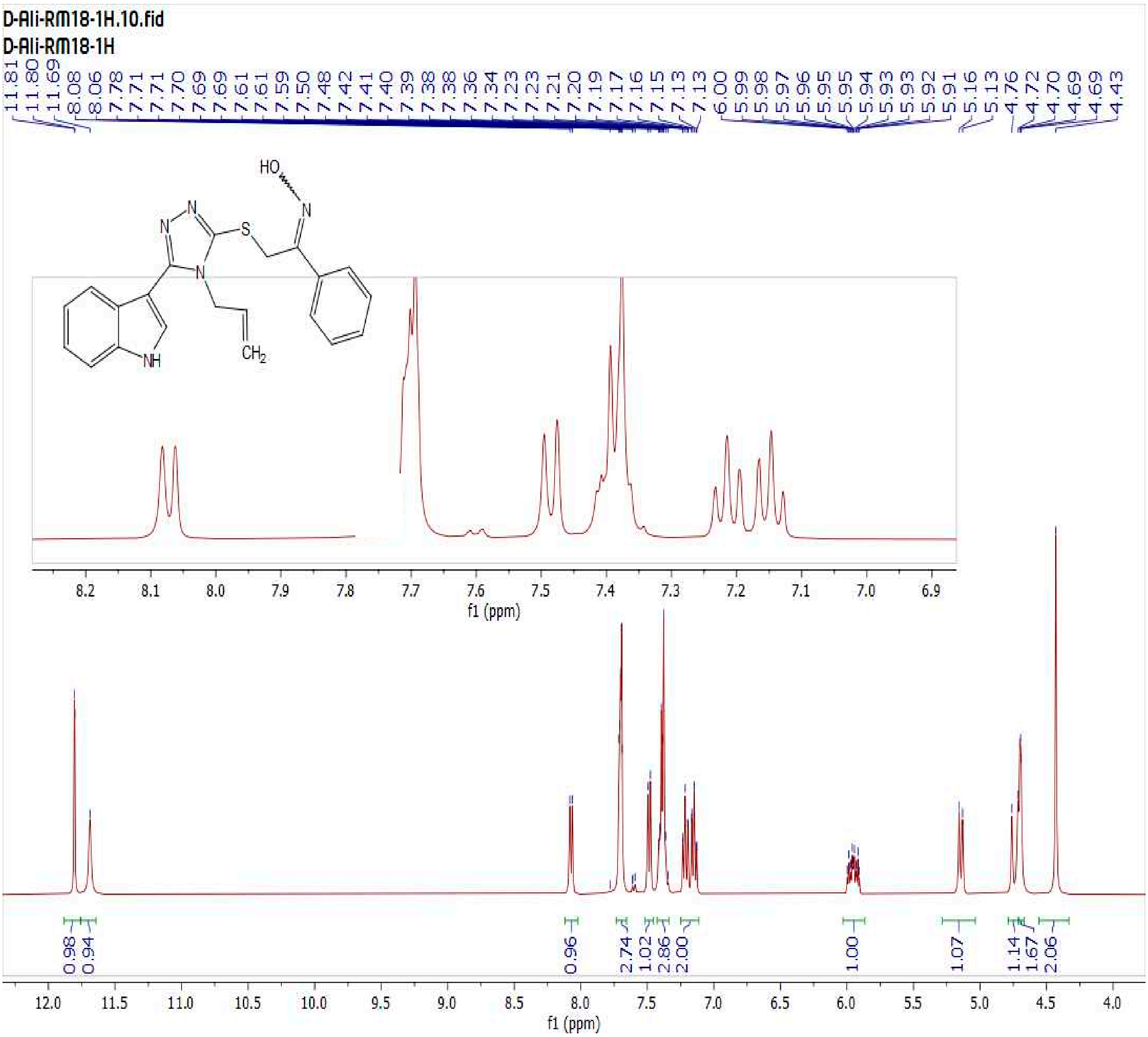

Fig. 24. ^{1}HNMR spectra of compound **7a** (DMSO-d6,400MHZ)

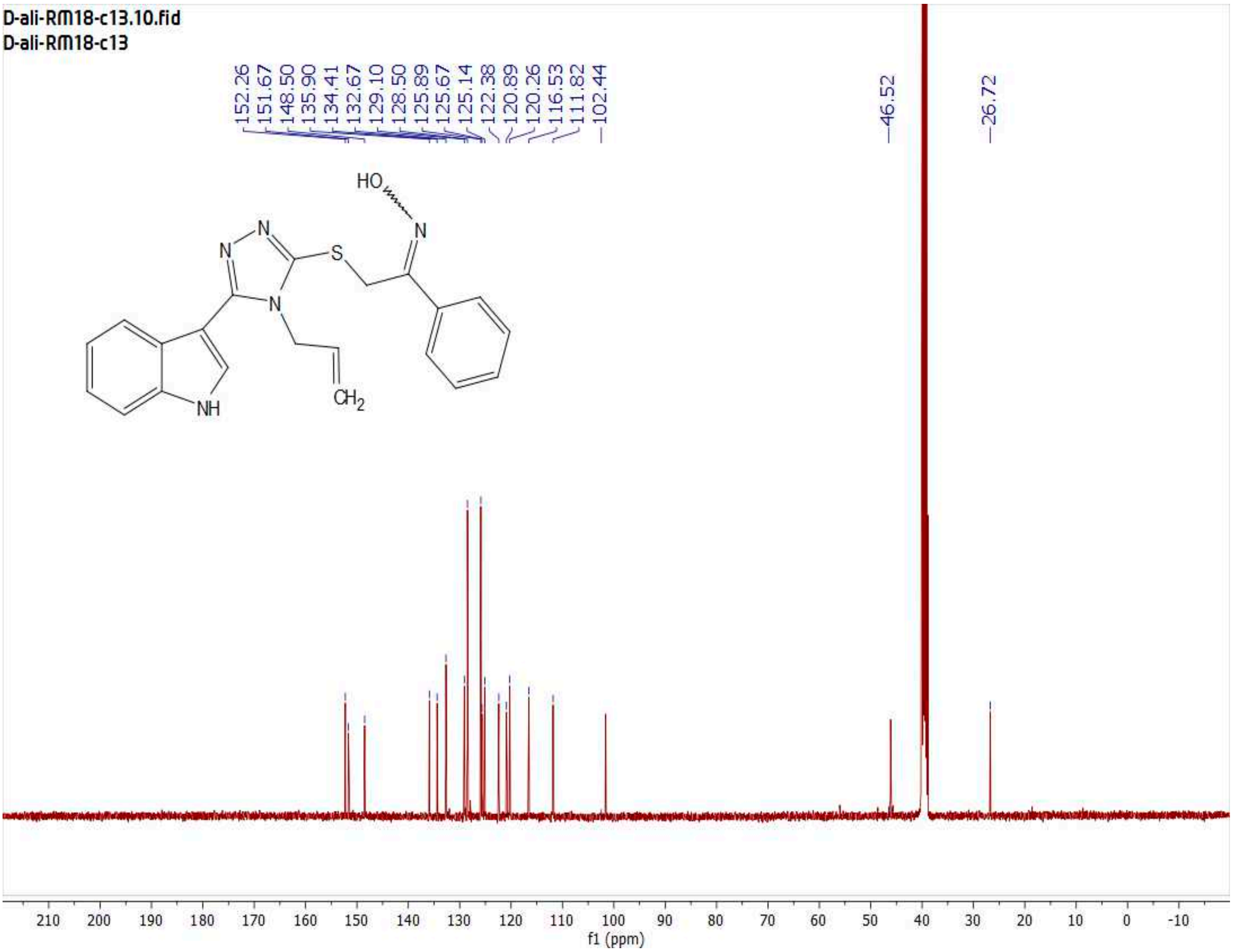

Fig. 25. ^{13}CNMR spectra of compound **7a** (DMSO-d6,101MHZ)

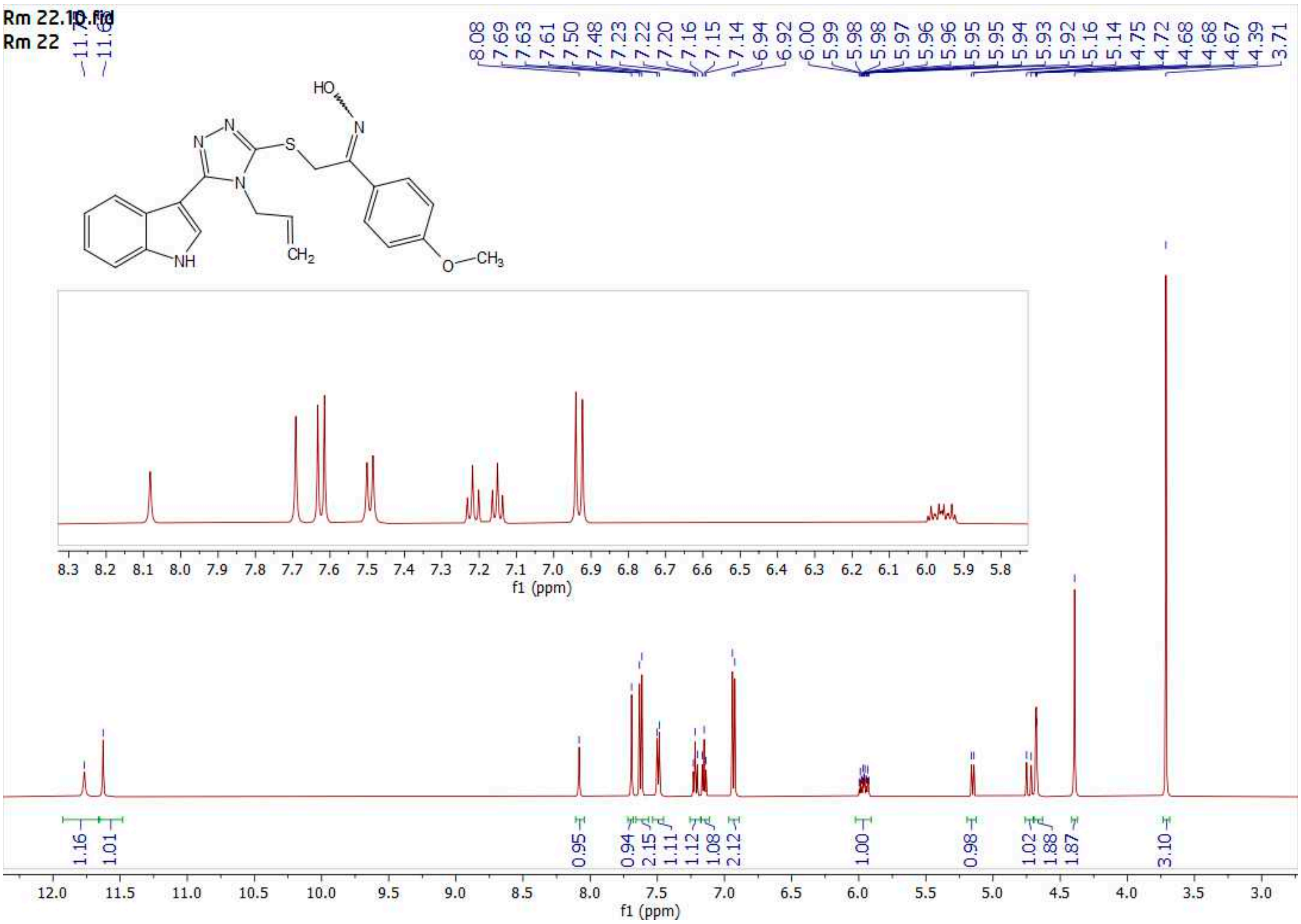

Fig. 26. ^{1}HNMR spectra of compound **7b** (DMSO-d6,300MHZ)

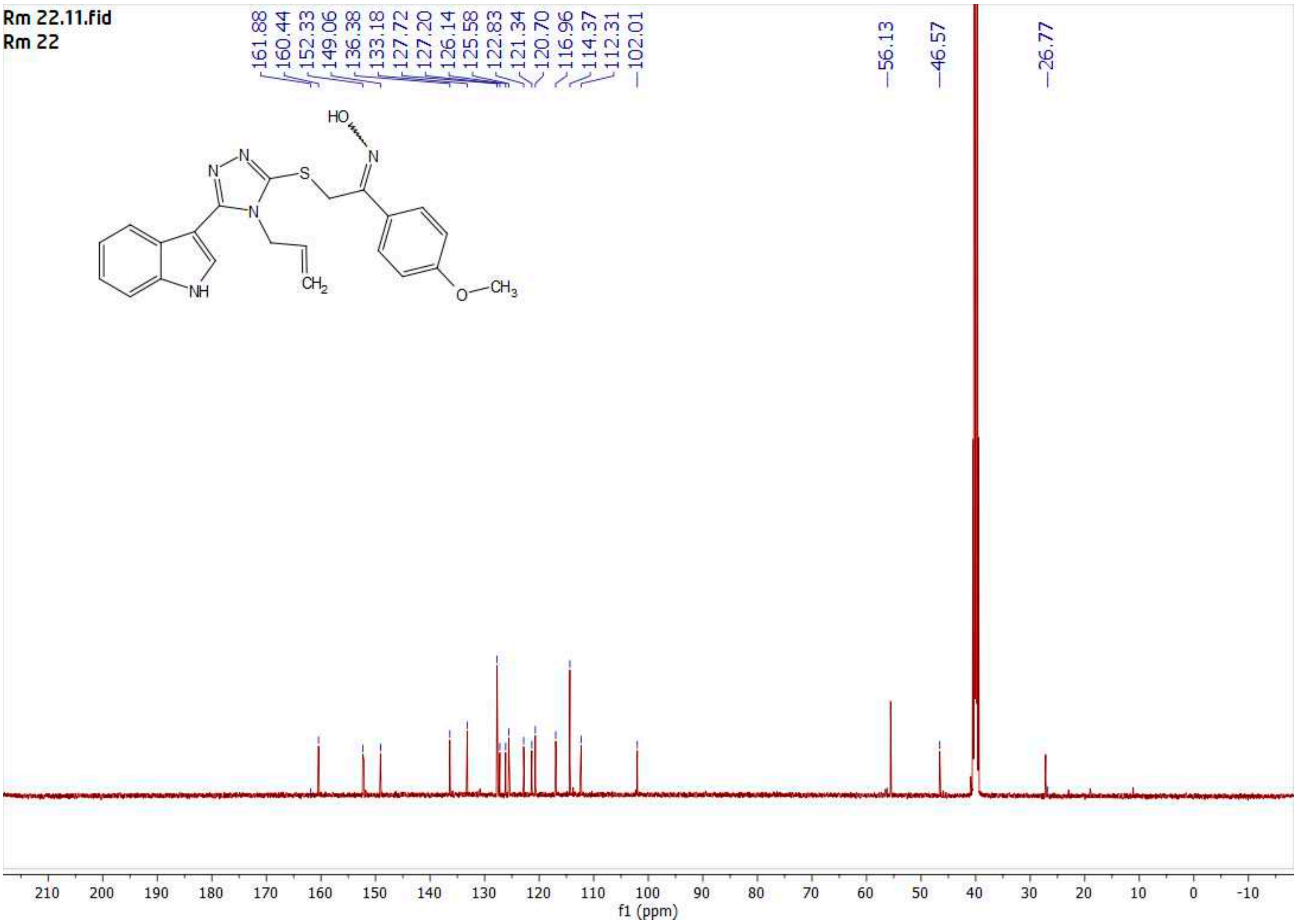

Fig. 27. ^{13}CNMR spectra of compound **7b** (DMSO-d_6,126MHZ)

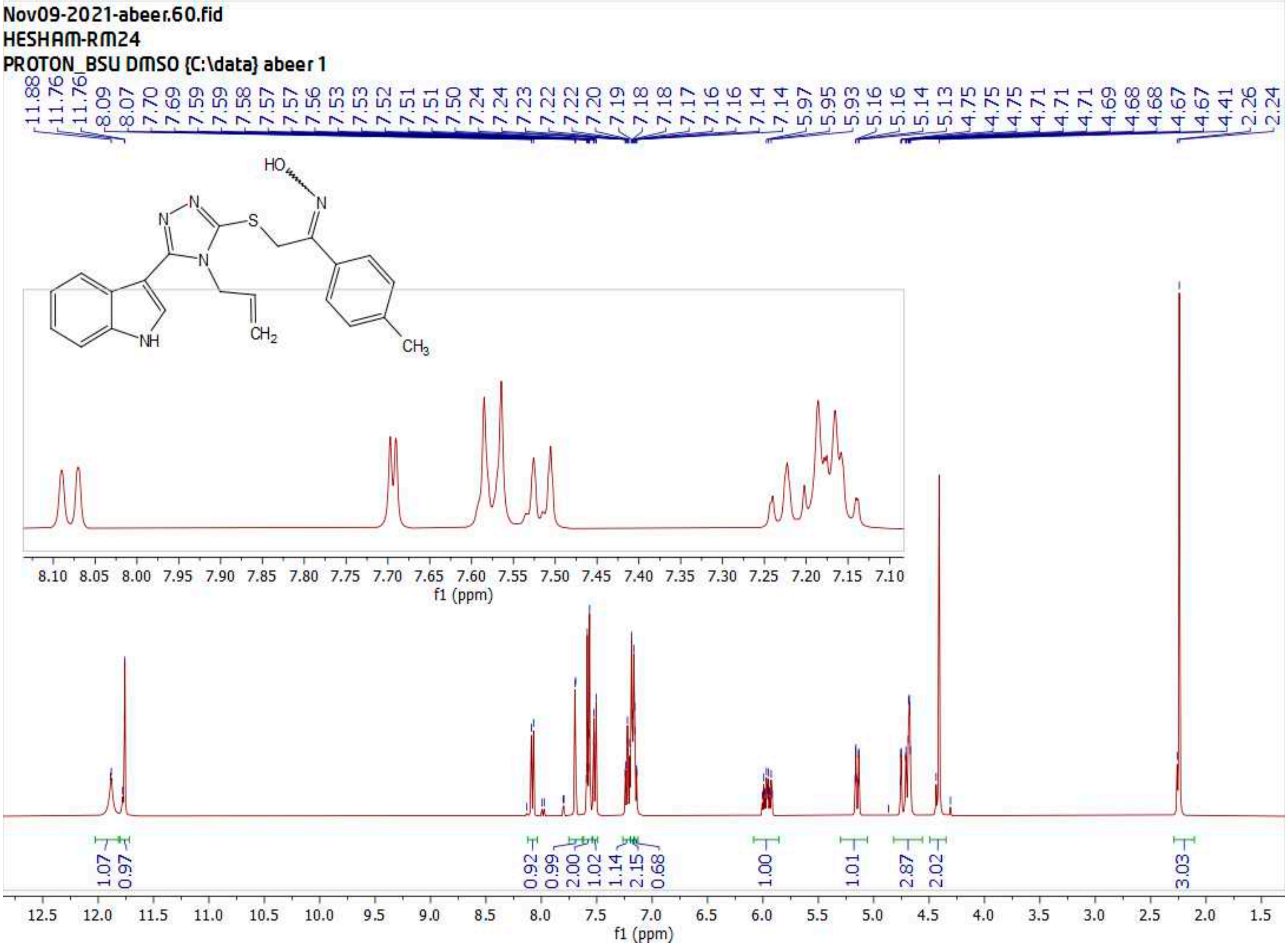

Fig.28. ^{1}HNMR spectra of compound **7c** (DMSO-d$_6$,400MHZ)

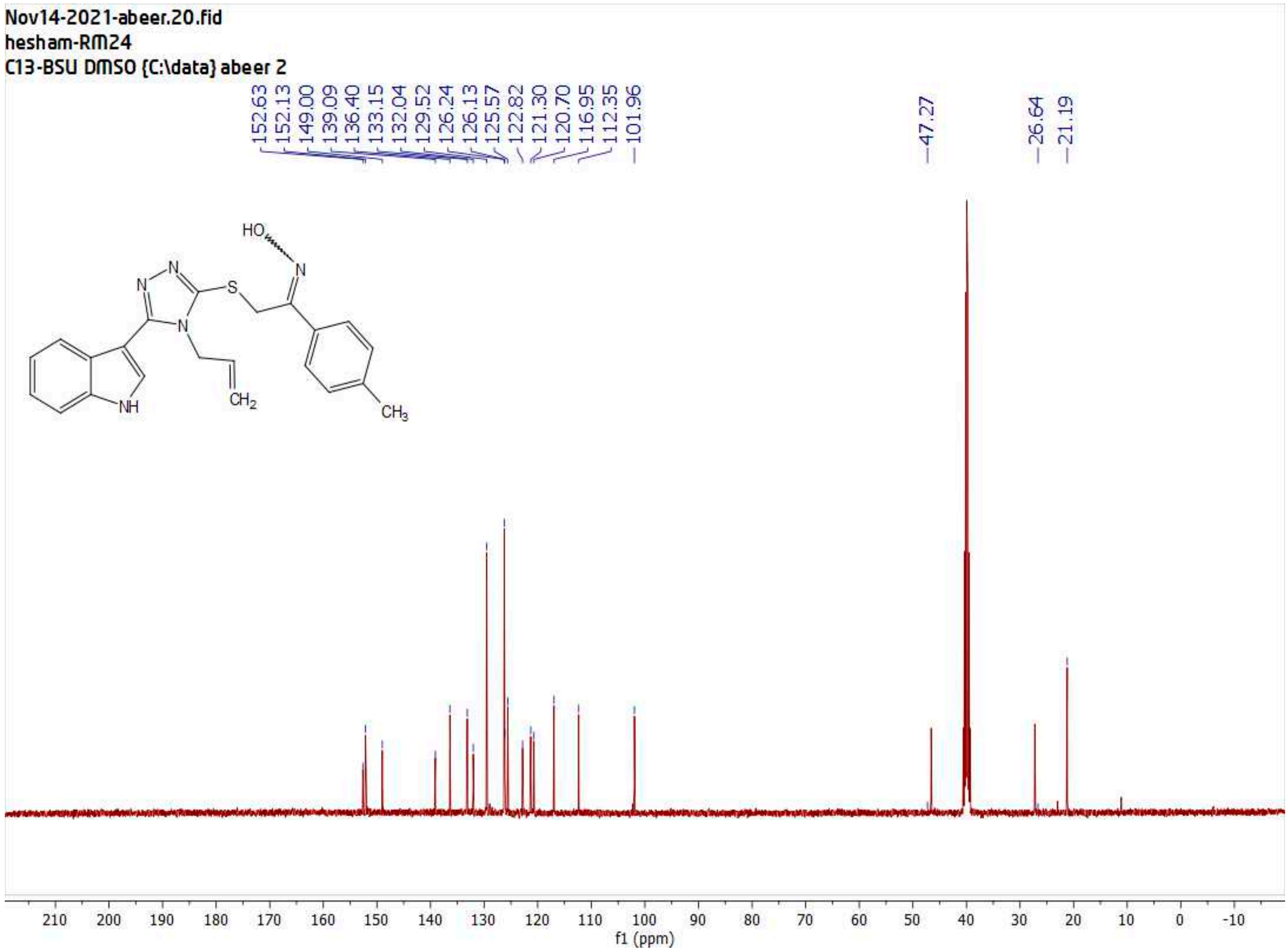

Fig. 29. ^{13}CNMR spectra of compound **7c** (DMSO-d_6,101MHZ)

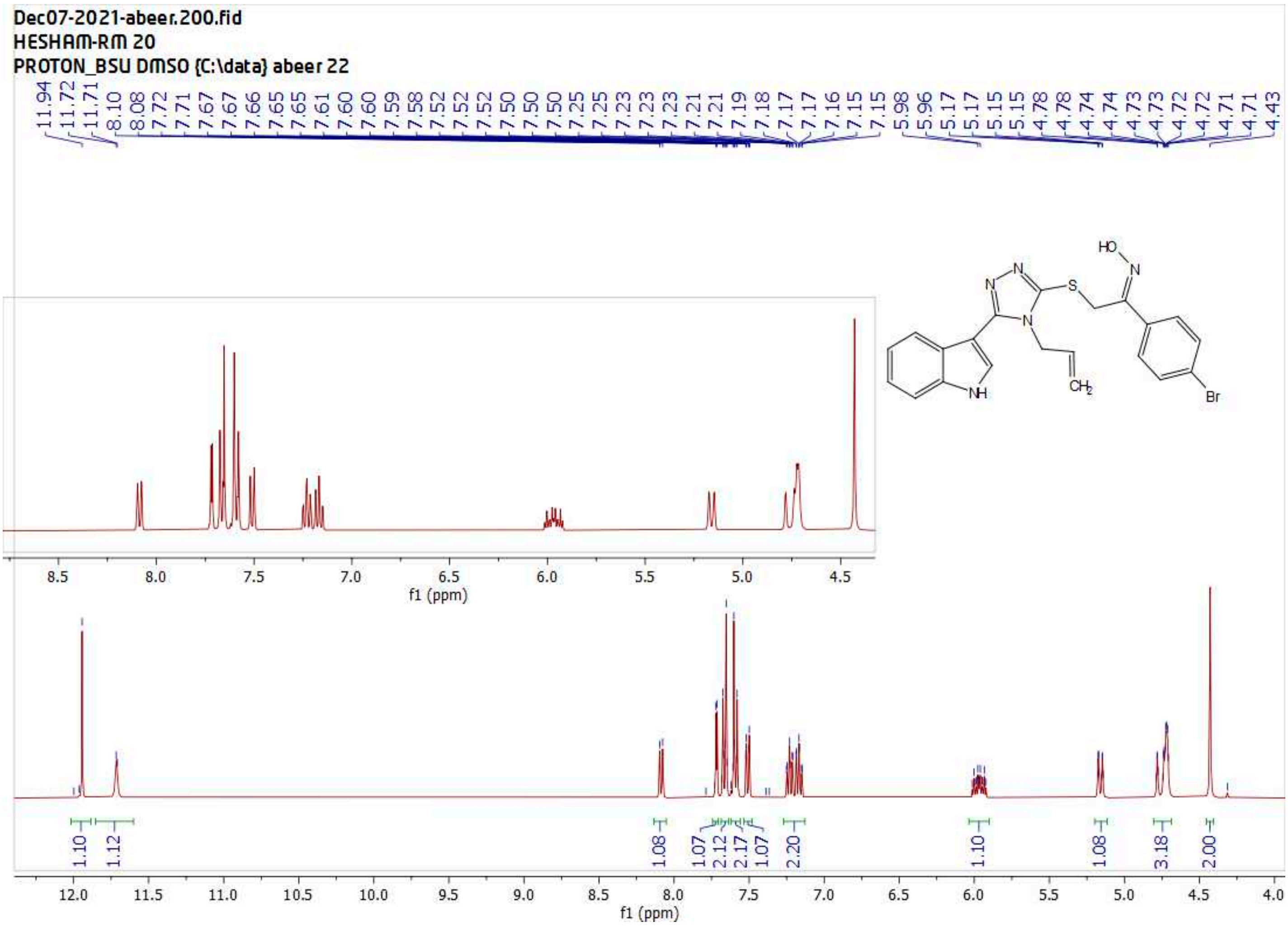

Fig. 30 ^{1}HNMR spectra of compound **7d** (DMSO-d_6,400MHZ)

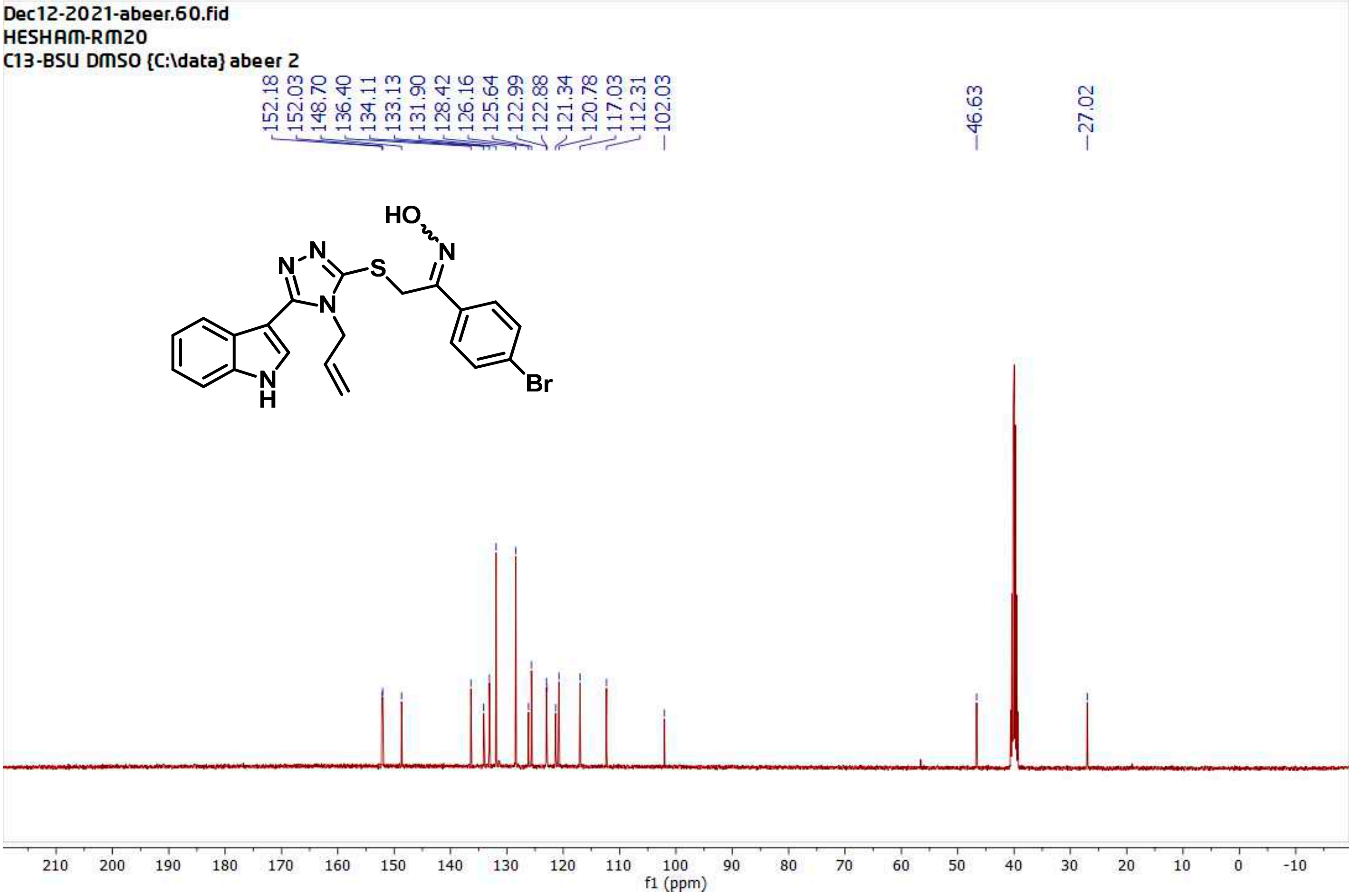

Fig. 31. ^{13}CNMR spectra of compound **7d** (DMSO-d_6,101MHZ)

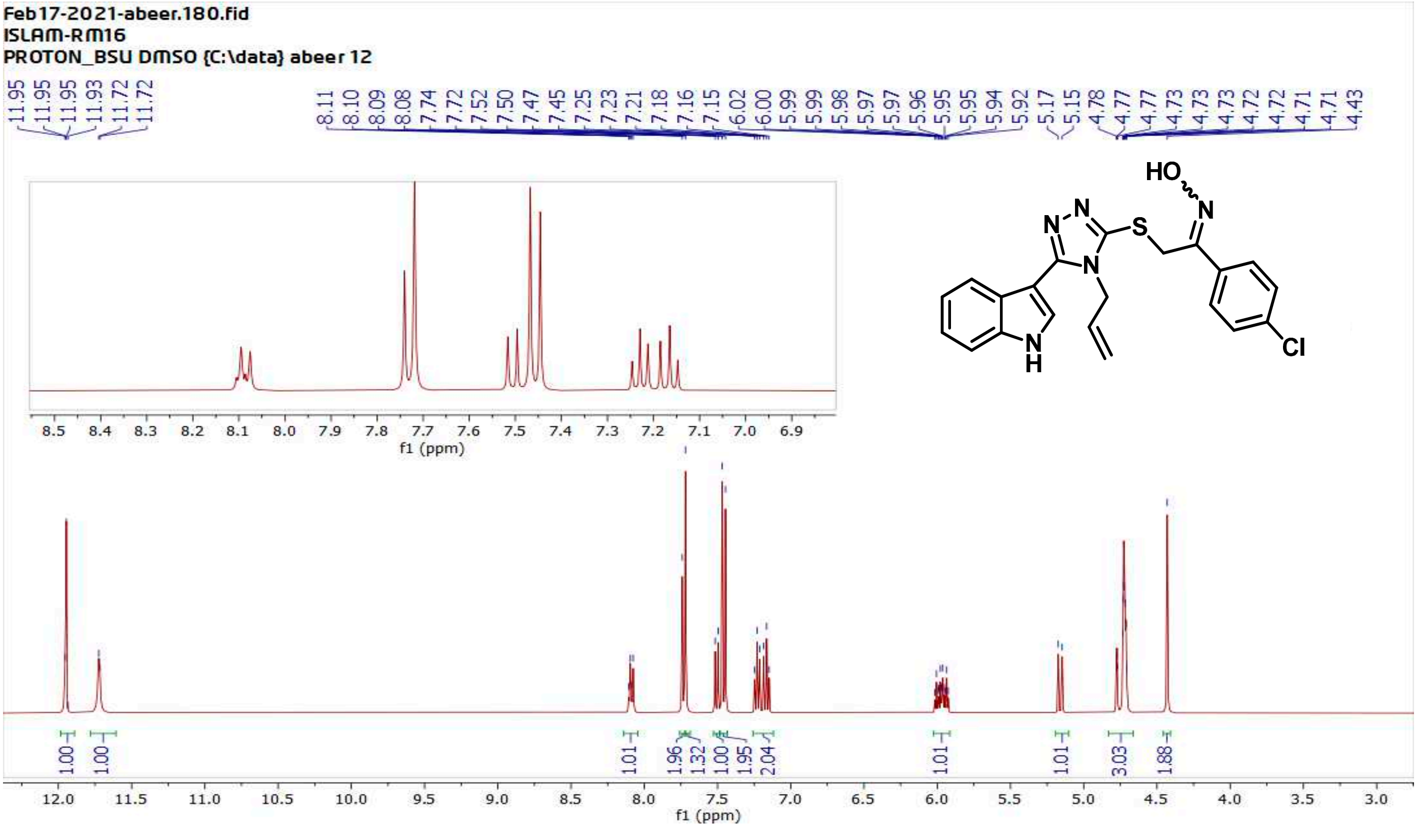

Fig. 32. ^{1}HNMR spectra of compound **7e** (DMSO-d_6,400MHZ)

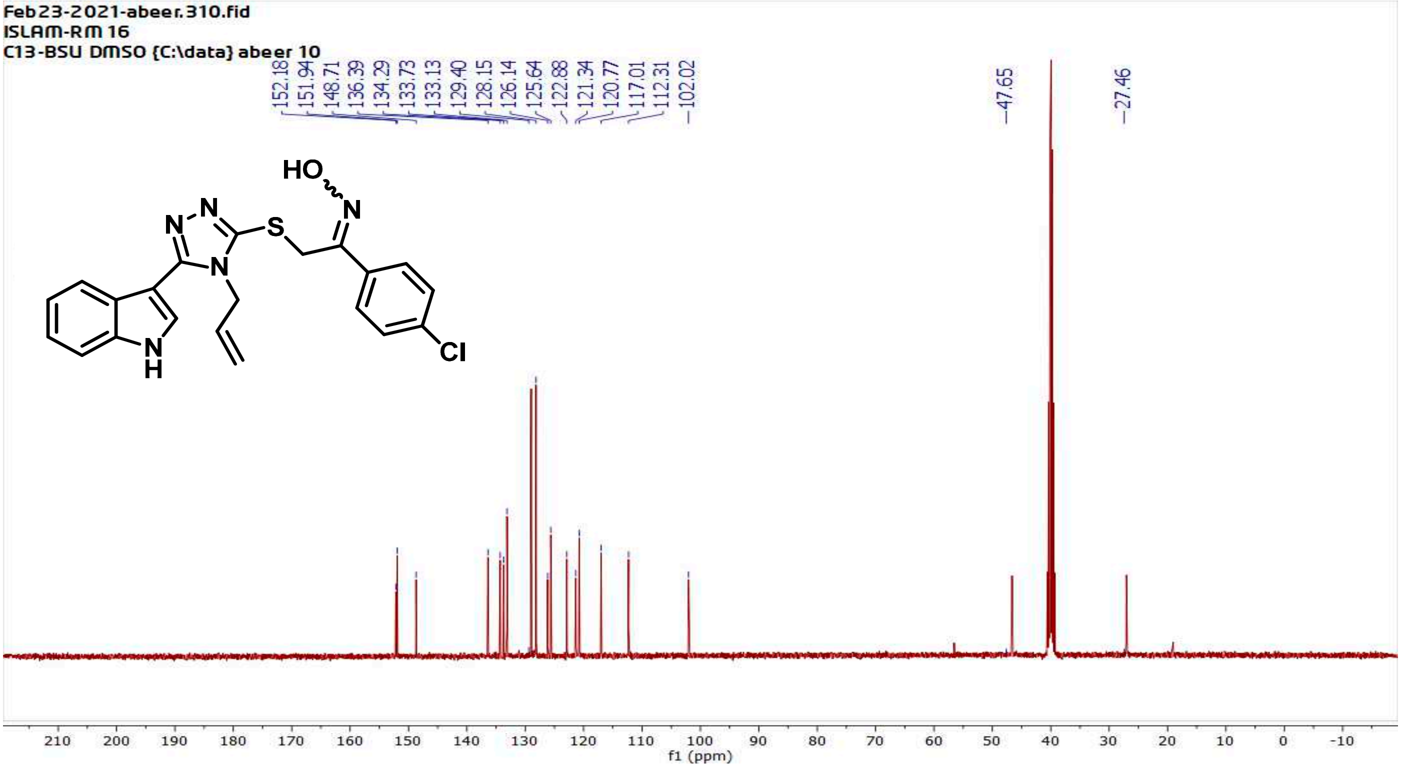

Fig. 33. ^{13}CNMR spectra of compound **7e** (DMSO-d6,101MHZ)

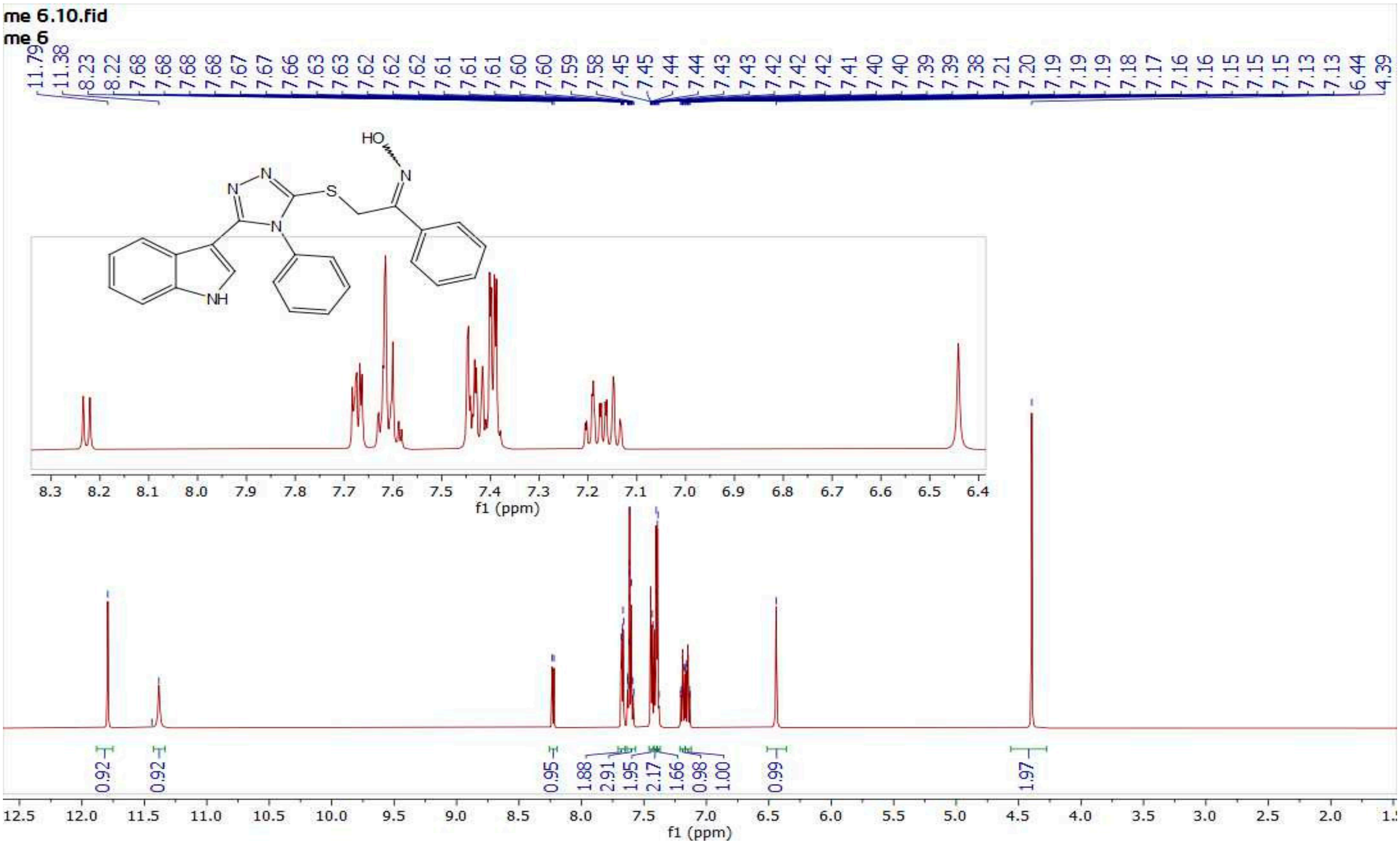

Fig.34. ^{1}HNMR spectra of compound **7f** (DMSO-d6,500MHZ)

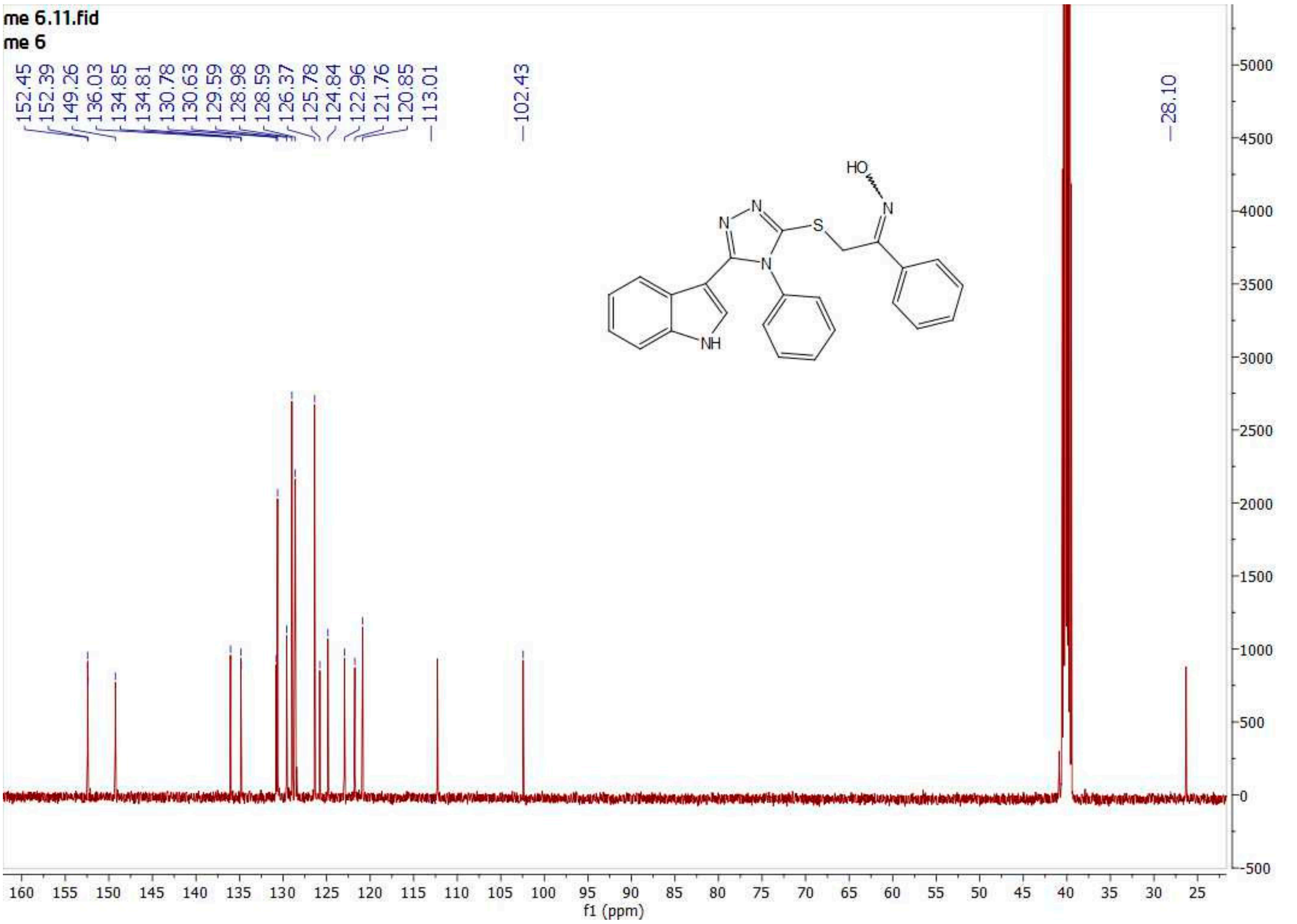

Fig.35. ^{13}CNMR spectra of compound **7f** (DMSO-d6,126MHZ)

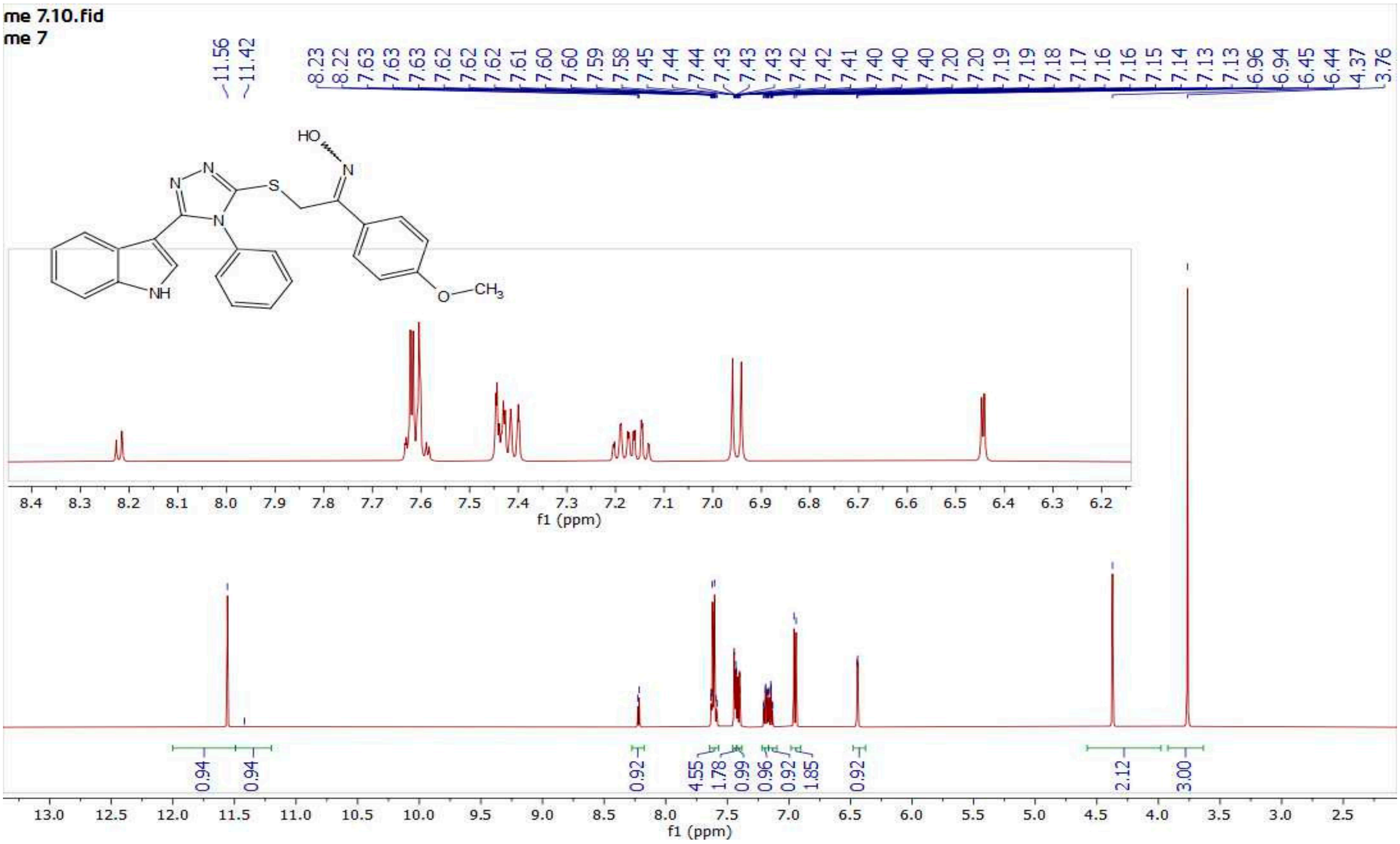

Fig. 36. ^{1}HNMR spectra of compound **7g** (DMSO-d6,400MHZ)

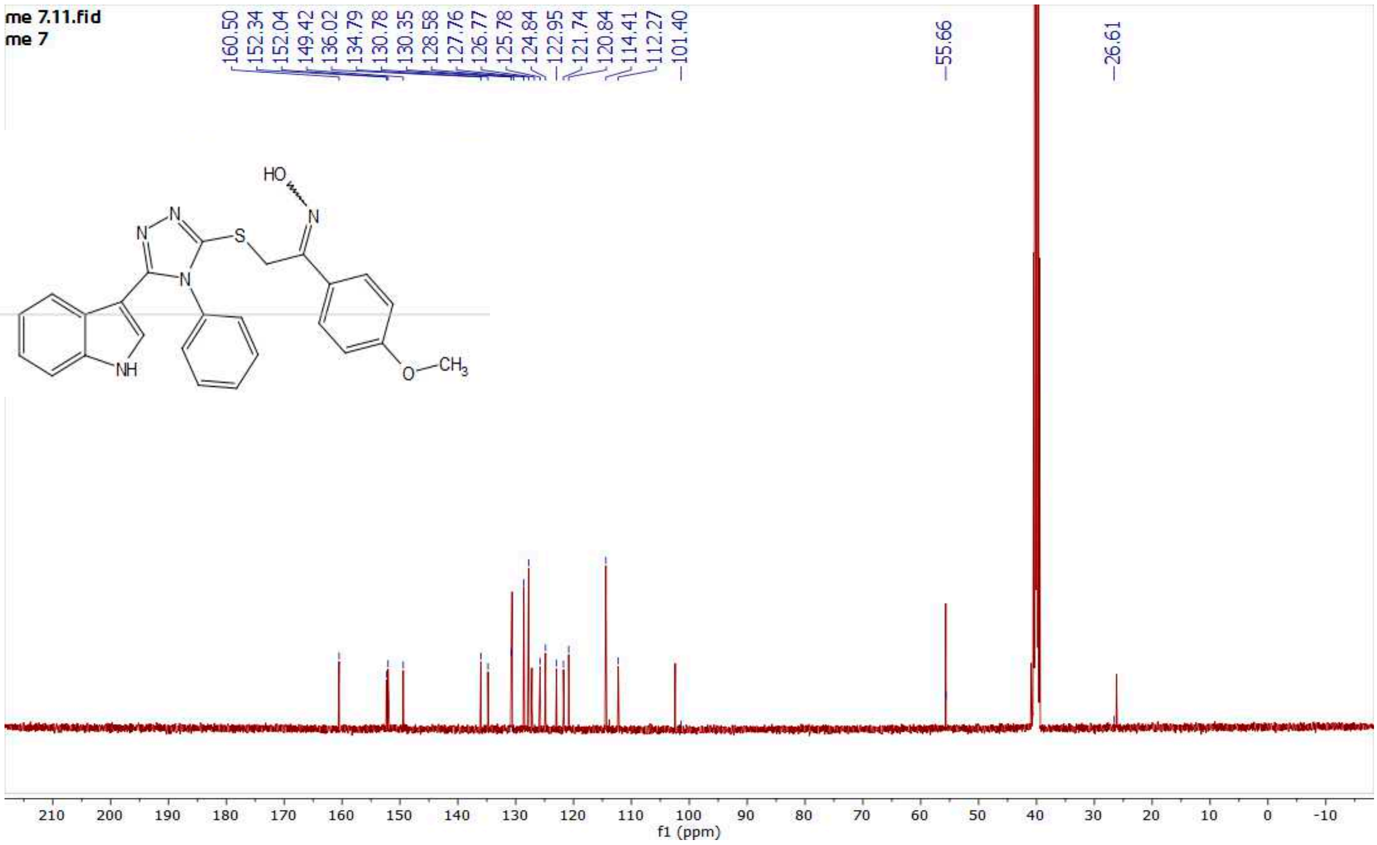

Fig. 37. ^{13}CNMR spectra of compound **7g** (DMSO-d$_6$,126MHZ)

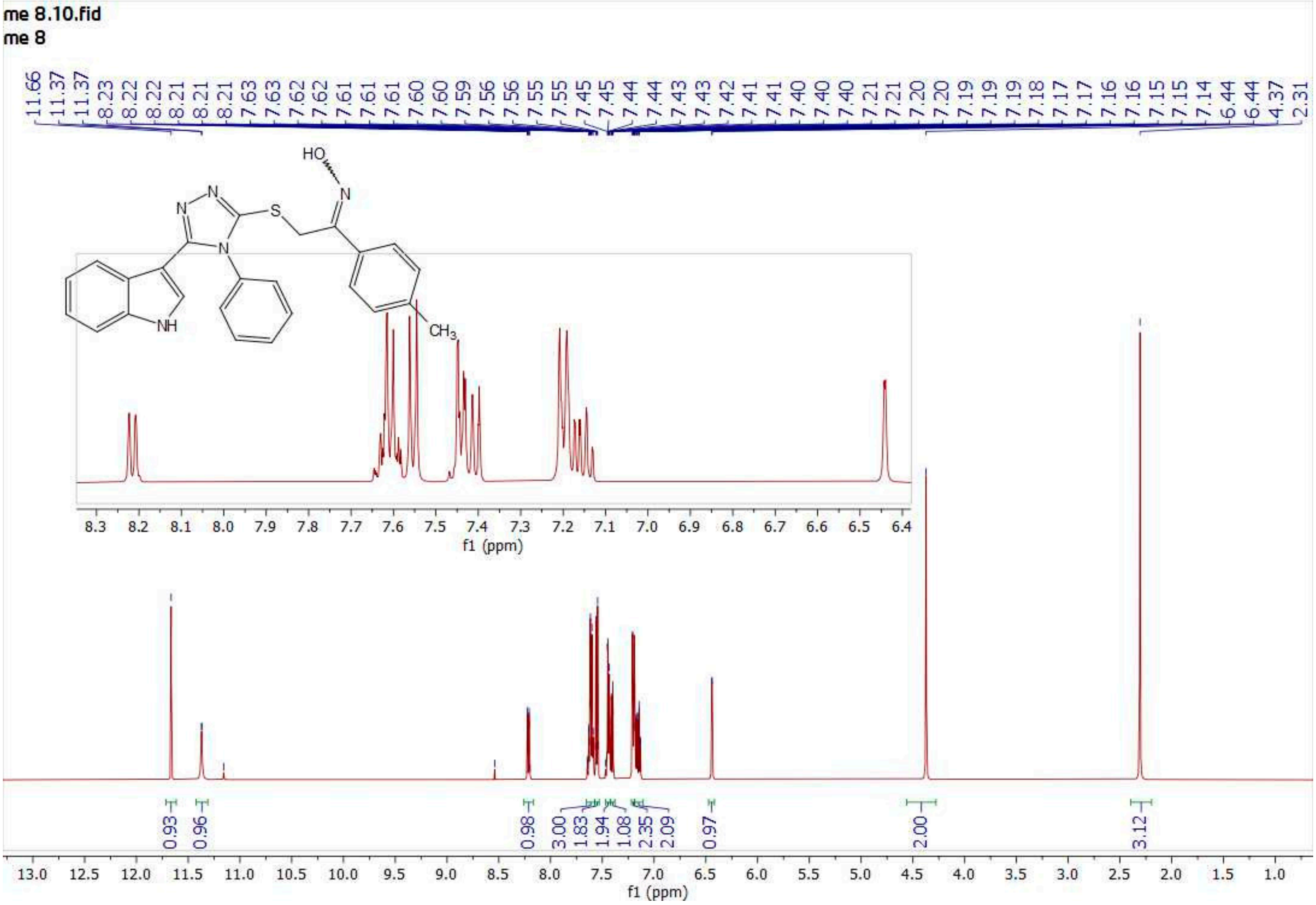

Fig. 38. ^{1}HNMR spectra of compound **7h** (DMSO-d_6,400MHZ)

-

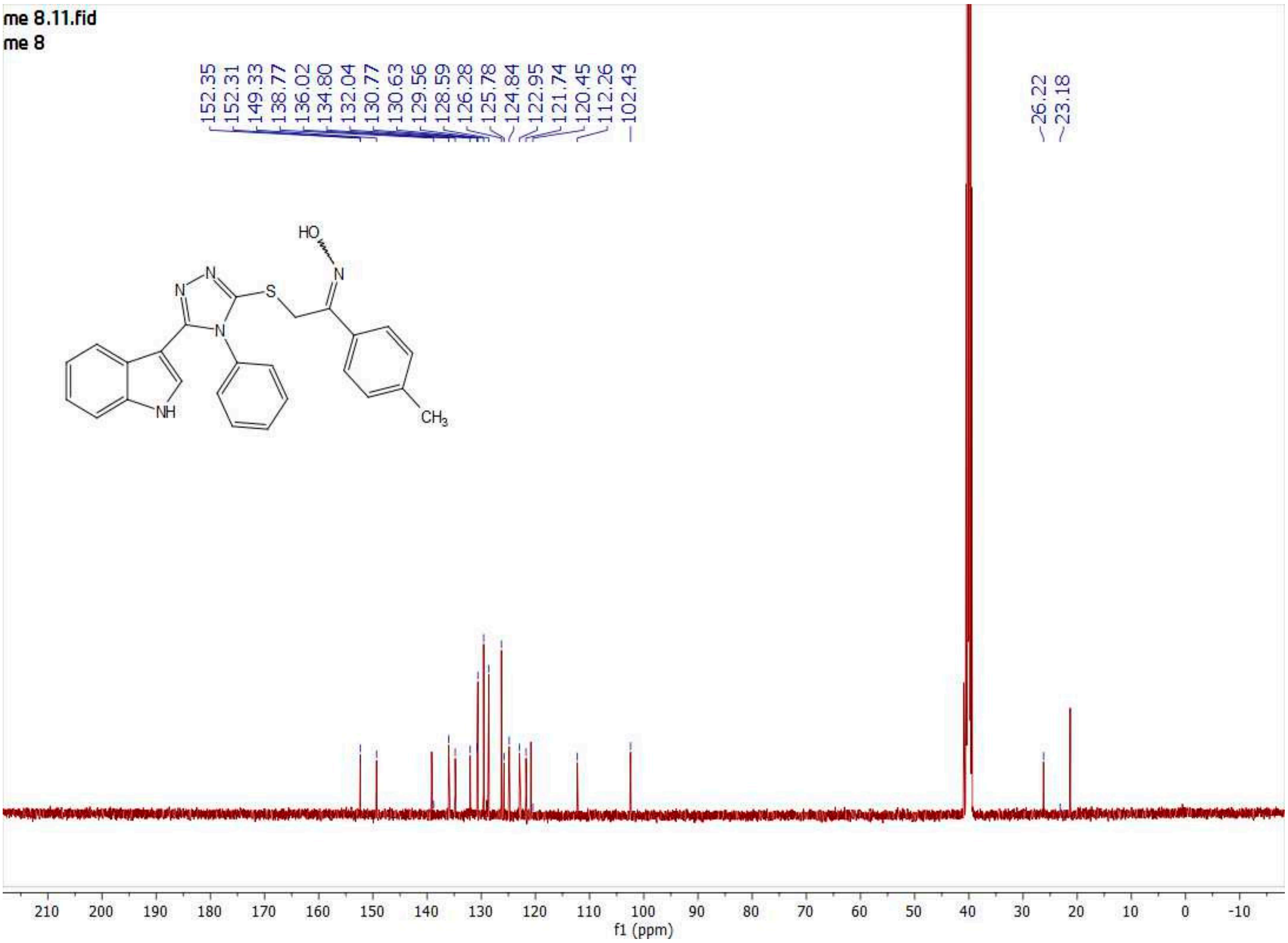

Fig.39. ^{13}CNMR spectra of compound **7h** (DMSO-d6,126MHZ)

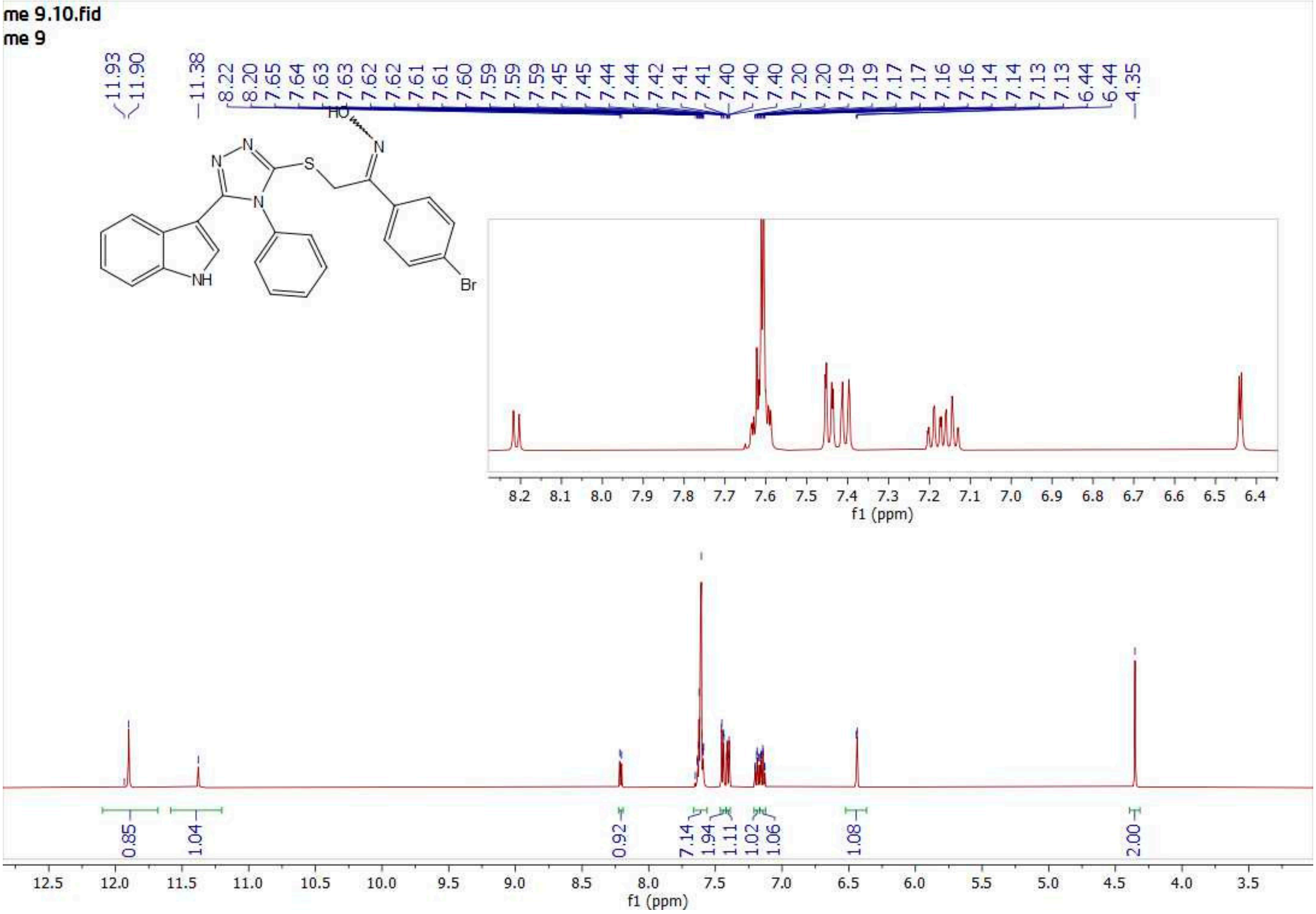

Fig. 40. ^{1}HNMR spectra of compound **7i** (DMSO-d6,400MHZ)

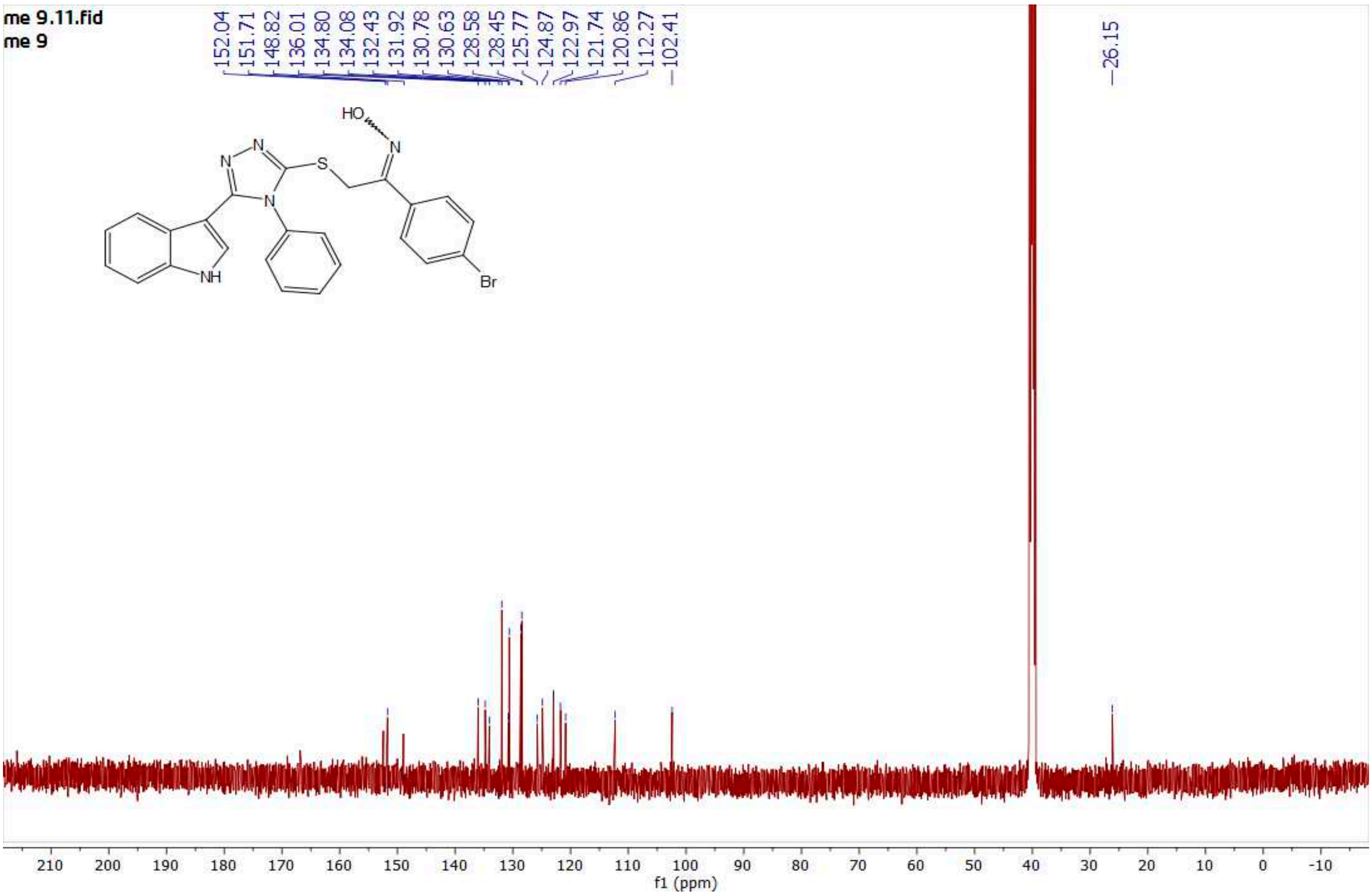

Fig. 41. ^{13}CNMR spectra of compound **7i** (DMSO-d6,126MHZ)

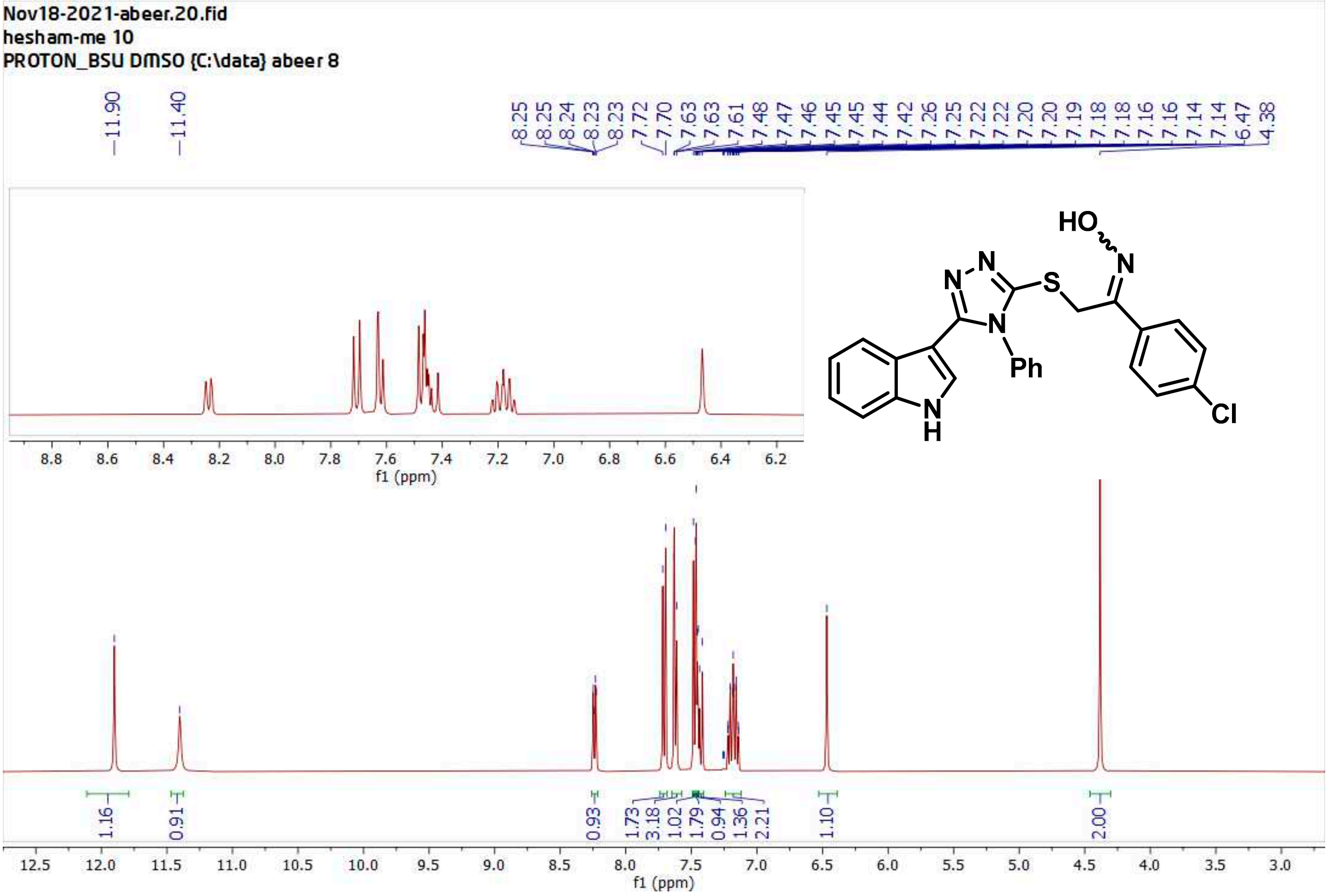

Fig. 42. ^{1}HNMR spectra of compound **7j** (DMSO-d_6,400MHZ)

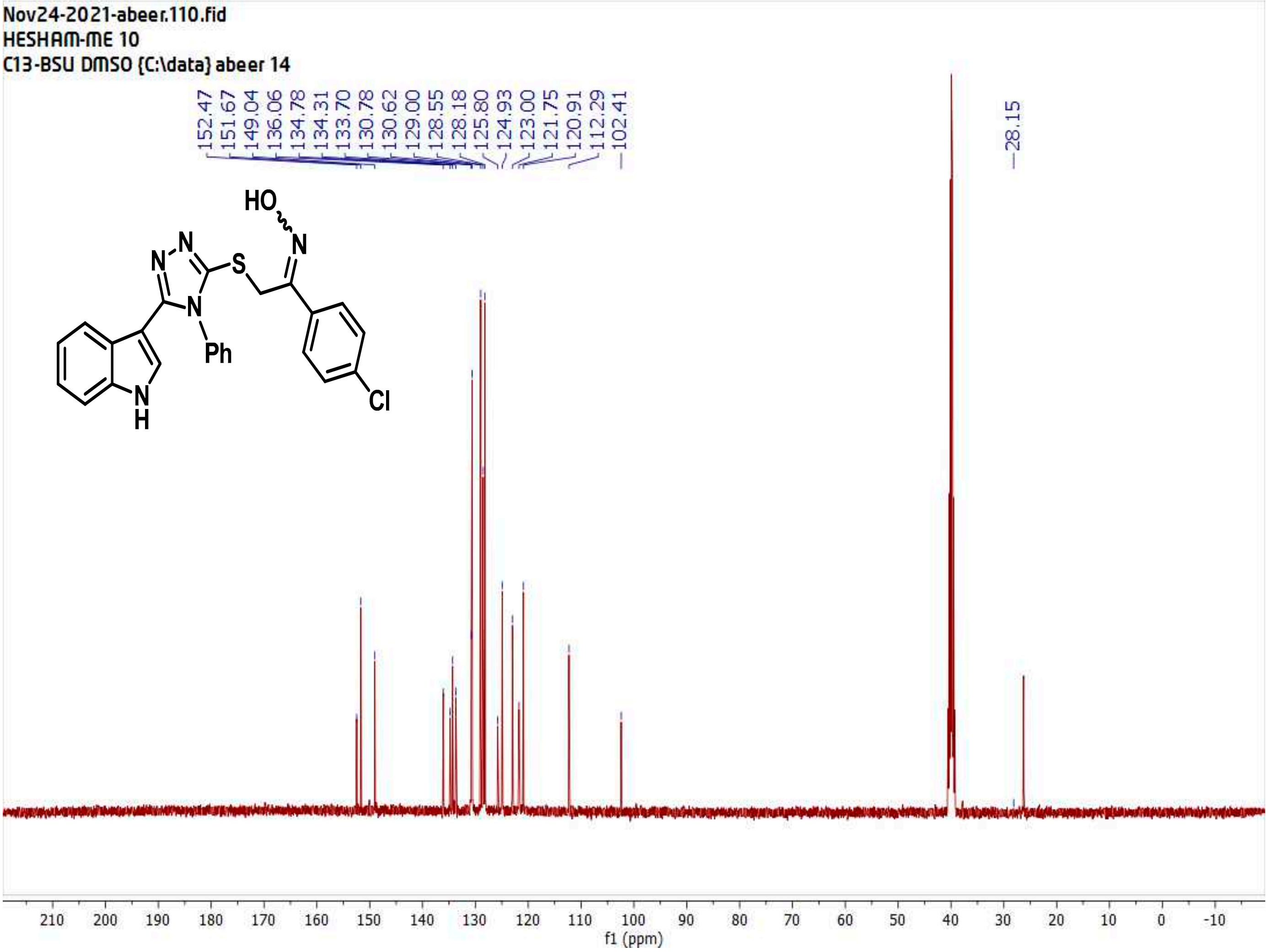

Fig. 43. ^{13}CNMR spectra of compound **7j** (DMSO-d$_6$,101MHZ

Al-Azhar University
The Regional Center for Mycology and Biotechnology

Requester Data:

Name: Dr. Israa Mahmoud
Authority: Faculty of Pharmacy, Minia University

Sample Data:

Twenty samples had been submitted for elemental analysis.

Analysis Report:

Sample Code	C%	H%	N%	S%
Rm 1	68.02	5.12	13.01	5.91
Rm 2	65.31	4.50	12.90	5.82
Rm 6	66.49	5.21	12.34	5.67
Rm 7	65.23	5.19	11.72	5.40
Rm 8	60.37	4.29	11.96	5.42
Rm 9	64.05	4.37	15.12	5.85
Rm 10	63.98	4.41	15.09	5.72
Rm 11	64.91	4.50	12.91	5.86
Rm 12	70.41	5.29	13.05	5.94
(6e) Rm 15	61.90	4.37	13.94	7.95
(7e) Rm 16	59.72	4.40	16.78	7.67
(6a) Rm 17	59.31	4.39	16.80	7.64
(7a) Rm 18	64.52	5.07	18.15	8.40
(6d) Rm 19	55.89	3.91	12.50	7.23
(7d) Rm 20	54.03	4.03	15.17	6.98
(6b) Rm 21	65.49	5.12	14.09	8.01
(7b) Rm 22	63.18	5.14	16.88	7.69
(6c) Rm 23	68.31	5.42	14.67	8.34
(7c) Rm 24	65.32	5.43	17.52	8.09
Rm 25	69.18	4.97	13.71	6.23

INVESTIGATOR M. Elnassag

DIRECTOR

جامعة الأزهر
المركز الإقليمي للفطريات وتطبيقاتها

Al-Azhar University Campus - Nasr City, Cairo, Egypt.
Tel: 0202 22620373 Fax : 0202 22620373
E.mail:rcmb@azhar.edu.eg
Website: http://www.azhar.edu.eg.htm * http://www.azhar.edu.eg/pages/fungi_center.htm
Facebook : RCMB AZHAR P.O. box mail : 11751 Nasr City Cairo, Egypt.

Al-Azhar University
The Regional Center for Mycology and Biotechnology

Requester Data:

Name: Dr. Israa Mahmoud
Authority: Faculty of Pharmacy, Minia University

Sample Data:

Nineteen samples had been submitted for elemental analysis.

Analysis Report:

	Sample Code	C%	H%	N%	S%
6f	Me 1	70.43	4.56	13.91	7.89
6g	Me 2	68.40	4.67	12.98	7.40
6h	Me 3	70.98	4.89	13.47	7.62
6i	Me 4	59.12	3.66	11.72	6.64
6j	Me 5	65.02	4.01	12.80	7.35
7f	Me 6	68.01	4.67	16.72	7.61
7g	Me 7	65.71	4.82	15.54	7.18
7h	Me 8	68.50	4.97	16.17	7.26
7i	Me 9	57.38	3.74	14.12	6.45
6j	Me 10	62.51	4.12	15.45	7.08
	Me 11	71.89	5.23	12.23	5.60
	Me 12	62.70	4.05	11.29	5.17
	Me 13	68.45	4.81	11.58	5.36
	Me 14	67.18	5.01	11.09	5.03
	Me 15	67.40	4.23	12.08	5.60
	Me 16	69.95	4.76	12.19	5.56
	Me 17	71.54	4.89	12.47	5.70
	Me 18	67.41	4.23	12.09	5.62
	Me 19	65.78	4.20	14.17	5.43

INVESTIGATOR **DIRECTOR**

Al-Azhar University Campus - Nasr City, Cairo, Egypt.
Tel: 0202 22620373 Fax : 0202 22620373
E.mail:rcmb@azhar.edu.eg
Website: http://www.azhar.edu.eg.htm * http://www.azhar.edu.eg/pages/fungi_center.htm
Facebook : RCMB AZHAR P.O. box mail : 11751 Nasr City Cairo, Egypt.

Table 1. Cell growth inhibition % from the NCI's *in vitro* human tumor cell lines screen for compounds **6a-e and 7a-e**.

**Subpanel cancer cell Lines**	**Compound**									
	**6a**	**6b**	**6c**	**6d**	**6e**	**7a**	**7b**	**7c**	**7d**	**7e**
**Leukemia**										
**CCRF-CEM**	8.76	8.66	9.66	2.08	2.28	0.66	8.83	2.91	26.74	17.18
**HL-60(TB)**	4.39	8.98	10.37	7.71	10.82	9.34	16.06	10	35.68	35
**K-562**	7.37	8.14	12.77	14.46	10.07	3.29	13.64	13.33	40.78	39.34
**MOLT-4**	7.17	6.64	9.13	8.82	10.63	2.51	8.18	20.4	43.74	40.02
**RPMI-8226**	13.38	16.75	13.83	1.17	7.71	0.38	14.9	8.76	37.89	29.73
**SR**	23.5	18.5	8.97	11.29	12.22	-10.27	3.76	16.5	28.47	12.13
**Non-small cell lung cancer**										
**A549/ATCC**	-0.25	-8.47	5.97	-3.29	3.63	-6.67	-9.29	4.9	9.17	6.38
**EKVX**	4.22	3.03	8.94	7.22	8.21	5.6	7.49	17.74	25.75	27.1
**HOP-62**	7.46	6.07	9.23	-2.78	4.08	9.66	2	8.72	0.14	7.33
**HOP-92**	18.68	17.29	49.73	8.6	21	5.05	8.91	7.98	20.99	26.49
**NCI-H226**	12.2	7.5	17.09	9.93	10.35	12.11	14.91	23.06	33.6	30.41
**NCI-H23**	0.39	5.52	-5.98	4.98	-7.09	-1.28	4.86	9.29	17.87	17.45
**NCI-H322M**	-1.89	2.42	0.83	0.55	-2.47	-0.17	-1.52	5.67	16.26	11.88
**NCI-H460**	-6.84	-1.3	1.59	-6.13	-3.47	3.34	5.34	9.2	19.74	24.71
**NCI-H522**	2.25	0.49	4.55	3.7	7.55	4.48	8.33	14.6	18.46	19.46
**Colon cancer**										
**COLO 205**	-22.1	2.83	-25.85	-7.53	-11.05	2.04	-11.92	-6.48	8.25	0.1
**HCC-2998**	-10.61	-3.48	-7.01	-4.47	-15.55	0.84	-8.35	1.5	-3.65	8.7
**HCT-116**	2.55	5.43	9.79	6.53	6.8	9.24	23.56	22.3	47.02	36.08
**HCT-15**	4	2.44	6.36	0.98	6.28	4.41	4.34	11.29	26.89	21.71

**HT29**	-11.8	-12.69	-6.98	-4.18	-7.4	-3.61	-5.85	-4.09	3.35	5.77
**KM12**	-11.81	-4.63	-6.02	-2.46	-3.89	-2.55	-5.28	0.27	8.58	4.48
**SW-620**	3.89	0.51	-0.34	-10.16	-2.66	0.27	-2.43	4.06	7.31	9.5
**CNS cancer**										
**SF-268**	-1.91	-8.99	-3.82	-1.95	-4.81	-7.26	-0.98	3.63	4.98	-1.1
**SF-295**	3.79	2.91	3.28	6.78	3.57	12.36	16.65	15.12	32.45	25.04
**SF-539**	2.68	1.18	1.45	8.23	0.21	3.26	4.37	-1.42	14	5.14
**SNB-19**	1.66	-1.42	3.56	-0.26	0.6	6.82	1.65	8.04	8.36	12.64
**SNB-75**	3.62	-14.78	-4.77	-6.14	-0.53	-3.17	-12.95	2.58	6.02	4.75
**U251**	-7.24	2.31	6.75	0.12	3.75	4.62	-5.26	-2.94	5.15	1.78
**Melanoma**										
**MALME-3M**	13.39	5.32	10.49	9.34	9.28	7.6	3.2	9.17	4.84	15.59
**M14**	2.35	-1.55	4.78	2.64	6.76	-13.41	2.83	5.59	19.25	16.02
**MDA-MB-435**	-5.02	-8.45	-2.15	-8.05	-5.05	-5.56	-10.63	-2.07	11.47	10.66
**SK-MEL-2**	-4.98	-33.18	1.54	-15.27	-17.1	-6.77	-7.88	1.01	8.97	3.64
**SK-MEL-28**	-5.22	1.56	-4.91	3.9	-10.06	-4.36	-3.89	-5.62	7.82	0.08
**SK-MEL-5**	3.38	3.34	6.71	4.07	3.94	1.57	10.46	11.05	40.88	27.45
**UACC-257**	2.08	-2.78	6.7	-7.6	2.2	-13.1	-11.52	-6.62	-1.32	6.8
**UACC-62**	18.52	15.64	24.69	16.91	14.68	13.57	20.91	30.95	36.26	35.82
**Ovarian cancer**										
**IGROV1**	11.64	11.05	19.58	13.26	7.38	7.6	17.57	23.25	30.8	29.68
**OVCAR-3**	-14.78	-16.93	-13.1	-10.42	-12.24	-14.95	-9.23	-6.11	-3.34	-7.49
**OVCAR-4**	2.53	-4.81	-10.4	-8.69	-8	-8.19	-1.81	6.11	24.47	19.93
**OVCAR-5**	-3.17	-2.88	-2.57	3.19	-5.38	-3.15	-4.1	1.32	6.82	-2.62
**OVCAR-8**	4.58	4.99	10.37	5.3	9.77	1.16	7.1	8.49	18.3	16.84
**NCI/ADR-RES**	2.62	-3.74	4.65	1.17	-2.31	-6.66	-3.18	1.27	6.71	3.86

**SK-OV-3**	-20.45	-30.48	-22.38	-13.09	-20.55	2.21	1.86	-0.26	3.28	3.52
**Renal cancer**										
**786-0**	-1.07	-1.31	2.26	1.44	3.55	4.91	0.73	-2.2	13.43	11.1
**ACHN**	6.19	8.68	11.03	11.74	7.39	3.82	7.56	11.38	17.87	10.15
**CAKI-1**	16.87	7.83	15.07	9.77	7.04	6.71	1.5	20.2	25.83	25.44
**RXF 393**	-2.2	-5.88	-0.07	-12.81	-9.22	-4.5	-12.09	-5.46	19.28	5.62
**SN12C**	7.27	3.73	5.47	4.39	4.38	2.4	8.24	11.69	16.28	14.94
**TK-10**	-43.87	-40.54	-39.15	-34.3	-24.26	-12.21	-27.16	-26.79	12.91	1.28
**UO-31**	34.27	26.94	29.69	31.6	28.54	20.94	19.23	34.99	38.32	39.53
**Prostate cancer**										
**PC-3**	10.91	20.33	22.23	21.68	22.32	9.77	14.91	7.62	31.62	24.41
**DU-145**	-9.05	-11.8	-8.9	-6.19	-14.45	-5.82	-5.85	-2.38	6.21	5.88
**Breast cancer**										
**MCF7**	2.77	5.93	9.88	2.11	7.08	-2.96	11.31	11.88	17.91	13.33
**MDA-MB-231/ATCC**	11.25	11.05	11.5	3.15	8.19	3.9	13.47	4.01	23.58	23.07
**HS 578 T**	2.96	0.85	-2.83	-5.71	-3.65	5.73	2.61	6.55	22.13	12.69
**BT-549**	-9.39	-1.84	2.35	-5.98	-0.26	-3.51	-3.8	-7.22	20.38	14.42
**T-47D**	5.03	11.14	9.92	2.98	3.25	8.67	26.4	26.49	39.77	46.5
**MDA-MB-468**	12.29	5.01	12.5	13.06	8	15.36	16.32	27.15	42.16	43.14

Table 2. Cell growth inhibition % from the NCI's *in vitro* human tumor cell lines screen for compounds **6f-j and 7f-j**.

Subpanel cancer cell Lines	compound									
	6f	6g	6h	6i	6j	7f	7g	7h	7i	7j
**Leukemia**										
**CCRF-CEM**	8.65	17.49	39.16	13.64	7.26	59.01	83.47	135	106.14	105.19
**HL-60(TB)**	12.79	18.68	56.65	55.54	11.07	75.34	96.13	113.75	136.27	125.94
**K-562**	47.22	41.78	72.84	18	31.01	67.56	81.25	98.41	111.09	93.07
**MOLT-4**	9.43	17.9	38.26	7.77	-3.56	76.13	83.29	127.84	119.16	107.37
**RPMI-8226**	18.68	27.33	41.06	45.62	16.83	68.09	90.19	119.08	113.13	113.41
**SR**	18.44	20.3	46.96	9.15	7.63	43.77	89.11	90.21	98.71	87.18
**Non-small cell lung cancer**										
**A549/ATCC**	14.5	26	46.73	70.12	62.45	46.89	65.83	81.56	118.21	78.52
**EKVX**	31.6	70.74	46.98	89.21	74.37	73.59	84.89	71.75	163.3	97.95
**HOP-62**	76.56	91.18	92.2	26.36	52.95	15.41	55.03	156.56	154.84	164.1
**HOP-92**	52.98	92.1	107.56	71.88	34.95	82.67	71.81	105.5	111.15	108.42
**NCI-H226**	95.52	99.94	141.33	60.88	82.39	56.73	81.8	141.06	93.7	85.66
**NCI-H23**	55.09	43.51	82.67	46.04	59.22	45.84	58.36	46.7	85.88	52.69
**NCI-H322M**	18.79	41.65	25.12	54.27	41.32	35.05	63.64	142.43	172.69	149.35
**NCI-H460**	38.2	34.49	70.73	65.5	39.91	66.02	82.75	98.01	121.92	89.51
**NCI-H522**	28.4	54.27	98.57	53.73	33.02	46.24	63.46	76.24	129.74	87.63
**Colon cancer**										
**COLO 205**	6.63	15.21	24.98	86.54	57.41	47.09	63.66	87.63	141.19	89.79
**HCC-2998**	16.91	22.24	33.62	68.32	21.87	43.28	57.58	141.9	166.48	83.03
**HCT-116**	61.96	47.51	92.32	45.72	54.25	59.99	71.92	97.38	136.26	99.26
**HCT-15**	20.55	26.72	56.21	56	26.69	74.77	84.65	162.39	192.84	148.11
**HT29**	52.07	16.91	92.87	42.58	57.44	50.7	68.04	93.65	176.88	95.53
**KM12**	30.54	11.86	48.96	65.37	21.27	59.86	78.78	49.84	97.89	78.61
**SW-620**	23.91	13.73	80.99	20.18	29.68	47.16	60.56	180.39	186.34	137
**CNS cancer**										
**SF-268**	68.28	58.96	70.26	50.85	30.63	23.07	51.45	160.1	155.61	149.04
**SF-295**	28.01	63.76	88.94	66.29	44.97	60.83	79	149.9	114.97	85.93
**SF-539**	92.41	71.5	77.87	37.98	50.52	22.95	42.73	89.94	164.9	70.52
**SNB-19**	80.81	70.24	80.34	29.83	59.98	48.51	59.71	118.53	109.28	82.56
**SNB-75**	-0.34	84.96	101.21	61.18	45.27	23.76	36.74	144.17	119.93	51.98
**U251**	89.22	65.6	90.22	38.43	69.86	46.25	60.59	89.6	90.27	69.48

**Melanoma**										
**LOX IMVI**	70.78	56.49	119.53	58.24	84.12	58.95	178.19	188.21	187.44	189.32
**MALME-3M**	62.6	116.15	64.85	29	36.02	23.91	26.9	83.33	110.21	88.86
**M14**	26.37	24.3	48.85	49.01	19.41	54.16	71.13	164.43	167.68	147.28
**MDA-MB-435**	12.78	30.9	61.42	59.08	20.84	54.89	105.25	200	200	199.76
**SK-MEL-2**	8.14	28	15.3	119.48	66.33	50.76	94.13	37.27	166.28	79.86
**SK-MEL-28**	0.04	50.3	51.05	65.07	31.7	45.51	62.61	37.46	126.35	78.78
**SK-MEL-5**	9.3	18.65	67.12	82.8	21.8	84.13	110.16	65.93	124.46	78.78
**UACC-257**	-3.2	40.54	22.72	47.45	27.7	20.06	49.88	64.28	108.6	55.74
**UACC-62**	26.6	74.17	76.14	64.77	47.2	59.17	68.63	152.56	190.48	143.19
**Ovarian cancer**										
**IGROV1**	44.91	39.53	75.77	35.91	48.56	41.83	56.87	162.41	165.42	134.1
**OVCAR-3**	39.19	46.42	34.8	57.53	26.06	45.03	69.47	85.26	99.3	65.77
**OVCAR-4**	102.47	99.43	115.56	53.81	85.1	62.23	83.17	187.15	183.4	181.32
**OVCAR-5**	11.43	22.56	86.97	41.7	9.28	14.29	44.99	148.92	182.53	143.38
**OVCAR-8**	85.34	84.48	88.71	56.47	75.51	43.18	70.3	127.82	141.65	93.63
**NCI/ADR-RES**	85.22	100	127.71	100	94.15	44.7	61.84	168.42	173.87	170.19
**SK-OV-3**	14.37	33.74	75.97	51.36	27.94	30.61	47.94	88.51	150.17	101.58
**Renal cancer**										
**786-0**	70.39	54.75	135.44	17.63	55.12	17.92	40.27	97.83	65.25	65.86
**A498**	-6.16	-11.29	14.19	63.97	42.74	14.8	30.17	8.13	37.85	16.29
**ACHN**	39.53	77.16	79.61	62.6	42.51	58.43	77.71	161.68	183.52	96.66
**CAKI-1**	46.77	38.51	87.64	55.73	35.54	48.94	79.93	127.37	194.29	110.75
**SN12C**	55.32	55.61	75.12	27.13	35.37	42.65	54.23	136.7	177.16	116.18
**TK-10**	27.99	36.09	102.18	35.12	16.04	33.99	57.35	165.14	182.84	123.17
**UO-31**	44.46	67.39	69.65	73.99	45.22	71.59	81.77	93.55	96.42	65.61
**Prostate cancer**										
**PC-3**	34.24	49.68	56.97	53.79	39.55	75.89	76.89	146.54	164.4	138.01
**DU-145**	14.33	31.12	21.05	41.52	11.57	40.16	57.04	160.24	188.46	130.92
**Breast cancer**										
**MCF7**	63.83	54.36	87.52	46.63	61.6	58.93	85.55	154.96	142.51	96.73
**MDA-MB-231/ATCC**	72.97	54.36	102.39	33.99	48.26	31.27	39.28	182.05	184.18	154.53
**HS 578 T**	75.87	84.49	100.73	47.68	66.79	33.3	61.19	153.52	139.38	131.67
**BT-549**	37.76	75.34	77.64	34.8	30.24	42.51	83.49	175.54	172.67	166.72
**T-47D**	16.46	43.31	53.34	78.02	45.41	80.17	89.65	147.3	94.04	96.09
**MDA-MB-468**	22.8	40.22	52.02	77.96	53.6	64.56	99.41	188.85	194.51	185.12

Panel/Cell Line	Growth Percent
**Leukemia**	
CCRF-CEM	91.24
HL-60(TB)	95.61
K-562	92.63
MOLT-4	92.83
RPMI-8226	86.62
SR	76.50
**Non-Small Cell Lung Cancer**	
A549/ATCC	100.25
EKVX	95.78
HOP-62	92.54
HOP-92	81.32
NCI-H226	87.80
NCI-H23	99.61
NCI-H322M	101.89
NCI-H460	106.84
NCI-H522	97.75
**Colon Cancer**	
COLO 205	122.10
HCC-2998	110.61
HCT-116	97.45
HCT-15	96.00
HT29	111.80
KM12	111.81
SW-620	96.11
**CNS Cancer**	
SF-268	101.91
SF-295	96.21
SF-539	97.32
SNB-19	98.34
SNB-75	96.38
U251	107.24
**Melanoma**	
MALME-3M	86.61
M14	97.65
MDA-MB-435	105.02
SK-MEL-2	104.98
SK-MEL-28	105.22
SK-MEL-5	96.62
UACC-257	97.92
UACC-62	81.48
**Ovarian Cancer**	
IGROV1	88.36
OVCAR-3	114.78
OVCAR-4	97.47
OVCAR-5	103.17
OVCAR-8	95.42
NCI/ADR-RES	97.38
SK-OV-3	120.45
**Renal Cancer**	
786-0	101.07
ACHN	93.81
CAKI-1	83.13
RXF 393	102.20
SN12C	92.73
TK-10	143.87
UO-31	65.73
**Prostate Cancer**	
PC-3	89.09
DU-145	109.05
**Breast Cancer**	
MCF7	97.23
MDA-MB-231/ATCC	88.75
HS 578T	97.04
BT-549	109.39
T-47D	94.97
MDA-MB-468	87.71
Mean	98.12
Delta	32.39
Range	78.14

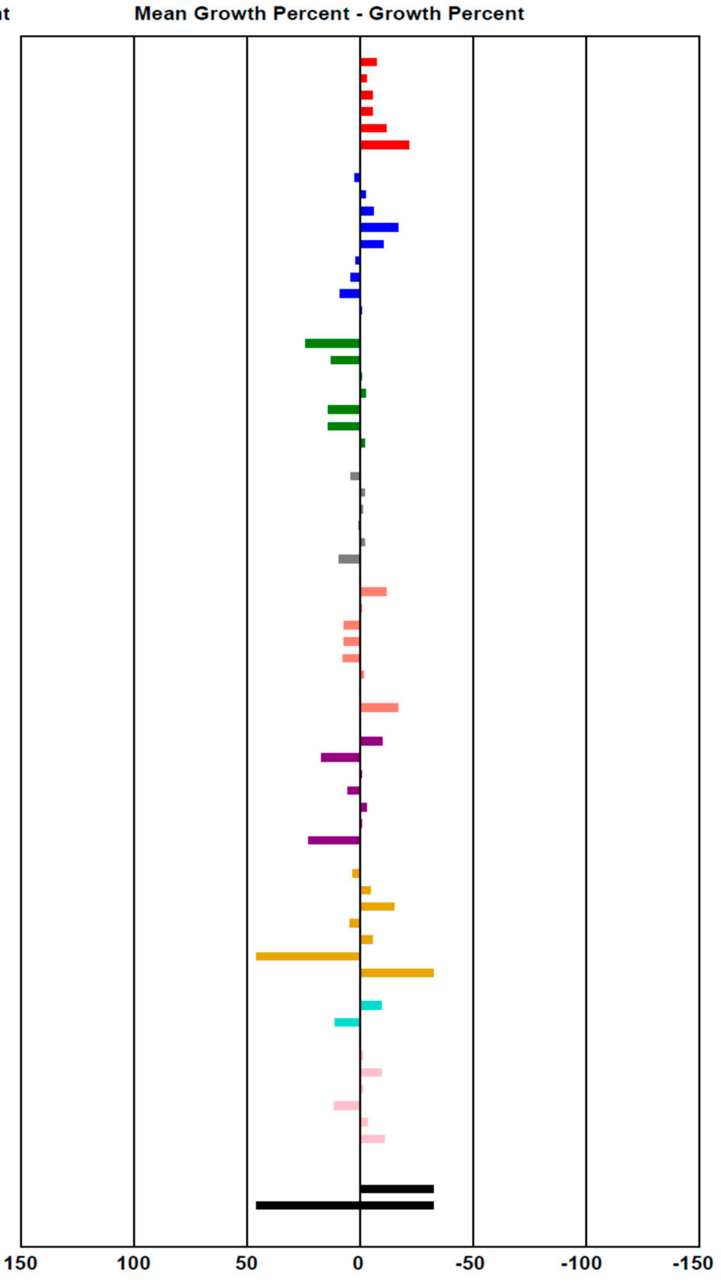

Fig. 44. One-dose growth (%) and mean graph of compound **6a**

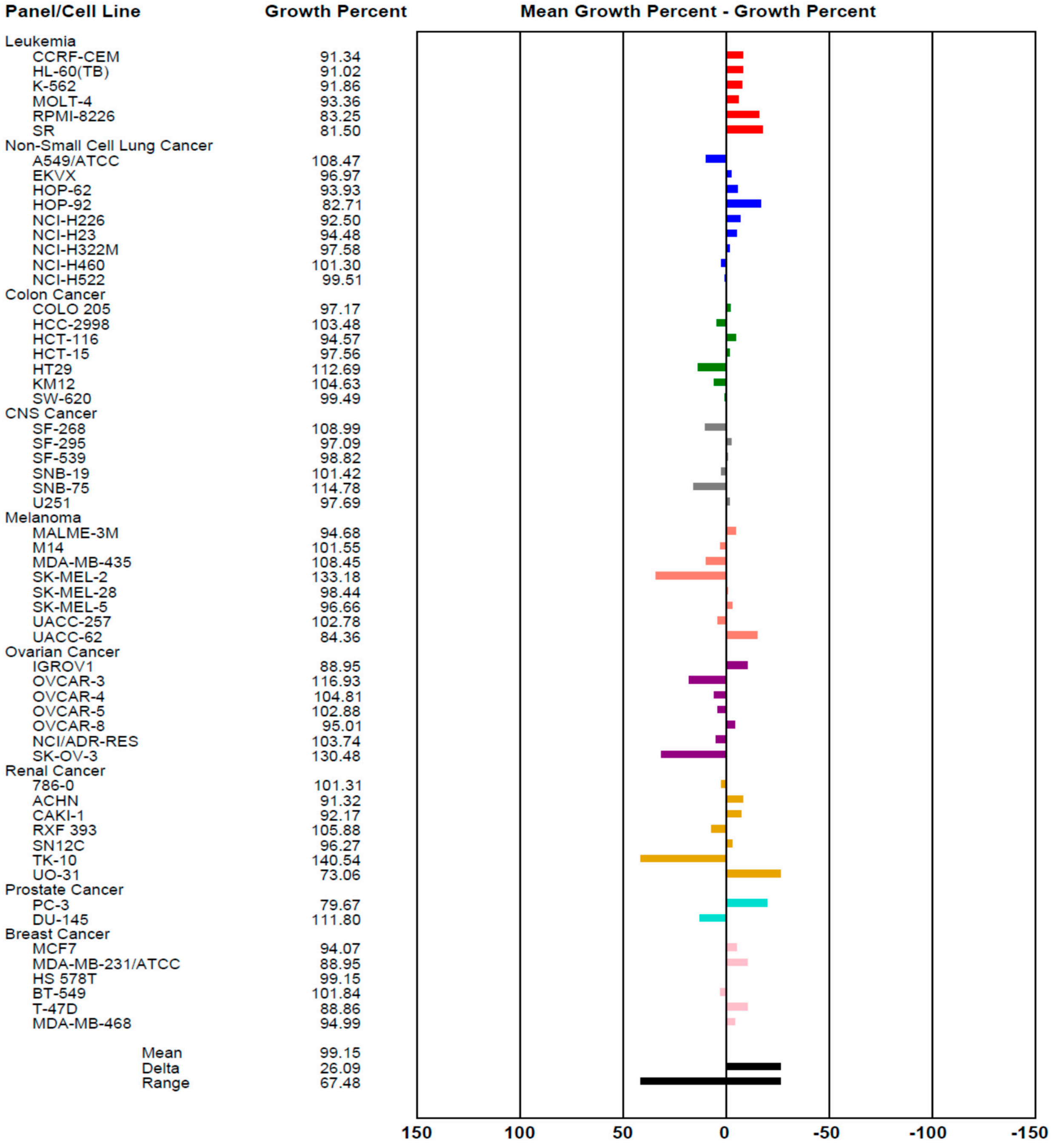

Fig.45. One-dose growth (%) and mean graph of compound **6b**

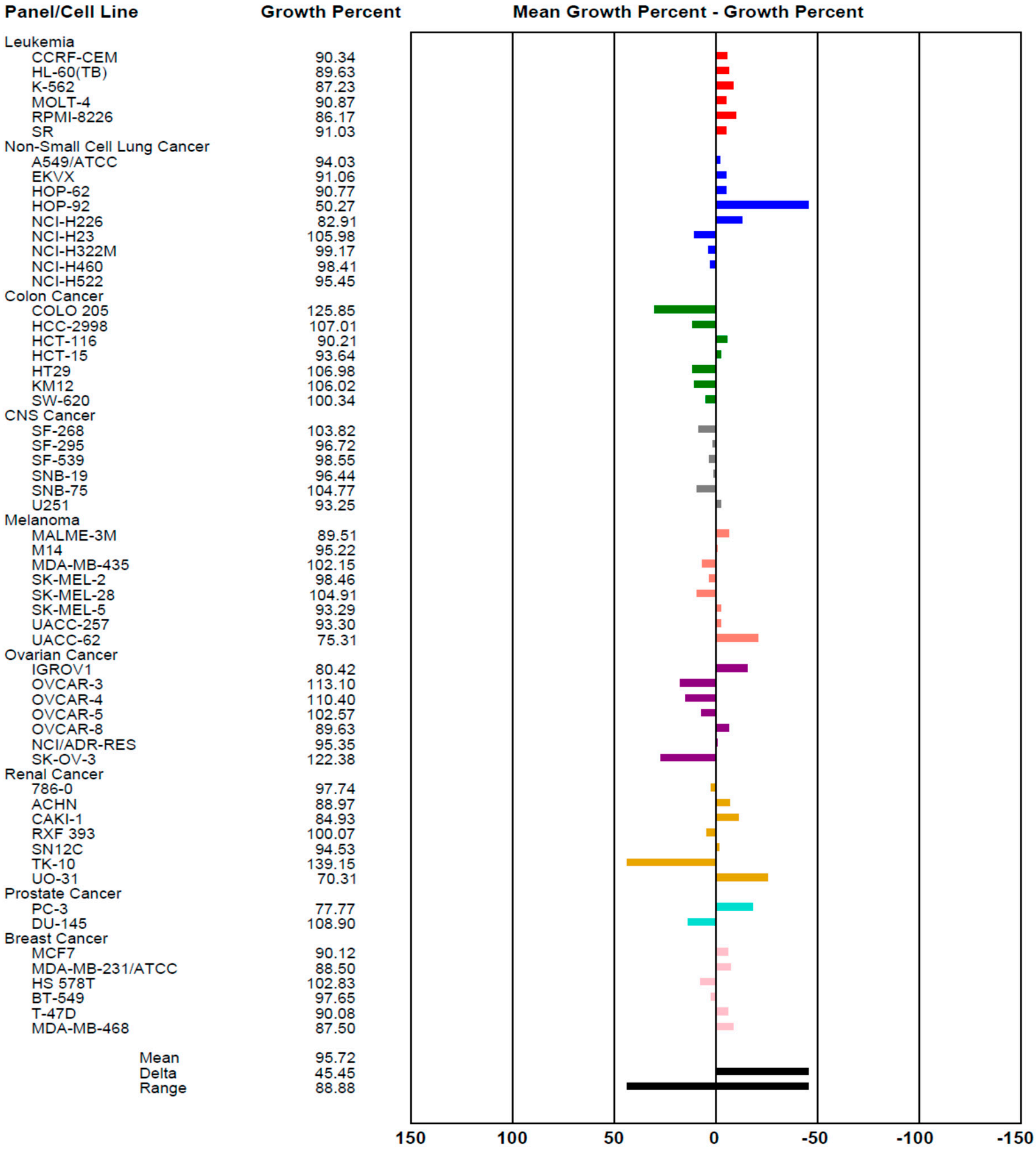

Fig.46. One-dose growth (%) and mean graph of compound **6c**

Panel/Cell Line	Growth Percent
Leukemia	
CCRF-CEM	97.92
HL-60(TB)	92.29
K-562	85.54
MOLT-4	91.18
RPMI-8226	98.83
SR	88.71
Non-Small Cell Lung Cancer	
A549/ATCC	103.29
EKVX	92.78
HOP-62	102.78
HOP-92	91.40
NCI-H226	90.07
NCI-H23	95.02
NCI-H322M	99.45
NCI-H460	106.13
NCI-H522	96.30
Colon Cancer	
COLO 205	107.53
HCC-2998	104.47
HCT-116	93.47
HCT-15	99.02
HT29	104.18
KM12	102.46
SW-620	110.16
CNS Cancer	
SF-268	101.95
SF-295	93.22
SF-539	91.77
SNB-19	100.26
SNB-75	106.14
U251	99.88
Melanoma	
MALME-3M	90.66
M14	97.36
MDA-MB-435	108.05
SK-MEL-2	115.27
SK-MEL-28	96.10
SK-MEL-5	95.93
UACC-257	107.60
UACC-62	83.09
Ovarian Cancer	
IGROV1	86.74
OVCAR-3	110.42
OVCAR-4	108.69
OVCAR-5	96.81
OVCAR-8	94.70
NCI/ADR-RES	98.83
SK-OV-3	113.09
Renal Cancer	
786-0	98.56
ACHN	88.26
CAKI-1	90.23
RXF 393	112.81
SN12C	95.61
TK-10	134.30
UO-31	68.40
Prostate Cancer	
PC-3	78.32
DU-145	106.19
Breast Cancer	
MCF7	97.89
MDA-MB-231/ATCC	96.85
HS 578T	105.71
BT-549	105.98
T-47D	97.02
MDA-MB-468	86.94
Mean	98.49
Delta	30.09
Range	65.90

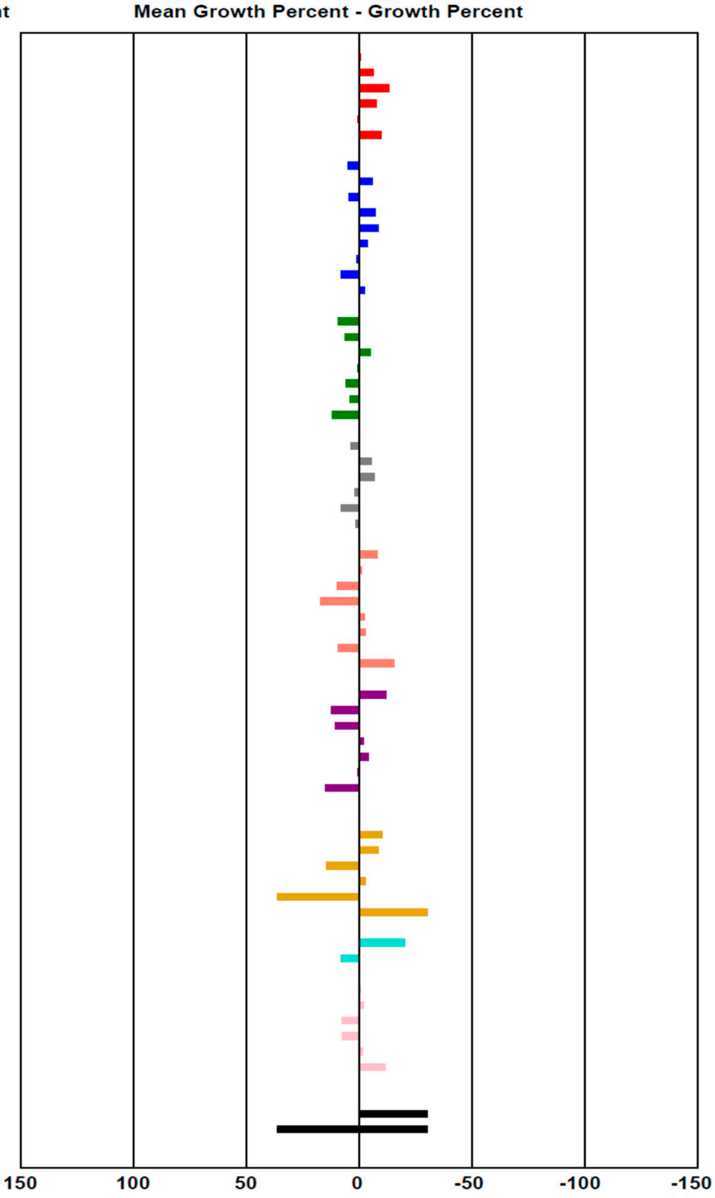

Fig.47. One-dose growth (%) and mean graph of compound **6d**

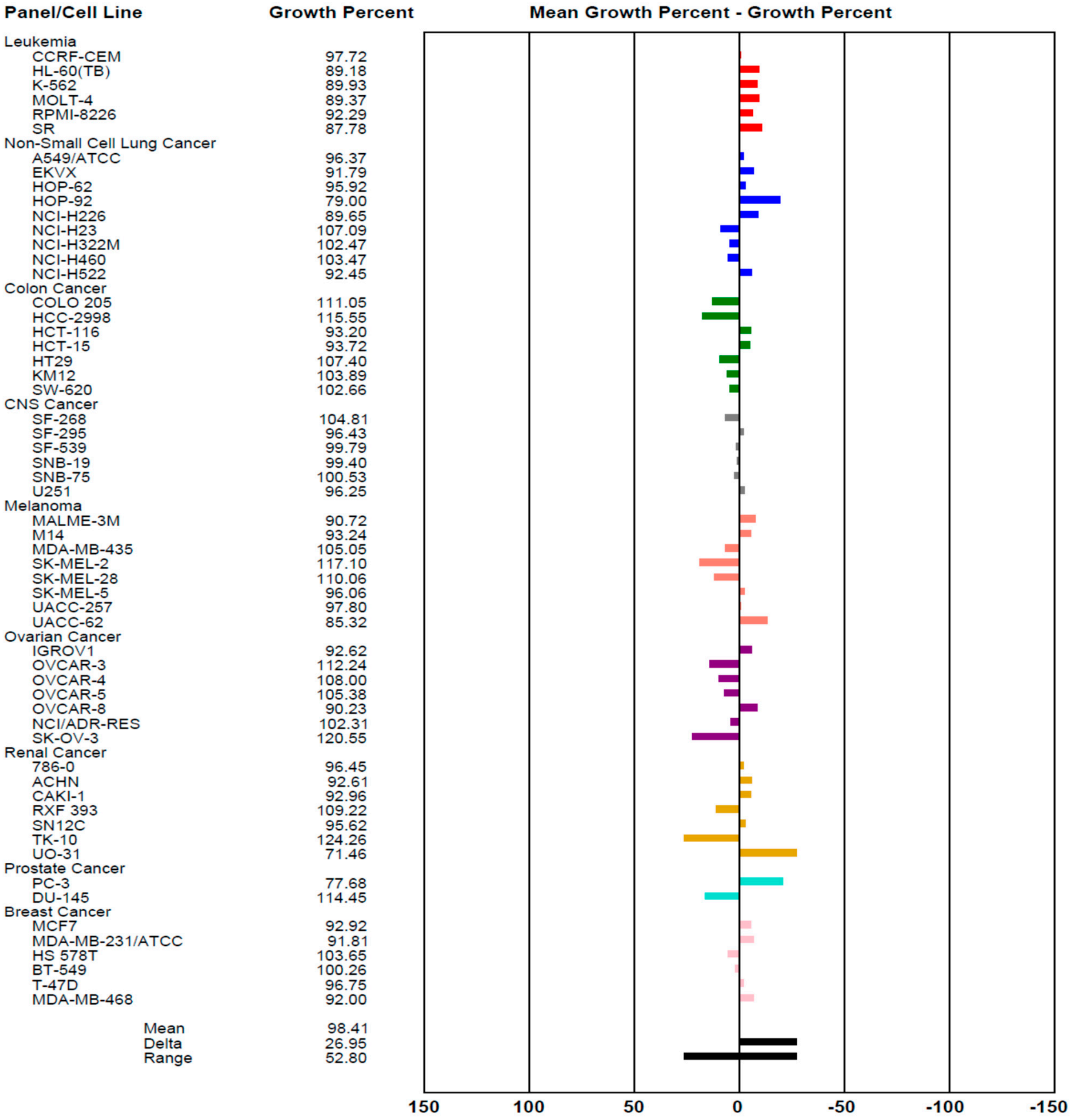

Fig. 48. One-dose growth (%) and mean graph of compound **6e**

Panel/Cell Line	Growth Percent
**Leukemia**	
CCRF-CEM	91.35
HL-60(TB)	87.21
K-562	52.78
MOLT-4	90.57
RPMI-8226	81.32
SR	81.56
**Non-Small Cell Lung Cancer**	
A549/ATCC	85.50
EKVX	68.40
HOP-62	23.44
HOP-92	47.02
NCI-H226	4.48
NCI-H23	44.91
NCI-H322M	81.21
NCI-H460	61.80
NCI-H522	71.60
**Colon Cancer**	
COLO 205	93.37
HCC-2998	83.09
HCT-116	38.04
HCT-15	79.45
HT29	47.93
KM12	69.46
SW-620	76.09
**CNS Cancer**	
SF-268	31.72
SF-295	71.99
SF-539	7.59
SNB-19	19.19
SNB-75	100.34
U251	10.78
**Melanoma**	
LOX IMVI	29.22
MALME-3M	37.40
M14	73.63
MDA-MB-435	87.22
SK-MEL-2	91.86
SK-MEL-28	99.96
SK-MEL-5	90.70
UACC-257	103.20
UACC-62	73.40
**Ovarian Cancer**	
IGROV1	55.09
OVCAR-3	60.81
OVCAR-4	-2.47
OVCAR-5	88.57
OVCAR-8	14.66
NCI/ADR-RES	14.78
SK-OV-3	85.63
**Renal Cancer**	
786-0	29.61
A498	106.16
ACHN	60.47
CAKI-1	53.23
SN12C	44.68
TK-10	72.01
UO-31	55.54
**Prostate Cancer**	
PC-3	65.76
DU-145	85.67
**Breast Cancer**	
MCF7	36.17
MDA-MB-231/ATCC	27.03
HS 578T	24.13
BT-549	62.24
T-47D	83.54
MDA-MB-468	77.20
Mean	60.84
Delta	63.31
Range	108.63

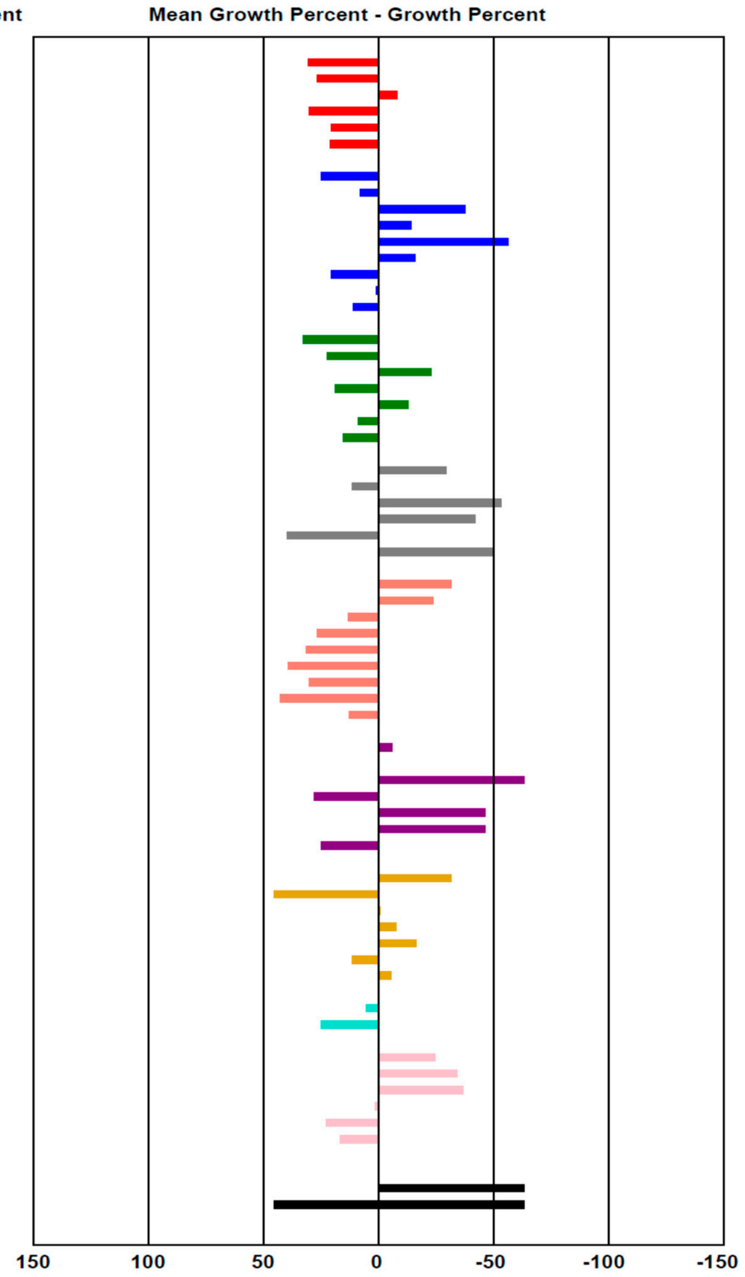

Fig.49. One-dose growth (%) and mean graph of compound **6f**

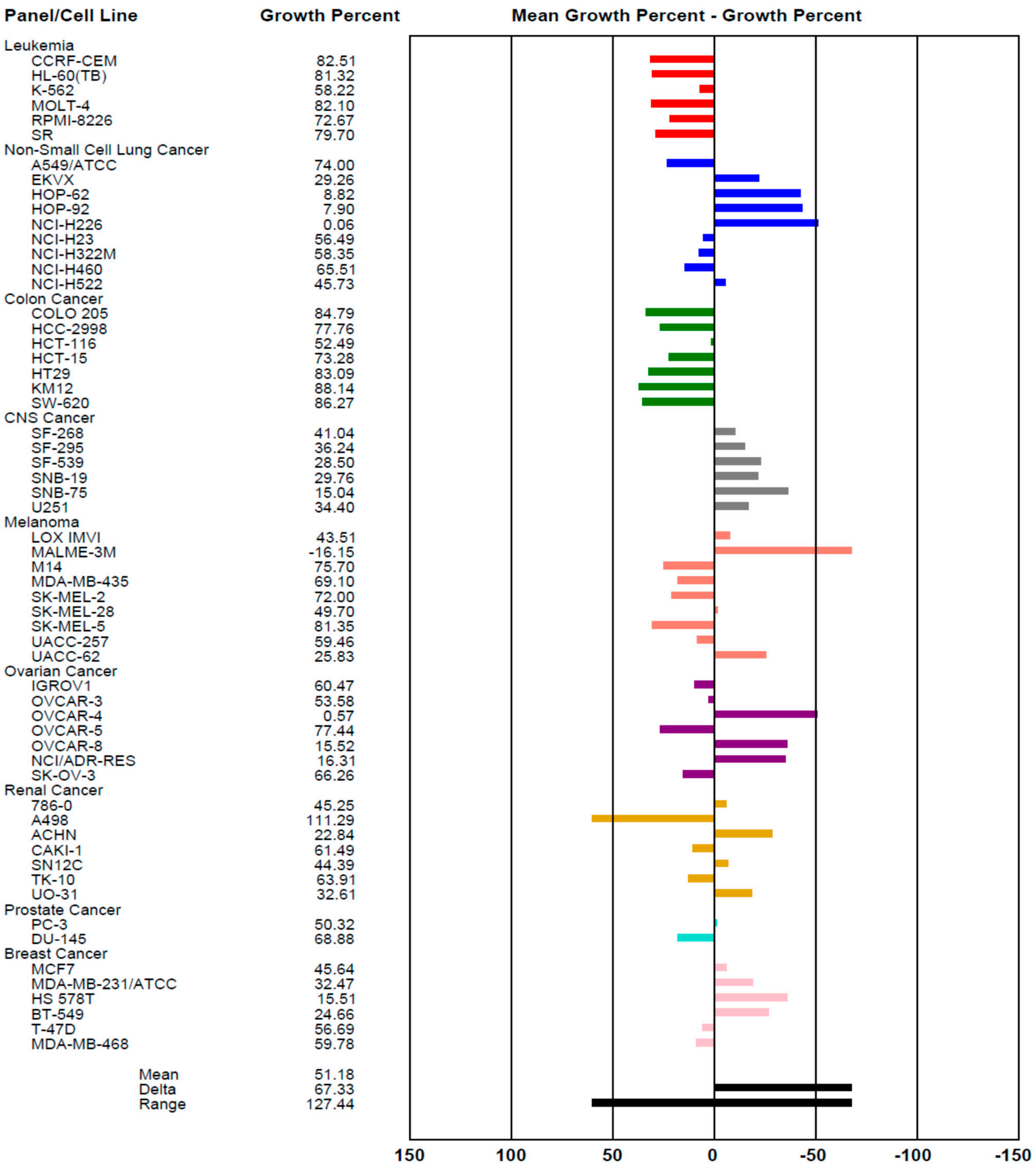

Fig. 50. One-dose growth (%) and mean graph of compound **6g**

Panel/Cell Line	Growth Percent
Leukemia	
CCRF-CEM	60.84
HL-60(TB)	43.35
K-562	27.16
MOLT-4	61.74
RPMI-8226	58.94
SR	53.04
Non-Small Cell Lung Cancer	
A549/ATCC	53.27
EKVX	53.02
HOP-62	7.80
HOP-92	-7.56
NCI-H226	-41.33
NCI-H23	17.33
NCI-H322M	74.88
NCI-H460	29.27
NCI-H522	1.43
Colon Cancer	
COLO 205	75.02
HCC-2998	66.38
HCT-116	7.68
HCT-15	43.79
HT29	7.13
KM12	51.04
SW-620	19.01
CNS Cancer	
SF-268	29.74
SF-295	11.06
SF-539	22.13
SNB-19	19.66
SNB-75	-1.21
U251	9.78
Melanoma	
LOX IMVI	-19.53
MALME-3M	35.15
M14	51.15
MDA-MB-435	38.58
SK-MEL-2	84.70
SK-MEL-28	48.95
SK-MEL-5	32.88
UACC-257	77.28
UACC-62	23.86
Ovarian Cancer	
IGROV1	24.23
OVCAR-3	65.20
OVCAR-4	-15.56
OVCAR-5	13.03
OVCAR-8	11.29
NCI/ADR-RES	-27.71
SK-OV-3	24.03
Renal Cancer	
786-0	-35.44
A498	85.81
ACHN	20.39
CAKI-1	12.36
SN12C	24.88
TK-10	-2.18
UO-31	30.35
Prostate Cancer	
PC-3	43.03
DU-145	78.95
Breast Cancer	
MCF7	12.48
MDA-MB-231/ATCC	-2.39
HS 578T	-0.73
BT-549	22.36
T-47D	46.66
MDA-MB-468	47.98
Mean	28.92
Delta	70.25
Range	127.14

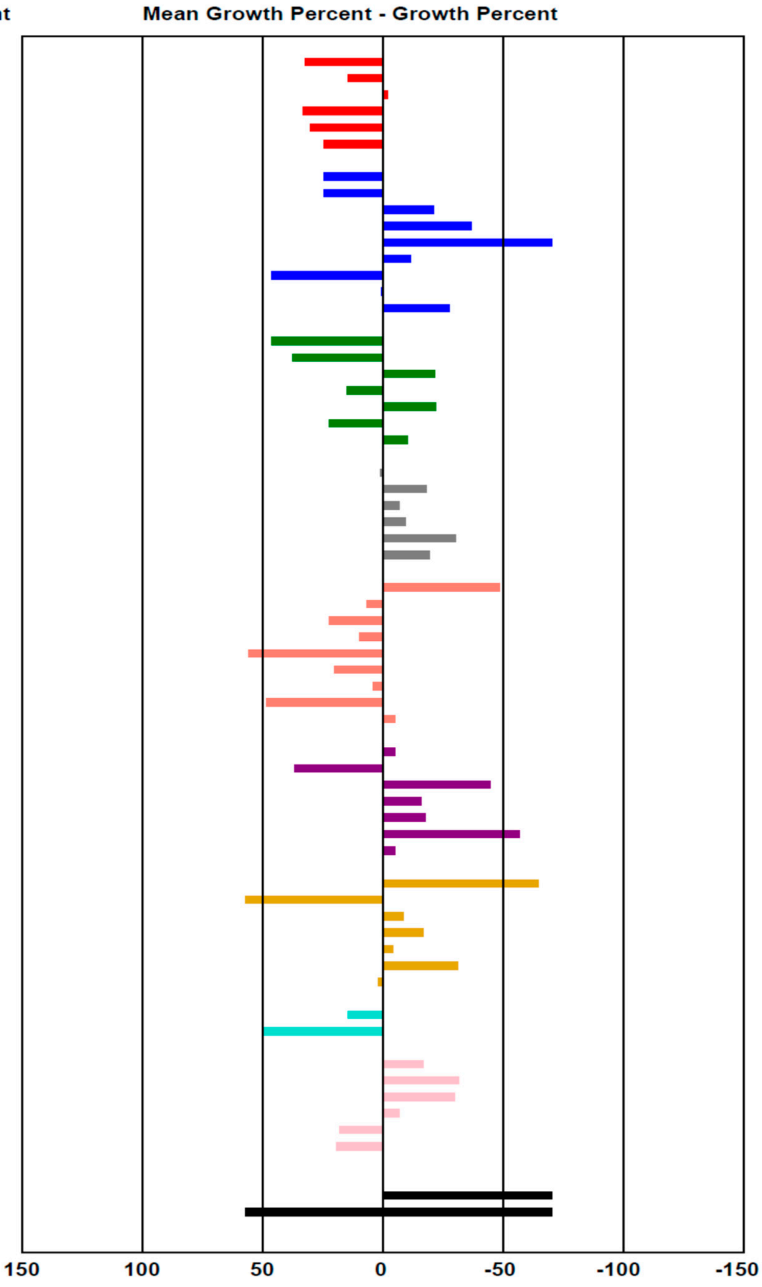

Fig. 51. One-dose growth (%) and mean graph of compound **6h**

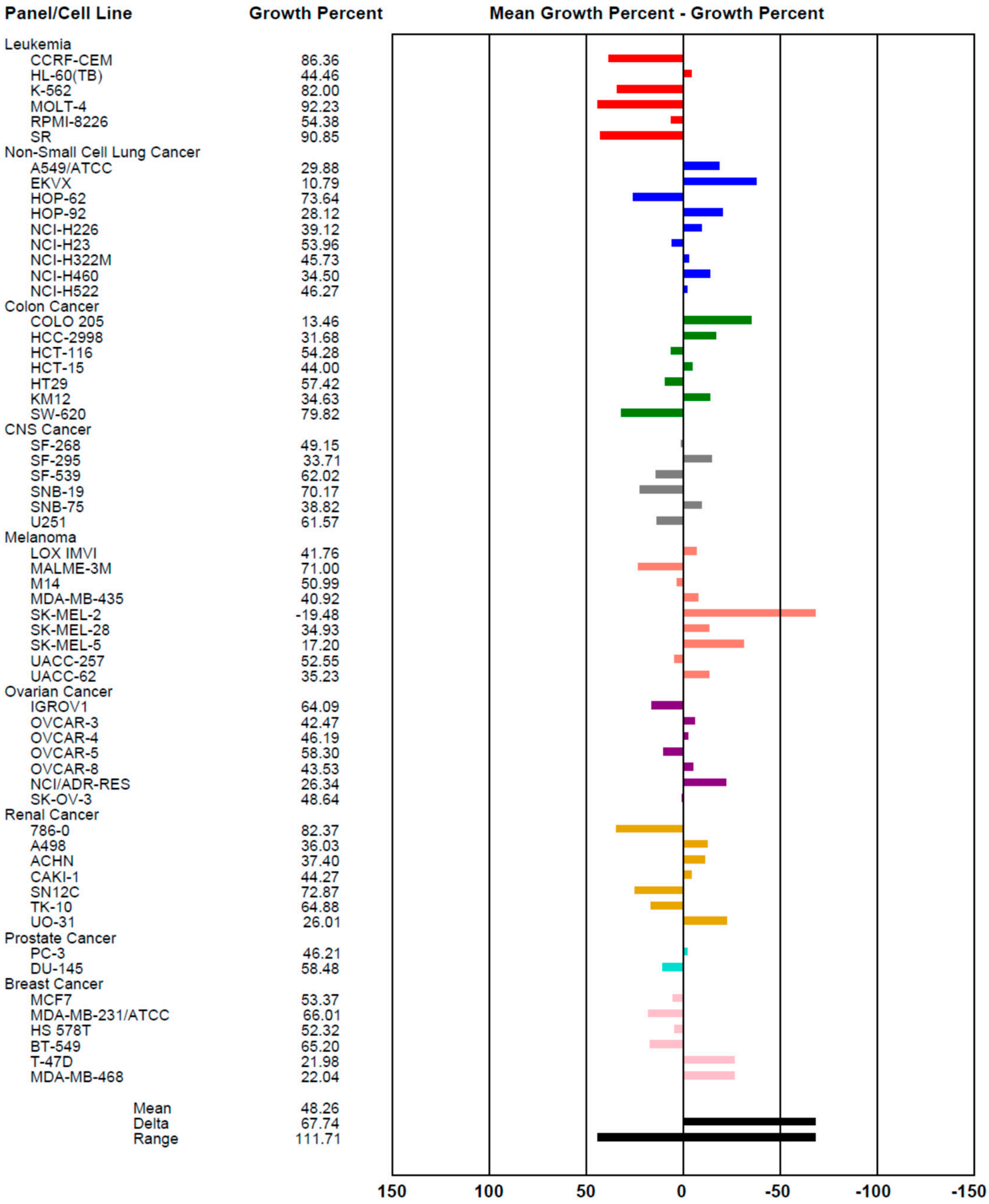

Fig. 52. One-dose growth (%) and mean graph of compound **6i**

Panel/Cell Line	Growth Percent
Leukemia	
CCRF-CEM	92.74
HL-60(TB)	88.93
K-562	68.99
MOLT-4	103.56
RPMI-8226	83.17
SR	92.37
Non-Small Cell Lung Cancer	
A549/ATCC	37.55
EKVX	25.63
HOP-62	47.05
HOP-92	65.05
NCI-H226	17.61
NCI-H23	40.78
NCI-H322M	58.68
NCI-H460	60.09
NCI-H522	66.98
Colon Cancer	
COLO 205	42.59
HCC-2998	78.13
HCT-116	45.75
HCT-15	73.31
HT29	42.56
KM12	78.73
SW-620	70.32
CNS Cancer	
SF-268	69.37
SF-295	55.03
SF-539	49.48
SNB-19	40.02
SNB-75	54.73
U251	30.14
Melanoma	
LOX IMVI	15.88
MALME-3M	63.98
M14	80.59
MDA-MB-435	79.16
SK-MEL-2	33.67
SK-MEL-28	68.30
SK-MEL-5	78.20
UACC-257	72.30
UACC-62	52.80
Ovarian Cancer	
IGROV1	51.44
OVCAR-3	73.94
OVCAR-4	14.90
OVCAR-5	90.72
OVCAR-8	24.49
NCI/ADR-RES	5.85
SK-OV-3	72.06
Renal Cancer	
786-0	44.88
A498	57.26
ACHN	57.49
CAKI-1	64.46
SN12C	64.63
TK-10	83.96
UO-31	54.78
Prostate Cancer	
PC-3	60.45
DU-145	88.43
Breast Cancer	
MCF7	38.40
MDA-MB-231/ATCC	51.74
HS 578T	33.21
BT-549	69.76
T-47D	54.59
MDA-MB-468	46.40
Mean	58.10
Delta	52.25
Range	97.71

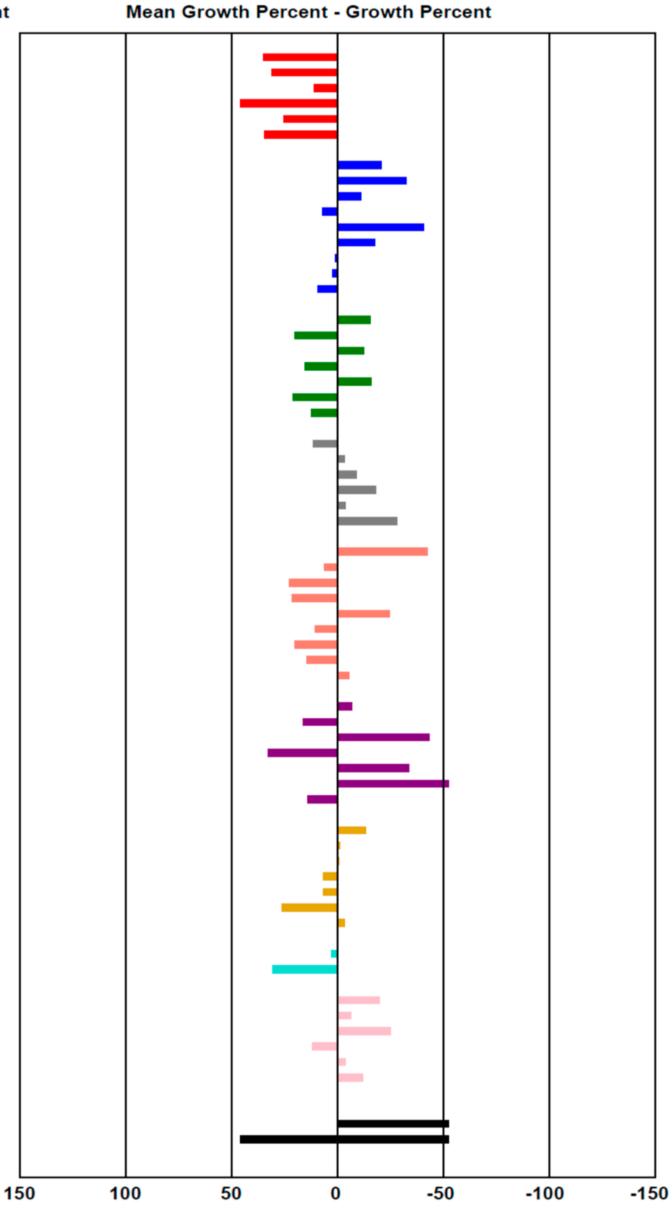

Fig. 53. One-dose growth (%) and mean graph of compound **6j**

**Leukemia**	
CCRF-CEM	99.34
HL-60(TB)	90.66
K-562	96.71
MOLT-4	97.49
RPMI-8226	99.62
SR	110.27
**Non-Small Cell Lung Cancer**	
A549/ATCC	106.67
EKVX	94.40
HOP-62	90.34
HOP-92	94.95
NCI-H226	87.89
NCI-H23	101.28
NCI-H322M	100.17
NCI-H460	96.66
NCI-H522	95.52
**Colon Cancer**	
COLO 205	97.96
HCC-2998	99.16
HCT-116	90.76
HCT-15	95.59
HT29	103.61
KM12	102.55
SW-620	99.73
**CNS Cancer**	
SF-268	107.26
SF-295	87.64
SF-539	96.74
SNB-19	93.18
SNB-75	103.17
U251	95.38
**Melanoma**	
MALME-3M	92.40
M14	113.41
MDA-MB-435	105.56
SK-MEL-2	106.77
SK-MEL-28	104.36
SK-MEL-5	98.43
UACC-257	113.10
UACC-62	86.43
**Ovarian Cancer**	
IGROV1	92.40
OVCAR-3	114.95
OVCAR-4	108.19
OVCAR-5	103.15
OVCAR-8	98.84
NCI/ADR-RES	106.66
SK-OV-3	97.79
**Renal Cancer**	
786-0	95.09
ACHN	96.18
CAKI-1	93.29
RXF 393	104.50
SN12C	97.60
TK-10	112.21
UO-31	79.06
**Prostate Cancer**	
PC-3	90.23
DU-145	105.82
**Breast Cancer**	
MCF7	102.96
MDA-MB-231/ATCC	96.10
HS 578T	94.27
BT-549	103.51
T-47D	91.33
MDA-MB-468	84.64
Mean	98.69
Delta	19.63
Range	35.89

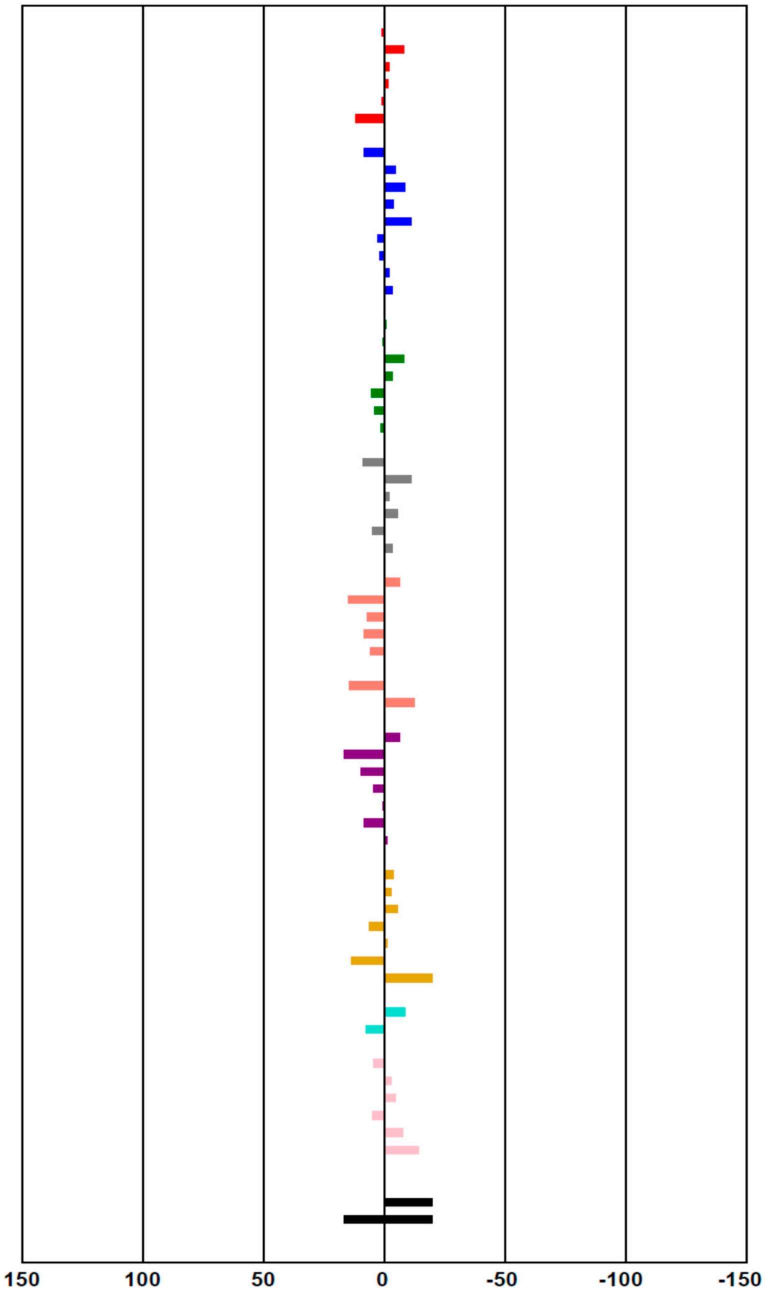

Fig. 54. One-dose growth (%) and mean graph of compound **7a**

Panel/Cell Line	Growth Percent
**Leukemia**	
CCRF-CEM	91.17
HL-60(TB)	83.94
K-562	86.36
MOLT-4	91.82
RPMI-8226	85.10
SR	96.24
**Non-Small Cell Lung Cancer**	
A549/ATCC	109.29
EKVX	92.51
HOP-62	98.00
HOP-92	91.09
NCI-H226	85.09
NCI-H23	95.14
NCI-H322M	101.52
NCI-H460	94.66
NCI-H522	91.67
**Colon Cancer**	
COLO 205	117.92
HCC-2998	108.35
HCT-116	76.44
HCT-15	95.66
HT29	105.85
KM12	105.28
SW-620	102.43
**CNS Cancer**	
SF-268	100.98
SF-295	83.35
SF-539	95.63
SNB-19	98.35
SNB-75	112.95
U251	105.26
**Melanoma**	
MALME-3M	96.80
M14	97.17
MDA-MB-435	110.63
SK-MEL-2	107.88
SK-MEL-28	103.89
SK-MEL-5	89.54
UACC-257	111.52
UACC-62	79.09
**Ovarian Cancer**	
IGROV1	82.43
OVCAR-3	109.23
OVCAR-4	101.81
OVCAR-5	104.10
OVCAR-8	92.90
NCI/ADR-RES	103.18
SK-OV-3	98.14
**Renal Cancer**	
786-0	99.27
ACHN	92.44
CAKI-1	98.50
RXF 393	112.09
SN12C	91.76
TK-10	127.16
UO-31	80.77
**Prostate Cancer**	
PC-3	85.09
DU-145	105.85
**Breast Cancer**	
MCF7	88.69
MDA-MB-231/ATCC	86.53
HS 578T	97.39
BT-549	103.80
T-47D	73.60
MDA-MB-468	83.68
Mean	96.84
Delta	23.24
Range	53.56

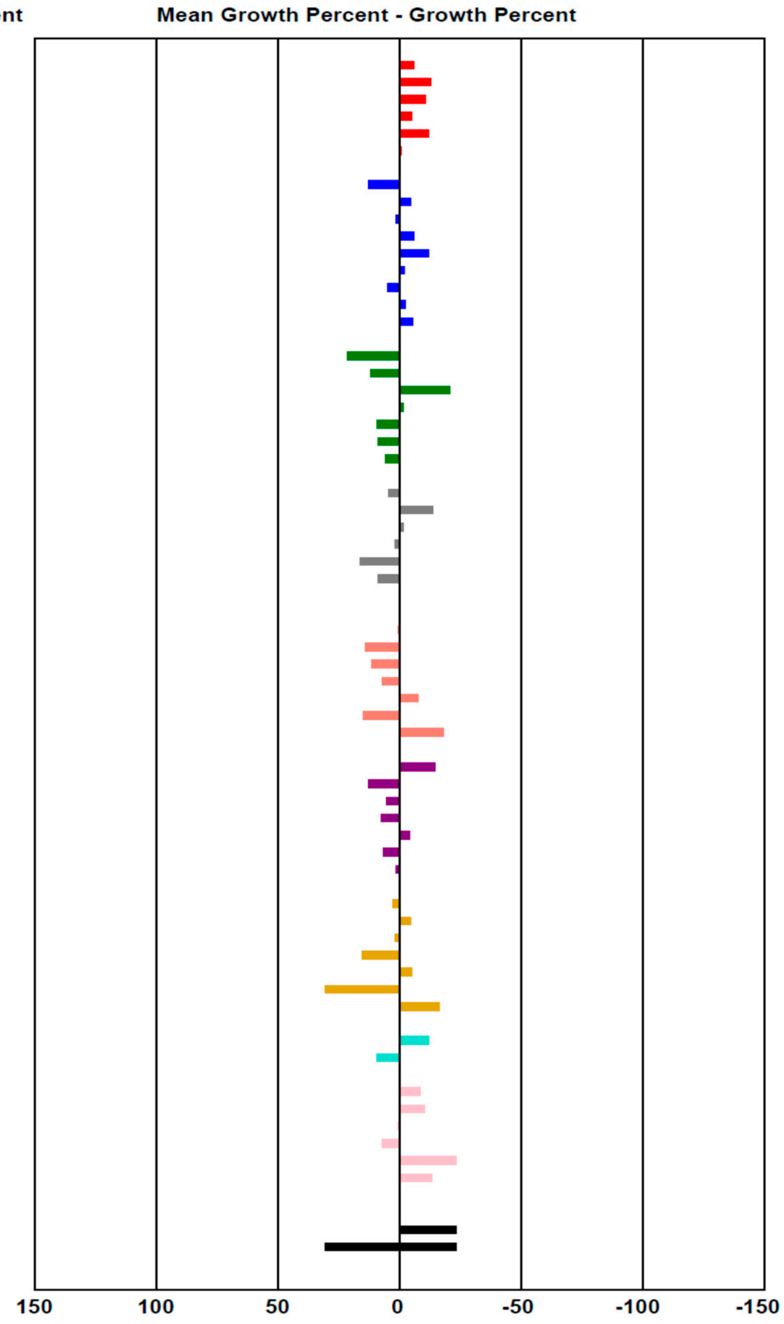

Fig. 55. One-dose growth (%) and mean graph of compound **7b**

Panel/Cell Line	Growth Percent
Leukemia	
CCRF-CEM	97.09
HL-60(TB)	90.00
K-562	86.67
MOLT-4	79.60
RPMI-8226	91.24
SR	83.50
Non-Small Cell Lung Cancer	
A549/ATCC	95.10
EKVX	82.26
HOP-62	91.28
HOP-92	92.02
NCI-H226	76.94
NCI-H23	90.71
NCI-H322M	94.33
NCI-H460	90.80
NCI-H522	85.40
Colon Cancer	
COLO 205	106.48
HCC-2998	98.50
HCT-116	77.70
HCT-15	88.71
HT29	104.09
KM12	99.73
SW-620	95.94
CNS Cancer	
SF-268	96.37
SF-295	84.88
SF-539	101.42
SNB-19	91.96
SNB-75	97.42
U251	102.94
Melanoma	
MALME-3M	90.83
M14	94.41
MDA-MB-435	102.07
SK-MEL-2	98.99
SK-MEL-28	105.62
SK-MEL-5	88.95
UACC-257	106.62
UACC-62	69.05
Ovarian Cancer	
IGROV1	76.75
OVCAR-3	106.11
OVCAR-4	93.89
OVCAR-5	98.68
OVCAR-8	91.51
NCI/ADR-RES	98.73
SK-OV-3	100.26
Renal Cancer	
786-0	102.20
ACHN	88.62
CAKI-1	79.80
RXF 393	105.46
SN12C	88.31
TK-10	126.79
UO-31	65.01
Prostate Cancer	
PC-3	92.38
DU-145	102.38
Breast Cancer	
MCF7	88.12
MDA-MB-231/ATCC	95.99
HS 578T	93.45
BT-549	107.22
T-47D	73.51
MDA-MB-468	72.85
Mean	92.72
Delta	27.71
Range	61.78

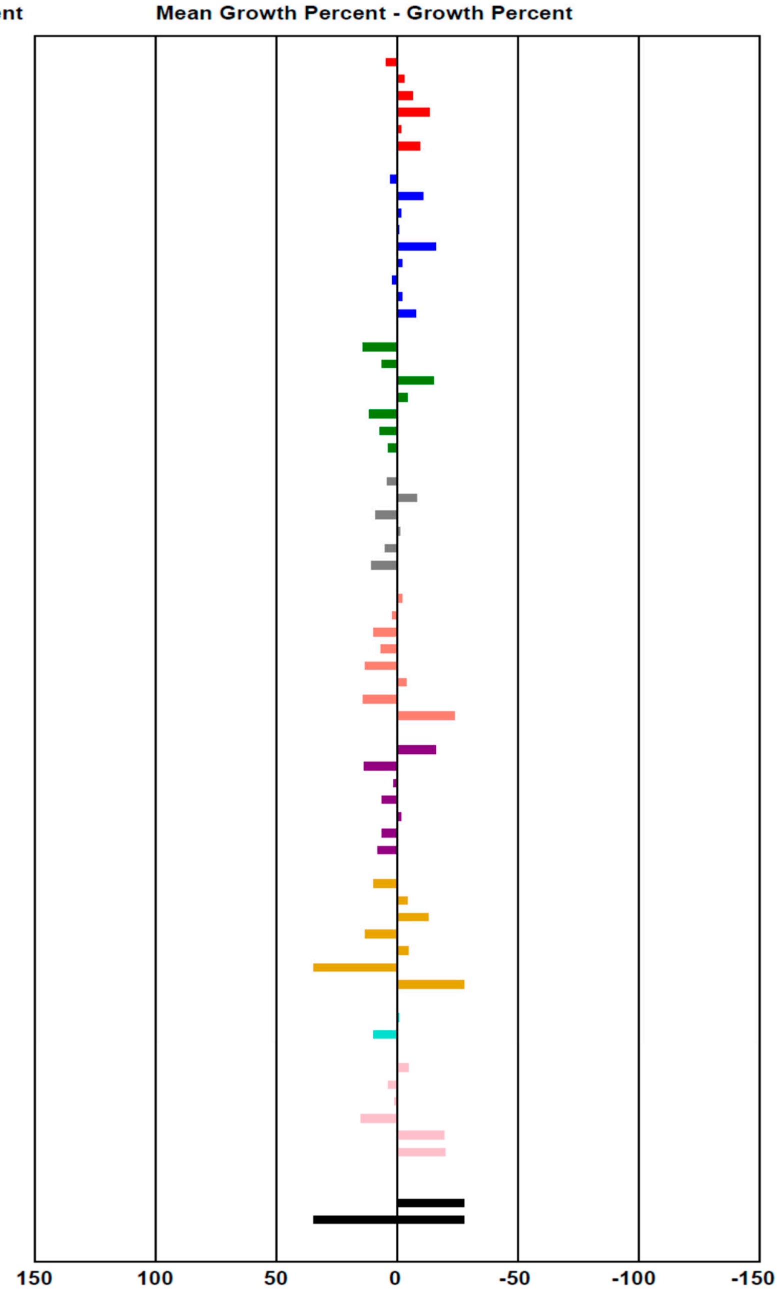

Fig.56. One-dose growth (%) and mean graph of compound **7c**

Panel/Cell Line	Growth Percent
Leukemia	
CCRF-CEM	73.26
HL-60(TB)	64.32
K-562	59.22
MOLT-4	56.26
RPMI-8226	62.11
SR	71.53
Non-Small Cell Lung Cancer	
A549/ATCC	90.83
EKVX	74.25
HOP-62	99.86
HOP-92	79.01
NCI-H226	66.40
NCI-H23	82.13
NCI-H322M	83.74
NCI-H460	80.26
NCI-H522	81.54
Colon Cancer	
COLO 205	91.75
HCC-2998	103.65
HCT-116	52.98
HCT-15	73.11
HT29	96.65
KM12	91.42
SW-620	92.69
CNS Cancer	
SF-268	95.02
SF-295	67.55
SF-539	86.00
SNB-19	91.64
SNB-75	93.98
U251	94.85
Melanoma	
MALME-3M	95.16
M14	80.75
MDA-MB-435	88.53
SK-MEL-2	91.03
SK-MEL-28	92.18
SK-MEL-5	59.12
UACC-257	101.32
UACC-62	63.74
Ovarian Cancer	
IGROV1	69.20
OVCAR-3	103.34
OVCAR-4	75.53
OVCAR-5	93.18
OVCAR-8	81.70
NCI/ADR-RES	93.29
SK-OV-3	96.72
Renal Cancer	
786-0	86.57
ACHN	82.13
CAKI-1	74.17
RXF 393	80.72
SN12C	83.72
TK-10	87.09
UO-31	61.68
Prostate Cancer	
PC-3	68.38
DU-145	93.79
Breast Cancer	
MCF7	82.09
MDA-MB-231/ATCC	76.42
HS 578T	77.87
BT-549	79.62
T-47D	60.23
MDA-MB-468	57.84
Mean	80.92
Delta	27.94
Range	50.67

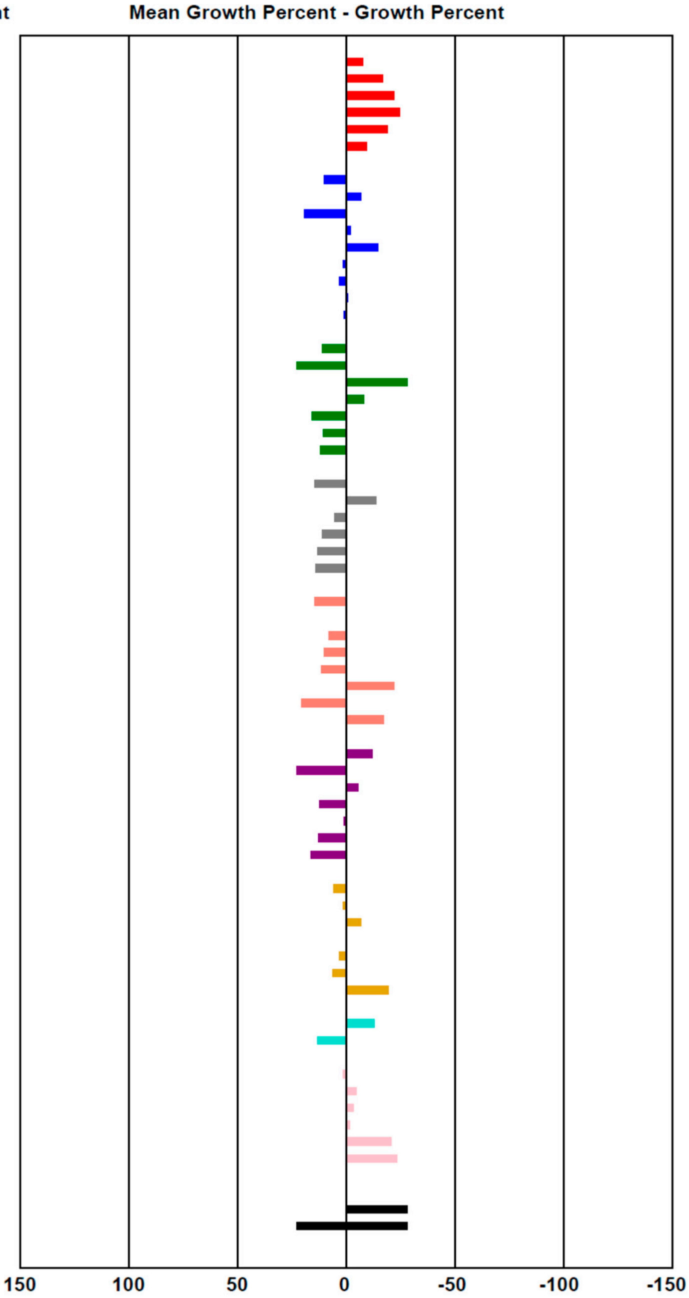

Fig. 57. One-dose growth (%) and mean graph of compound **7d**

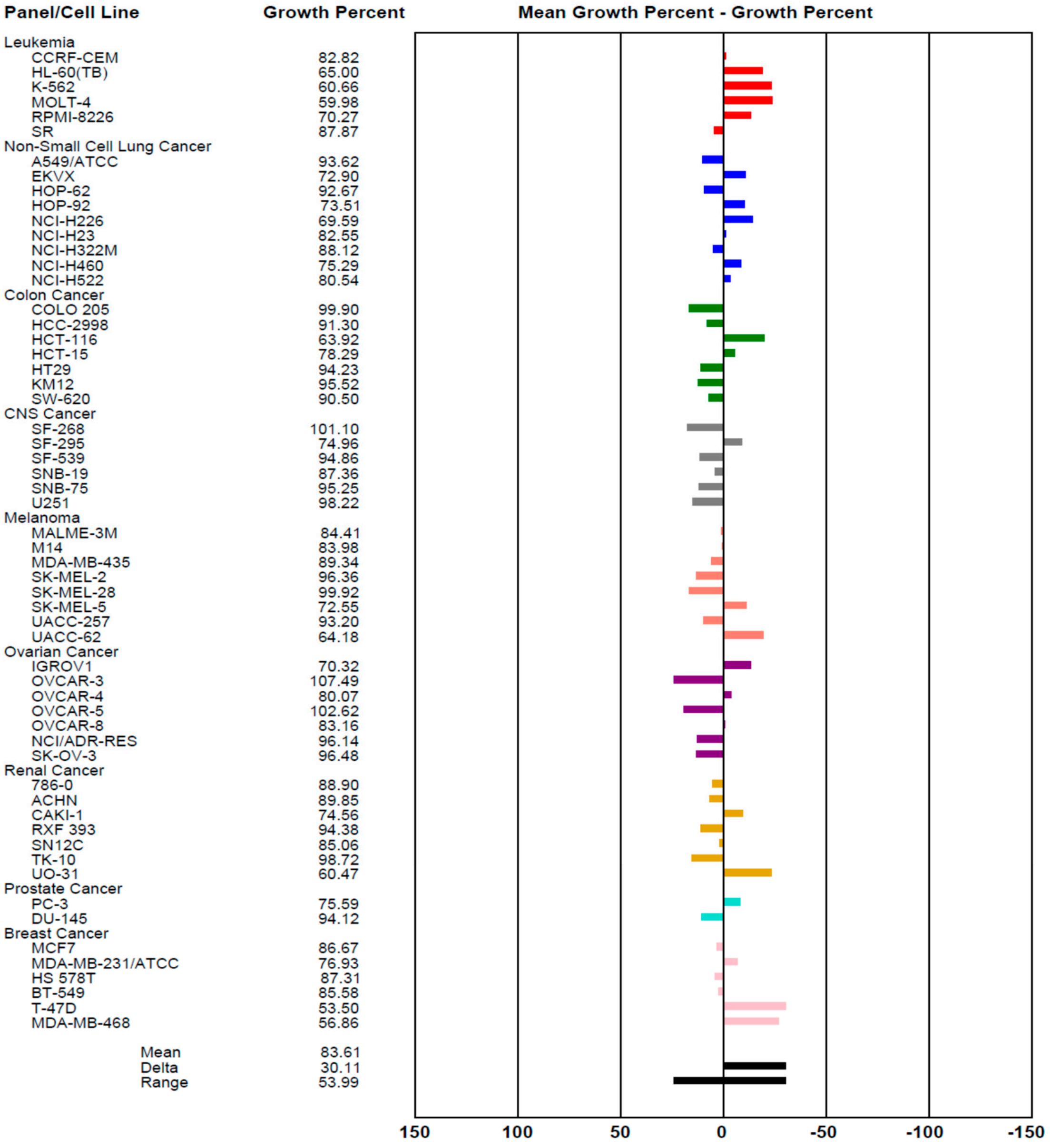

Fig. 58. One-dose growth (%) and mean graph of compound **7e**

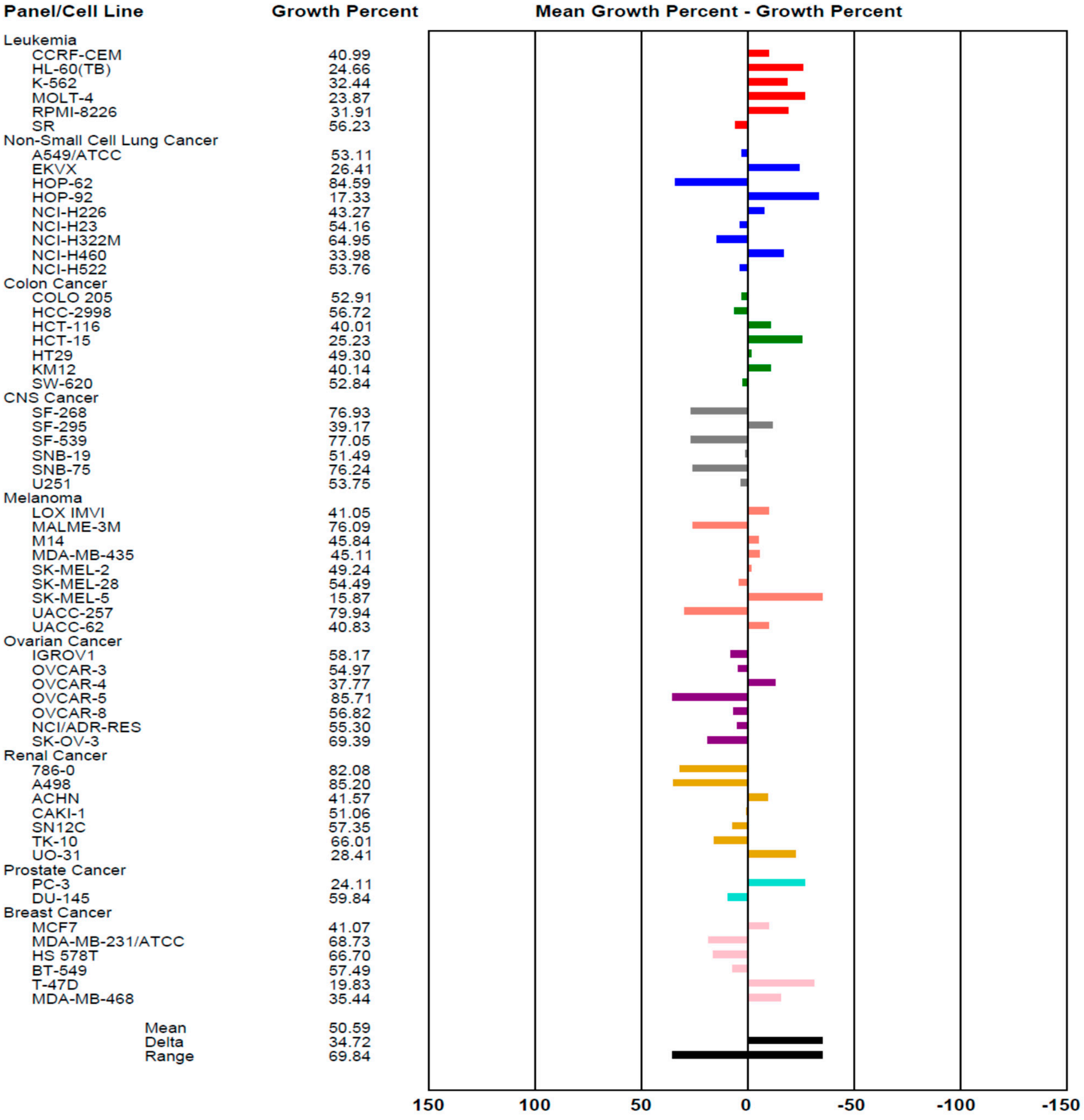

Fig.59. One-dose growth (%) and mean graph of compound **7f**

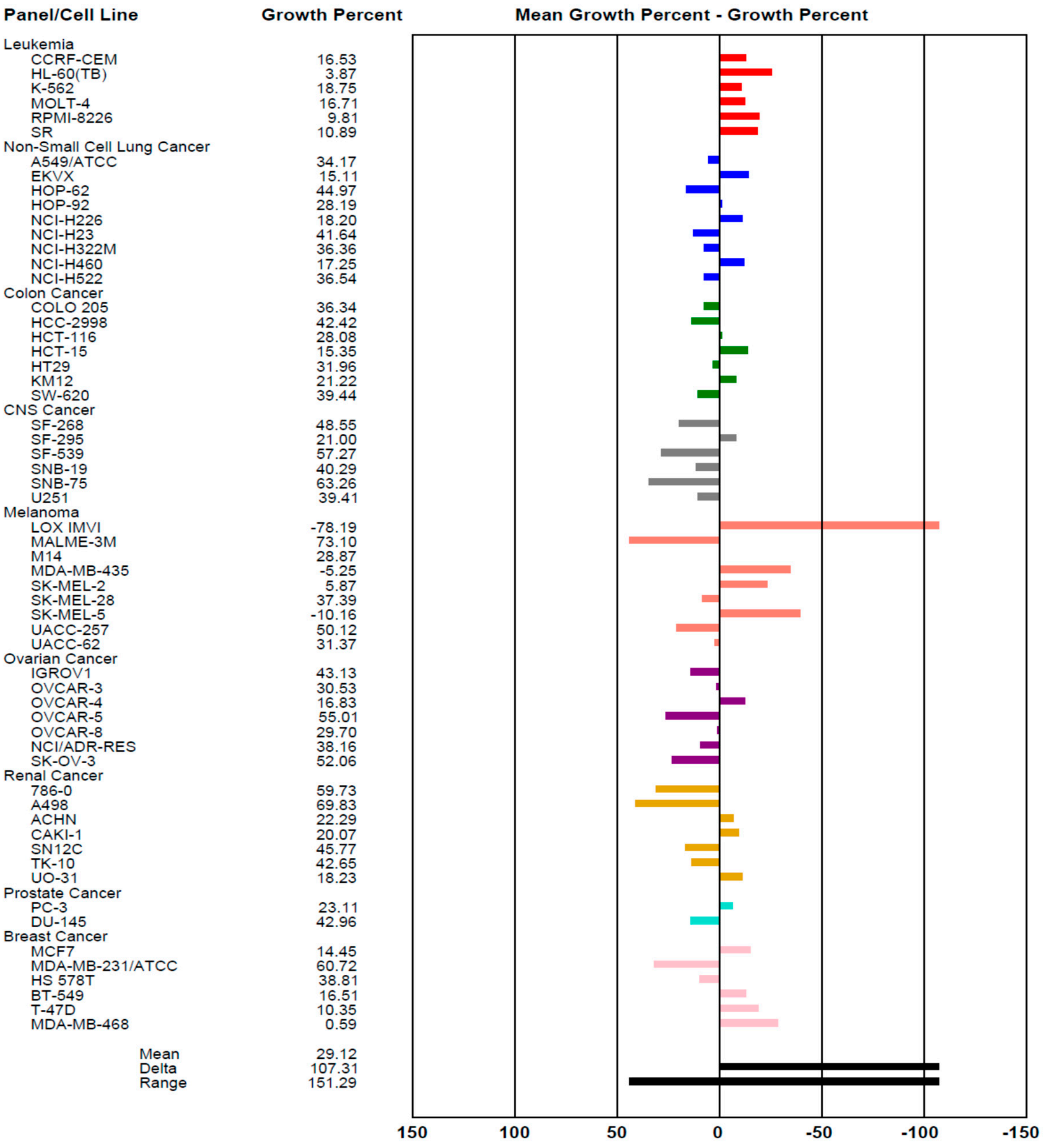

Fig. 60. One-dose growth (%) and mean graph of compound **7g**

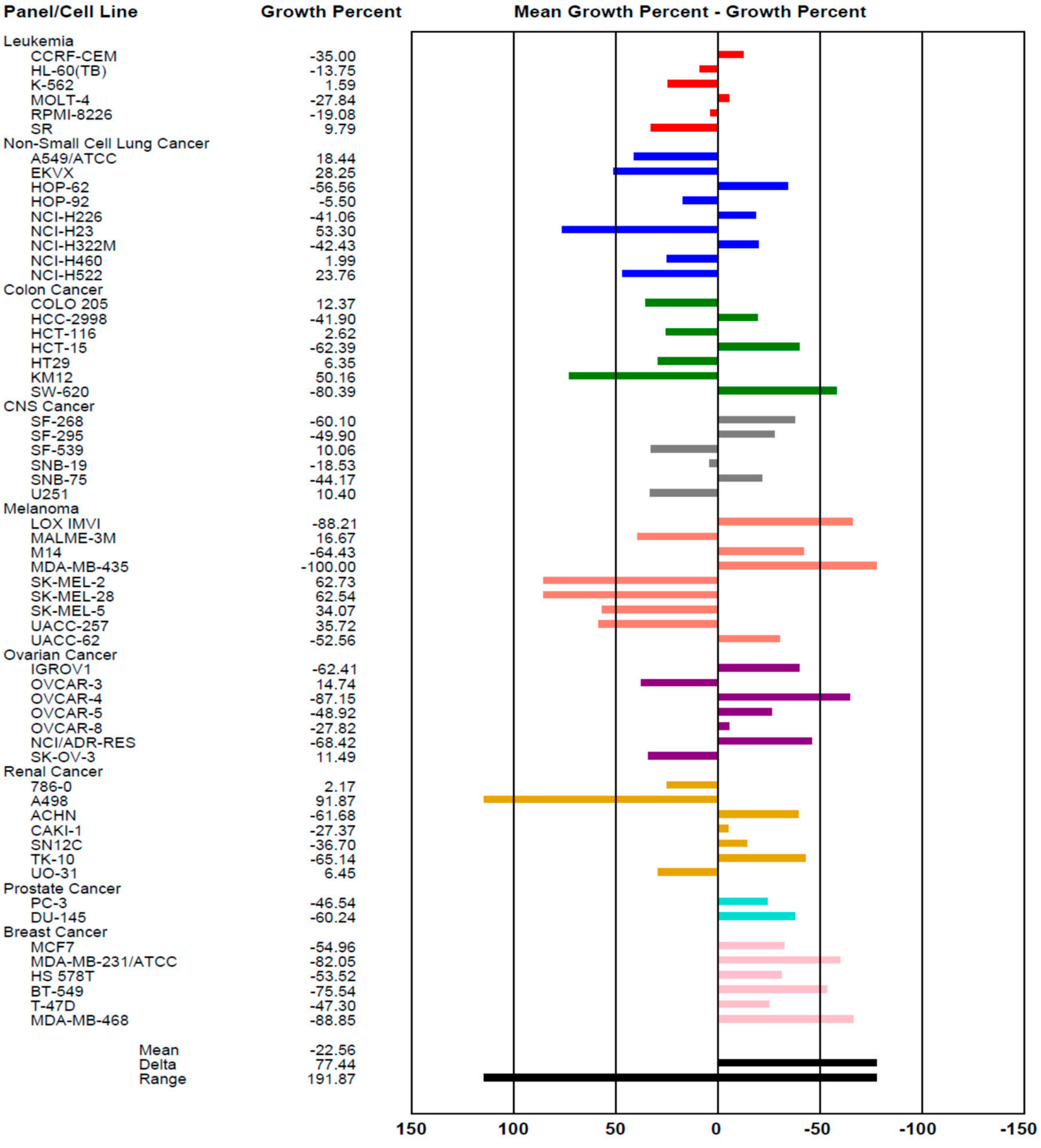

Fig. 61. One-dose growth (%) and mean graph of compound **7h**

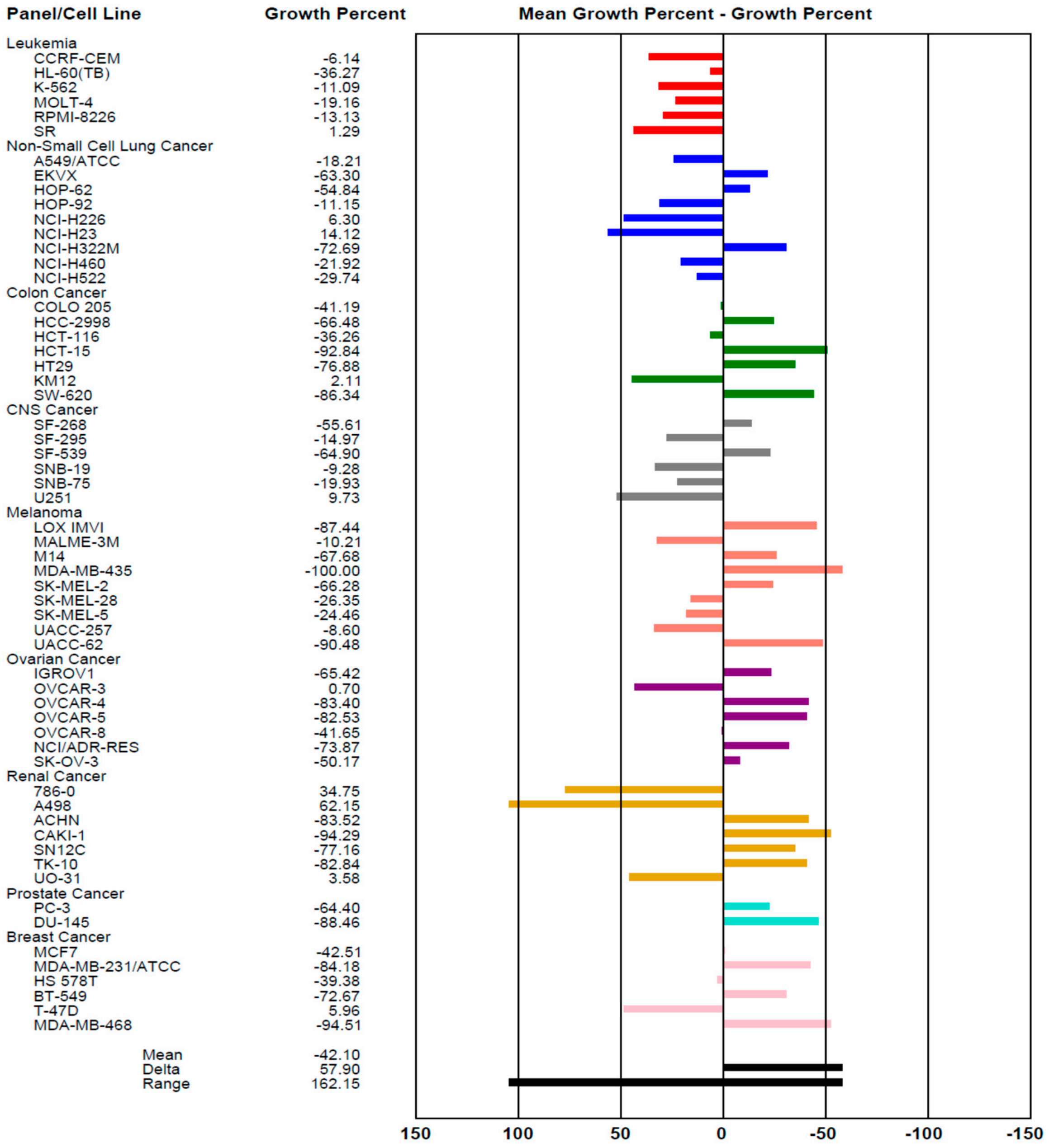

Fig. 62. One-dose growth (%) and mean graph of compound **7i**

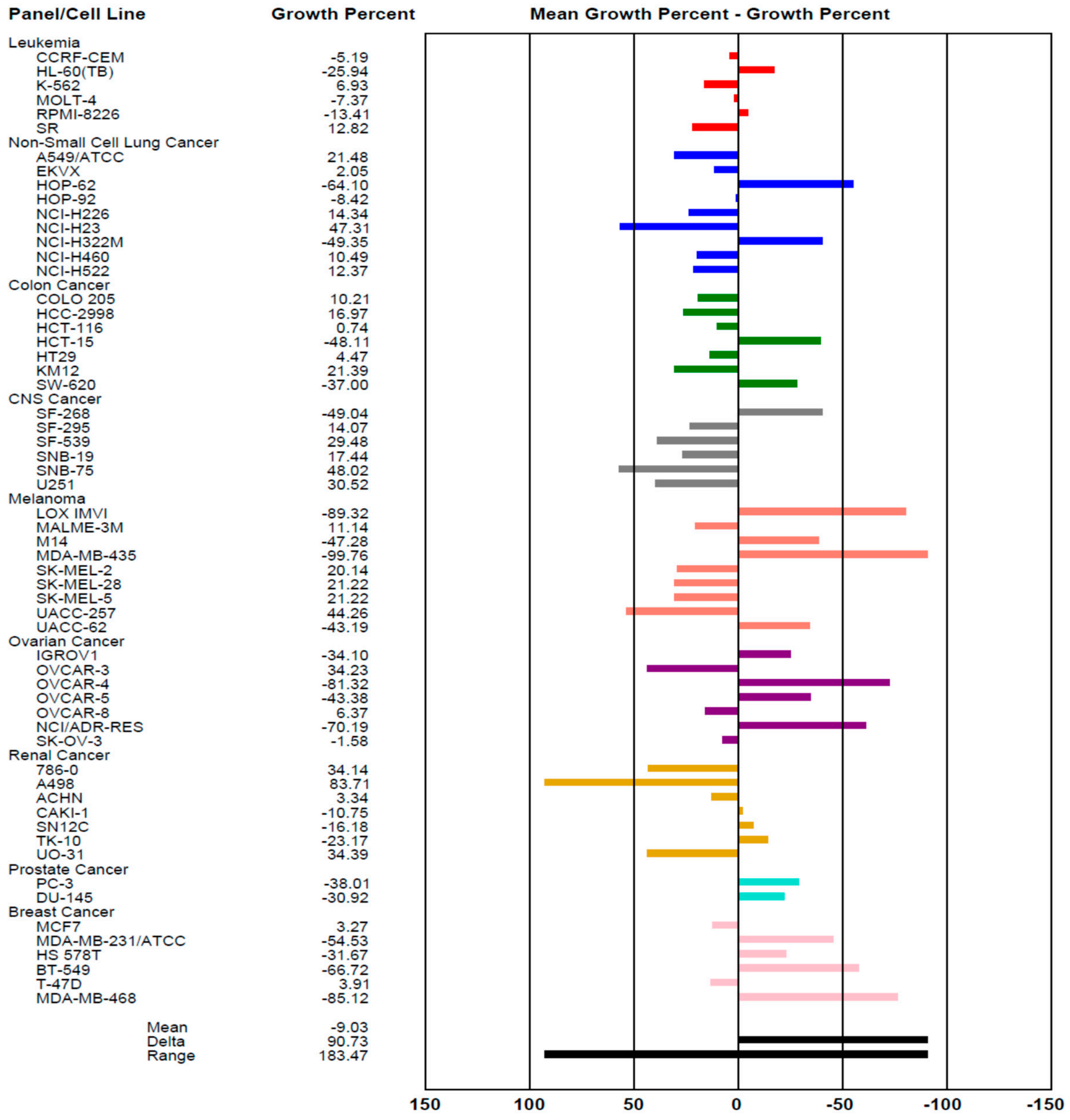

Fig. 63. One-dose growth (%) and mean graph of compound **7j**

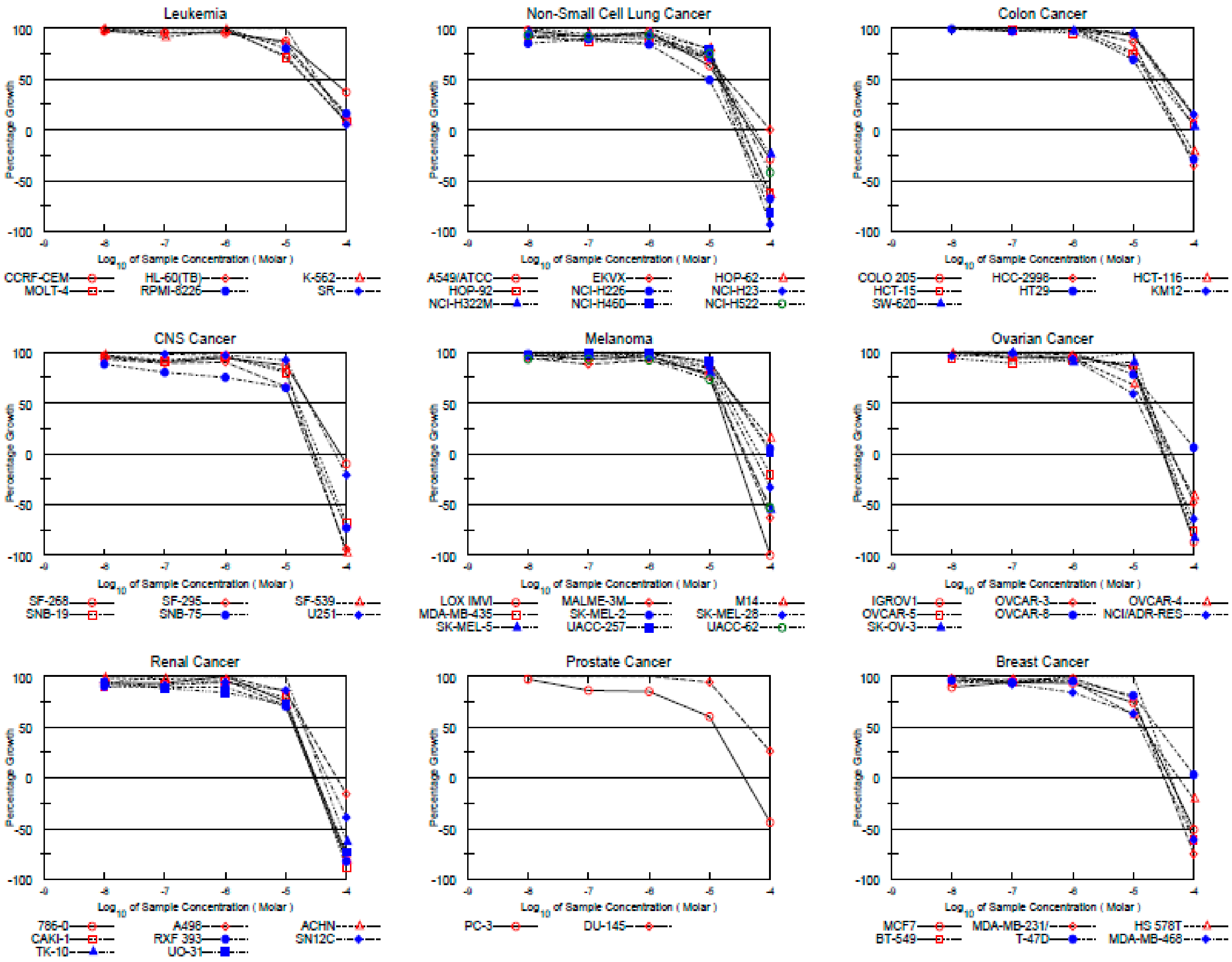

Fig. 64. Dose response curves for compound **6h**

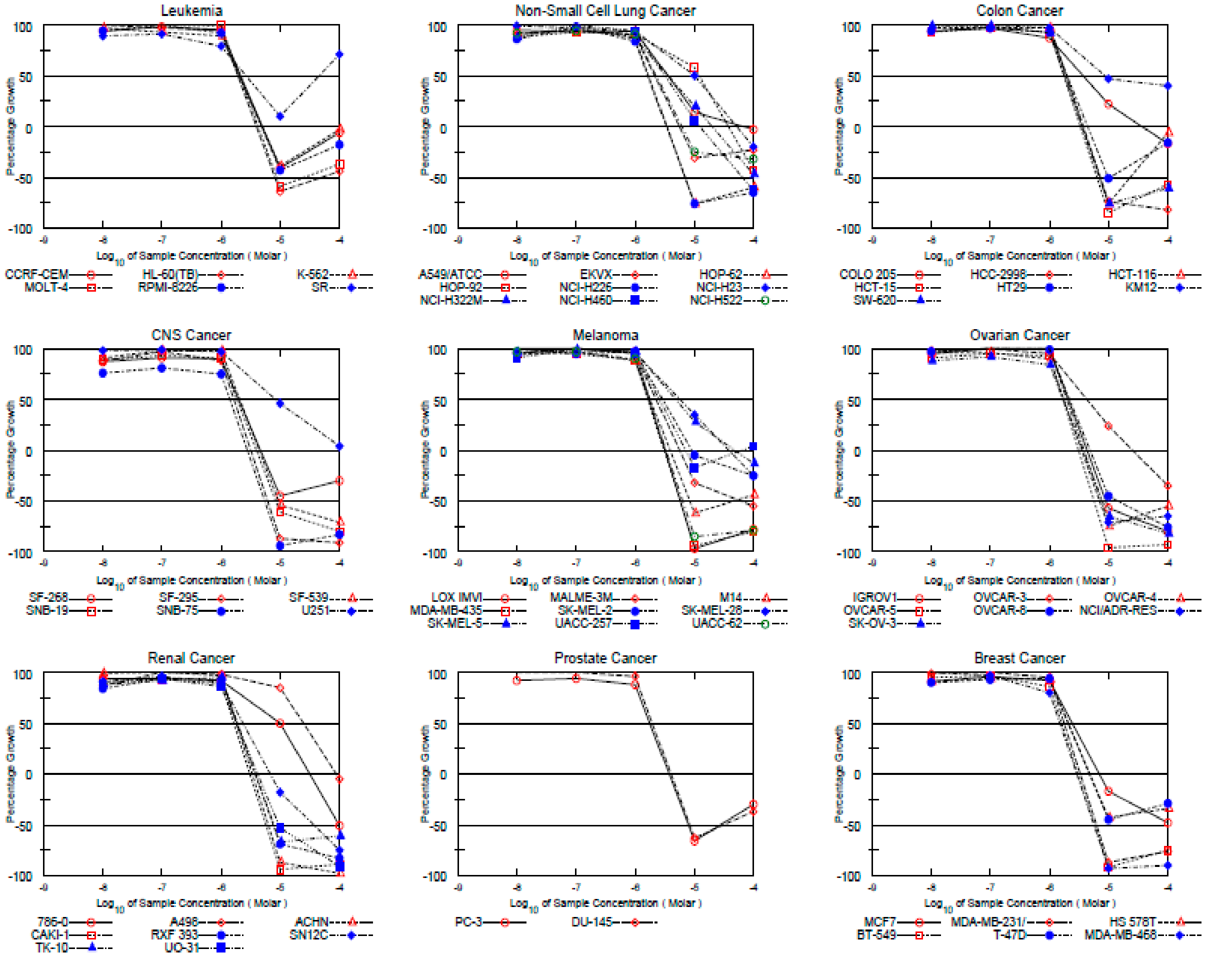

Fig. 65. Dose response curves for compound **7h**

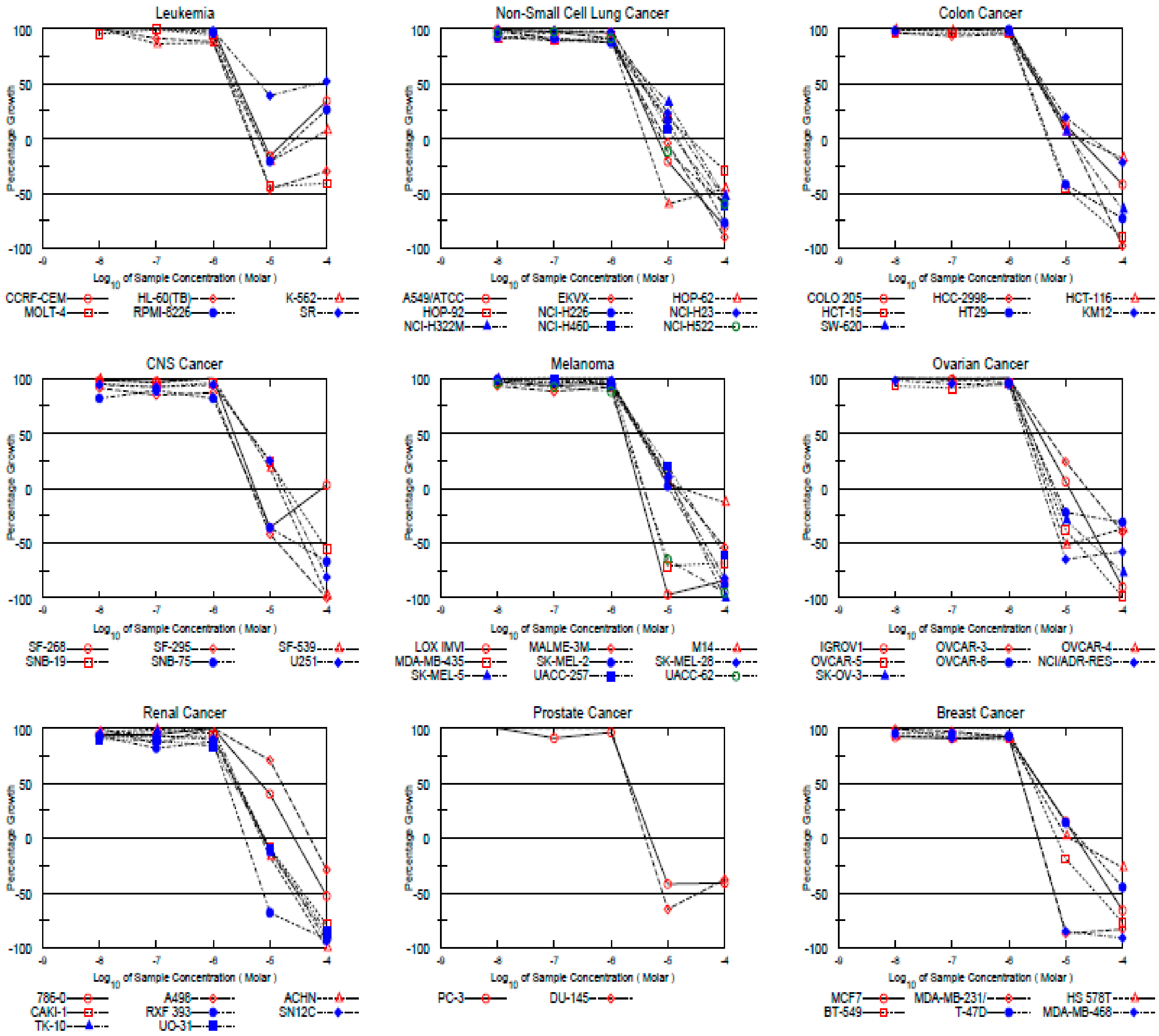

Fig. 66. Dose response curves for compound **7i**

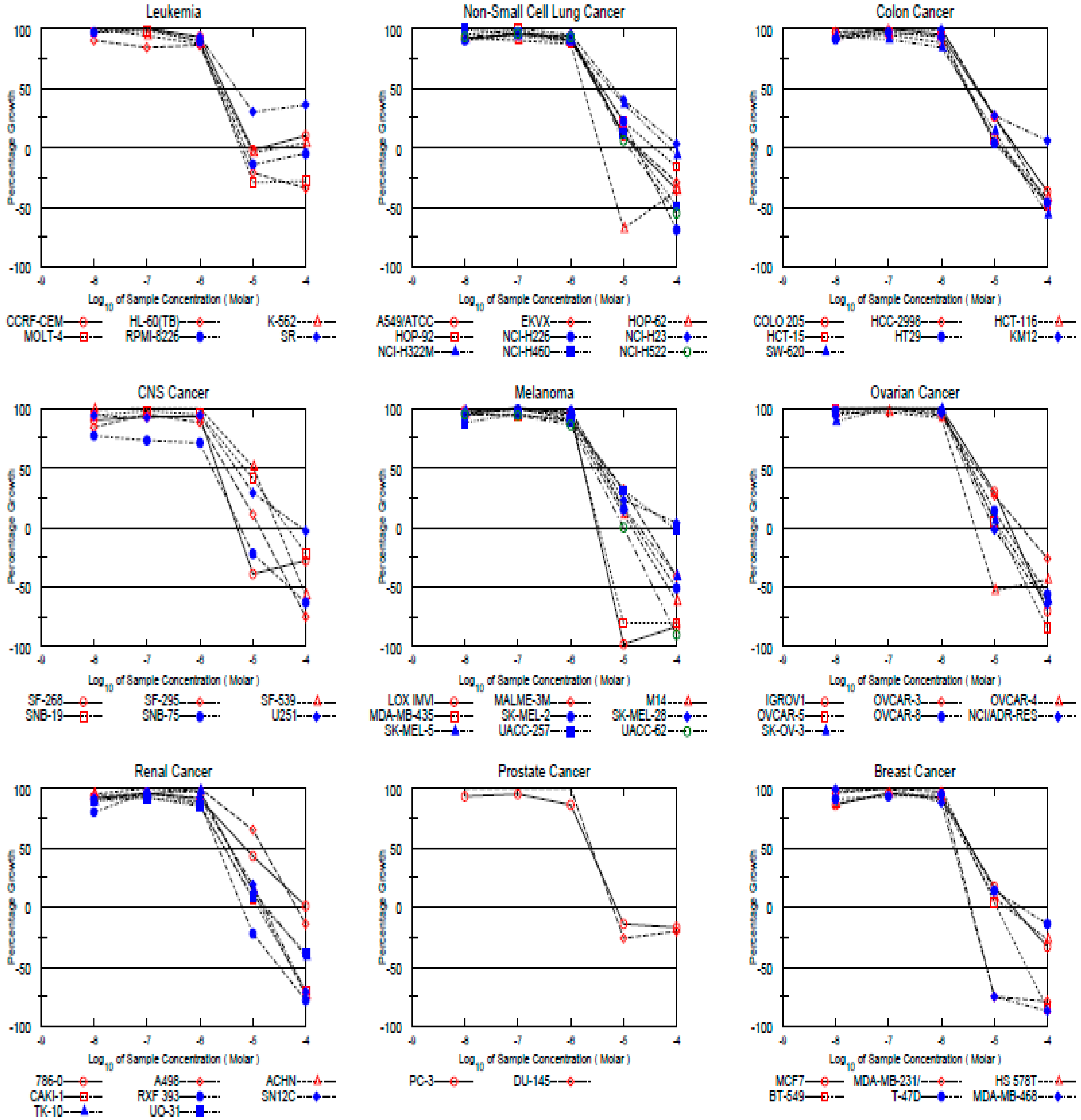

Fig.67. Dose response curves for compound **7j**

National Cancer Institute Developmental Therapeutics Program
In-Vitro Testing Results

NSC : D - 834249 / 1	Experiment ID : 2204NS06	Test Type : 08	Units : Molar
Report Date : June 08, 2022	Test Date : April 25, 2022	QNS :	MC :
COMI : me3	Stain Reagent : SRB Dual-Pass Related	SSPL : 0XUD	

	Time		Log10 Concentration: Mean Optical Densities					Percent Growth							
Panel/Cell Line	Zero	Ctrl	-8.0	-7.0	-6.0	-5.0	-4.0	-8.0	-7.0	-6.0	-5.0	-4.0	GI50	TGI	LC50
Leukemia															
CCRF-CEM	0.438	1.608	1.571	1.555	1.554	1.460	0.866	97	95	95	87	37	5.44E-5	> 1.00E-4	> 1.00E-4
HL-60(TB)	0.630	2.438	2.417	2.374	2.358	2.169	0.871	99	96	96	85	13	3.08E-5	> 1.00E-4	> 1.00E-4
K-562	0.212	2.029	1.986	1.873	1.992	1.533	0.338	98	91	98	73	7	2.21E-5	> 1.00E-4	> 1.00E-4
MOLT-4	0.668	2.468	2.472	2.523	2.562	1.952	0.819	100	103	105	71	8	2.18E-5	> 1.00E-4	> 1.00E-4
RPMI-8226	0.619	2.378	2.421	2.546	2.416	2.033	0.905	102	110	102	80	16	2.98E-5	> 1.00E-4	> 1.00E-4
SR	0.173	0.471	0.487	0.491	0.555	0.545	0.188	105	107	128	125	5	4.20E-5	> 1.00E-4	> 1.00E-4
Non-Small Cell Lung Cancer															
A549/ATCC	0.404	2.587	2.537	2.385	2.505	1.787	0.287	98	91	96	63	-29	1.39E-5	4.85E-5	> 1.00E-4
EKVX	0.576	2.038	1.905	1.882	1.887	1.591	0.574	91	89	90	69	0	1.90E-5	9.89E-5	> 1.00E-4
HOP-62	0.821	2.464	2.479	2.372	2.382	2.134	0.296	101	94	95	80	-64	1.61E-5	3.59E-5	8.00E-5
HOP-92	1.108	1.817	1.763	1.722	1.720	1.622	0.427	92	87	86	72	-62	1.47E-5	3.47E-5	8.21E-5
NCI-H226	0.719	1.588	1.458	1.489	1.453	1.143	0.228	85	89	84	49	-68	9.22E-6	2.61E-5	6.98E-5
NCI-H23	0.409	1.393	1.328	1.314	1.317	1.127	0.028	93	92	92	73	-93	1.37E-5	2.75E-5	5.49E-5
NCI-H322M	0.761	2.006	1.959	1.908	1.926	1.631	0.578	96	92	94	70	-24	1.63E-5	5.55E-5	> 1.00E-4
NCI-H460	0.290	2.699	2.867	2.845	2.727	2.197	0.053	107	106	101	79	-82	1.52E-5	3.10E-5	6.35E-5
NCI-H522	1.172	3.018	2.892	2.865	2.880	2.550	0.681	93	92	93	75	-42	1.63E-5	4.37E-5	> 1.00E-4
Colon Cancer															
COLO 205	0.551	2.190	2.231	2.209	2.262	2.072	0.764	103	101	104	93	13	3.44E-5	> 1.00E-4	> 1.00E-4
HCC-2998	0.488	1.851	1.936	1.803	1.878	1.664	0.319	106	97	102	86	-35	2.00E-5	5.17E-5	> 1.00E-4
HCT-116	0.239	2.212	2.269	2.312	2.291	1.752	0.188	103	105	104	77	-22	1.87E-5	6.04E-5	> 1.00E-4
HCT-15	0.337	2.338	2.372	2.292	2.234	1.836	0.429	102	98	95	75	5	2.26E-5	> 1.00E-4	> 1.00E-4
HT29	0.293	2.108	2.096	2.059	2.192	1.547	0.208	99	97	105	69	-29	1.56E-5	5.05E-5	> 1.00E-4
KM12	0.788	3.305	3.307	3.303	3.297	3.167	1.159	100	100	100	95	15	3.61E-5	> 1.00E-4	> 1.00E-4
SW-620	0.285	1.957	2.069	2.024	1.907	1.856	0.328	107	104	97	94	3	3.03E-5	> 1.00E-4	> 1.00E-4
CNS Cancer															
SF-268	1.015	2.854	2.785	2.665	2.743	2.623	0.914	96	90	94	87	-10	2.42E-5	7.89E-5	> 1.00E-4
SF-295	0.571	2.178	2.066	2.002	2.011	1.630	0.037	93	89	90	66	-94	1.26E-5	2.59E-5	5.33E-5
SF-539	0.761	2.286	2.238	2.163	2.229	2.008	0.017	97	92	96	82	-98	1.50E-5	2.85E-5	5.42E-5
SNB-19	0.644	2.115	2.023	1.983	2.037	1.814	0.205	94	91	95	80	-68	1.58E-5	3.45E-5	7.53E-5
SNB-75	1.353	2.355	2.231	2.157	2.103	2.007	0.363	88	80	75	65	-73	1.29E-5	2.96E-5	6.80E-5
U251	0.368	2.099	2.145	2.068	2.044	1.963	0.292	103	98	97	92	-21	2.36E-5	6.56E-5	> 1.00E-4
Melanoma															
LOX IMVI	0.264	2.121	2.073	1.988	2.037	1.699	-0.034	97	93	96	77	-100	1.43E-5	2.73E-5	5.22E-5
MALME-3M	0.737	1.476	1.430	1.387	1.416	1.330	0.270	94	88	92	80	-63	1.62E-5	3.62E-5	8.06E-5
M14	0.430	1.866	1.889	1.921	1.950	1.657	0.651	102	104	106	85	15	3.21E-5	> 1.00E-4	> 1.00E-4
MDA-MB-435	0.533	2.324	2.339	2.256	2.339	2.127	0.426	101	96	101	89	-20	2.28E-5	6.54E-5	> 1.00E-4
SK-MEL-2	1.302	3.108	3.080	3.111	3.046	2.953	1.394	98	100	97	91	5	3.02E-5	> 1.00E-4	> 1.00E-4
SK-MEL-28	0.782	2.046	1.999	2.002	1.983	1.850	0.521	96	96	95	84	-33	1.96E-5	5.21E-5	> 1.00E-4
SK-MEL-5	0.718	3.185	3.091	3.106	3.090	2.672	0.326	96	97	96	79	-55	1.65E-5	3.91E-5	9.24E-5
UACC-257	0.933	2.323	2.302	2.312	2.308	2.201	0.957	98	99	99	91	2	2.89E-5	> 1.00E-4	> 1.00E-4
UACC-62	0.894	2.495	2.387	2.388	2.373	2.065	0.423	93	93	92	73	-53	1.53E-5	3.81E-5	9.51E-5
Ovarian Cancer															
IGROV1	0.588	2.231	2.246	2.149	2.145	1.994	0.077	101	95	95	86	-87	1.61E-5	3.13E-5	6.10E-5
OVCAR-3	0.576	1.905	1.993	1.900	1.864	1.689	0.300	107	100	97	84	-48	1.80E-5	4.32E-5	> 1.00E-4
OVCAR-4	0.876	2.351	2.319	2.267	2.261	1.873	0.510	98	94	94	68	-42	1.45E-5	4.15E-5	> 1.00E-4
OVCAR-5	0.942	1.775	1.722	1.681	1.712	1.780	0.229	94	89	92	101	-76	1.94E-5	3.72E-5	7.15E-5
OVCAR-8	0.550	2.616	2.676	2.631	2.646	2.157	0.680	103	101	101	78	6	2.45E-5	> 1.00E-4	> 1.00E-4
NCI/ADR-RES	0.388	1.457	1.412	1.450	1.380	1.021	0.139	96	99	93	59	-64	1.19E-5	3.02E-5	7.68E-5
SK-OV-3	0.808	1.914	1.977	1.906	1.799	1.801	0.137	106	99	90	90	-83	1.70E-5	3.31E-5	6.44E-5
Renal Cancer															
786-0	0.541	2.253	2.142	2.133	2.254	1.833	0.124	93	93	100	75	-77	1.47E-5	3.12E-5	6.64E-5
A498	1.641	2.256	2.300	2.269	2.342	2.161	1.380	107	102	114	85	-16	2.21E-5	6.94E-5	> 1.00E-4
ACHN	0.530	2.111	2.086	2.060	2.047	1.653	0.099	98	97	96	71	-81	1.37E-5	2.92E-5	6.23E-5
CAKI-1	0.682	2.349	2.170	2.211	2.272	2.001	0.084	89	92	95	79	-88	1.49E-5	2.98E-5	5.94E-5
RXF 393	0.894	1.461	1.405	1.398	1.398	1.292	0.160	90	89	89	70	-82	1.36E-5	2.89E-5	6.15E-5
SN12C	0.748	2.665	2.553	2.492	2.541	2.396	0.459	94	91	94	86	-39	1.94E-5	4.89E-5	> 1.00E-4
TK-10	0.927	2.200	2.106	2.220	2.320	2.388	0.348	93	102	109	115	-63	2.32E-5	4.44E-5	8.50E-5
UO-31	0.877	2.646	2.514	2.428	2.356	2.149	0.238	93	88	84	72	-73	1.42E-5	3.14E-5	6.95E-5
Prostate Cancer															
PC-3	0.498	1.667	1.626	1.504	1.494	1.195	0.281	97	86	85	60	-44	1.24E-5	3.78E-5	> 1.00E-4
DU-145	0.459	2.019	2.078	2.077	2.084	1.925	0.865	104	104	104	94	26	4.43E-5	> 1.00E-4	> 1.00E-4
Breast Cancer															
MCF7	0.433	2.256	2.058	2.144	2.123	1.791	0.212	89	94	93	74	-51	1.57E-5	3.92E-5	9.81E-5
MDA-MB-231/ATCC	0.524	1.516	1.497	1.473	1.492	1.308	0.132	98	96	98	79	-75	1.54E-5	3.26E-5	6.90E-5
HS 578T	1.216	2.274	2.198	2.236	2.220	1.877	0.964	93	96	95	62	-21	1.41E-5	5.63E-5	> 1.00E-4
BT-549	1.308	1.914	1.892	1.877	1.929	2.058	0.510	96	94	102	124	-61	2.51E-5	4.67E-5	8.72E-5
T-47D	1.636	3.266	3.196	3.172	3.192	2.953	1.688	96	94	95	81	3	2.49E-5	> 1.00E-4	> 1.00E-4
MDA-MB-468	0.701	1.459	1.466	1.397	1.341	1.182	0.272	101	92	84	63	-61	1.28E-5	3.23E-5	8.13E-5

Fig.68. ***In- vitro*** **five dose analysis of compound 6h**

National Cancer Institute Developmental Therapeutics Program
In-Vitro Testing Results

NSC : D - 834254 / 1	Experiment ID : 2204NS06	Test Type : 08	Units : Molar
Report Date : June 08, 2022	Test Date : April 25, 2022	QNS :	MC :
COMI : me8	Stain Reagent : SRB Dual-Pass Related	SSPL : 0XUD	

	Log10 Concentration														
	Time		Mean Optical Densities					Percent Growth							
Panel/Cell Line	Zero	Ctrl	-8.0	-7.0	-6.0	-5.0	-4.0	-8.0	-7.0	-6.0	-5.0	-4.0	GI50	TGI	LC50
Leukemia															
CCRF-CEM	0.438	1.608	1.539	1.588	1.550	0.258	0.411	94	98	95	-41	-6	2.14E-6	4.98E-6	> 1.00E-4
HL-60(TB)	0.630	2.438	2.439	2.387	2.352	0.227	0.351	100	97	95	-64	-44	1.92E-6	3.96E-6	.
K-562	0.212	2.029	1.975	1.909	1.822	0.130	0.206	97	93	89	-39	-3	2.01E-6	4.97E-6	> 1.00E-4
MOLT-4	0.668	2.468	2.523	2.645	2.447	0.274	0.418	103	110	99	-59	-37	2.04E-6	4.23E-6	.
RPMI-8226	0.619	2.378	2.267	2.401	2.239	0.353	0.510	94	101	92	-43	-18	2.05E-6	4.81E-6	> 1.00E-4
SR	0.173	0.471	0.440	0.446	0.410	0.204	0.386	89	91	79	10	71	.	> 1.00E-4	> 1.00E-4
Non-Small Cell Lung Cancer															
A549/ATCC	0.404	2.587	2.474	2.425	2.457	0.710	0.394	95	93	94	14	-3	3.55E-6	6.97E-5	> 1.00E-4
EKVX	0.576	2.038	1.899	1.967	1.892	0.400	0.443	91	95	90	-31	-23	2.15E-6	5.58E-6	> 1.00E-4
HOP-62	0.821	2.464	2.323	2.402	2.252	0.197	0.329	91	96	87	-76	-60	1.69E-6	3.42E-6	6.93E-6
HOP-92	1.108	1.817	1.735	1.769	1.752	1.518	0.632	88	93	91	58	-43	1.19E-5	3.74E-5	> 1.00E-4
NCI-H226	0.719	1.588	1.467	1.572	1.448	0.176	0.249	86	98	84	-76	-65	1.63E-6	3.36E-6	6.91E-6
NCI-H23	0.409	1.393	1.380	1.361	1.331	0.904	0.328	99	97	94	50	-20	1.01E-5	5.22E-5	> 1.00E-4
NCI-H322M	0.761	2.006	1.853	2.010	1.840	1.008	0.403	88	100	87	20	-47	3.54E-6	1.98E-5	> 1.00E-4
NCI-H460	0.290	2.699	2.698	2.759	2.565	0.434	0.109	100	102	94	6	-63	3.18E-6	1.22E-5	6.55E-5
NCI-H522	1.172	3.018	2.869	2.902	2.843	0.874	0.792	92	94	91	-25	-32	2.24E-6	6.04E-6	> 1.00E-4
Colon Cancer															
COLO 205	0.551	2.190	2.094	2.139	1.985	0.918	0.459	94	97	87	22	-17	3.77E-6	3.74E-5	> 1.00E-4
HCC-2998	0.488	1.851	1.950	1.884	1.963	0.127	0.086	107	102	108	-74	-82	2.09E-6	3.93E-6	7.39E-6
HCT-116	0.239	2.212	2.161	2.161	2.152	0.059	0.225	97	97	97	-76	-6	1.87E-6	3.65E-6	.
HCT-15	0.337	2.338	2.198	2.270	2.201	0.051	0.144	93	97	93	-85	-57	1.75E-6	3.34E-6	6.37E-6
HT29	0.293	2.108	2.002	2.059	1.978	0.144	0.248	94	97	93	-51	-16	1.99E-6	4.43E-6	.
KM12	0.788	3.305	3.310	3.325	3.239	1.966	1.796	100	101	97	47	40	8.64E-6	> 1.00E-4	> 1.00E-4
SW-620	0.285	1.957	1.948	1.943	1.829	0.070	0.111	99	99	92	-76	-61	1.79E-6	3.55E-6	7.04E-6
CNS Cancer															
SF-268	1.015	2.854	2.608	2.684	2.675	0.561	0.706	87	91	90	-45	-30	1.99E-6	4.66E-6	> 1.00E-4
SF-295	0.571	2.178	1.985	2.131	1.981	0.075	0.050	88	97	88	-87	-91	1.64E-6	3.18E-6	6.15E-6
SF-539	0.761	2.286	2.152	2.255	2.250	0.351	0.220	91	98	98	-54	-71	2.06E-6	4.41E-6	9.43E-6
SNB-19	0.644	2.115	1.960	2.018	1.986	0.251	0.132	89	93	91	-61	-80	1.87E-6	3.97E-6	8.46E-6
SNB-75	1.353	2.355	2.112	2.165	2.100	0.081	0.234	76	81	75	-94	-83	1.40E-6	2.77E-6	5.48E-6
U251	0.368	2.099	2.061	2.073	2.048	1.158	0.432	98	99	97	46	4	8.22E-6	> 1.00E-4	> 1.00E-4
Melanoma															
LOX IMVI	0.264	2.121	2.165	2.183	2.074	0.008	0.059	102	103	97	-97	-78	1.75E-6	3.17E-6	5.73E-6
MALME-3M	0.737	1.476	1.444	1.486	1.440	0.499	0.330	96	101	95	-32	-55	2.26E-6	5.57E-6	5.91E-5
M14	0.430	1.866	1.783	1.823	1.694	0.163	0.240	94	97	88	-62	-44	1.79E-6	3.86E-6	.
MDA-MB-435	0.533	2.324	2.328	2.243	2.123	0.030	0.109	100	95	89	-94	-80	1.63E-6	3.05E-6	5.72E-6
SK-MEL-2	1.302	3.108	3.038	3.011	3.049	1.239	0.983	96	95	97	-5	-25	2.88E-6	8.96E-6	> 1.00E-4
SK-MEL-28	0.782	2.046	1.984	2.083	1.992	1.225	0.590	95	103	96	35	-25	5.67E-6	3.87E-5	> 1.00E-4
SK-MEL-5	0.718	3.185	3.089	3.149	3.110	1.401	0.626	96	99	97	28	-13	4.76E-6	4.81E-5	> 1.00E-4
UACC-257	0.933	2.323	2.193	2.357	2.271	0.778	0.987	91	102	96	-17	4	2.57E-6	.	> 1.00E-4
UACC-62	0.894	2.495	2.453	2.455	2.330	0.135	0.185	97	97	90	-85	-79	1.69E-6	3.26E-6	6.31E-6
Ovarian Cancer															
IGROV1	0.588	2.231	2.150	2.280	2.233	0.255	0.116	95	103	100	-57	-80	2.09E-6	4.35E-6	9.07E-6
OVCAR-3	0.576	1.905	1.875	1.994	1.834	0.892	0.377	98	107	95	24	-35	4.26E-6	2.55E-5	> 1.00E-4
OVCAR-4	0.876	2.351	2.297	2.297	2.203	0.218	0.390	96	96	90	-75	-55	1.75E-6	3.51E-6	7.04E-6
OVCAR-5	0.942	1.775	1.697	1.736	1.716	0.041	0.070	91	95	93	-96	-93	1.69E-6	3.11E-6	5.73E-6
OVCAR-8	0.550	2.616	2.554	2.711	2.594	0.305	0.133	97	105	99	-45	-76	2.19E-6	4.89E-6	1.49E-5
NCI/ADR-RES	0.388	1.457	1.421	1.470	1.481	0.114	0.136	97	101	102	-71	-65	2.00E-6	3.90E-6	7.59E-6
SK-OV-3	0.808	1.914	1.778	1.828	1.732	0.280	0.146	88	92	84	-65	-82	1.68E-6	3.64E-6	7.89E-6
Renal Cancer															
786-0	0.541	2.253	2.159	2.153	2.112	1.400	0.266	94	94	92	50	-51	1.00E-5	3.14E-5	9.81E-5
A498	1.641	2.256	2.173	2.262	2.247	2.167	1.567	87	101	98	85	-5	2.48E-5	8.91E-5	> 1.00E-4
ACHN	0.530	2.111	2.093	2.196	2.057	0.067	0.008	99	105	97	-87	-98	1.79E-6	3.35E-6	6.26E-6
CAKI-1	0.682	2.349	2.194	2.240	2.167	0.040	0.078	91	93	89	-94	-89	1.63E-6	3.06E-6	5.74E-6
RXF 393	0.894	1.461	1.371	1.429	1.422	0.279	0.149	84	94	93	-69	-83	1.85E-6	3.76E-6	7.65E-6
SN12C	0.748	2.665	2.496	2.586	2.465	0.614	0.190	91	96	90	-18	-75	2.33E-6	6.80E-6	3.67E-5
TK-10	0.927	2.200	2.053	2.098	2.134	0.311	0.358	88	92	95	-67	-61	1.90E-6	3.87E-6	7.90E-6
UO-31	0.877	2.646	2.456	2.545	2.406	0.410	0.081	89	94	86	-53	-91	1.82E-6	4.15E-6	9.47E-6
Prostate Cancer															
PC-3	0.498	1.667	1.574	1.592	1.529	0.171	0.351	92	94	88	-66	-30	1.77E-6	3.74E-6	.
DU-145	0.459	2.019	2.040	2.060	1.957	0.169	0.288	101	103	96	-63	-37	1.94E-6	4.01E-6	.
Breast Cancer															
MCF7	0.433	2.256	2.084	2.181	2.104	0.361	0.226	91	96	92	-17	-48	2.42E-6	7.01E-6	> 1.00E-4
MDA-MB-231/ATCC	0.524	1.516	1.503	1.570	1.467	0.069	0.128	99	105	95	-87	-76	1.77E-6	3.33E-6	6.27E-6
HS 578T	1.216	2.274	2.180	2.209	2.202	0.689	0.802	91	94	93	-43	-34	2.07E-6	4.82E-6	> 1.00E-4
BT-549	1.308	1.914	1.885	1.894	1.827	0.099	0.328	95	97	86	-92	-75	1.59E-6	3.03E-6	5.78E-6
T-47D	1.636	3.266	3.109	3.144	3.167	0.902	1.158	90	93	94	-45	-29	2.07E-6	4.75E-6	> 1.00E-4
MDA-MB-468	0.701	1.459	1.472	1.437	1.311	0.048	0.070	102	97	80	-93	-90	1.50E-6	2.91E-6	5.64E-6

Fig.69. ***In- vitro*** **five dose analysis of compound 7h**

National Cancer Institute Developmental Therapeutics Program
In-Vitro Testing Results

NSC : D - 834255 / 1	Experiment ID : 2204NS06	Test Type : 08	Units : Molar
Report Date : June 08, 2022	Test Date : April 25, 2022	QNS :	MC :
COMI : me9	Stain Reagent : SRB Dual-Pass Related	SSPL : 0XUD	

			Log10 Concentration												
	Time		Mean Optical Densities					Percent Growth							
Panel/Cell Line	Zero	Ctrl	-8.0	-7.0	-6.0	-5.0	-4.0	-8.0	-7.0	-6.0	-5.0	-4.0	GI50	TGI	LC50
**Leukemia**															
CCRF-CEM	0.438	1.786	1.827	1.780	1.822	0.368	0.894	103	100	103	-16	34	2.78E-6	.	> 1.00E-4
HL-60(TB)	0.630	2.616	2.676	2.441	2.374	0.343	0.441	103	91	88	-46	-30	1.92E-6	4.55E-6	> 1.00E-4
K-562	0.212	2.159	2.161	1.887	1.915	0.168	0.340	100	86	87	-21	7	2.22E-6	.	> 1.00E-4
MOLT-4	0.668	2.694	2.598	2.669	2.577	0.374	0.392	95	99	94	-44	-41	2.09E-6	4.80E-6	> 1.00E-4
RPMI-8226	0.619	2.592	2.630	2.637	2.505	0.487	1.139	102	102	96	-21	26	2.45E-6	.	> 1.00E-4
SR	0.173	0.549	0.639	0.638	0.541	0.320	0.368	124	123	98	39	52	.	> 1.00E-4	> 1.00E-4
**Non-Small Cell Lung Cancer**															
A549/ATCC	0.404	2.409	2.380	2.345	2.338	0.321	0.080	99	97	96	-21	-80	2.49E-6	6.66E-6	3.10E-5
EKVX	0.576	2.060	1.946	1.896	1.886	0.554	0.056	92	89	88	-4	-90	2.60E-6	9.09E-6	3.42E-5
HOP-62	0.821	2.535	2.359	2.490	2.373	0.326	0.441	90	97	91	-60	-46	1.86E-6	3.98E-6	.
HOP-92	1.108	2.002	2.021	1.906	1.923	1.270	0.785	102	89	91	18	-29	3.66E-6	2.41E-5	> 1.00E-4
NCI-H226	0.719	1.552	1.487	1.481	1.445	0.853	0.167	92	91	87	16	-77	3.33E-6	1.49E-5	5.14E-5
NCI-H23	0.409	1.397	1.374	1.380	1.365	0.638	0.181	98	98	97	23	-56	4.31E-6	1.96E-5	8.43E-5
NCI-H322M	0.761	2.039	2.004	1.929	1.873	1.179	0.361	97	91	87	33	-53	4.80E-6	2.42E-5	9.33E-5
NCI-H460	0.290	2.908	3.138	3.132	2.946	0.488	0.112	109	109	101	8	-61	3.53E-6	1.29E-5	6.84E-5
NCI-H522	1.172	2.937	2.865	2.882	2.786	1.035	0.467	96	97	91	-12	-60	2.52E-6	7.70E-6	6.16E-5
**Colon Cancer**															
COLO 205	0.551	2.230	2.273	2.291	2.276	0.750	0.321	103	104	103	12	-42	3.80E-6	1.66E-5	> 1.00E-4
HCC-2998	0.488	1.952	1.898	1.855	1.876	0.630	0.010	96	93	95	10	-98	3.36E-6	1.23E-5	3.58E-5
HCT-116	0.239	2.410	2.378	2.356	2.446	0.361	0.197	99	98	102	6	-18	3.45E-6	1.74E-5	> 1.00E-4
HCT-15	0.337	2.372	2.296	2.300	2.293	0.181	0.033	96	96	96	-46	-90	2.11E-6	4.72E-6	1.21E-5
HT29	0.293	2.017	1.975	2.035	2.002	0.170	0.078	98	101	99	-42	-73	2.23E-6	5.03E-6	1.78E-5
KM12	0.788	3.303	3.326	3.292	3.316	1.260	0.619	101	100	101	19	-22	4.15E-6	2.92E-5	> 1.00E-4
SW-620	0.285	2.172	2.230	2.292	2.118	0.396	0.101	103	106	97	6	-65	3.28E-6	1.21E-5	6.21E-5
**CNS Cancer**															
SF-268	1.015	2.855	2.823	2.797	2.886	0.647	1.074	98	97	102	-36	3	2.37E-6	.	> 1.00E-4
SF-295	0.571	2.237	2.094	1.987	2.021	0.329	-0.003	91	85	87	-42	-100	1.93E-6	4.70E-6	1.36E-5
SF-539	0.761	2.327	2.308	2.268	2.324	1.038	0.015	99	96	100	18	-98	4.04E-6	1.42E-5	3.84E-5
SNB-19	0.644	2.164	2.089	2.038	2.108	1.002	0.285	95	92	96	24	-56	4.32E-6	1.98E-5	8.45E-5
SNB-75	1.353	2.551	2.333	2.417	2.339	0.863	0.449	82	89	82	-36	-67	1.87E-6	4.95E-6	2.81E-5
U251	0.368	1.905	1.812	1.782	1.811	0.759	0.070	94	92	94	25	-81	4.38E-6	1.73E-5	5.12E-5
**Melanoma**															
LOX IMVI	0.264	2.225	2.158	2.088	2.120	0.008	0.043	97	93	95	-97	-84	1.71E-6	3.12E-6	5.69E-6
MALME-3M	0.737	1.509	1.457	1.414	1.444	0.813	0.340	93	88	92	10	-54	3.22E-6	1.43E-5	8.70E-5
M14	0.430	1.964	1.943	1.998	1.996	0.493	0.376	99	102	102	4	-13	3.40E-6	1.76E-5	> 1.00E-4
MDA-MB-435	0.533	2.595	2.799	2.666	2.645	0.155	0.168	110	103	102	-71	-68	2.01E-6	3.89E-6	7.57E-6
SK-MEL-2	1.302	3.109	3.085	3.090	3.050	1.331	0.152	99	99	97	2	-88	3.10E-6	1.04E-5	3.75E-5
SK-MEL-28	0.782	2.091	2.084	2.029	2.049	0.897	0.145	99	95	97	9	-82	3.40E-6	1.25E-5	4.48E-5
SK-MEL-5	0.718	3.208	3.180	3.114	3.123	1.014	0.001	99	96	97	12	-100	3.55E-6	1.28E-5	3.58E-5
UACC-257	0.933	2.237	2.217	2.220	2.150	1.190	0.362	98	99	93	20	-61	3.87E-6	1.75E-5	7.26E-5
UACC-62	0.894	2.631	2.539	2.494	2.420	0.309	0.041	95	92	88	-65	-95	1.77E-6	3.74E-6	7.93E-6
**Ovarian Cancer**															
IGROV1	0.588	2.243	2.303	2.220	2.237	0.695	0.057	104	99	100	6	-90	3.41E-6	1.17E-5	3.83E-5
OVCAR-3	0.576	1.973	2.075	2.104	2.101	0.916	0.345	107	109	109	24	-40	4.98E-6	2.38E-5	> 1.00E-4
OVCAR-4	0.876	2.411	2.437	2.378	2.417	0.424	0.555	102	98	100	-52	-37	2.14E-6	4.57E-6	.
OVCAR-5	0.942	1.769	1.714	1.692	1.728	0.588	0.005	93	91	95	-38	-99	2.18E-6	5.21E-6	1.59E-5
OVCAR-8	0.550	2.557	2.553	2.596	2.478	0.429	0.378	100	102	96	-22	-31	2.45E-6	6.50E-6	> 1.00E-4
NCI/ADR-RES	0.388	1.483	1.460	1.429	1.419	0.137	0.163	98	95	94	-65	-58	1.90E-6	3.92E-6	8.08E-6
SK-OV-3	0.808	1.846	1.873	1.843	1.881	0.573	0.187	103	100	103	-29	-77	2.53E-6	6.03E-6	2.74E-5
**Renal Cancer**															
786-0	0.541	2.385	2.272	2.280	2.386	1.281	0.256	94	94	100	40	-53	6.85E-6	2.70E-5	9.34E-5
A498	1.641	2.251	2.260	2.166	2.255	2.075	1.172	101	86	101	71	-29	1.63E-5	5.17E-5	> 1.00E-4
ACHN	0.530	2.204	2.130	2.188	2.127	0.440	-0.007	96	99	95	-17	-100	2.53E-6	7.05E-6	2.49E-5
CAKI-1	0.682	2.479	2.340	2.336	2.355	0.618	0.141	92	92	93	-9	-79	2.63E-6	8.10E-6	3.80E-5
RXF 393	0.894	1.524	1.478	1.408	1.446	0.285	0.074	93	82	88	-68	-92	1.75E-6	3.65E-6	7.65E-6
SN12C	0.748	2.757	2.609	2.609	2.558	0.675	0.048	93	93	90	-10	-94	2.52E-6	7.97E-6	3.01E-5
TK-10	0.927	2.101	2.029	2.068	2.119	0.802	0.101	94	97	101	-13	-89	2.80E-6	7.63E-6	3.04E-5
UO-31	0.877	2.716	2.531	2.521	2.415	0.789	0.135	90	89	84	-10	-85	2.29E-6	7.81E-6	3.43E-5
**Prostate Cancer**															
PC-3	0.498	1.893	1.892	1.769	1.835	0.290	0.292	100	91	96	-42	-41	2.15E-6	4.96E-6	> 1.00E-4
DU-145	0.459	1.972	2.041	1.984	2.000	0.161	0.284	105	101	102	-65	-38	2.05E-6	4.08E-6	.
**Breast Cancer**															
MCF7	0.433	2.318	2.172	2.152	2.163	0.712	0.148	92	91	92	15	-66	3.49E-6	1.53E-5	6.36E-5
MDA-MB-231/ATCC	0.524	1.538	1.526	1.510	1.453	0.069	0.090	99	97	92	-87	-83	1.71E-6	3.26E-6	6.21E-6
HS 578T	1.216	2.366	2.286	2.266	2.248	1.241	0.887	93	91	90	2	-27	2.84E-6	1.19E-5	> 1.00E-4
BT-549	1.308	1.958	1.997	2.052	2.084	1.054	0.305	106	114	119	-19	-77	3.16E-6	7.25E-6	3.42E-5
T-47D	1.636	3.296	3.211	3.207	3.186	1.867	0.898	95	95	93	14	-45	3.51E-6	1.72E-5	> 1.00E-4
MDA-MB-468	0.701	1.463	1.476	1.396	1.401	0.104	0.063	102	91	92	-85	-91	1.72E-6	3.30E-6	6.32E-6

Fig.70. ***In- vitro*** **five dose analysis of compound 7i**

National Cancer Institute Developmental Therapeutics Program
In-Vitro Testing Results

NSC : D - 834256 / 1	Experiment ID : 2204NS06	Test Type : 08	Units : Molar
Report Date : June 08, 2022	Test Date : April 25, 2022	QNS :	MC :
COMI : me10	Stain Reagent : SRB Dual-Pass Related	SSPL : 0XUD	

			Log10 Concentration												
	Time		Mean Optical Densities					Percent Growth							
Panel/Cell Line	Zero	Ctrl	-8.0	-7.0	-6.0	-5.0	-4.0	-8.0	-7.0	-6.0	-5.0	-4.0	GI50	TGI	LC50
**Leukemia**															
CCRF-CEM	0.438	1.786	1.815	1.796	1.693	0.431	0.570	102	101	93	-2	10	2.85E-6	.	> 1.00E-4
HL-60(TB)	0.630	2.616	2.422	2.308	2.342	0.496	0.417	90	84	86	-21	-34	2.17E-6	6.34E-6	> 1.00E-4
K-562	0.212	2.159	2.175	2.048	1.912	0.203	0.291	101	94	87	-4	4	2.55E-6	.	> 1.00E-4
MOLT-4	0.668	2.694	2.638	2.659	2.484	0.474	0.478	97	98	90	-29	-28	2.16E-6	5.69E-6	> 1.00E-4
RPMI-8226	0.619	2.592	2.534	2.627	2.371	0.532	0.587	97	102	89	-14	-5	2.38E-6	7.29E-6	> 1.00E-4
SR	0.173	0.549	0.570	0.578	0.522	0.284	0.309	106	108	93	30	36	4.74E-6	> 1.00E-4	> 1.00E-4
**Non-Small Cell Lung Cancer**															
A549/ATCC	0.404	2.409	2.242	2.300	2.286	0.610	0.261	92	95	94	10	-35	3.35E-6	1.68E-5	> 1.00E-4
EKVX	0.576	2.060	1.928	2.000	1.905	0.745	0.410	91	96	90	11	-29	3.21E-6	1.92E-5	> 1.00E-4
HOP-62	0.821	2.535	2.403	2.364	2.318	0.264	0.525	92	90	87	-68	-36	1.74E-6	3.65E-6	.
HOP-92	1.108	2.002	1.954	1.998	1.907	1.305	0.928	95	99	89	22	-16	3.84E-6	3.76E-5	> 1.00E-4
NCI-H226	0.719	1.552	1.469	1.523	1.481	0.906	0.222	90	97	91	22	-69	3.99E-6	1.76E-5	6.18E-5
NCI-H23	0.409	1.397	1.420	1.429	1.347	0.802	0.435	102	103	95	40	3	6.52E-6	> 1.00E-4	> 1.00E-4
NCI-H322M	0.761	2.039	1.918	1.960	1.897	1.240	0.717	91	94	89	37	-6	5.71E-6	7.33E-5	> 1.00E-4
NCI-H460	0.290	2.908	2.888	2.813	2.692	0.603	0.148	99	96	92	12	-49	3.33E-6	1.57E-5	> 1.00E-4
NCI-H522	1.172	2.937	2.819	2.864	2.808	1.278	0.523	93	96	93	6	-55	3.11E-6	1.25E-5	8.17E-5
**Colon Cancer**															
COLO 205	0.551	2.230	2.120	2.239	2.142	0.987	0.349	93	101	95	26	-37	4.47E-6	2.59E-5	> 1.00E-4
HCC-2998	0.488	1.952	1.906	1.942	1.964	0.863	0.259	97	99	101	26	-47	4.74E-6	2.25E-5	> 1.00E-4
HCT-116	0.239	2.410	2.250	2.306	2.147	0.383	0.139	93	95	88	7	-42	2.92E-6	1.37E-5	> 1.00E-4
HCT-15	0.337	2.372	2.297	2.312	2.244	0.485	0.177	96	97	94	7	-48	3.20E-6	1.36E-5	> 1.00E-4
HT29	0.293	2.017	1.869	1.959	1.902	0.356	0.159	91	97	93	4	-46	3.04E-6	1.18E-5	> 1.00E-4
KM12	0.788	3.303	3.313	3.320	3.266	1.456	0.944	100	101	99	27	6	4.72E-6	> 1.00E-4	> 1.00E-4
SW-620	0.285	2.172	2.046	2.002	1.869	0.559	0.127	93	91	84	14	-56	3.08E-6	1.61E-5	8.32E-5
**CNS Cancer**															
SF-268	1.015	2.855	2.663	2.721	2.720	0.624	0.735	90	93	93	-39	-28	2.11E-6	5.08E-6	> 1.00E-4
SF-295	0.571	2.237	1.969	2.159	2.035	0.754	0.141	84	95	88	11	-75	3.11E-6	1.34E-5	5.08E-5
SF-539	0.761	2.327	2.306	2.389	2.331	1.557	0.325	99	104	100	51	-57	1.02E-5	2.95E-5	8.56E-5
SNB-19	0.644	2.164	2.072	2.127	2.090	1.272	0.504	94	98	95	41	-22	6.89E-6	4.52E-5	> 1.00E-4
SNB-75	1.353	2.551	2.281	2.232	2.201	1.056	0.501	77	73	71	-22	-63	1.68E-6	5.80E-6	4.82E-5
U251	0.368	1.905	1.809	1.777	1.813	0.810	0.358	94	92	94	29	-3	4.73E-6	8.12E-5	> 1.00E-4
**Melanoma**															
LOX IMVI	0.264	2.225	2.177	2.236	2.142	0.005	0.045	98	101	96	-98	-83	1.72E-6	3.12E-6	5.65E-6
MALME-3M	0.737	1.509	1.467	1.512	1.438	0.987	0.433	95	100	91	32	-41	4.99E-6	2.75E-5	> 1.00E-4
M14	0.430	1.964	1.890	1.893	1.794	0.598	0.164	95	95	89	11	-62	3.15E-6	1.41E-5	6.87E-5
MDA-MB-435	0.533	2.595	2.497	2.468	2.406	0.109	0.105	95	94	91	-80	-80	1.74E-6	3.41E-6	6.71E-6
SK-MEL-2	1.302	3.109	3.038	3.096	3.005	1.581	0.638	96	99	94	15	-51	3.64E-6	1.71E-5	9.65E-5
SK-MEL-28	0.782	2.091	2.038	2.169	2.069	1.087	0.836	96	106	98	23	4	4.41E-6	> 1.00E-4	> 1.00E-4
SK-MEL-5	0.718	3.208	3.144	3.210	3.141	1.139	0.422	97	100	97	17	-41	3.87E-6	1.95E-5	> 1.00E-4
UACC-257	0.933	2.237	2.080	2.177	2.098	1.331	0.916	88	95	89	31	-2	4.67E-6	8.78E-5	> 1.00E-4
UACC-62	0.894	2.631	2.540	2.535	2.384	0.899	0.094	95	94	86	0	-90	2.62E-6	1.01E-5	3.63E-5
**Ovarian Cancer**															
IGROV1	0.588	2.243	2.268	2.307	2.313	1.081	0.175	102	104	104	30	-70	5.35E-6	1.98E-5	6.27E-5
OVCAR-3	0.576	1.973	1.990	1.986	1.888	0.960	0.425	101	101	94	27	-26	4.58E-6	3.24E-5	> 1.00E-4
OVCAR-4	0.876	2.411	2.347	2.372	2.294	0.412	0.492	96	97	92	-53	-44	1.96E-6	4.32E-6	.
OVCAR-5	0.942	1.769	1.762	1.798	1.770	0.984	0.148	99	104	100	5	-84	3.37E-6	1.14E-5	4.13E-5
OVCAR-8	0.550	2.557	2.523	2.612	2.519	0.827	0.242	98	103	98	14	-56	3.72E-6	1.58E-5	8.18E-5
NCI/ADR-RES	0.388	1.483	1.419	1.501	1.441	0.380	0.139	94	102	96	-2	-64	2.95E-6	9.50E-6	5.91E-5
SK-OV-3	0.808	1.846	1.732	1.879	1.834	0.875	0.312	89	103	99	6	-61	3.38E-6	1.24E-5	6.78E-5
**Renal Cancer**															
786-0	0.541	2.385	2.216	2.304	2.228	1.341	0.557	91	96	91	43	1	7.29E-6	> 1.00E-4	> 1.00E-4
A498	1.641	2.251	2.212	2.221	2.258	2.037	1.405	94	95	101	65	-14	1.54E-5	6.58E-5	> 1.00E-4
ACHN	0.530	2.204	2.115	2.209	2.151	0.767	0.137	95	100	97	14	-74	3.69E-6	1.45E-5	5.33E-5
CAKI-1	0.682	2.479	2.332	2.330	2.251	0.814	0.203	92	92	87	7	-70	2.93E-6	1.24E-5	5.48E-5
RXF 393	0.894	1.524	1.399	1.497	1.451	0.695	0.194	80	96	88	-22	-78	2.22E-6	6.29E-6	3.12E-5
SN12C	0.748	2.757	2.569	2.665	2.604	1.133	0.216	91	95	92	19	-71	3.79E-6	1.63E-5	5.83E-5
TK-10	0.927	2.101	1.970	2.044	2.075	1.088	0.544	89	95	98	14	-41	3.70E-6	1.77E-5	> 1.00E-4
UO-31	0.877	2.716	2.515	2.577	2.437	1.045	0.547	89	92	85	9	-38	2.88E-6	1.57E-5	> 1.00E-4
**Prostate Cancer**															
PC-3	0.498	1.893	1.795	1.825	1.702	0.430	0.412	93	95	86	-14	-17	2.31E-6	7.29E-6	> 1.00E-4
DU-145	0.459	1.972	2.000	2.092	2.022	0.341	0.368	102	108	103	-26	-20	2.59E-6	6.32E-6	> 1.00E-4
**Breast Cancer**															
MCF7	0.433	2.318	2.049	2.241	2.174	0.747	0.290	86	96	92	17	-33	3.63E-6	2.16E-5	> 1.00E-4
MDA-MB-231/ATCC	0.524	1.538	1.497	1.559	1.504	0.132	0.109	96	102	97	-75	-79	1.87E-6	3.66E-6	7.17E-6
HS 578T	1.216	2.366	2.212	2.304	2.257	1.306	0.883	87	95	91	8	-27	3.09E-6	1.66E-5	> 1.00E-4
BT-549	1.308	1.958	1.936	1.965	2.030	1.333	0.202	97	101	111	4	-85	3.71E-6	1.11E-5	4.07E-5
T-47D	1.636	3.296	3.151	3.187	3.216	1.867	1.407	91	93	95	14	-14	3.60E-6	3.14E-5	> 1.00E-4
MDA-MB-468	0.701	1.463	1.453	1.462	1.375	0.175	0.089	99	100	88	-75	-87	1.72E-6	3.47E-6	7.02E-6

Fig.71. ***In- vitro*** **five dose analysis of compound 7j**

No	Compound	M.Wt	Tub. (IC_{50})	
	code	(g/mol)	IC_{50}±SD (µg/ml)	IC_{50} (µM) ≡ (µmol/L)
1	22 (7i)	504.40	1.53±0.06	3.03±0.11
3	29 (7j)	459.95	2.88±0.09	6.26±0.15
9	36 (6h)	424.52	4.039±0.15	9.50±0.30
10	37 (7h)	439.52	8.075±0.3	18.37±0.70
11	CA4	334.4	2.786±0.1	8.33±0.29

Tub. enzyme inhibition assay												
	Conc.		Optical density			Temperature						
code	conc	Log Conc.	RFU2	RFU1	ΔRFU	T2	T1	ΔT	slope	%inhibition	K. Activity	IC50 (µg/ml)
7i	100	2	3686	0	3686	30	0	30	948.4	87	15.546	1.53±0.06
	10	1	8966	0	8966	30	0	30	948.4	68.5	37.815	
	1	0	14954	0	14954	30	0	30	948.4	47.4	63.07	
	0.1	-1	22472	0	22472	30	0	30	948.4	21	94.779	
	0.01	-2	26528	0	26528	30	0	30	948.4	6.76	111.89	
(Tub.control)	Non	Non	28453	0	28453	30	0	30	948.4	0	120	
code	conc	Log Conc.	RFU2	RFU1	ΔRFU	T2	T1	ΔT	slope	%inhibition	K. Activity	2.88±0.09
7j	100	2	3529	0	3529	30	0	30	948.4	87.6	14.884	
	10	1	11707	0	11707	30	0	30	948.4	58.9	49.376	

	1	0	18537	0	18537	30	0	30	948.4	34.8	78.182	
	0.1	-1	24026	0	24026	30	0	30	948.4	15.6	101.33	
	0.01	-2	26964	0	26964	30	0	30	948.4	5.23	113.72	
**(control)**	Non	Non	28453	0	28453	30	0	30	948.4	0	120	
**code**	**conc**	**Log Conc.**	**RFU2**	**RFU1**	**ΔRFU**	**T2**	**T1**	**ΔT**	**slope**	**%inhibition**	**K. Activity**	
**6h**	100	2	5361	0	5361	30	0	30	948.4	81.2	22.611	**4.039±0.15**
	10	1	11943	0	11943	30	0	30	948.4	58	50.371	
	1	0	19927	0	19927	30	0	30	948.4	30	84.045	
	0.1	-1	23671	0	23671	30	0	30	948.4	16.8	99.836	
	0.01	-2	26754	0	26754	30	0	30	948.4	5.97	112.84	
**(Tub. Control)**	Non	Non	28453	0	28453	30	0	30	948.4	0	120	
**code**	**conc**	**Log Conc.**	**RFU2**	**RFU1**	**ΔRFU**	**T2**	**T1**	**ΔT**	**slope**	**%inhibition**	**K. Activity**	
**7h**	100	2	6617	0	6617	30	0	30	948.4	76.7	27.908	**8.075±0.3**
	10	1	14259	0	14259	30	0	30	948.4	49.9	60.139	
	1	0	21038	0	21038	30	0	30	948.4	26.1	88.73	
	0.1	-1	25909	0	25909	30	0	30	948.4	8.94	109.27	
	0.01	-2	27881	0	27881	30	0	30	948.4	2.01	117.59	
**(Tub. Control)**	Non	Non	28453	0	28453	30	0	30	948.4	0	120	
**code**	**conc**	**Log Conc.**	**RFU2**	**RFU1**	**ΔRFU**	**T2**	**T1**	**ΔT**	**slope**	**%inhibition**	**K. Activity**	
**CA4**	100	2	4492	**0**	4492	30	0	30	948.4	84.2	18.946	**2.786±0.1**
	10	1	11657	**0**	11657	30	0	30	948.4	59	49.165	
	1	0	18535	**0**	18535	30	0	30	948.4	34.9	78.174	
	0.1	-1	22427	**0**	22427	30	0	30	948.4	21.2	94.589	
	0.01	-2	25976	**0**	25976	30	0	30	948.4	8.7	109.56	
**(Tub. Control)**	Non	Non	28453	**0**	28453	30	0	30	948.4	0	120	

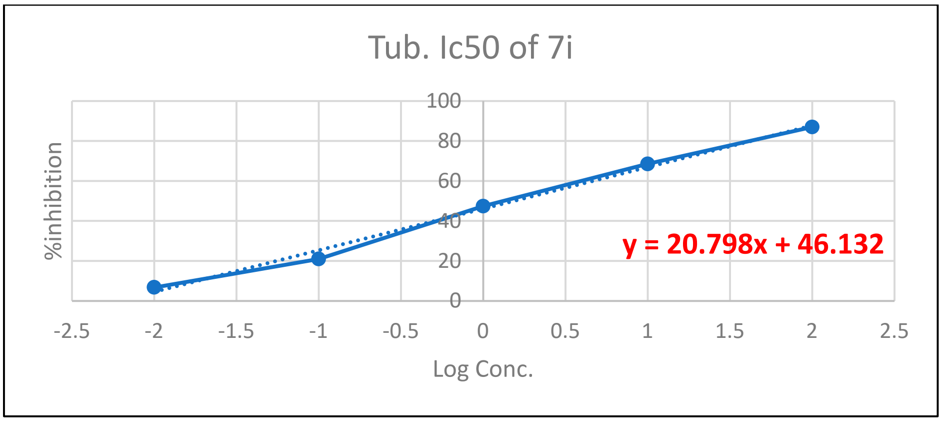
Tub. Ic50 of 7i
%inhibition
Log Conc.
y = 20.798x + 46.132

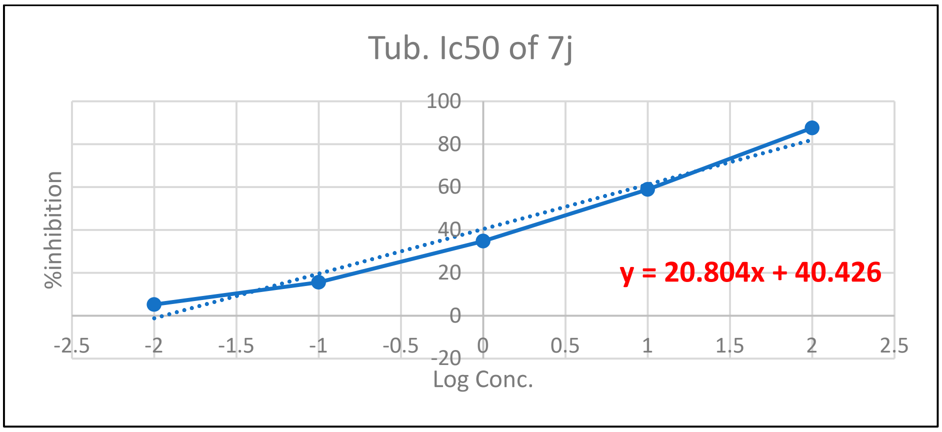
Tub. Ic50 of 7j
%inhibition
Log Conc.
y = 20.804x + 40.426

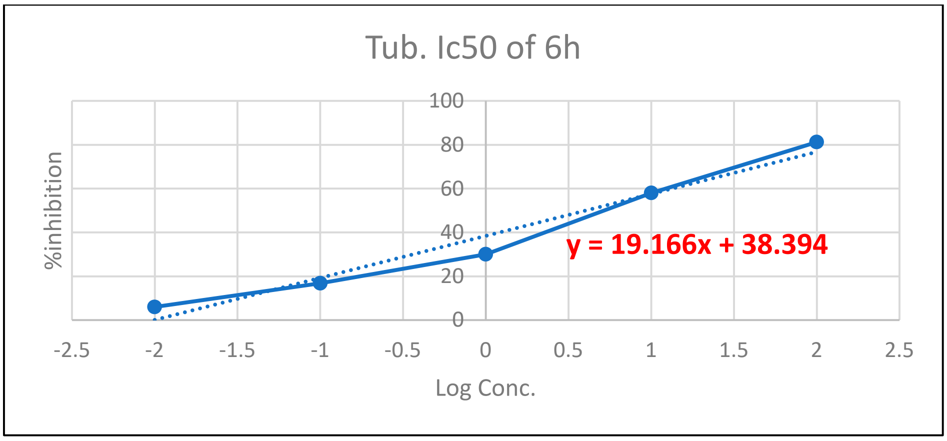
Tub. Ic50 of 6h
%inhibition
Log Conc.
y = 19.166x + 38.394

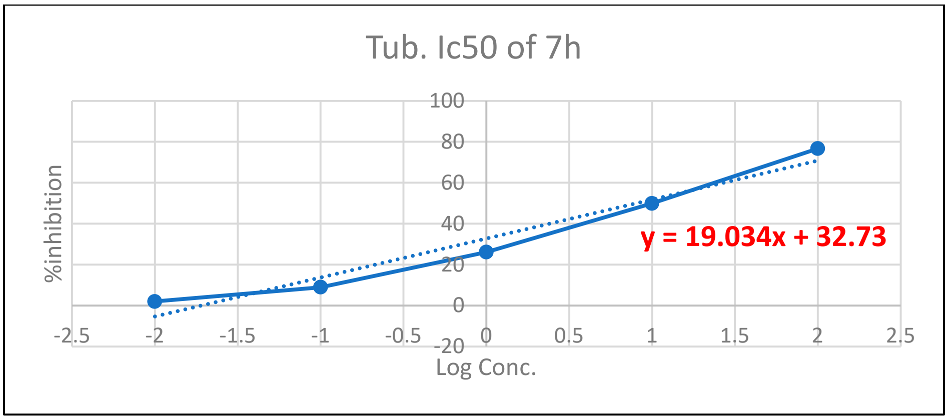
Tub. Ic50 of 7h
%inhibition
100
80
60
40
20
0
-20
-2.5
-2
-1.5
-1
-0.5
0
0.5
1
1.5
2
2.5
Log Conc.
y = 19.034x + 32.73
